# Identification of Conserved and Novel MicroRNAs in the Pacific Oyster *Crassostrea gigas* by Deep Sequencing

**DOI:** 10.1371/journal.pone.0104371

**Published:** 2014-08-19

**Authors:** Fei Xu, Xiaotong Wang, Yue Feng, Wen Huang, Wei Wang, Li Li, Xiaodong Fang, Huayong Que, Guofan Zhang

**Affiliations:** 1 National & Local Joint Engineering Laboratory of Ecological Mariculture, Institute of Oceanology, Chinese Academy of Sciences, Qingdao, China; 2 BGI-Tech, Shenzhen, China; 3 Graduate University of Chinese Academy of Sciences, Beijing, China; Australian Museum, Australia

## Abstract

MicroRNAs (miRNAs) play important roles in regulatory processes in various organisms. To date many studies have been performed in the investigation of miRNAs of numerous bilaterians, but limited numbers of miRNAs have been identified in the few species belonging to the clade Lophotrochozoa. In the current study, deep sequencing was conducted to identify the miRNAs of *Crassostrea gigas* (Lophotrochozoa) at a genomic scale, using 21 libraries that included different developmental stages and adult organs. A total of 100 hairpin precursor loci were predicted to encode miRNAs. Of these, 19 precursors (pre-miRNA) were novel in the oyster. As many as 53 (53%) miRNAs were distributed in clusters and 49 (49%) precursors were intragenic, which suggests two important biogenetic sources of miRNAs. Different developmental stages were characterized with specific miRNA expression patterns that highlighted regulatory variation along a temporal axis. Conserved miRNAs were expressed universally throughout different stages and organs, whereas novel miRNAs tended to be more specific and may be related to the determination of the novel body plan. Furthermore, we developed an index named the miRNA profile age index (miRPAI) to integrate the evolutionary age and expression levels of miRNAs during a particular developmental stage. We found that the swimming stages were characterized by the youngest miRPAIs. Indeed, the large-scale expression of novel miRNAs indicated the importance of these stages during development, particularly from organogenetic and evolutionary perspectives. Some potentially important miRNAs were identified for further study through significant changes between expression patterns in different developmental events, such as metamorphosis. This study broadened the knowledge of miRNAs in animals and indicated the presence of sophisticated miRNA regulatory networks related to the biological processes in lophotrochozoans.

## Introduction

MicroRNAs (miRNAs) are endogenous single-stranded non-coding RNAs measuring ∼22 nucleotides in length, which regulate gene expression at the post-transcriptional level. They are vital components of the RNA-induced silencing complex (RISC) that regulate target genes via complementary binding to their 3′ untranslated regions (3′ UTRs), and leads to either inhibition of translation or mRNA degradation [1], [2]. MiRNAs have been discovered in a wide variety of organisms, including animals, plants, viruses, and even unicellular organisms, and are known to participate in important life processes such as early development, cell proliferation, differentiation, apoptosis, and stress responses [3], [4]. Given their importance in so many developmental processes, researchers have focused much attention on their identification in a wide variety of species. The latest release of the miRNA database (miRBase 20) contains 24,521 hairpin precursors (pre-miRNA) in 206 species (http://www.mirbase.org/) [5]. The species with the most reported miRNAs is *Homo sapiens*, with a total of 1,872 precursors [6]. However, the investigation efforts of miRNAs in Lophotrochozoa have been relatively limited. There are only 461 precursors in this group with 65 in Mollusca, and only a few of these molluscan miRNAs have been studied in detail [7]. Many biological issues, such as the relationships between miRNAs and the evolution of animal genomes, cannot be clarified unless comprehensive data are available for species throughout the animal kingdom.

Although 258 mature miRNAs in pearl oyster *Pinctada martensii* have been reported to be identified with Solexa deep sequencing [8], few supports were provided to meet the miRNA requisite criteria for miRNA annotation proposed in recent reports [6], [9]. This illustrates the problem faced by the miRNA database that some early entries were actually poorly predicted and may be not *bona fide* miRNAs [10], [11]. As a result, relatively stricter annotation criteria based on miRNA biological processing were recently proposed for miRNA annotation in deep sequencing experiment. In animals, the precursor (pre-miRNA) is produced in the nucleus and exported into the cytoplasm. The DICER enzyme then recognizes the stem-loop and cleaves the loop region with two nucleotide overhangs at both 3′ ends [12]. Subsequently, the duplex produced two separate strands (miRNAs from 5p and 3p arms). Because much evidence suggests that both 5p and 3p miRNAs may be biologically functional, high-confidence miRNAs are requested to be supported by reads from both arms of the precursor [5].

In order to identify novel Bivalvia miRNAs and broaden the knowledge of miRNAs in animals, we conducted deep sequencing experiments in the Pacific oyster *Crassostrea gigas*, and annotated the miRNAs according to the recently proposed criteria. *C. gigas* is a relatively well-studied lophotrochozoan that undergoes indirect development [13]. Dramatic changes occur during its larval development. The trochophore is a typical development stage in oyster and many other lophotrochozoans. The larvae later develop into phylotypic stages, such as the veliger in molluscs [14]. The oyster also changes from the planktonic larval stage to the sessile adult stage through metamorphosis, a complex process requiring intricate gene regulation mechanisms. Several studies have focused on the biological regulation processes at the gene, mRNA, or protein levels [15], [16]. However, no study focused on the role of miRNA regulation during development has yet been reported in molluscs. The recent release, sequenced with the next generation technique, of the complete genome assembly of *C. gigas* [17] serves as an excellent reference for genome wide identification of the oyster's miRNAs. It also allows the analysis of characteristics of miRNA regulation in *C. gigas* at the whole genome level. Therefore, in the current study, through deep sequencing, we identified the miRNAs of *C. gigas* and then determined their expression patterns during different developmental stages and in different organs. Our findings not only extend the repertoire of lophotrochozoan miRNAs, but also provide further insights into the function and the evolution of miRNAs.

## Results and Discussion

### Profiles of oyster miRNA

An average of 14.08 million reads (from 10.75 M to 21.82 M) was obtained using 21 libraries (Tables S1 & S2 in File S1). Among the reads that could be mapped onto the genome, an average of 52.14% was predicted to be miRNAs. But only 0.90% was predicted in eggs, in comparison with the 87.05% in the adult adductor muscle (Table S3). The low proportion of miRNAs in the egg maybe due to the abundance of piwi-interacting RNAs (piRNAs) detected in the egg or that it contains the largest proportion of expressed repeat elements of all the developmental stages. A total of 100 loci were predicted to encode pre-miRNAs that produce *bona fide* miRNAs in the Pacific oyster genome (Tables 1, S4 & S5), *i.e.*, which met the requisite criteria for miRNA annotation proposed in recent reports [6], [9]. These miRNAs represent most of the families identified in related taxa (Table S6). The average length of these precursors was 79 bp (71–91 bp). All of the precursors were supported by mature sequences from both arms of the hairpin. Most of the miRNAs were confirmed by more than two experiments and the read number was >100 in at least one single experiment. All of the candidate precursors could be mapped onto only one site in the genome except for the *cgi-miR-124*, and they all had at least 16 nucleotides (nt) that were complementary between the guide miRNA and the star miRNA region. At the same time, some predicted loci that folded well, but failed to meet the criteria (e.g. too few reads), are also provided in the supplementary information (Table S7). One of these candidate loci, *mir-184*, had as many as 67 hits in BLAST searches of the oyster genome (Table S8), and produced 14 types of miRNAs (Figure S1 in File S1). These hits located two loci: 718110∼722565 bp of scaffold121, and 63164∼67661 bp of scaffold43150. Further studies are needed to confirm the existence and number of *mir-184* loci in oyster genome, we thus do not analysis this miRNA in this study.

**Table 1:** Number of predicted precursors for each library.

Sample	Number	Sample	Number	Sample	Number
m01.Early	98	s05.D	100	t02.Mai	87
m02.Late	99	s06.U	98	t03.Dgl	91
m03.Adult	89	s07.P1	95	t04.Gil	84
s01.E	79	s08.P2	93	t05.Amu	84
s02.B	93	s09.S	90	t06.Hem	89
s03.T1	99	s10.J	93	t07.Lpa	88
s04.T2	98	t01.Mao	86	t08.Fgo	89

Note: The precursors with more than ten sequenced reads from the mature miRNA of 5p or 3p arm were counted.

### Genomic context of oyster miRNAs

Based on our results, in oyster, a total of 49 precursors (49%) were located in intronic regions. This proportion was comparable with that in vertebrates and fruit fly *Drosophila melanogaster* (39–70%) [18], [19] but was higher than that in the silk worm *Bombyx mori* (9%) [20], the nematodes *Caenorhabditis elegans* (17%) [19] and *Brugia pahangi* (16%) [21]. Two precursors overlapped with the *in silico*-predicted exons, but were poorly supported by EST or homology genes. Thus, we manually checked the gene models and found that these two precursors were actually non-exonic (Figures S2 & S3 in File S1). As a result, all of the precursors were intronic, with 38 (38%) located in the sense strand and 11 (12%) located in the antisense strand. We divided the whole oyster genome sequence into contiguous windows based on the average length of the precursors (79 bp). The frequency of windows located within the gene region on the sense strand was significantly less (20%, *P*<0.01) than that of the precursors. This indicated that oyster miRNAs were preferentially located in intragenic regions rather than intergenic regions. Furthermore, the distribution of miRNAs was not random throughout the genome. We observed that most of the 49 intergenic precursors were far away from gene regions. When we extended the frames by 500 bp, 1,000 bp, or 2,000 bp upstream and downstream of the precursors, only five additional precursors overlapped with gene regions and the proportion of intragenic sequences around precursors increased slightly from 49% to 54%. In contrast, the percentage of contiguous windows in the divided genome overlapping gene regions increased from 41% to 61% at 1,079 bp (500 bp upstream plus 79 bp windows plus 500 bp downstream), 2,079 bp, and 4,079 bp lengths (Figure S4 in File S1). The intronic distribution for intragenic precursors and the distance from gene regions of intergenic precursors indicated that the physical distribution of oyster miRNA was constrained by some undefined biological mechanisms. Indeed, some characteristics of physical location, such as intragenic distribution and clustering, are common in the genomes of many organisms [19]. Such constraints may allow miRNAs to be transcribed efficiently within the same primary miRNA (pri-miRNA) and may also be important during miRNA genesis [22], [23]. However, reasons for intergenic miRNAs to be distant from gene regions have seldom been discussed. For this study, there may be a bias since only a few intergenic miRNAs were assayed. It is also possible that this observation might due to the biogenesis mechanism of miRNAs, which starts with the transcription of several hundred nucleotides of pri-miRNAs. The miRNA genes may have integrated coding and regulatory regions, as is the case for protein coding genes [24], thereby causing a requirement for increased distance between precursors and neighboring genes.

The clustering of miRNAs [23] was also common in the oyster genome. In this study, we defined contiguous precursors with a maximum gap of 10 Kbp as a cluster. A total of 53 precursors (53%) formed 20 clusters (Table S9 in File S1). Forty eight precursors (91% of the 53) from 18 clusters encoded conserved miRNAs. Most of these conserved precursors were duplicated, such as the *mir-9* and *mir-2* families. For the two clusters encoding novel miRNAs (clusters 19 & 20 shown in the Table S9 in File S1), the sequences of the clustered mature miRNA were different, indicating their different origin. Indeed, miRNAs from different families that clustered together have also been observed in other organisms, such as the conserved mir-17-92 cluster in vertebrates [25], [26]. Therefore, we speculate that there might be an underlying mechanism for selecting novel miRNAs in particular hotspot regions throughout the genome. According to the accidental origin hypothesis, which refers to stem-loop-like sequences as one of the most common local structures in the genome [26], if a novel miRNA evolved from a stem-loop-like sequences, it might be easier for its neighbor stem-loop-like sequences to be co-transcribed and spliced into precursors than other sites. Clustered precursors with distinct sequences may have evolved in this way.

Intragenic miRNAs are generally neither located in the same gene in different species, nor co-expressed at the same level as the host gene [27]. In most animals studied, *mir-2* is located in the intergenic region. However, in oyster, one cluster containing six *mir-2* was located in the intronic region (Table S4) of the *protein phosphatase 4, regulatory subunit 1* (*PPP4R1*, CGI_10023188) gene. The other *mir-2* was located in the intronic region of the gene *structural maintenance of chromosomes 4* (*SMC4*, CGI _10009524). In *Caenorhabditis elegans*, there is only one *mir-2* but it is also located in the intron of the *ppfr-1* gene, an ortholog of the *PPP4R1* gene. However, the cluster of *mir-2* in *Drosophila melanogaster* is located in the intron of the gene *spitz* while another member of the *mir-2* family, *mir-13*, is located in the intron of the *CG7033* gene. This suggests that the position of *mir-2* intronically embedded in *PPP4R1* is conserved between the oyster and *C. elegans*, and may represent the genomic organization of their common ancestor. The expansion of *mir-2* occurred by tandem duplication during evolution [28]. Furthermore, the expression patterns of the six *mir-2* in the intron of *PPP4R1* (CGI_10023188) had a similar expression pattern to the host gene (the largest Pearson correlation coefficient *r* = 0.93 for the six *mir*-2, *P* = 6.06E-08, Table S10), which supported a previous report that miRNAs were co-expressed with their host genes [29]. However, the co-expression pattern was not common for all the 49 oyster intronic precursors (Table S10). Taking the *cgi-miR-33* (m0361_5p) for example, the host gene *Sterol regulatory element-binding transcription factor 1* (*SREBF-1*, CGI_10004108) only showed a weak co-expression pattern (*r* = 0.58, *P* = 0.02) with this miRNA. Actually, strong co-expression relationships (*r*>0.8, *P*<0.0001) of the host genes and their intragenic miRNAs were detected only in 21 of the 76 pairs (Table S10). This result suggests that parallel transcription of miRNAs with their host gene might not result in the similar co-expression patterns of the mature mRNA and miRNA. Plausible mechanisms to explain the abundance differences of the miRNA and mRNA of the host gene can be proposed. Firstly, the difference in the biogenesis pathways between miRNA and mRNA could have an influence. Different proteins regulate the maturation steps for these two type of RNAs, thus resulting in differential biosynthesis duration after transcription. Secondly, the RNAseq-detected relative abundance ratio of the free molecules might not be the real abundance ratio of mRNA and miRNA in cell. The miRNA abundance can be controlled by binding multiple mRNAs [30] which share common miRNA regulatory elements (MRE) [31]. The bound miRNAs will not be isolated and sequenced by the RNAseq technique. In addition, the degradation system of the miRNA and mRNA could be controlled by different mechanisms, resulting in the abundance ratio inconsistency in different samples. The mRNA can be regulated by miRNA, siRNA and some other molecules, while miRNA may be regulated by circular RNAs (circRNAs) [32]. This will lead to different longevity in the cell for the two types of molecules, thus resulting in the non-linear correlation of the expression level of miRNA and the host gene mRNA.

**Figure 1:**
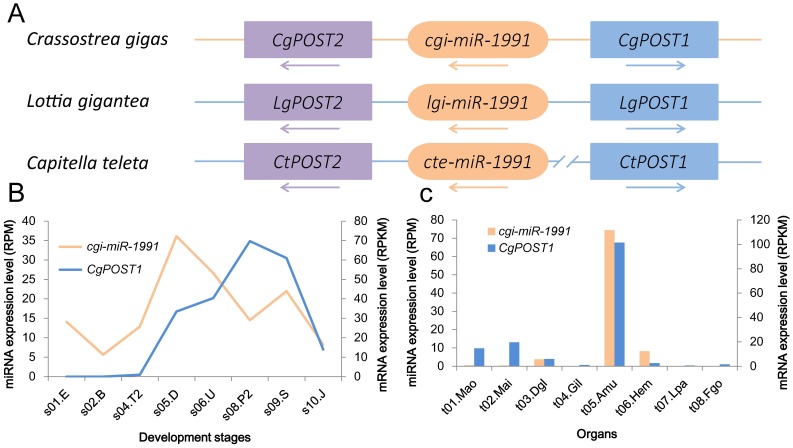
Genomics and expression of *mir-1991* and the Post gene cluster. **A**. Location of the *mir-1991* in the *Post* clusters in *Crassostrea gigas*, *Lottia gigantea* and *Capitella teleta*. Arrow shows the strand orientation of the gene. **B**. The expression pattern of oyster miRNA *cgi-miR-1991* and oyster gene *CgPOST1* in different development stages. The samples are abbreviated as follows: s01.E, egg; s02.B, blastula; s04.T2, trochophore; s05.D, D-shape larva; s06.U, umbo larva; s08.P2, pediveliger larva; s09.S, spat; s10.J, juvenile. c. The expression pattern of oyster miRNA *cgi-miR-1991* and oyster gene *CgPOST1* in different organs. The samples are abbreviated as follows: t01.Mao, outer edge of mantle along the margin of the shell; t02.Mai, inner pallial part covering the inner surface of the shell; t03.Dgl, digestive gland; t04.Gil, Gill; t05.Amu, adductor muscle; t06.Hem, hemolymph; t07.Lpa, labial palps; t08.Fgo, female gonad.

In addition, the *cgi-miR-1991* embedded between genes *CgPost2* and *CgPost1* in the Hox cluster (Figure 1A). The *mir-1991* was assigned into the *mir-10* family recently because their seed sequences are identical [33]. The *mir-10* genes are also found within the Hox cluster and regulate the Hox genes expression level [34], indicating that the Hox cluster embedded miRNA family should have deep origin and the function maybe keep conserved. In this study, the *cgi-miR-1991* showed significant co-expression with its neighbor gene *CgPost1* (*r* = 0.81, *P* = 9.37E-05. Figure 1B). The result indicated that there could be some interaction between *cgi-miR-1991* and the *CgPost1*. Furthermore, the location of *mir-1991* is conserved in the three neotrochozoans *C. gigas*, *Lottia gigantea* and *Capitella teleta* (Figure 1A), suggesting that function may have been conserved in these very different animals.

### Expression pattern of oyster miRNAs

A total of 81 precursors produced mature miRNAs with significantly different abundance between the 5p and 3p arms (the reads number difference was greater than five fold). Thus, we assigned the mature miRNA with the larger reads number as the guide miRNA. However, several precursors were sequenced with comparable reads for the mature miRNAs from both the 5p and 3p arms. Because much evidence suggests that both 5p and 3p miRNAs may be biologically functional, we retained most of the mature miRNAs for further expression pattern analysis except for those having only few reads were sequenced (less than 50). In total, 180 mature miRNAs were selected (Table S11) to analyze expression patterns and the possible biological process they may involved.

**Figure 2:**
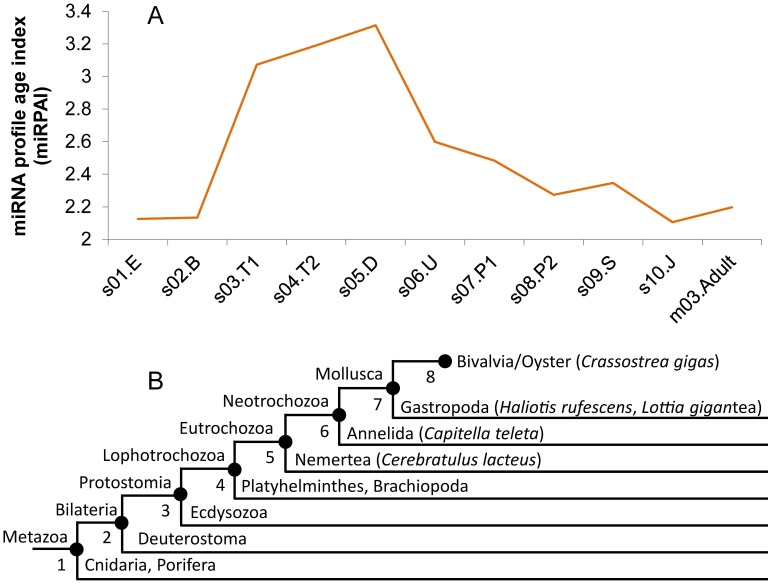
The miRPAI curve during oyster development. **A**. The miRPAIs for the 11 development stages were calculated by integrating the evolutionary acquisition (phylostrata) and the expression level of each miRNA (see the method part for details). Higher miRPAI indicates more expression of younger miRNAs in the stage. The samples are abbreviated as follows: s01.E, egg; s02.B, blastula; s03.T1, trochophore; s04.T2, trochophore; s05.D, D-shape larva; s06.U, umbo larva; s07.P1, pediveliger larva; s08.P2, pediveliger larva; s09.S, spat; s10.J, juvenile; and m03.Adult, mixture of tissues from an adult. **B**. The taxonomic nomenclature of the phylostrata analyses.

Evolutionary acquisition was assigned to oyster mature miRNAs (Tables S4, S5 & S11) based on the literature [33] and sequence homology with records in miRBase [6]. Most of the assignments were to Bilateria (80 miRNAs from 45 precursors) and Protostomia (43 from 22 precursors), as well as novel oyster miRNAs (31 from 19 precursors). The expression patterns were significantly different among different groups of miRNAs. The deeply evolved Bilateria and Protostomia miRNAs had low expression levels from the trochophore to veliger stages, whereas those of Eutrochozoa and younger miRNAs were highly expressed during this development period. As a result, the miRNA profile age index (miRPAI) from the trochophore to veliger stages was higher than that at other stages (Figure 2A). The trochophore is the main characteristic of Trochozoans [35] and the veliger larvae are thought to correspond to the phylotypic stage in Mollusca [14]. The transition from the trochophore to the D-shaped veliger larvae when most of the organs form within about 10 hours is probably the most dramatic event during oyster development. Therefore, novel miRNAs may play an important role in novel body plan development during this stage. In this context, the age of the miRNAs profile was younger during this ontogenetic period than others.

To further analyze the spatiotemporal expression patterns of the 21 samples, we assessed the expression specificity and the coefficient of variation (*CV*). If the reads of a miRNA were only sequenced in a few samples or the variation of expression level was high among different samples, the miRNA was considered to be specially expressed in certain development stages or organs, and may indicate the specialization of its biological function. We found that miRNAs with a deeper origin were detected in more samples while the variation of the expression level (*CV* of RPM, reads per million) was lower than oyster novel miRNAs (Figures S5 & S6 in File S1). The results indicated that conserved miRNAs functioned in a more global pattern whereas the novel ones were more specific. It has been reported that the role of a miRNA may not be only restricted to the homologous tissues of different organisms, but could also be co-opted by new tissues with time [36], [37]. This might explain why deeper evolved miRNAs were involved in more global processes. Meanwhile, the younger or species-specific novel miRNAs may be more differentially-expressed and should be a better choice for tissue/organ markers.

**Figure 3:**
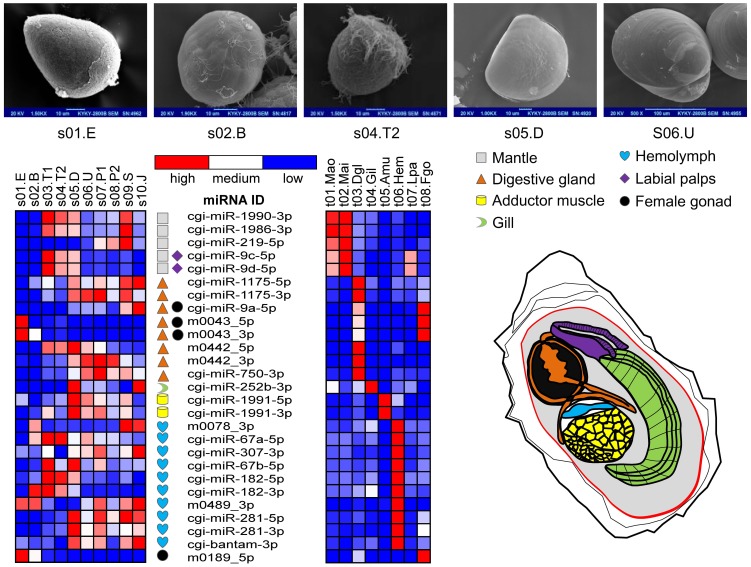
Organ-specific expressed miRNAs and their expression patterns during different development stages and in different organs. The abbreviations of organs and developmental samples in each heat map are as detailed in the legend to Figure 1. A color bar is provided where blue indicates a low expression level, red indicates a high expression level, and the white indicates medium expression levels. The electron microscope (EM) photos show part of the development stages and there is also a sketch of the main adult organs. The EM photos for s04.T2, s05.D, and s06.U were from Zhang *et. al.* [17]. The organs are indicated with different colors according to the scheme shown in the sketch while they are also indicated in front of the miRNA IDs.

The specific expression patterns of some miRNAs reflect their potential involvement in regulatory processes in certain developmental or organ-functional events. The miRNAs with significant expression patterns would be potential key candidates for further study on their novel biological functions. For example, the oyster-specific *cgi-miR-1990-3p* showed a mantle-specific expression pattern, while the *cgi-miR-1991-5p* was specifically expressed in the adductor muscle (Table S12 in File S1 & Figure 3). Meanwhile, some miRNAs were highly expressed during the pediveliger stages (s07.P1 or s08.P2) when the oyster was competent for metamorphosis, including *cgi-miR-33-5p*, an intragenic miRNA located in the intron of gene *sterol regulatory element-binding transcription factor 1* (*SREBF-1*, CGI_10004108), which indicated the potential regulation of *cgi-miR-33-5p* during metamorphosis. Interestingly, its homologs in humans, *hsa-miR-33a-5p* and *hsa-miR-33b-5p*, are also located in *SREBF-2* and *SREBF-1*, respectively, and they have been reported to control the insulin signaling pathway [38] and fatty acid metabolism in cooperation with the host gene [39]. The products of the SREBF family are post-translationally activated in situations with reduced lipid abundance and are responsible for cholesterol, fatty acid, and phospholipid synthesis, while miR-33 reduces the degradation of these materials by inhibiting the related genes *ATP-binding cassette*, *sub-family A*, *member 1* (*ABCA1*), and *carnitine palmitoyltransferase 1A* (*CPT1A*) [40]. The conserved sequence and physical location of miR-33 in oyster suggested that it may have similar biological functions and may be involved in metabolism regulation during metamorphosis.

## Conclusions

Deep sequencing is an efficient method to study the evolution and developmental function of miRNAs in Lophotrochozoa, which is a poorly studied bilaterian clade. In the current study, using a comprehensive sample collection strategy and experimental design, we identified a large number of miRNAs in *C. gigas* and we also produced a miRNA expression profile that would provide new insights into the function of these miRNAs. Many novel miRNAs were identified in the oyster *C. gigas* by this study. The presence of miRNA clusters and the co-location of miRNAs with mRNA suggested two important biogenesis sources of miRNAs. The different organs and developmental stages were characterized by specific miRNA expression patterns, which highlights regulatory variation in the spatiotemporal levels. The novel miRNAs tended to be more specific to certain developmental stages or organs. The high level expression of younger miRNAs from trochophore to D-shape larvae suggested the specificity of this typical developmental stage in Eutrochozoans.

## Materials and Methods

### Experiment design and sample preparation

An adult 2–3-year old wild individual Pacific oyster *C. gigas* was dissected. Organs samples were taken from the outer edge of mantle along the margin of the shell (t01.Mao), the inner pallial part covering the inner surface of the shell (02.Mai), digestive gland (t03.Dgl), gills (t04.Gil), adductor muscle (t05.Amu), hemocyte (t06.Hem), labial palp (t07.Lpa), female gonad (t08.Fgo), and a mixture of the organs (m03.Adult). These samples were used for genome resequencing and transcriptome sequencing, as described in a previous study [17]. The oysters sampled at different developmental stages were F_2_ mass-mating progeny. Their parents were 51 females and one male from the “G3” family. The progeny were discussed in detail in a previous report [17]. A total of 250 million zygotes were dispersed into a pool of sand-filtered seawater maintained at 26°C, with a salinity of 30 and a volume of about 25 m^3^. The samples used for small RNA sequencing included an egg sample (s01.E), 14 samples (including embryonic and partial non-feeding larval stages) collected at an average interval of about 2 h for the first 24 h (m01.Early, s02.B, s03.T1, s04.T2, s05.D), 11 larval samples collected at an average interval of 1.6 days for 18 subsequent feeding days (m02.Late, s06.U, s07.P1, s08.P2), one spat sample collected at day 22 (s09.S, also mixed with the library m02.Late), and one juvenile sample collected at day 215 (s10.J).

We constructed 21 small RNA libraries, including three libraries using mixed samples (prefixed with “m” in the sample IDs), 10 libraries using samples from nine typical developmental stages (egg, blastula, trochophore, D-shape, umbo, pediveliger, spat, and juvenile, which were prefixed with “s” in the sample IDs), and eight libraries using samples from adult organs (prefixed with “t” in the sample IDs). Additional details are shown in Table S1 in File S1. These samples included the major organs and developmental changes of oyster, such as embryogenesis, morphogenesis, shell genesis, and metamorphosis.

The samples used for scanning electron microscopy were fixed with 5% glutaraldehyde in PBS for 3 h at room temperature. The fixed samples were rinsed twice with 0.1 M cacodylate buffer (pH 7.2, 1,000 milliosmoles) and dehydrated using a graded acetone series, before drying with carbon dioxide at the critical point. The samples were then sputter-coated with gold and observed at 20/25 kV using a KYKY-2800B scanning electron microscope. The developmental stages were classified according to the literature [17].

### Small RNA sequencing

Total RNA was extracted using TRIzol reagent (Invitrogen, Gaithersburg, MD), according to the manufacturer's instructions. For smRNA-seq, we gel purified 18–30 nt RNAs from the samples. Illumina 5′ and 3′ RNA adapters were sequentially ligated to the RNA fragments and the ligated products were selected by size on denaturing polyacrylamide gels. The adapter-linked RNA was reverse transcribed with small RNA RT primers and amplified by 15 PCR cycles with small RNA PCR primers 1 and 2 (Illumina). The libraries were sequenced with the Illumina genome analyzer HiSeq 2000. After masking the adaptor sequences and removing reads with excessively small tags or from contaminating adapter-adapter ligation, the clean reads were processed for computational analysis.

### Small RNA read alignment

Small RNAs that measured no less than 18 nt with average single base error rates of <0.01 were retained. The high quality reads were determined according to the following criteria: no more than four bases with a quality score <10 and no more than six bases with a quality score <13. The high quality clean small RNA reads were mapped to the *C. gigas* genome using SOAP [41]. We excluded small RNAs that mapped to annotated exons, repeats, rRNAs, tRNAs, or snRNAs [17]. The remaining reads were retained for further analysis [42].

### miRNA prediction, homology analysis

To identify potential miRNA genes, we scanned the *C. gigas* genome for pre-miRNA structures to obtain all candidate precursors using MIREAP [43], which was specially designed to identify miRNAs from deeply sequenced small RNA libraries [43], [44]. The outputs were then manually checked according to the requisite criteria for miRNA annotation proposed in recent reports [6], [9]. In brief, miRNAs meeting the following criteria were retained: 1. Precursors only mapped onto a single locus of the genome; 2. The minimum free energy for the precursor secondary structure should be lower than −21 kcal/mol; 3. The 5′-end of the major mapped reads should be consistent; 4. Mature miRNAs were sequenced from both arms (at least 50 total mature miRNA reads were sequenced from all the samples); 5. The mature sequences from both arms should have at least 16 nt complementary; 6. The mature miRNAs either conserved in widely-diverge taxa or meeting the 2 nt overhang criterion for the mature sequences from both arms. The secondary structure drawing and reads quantitation was conducted with MiRDeep2 [45], and was provided in supplementary File S2. We calculated the expression values of miRNAs using reads per million (RPM) and identified differentially expressed miRNAs in *C. gigas* [46]. In the homology analysis, the predicted oyster mature miRNAs were used to search for known miRNAs in miRBase version 20 (http://www.mirbase.org/) using BLASTN. The minimal mature miRNA identity was set as 0.8 for conserved miRNAs, while the seed (2–8 nt) was required to have 100% conservation. Then the evolutionary acquisition (phylostratigraphic levels) was assigned to the conserved mature miRNAs based on the literature [33]. To further analyze the evolution of miRNAs of Lophotrochozoa, subgroups of this taxon were used, i.e. the groups Eutrochozoa and Neotrochozoa [7]. The taxonomic nomenclature used in this article is shown in Figure 2B.

### Correlation and coefficient of variation analysis

The RNAs from 16 samples in this study (s01.E, s02.B, s04.T2, s05.D, s07.P2, s09.S, s10.J, t01.Mao, t02.Mai, t03.Dgl, t04.Gil, t05.Amu, t06.Hem, t07.Lpa, and t08.Fgo) were also used for RNA-seq in a previous study [17], so the miRNA expression patterns (RPM value) and the mRNA expression patterns (reads per kilobase of exon per million mapped reads, RPKM) of the related genes were available here. We calculated Pearson's correlation coefficient (*r*) for the expression patterns of the mature miRNA and the host genes, as well as the *P* value. If the *r* value was >0.8 (*P*<0.0001), the expression pattern was considered to have a significantly positive correlation (co-expression), *i.e.*, if the host gene was expressed at a higher level in one sample compared with other samples, the harbored miRNA had a correspondingly higher expression level than the others. At the same time, the coefficient of variation (*CV*) is the standard deviation of RPM value expressed as a fraction of the mean. It can be used to reflect the stability of miRNA expression in different samples.

### miRNA profile age index (miRPAI) calculation

The miRNA profile age index (miRPAI) was calculated by referring to the principle of transcriptome age index (TAI) [47], [48]. In brief, the miRPAI for each ontogenetic stage was calculated using the formula 
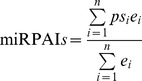
, where ps*_i_* is the evolutionary acquisition (phylostratum) of the miRNA *i* (a higher value of ps*_i_* indicates a younger miRNA), *e_i_* is the read number obtained by Illumina sequencing of miRNA *i*, and *n* is the total number of miRNAs analyzed. As a younger miRNA was assigned a larger phylostratum age, a higher miRPAI value indicates a younger miRNA profile age.

### Other statistic methods

The chi-square test was used to calculate the significance of the intragenic proportion difference between miRNA and artificially divided whole genome contiguous windows. The R software was used in statistical computing and boxplot drawing. A heat map was constructed using the HeatMapViewer modules in GenePattern (http://genepattern.broadinstitute.org). The organs in which miRNAs were specifically expressed or absent were identified by screening the RPM values with the following thresholds: for specifically-expressed miRNAs, the RPM value of the miRNA had to be five-fold or higher than in any other sample; for the specifically lowly expressed miRNAs, the RPM value of the miRNA had to be one fifth or less than that in any other sample. The miRNA expression levels in the mantle were calculated by averaging the RPM in t01.Mao and t02.Mai, which were two different parts dissected from the whole mantle.

### Accession numbers

The raw sequencing data was deposited in the NCBI Gene Expression Omnibus (GEO, http://www.ncbi.nlm.nih.gov/geo) with the accession number of GSE31009

## Supporting Information

Table S3 **Reads annotation results for different RNA categories.**
(XLSX)

Table S4 **Details of the 81 conserved precursors and their mature miRNAs.** This table shows the following information for the 81 conserved precursors and the mature miRNAs: precursor ID; mature miRNA ID; homologs in the miRBase v20; miRNA family information; the predicted free energy of folding; sequence and secondary structure of the hairpin precursor; sequence of the predicted mature miRNA; physical location of the hairpin precursor based on the oyster genome version 1 (scaffold ID, start site, end site, and strand); miRNA cluster information (a cluster was defined as having a maximum gap of 10 Kbp between contiguous precursors); miRNA age and phylostratum (evolutionary acquisition, see the main text); the gene ID that overlapped with the miRNA and the strand information; read number and the RPM value for each miRNA in each sample.
(XLSX)

Table S5 **Details of the 19 predicted novel oyster precursors and their mature miRNA products.** This table shows the following information for the 19 predicted novel oyster precursors and their mature miRNAs: precursor ID; mature miRNA ID; the predicted free energy of folding; sequence and secondary structure of the hairpin precursor; sequence of the predicted mature miRNA; physical location of the hairpin precursor based on the oyster genome version 1 (scaffold ID, start site, end site, and strand); miRNA cluster information (a cluster was defined as having a maximum gap of 10 Kbp between contiguous precursors); miRNA age and phylostratum (evolutionary acquisition, see the main text); the gene ID that overlapped with the miRNA and the strand information; read number and the RPM value for each miRNA in each sample.
(XLSX)

Table S6 **The family distribution of conserved oyster miRNAs.**
(XLSX)

Table S7 **Details of the 27 potential oyster precursors and their mature miRNA products.** This table shows the following information for the 27 potential oyster precursors and their mature miRNAs: precursor ID; mature miRNA ID; the reason excluding the corresponding precursor from oyster miRNA gene set; the predicted free energy of folding; sequence and secondary structure of the hairpin precursor; sequence of the predicted mature miRNA; physical location of the hairpin precursor based on the oyster genome version 1 (scaffold ID, start site, end site, and strand); miRNA cluster information (a cluster was defined as having a maximum gap of 10 Kbp between contiguous precursors); the gene ID that overlapped with the miRNA and the strand information; read number and the RPM value for each miRNA in each sample.
(XLSX)

Table S8 **The BLAST hits of** ***mir-184*** **in the oyster genome.**
(XLSX)

Table S10 **The Pearson correlation coefficient between miRNA expression and host genes.** The expression level of the host genes were from the literature and were measured with RPKM (Zhang et. al. 2012). The RNAs used for miRNA sequencing in this study and those used in the RNA sequencing in the previous report were from the same samples. The correspondence of the sample names in this study and the previous report are as follows: s01.E_RPM - E_RPKM, s02.B_RPM - B_RPKM, s04.T2_RPM - T2_RPKM, s05.D_RPM - D1_RPKM, s06.U_RPM - U2_RPKM, s08.P2_RPM - P1_RPKM, s09.S_RPM - S_RPKM, s10.J_RPM - J_RPKM, t01.Mao_RPM - Man1_RPKM, t02.Mai_RPM - Man2_RPKM, t03.Dgl_RPM - Dgl_RPKM, t04.Gil_RPM - Gil_RPKM, t05.Amu_RPM - Amu_RPKM, t06.Hem_RPM - Hem_RPKM, t07.Lpa_RPM - Lpa_RPKM, t08.Fgo_RPM - Fgo_RPKM.
(XLSX)

Table S11 **The 180 miRNAs chosen for analyzing expression patterns.**
(XLSX)

File S1 Contains Figures S1–S6 and Tables S1, S2, S9, and S12.
(DOC)

File S2 **The compressed/ZIP file archive for the predicted precursors' secondary structures and reads alignment.**
(ZIP)
