# Supplementary material for: Identification of Conserved and Novel MicroRNAs in the Pacific Oyster Crassostrea gigas by Deep Sequencing
Source: PLoS One. 2014 Aug 19;9(8):e104371. doi: 10.1371/journal.pone.0104371 (PMC4138081; doi:10.1371/journal.pone.0104371)
Supplement: File S1 — Contains Figures S1–S6 and Tables S1, S2, S9, and S12. (DOC) [file pone.0104371.s009.doc]

**Additional tables for**

**Identification of conserved and novel microRNAs in the Pacific oyster *Crassostrea gigas* by deep sequencing**

Fei Xu^1^, Xiaotong Wang^1^, Yue Feng^2^, Wen Huang^1,3^, Wei Wang^1^, Li Li^1^, Xiaodong Fang^2^, Huayong Que^1^, Guofan Zhang^1#^

^1^ National & Local Joint Engineering Laboratory of Ecological Mariculture, Institute of Oceanology, Chinese Academy of Sciences, Qingdao 266071, China.

^2^ BGI-Tech, Shenzhen 518083, China.

^3^ Graduate University of Chinese Academy of Sciences, Beijing 100049, China

#Corresponding authors: G.Z. (gzhang@qdio.ac.cn)


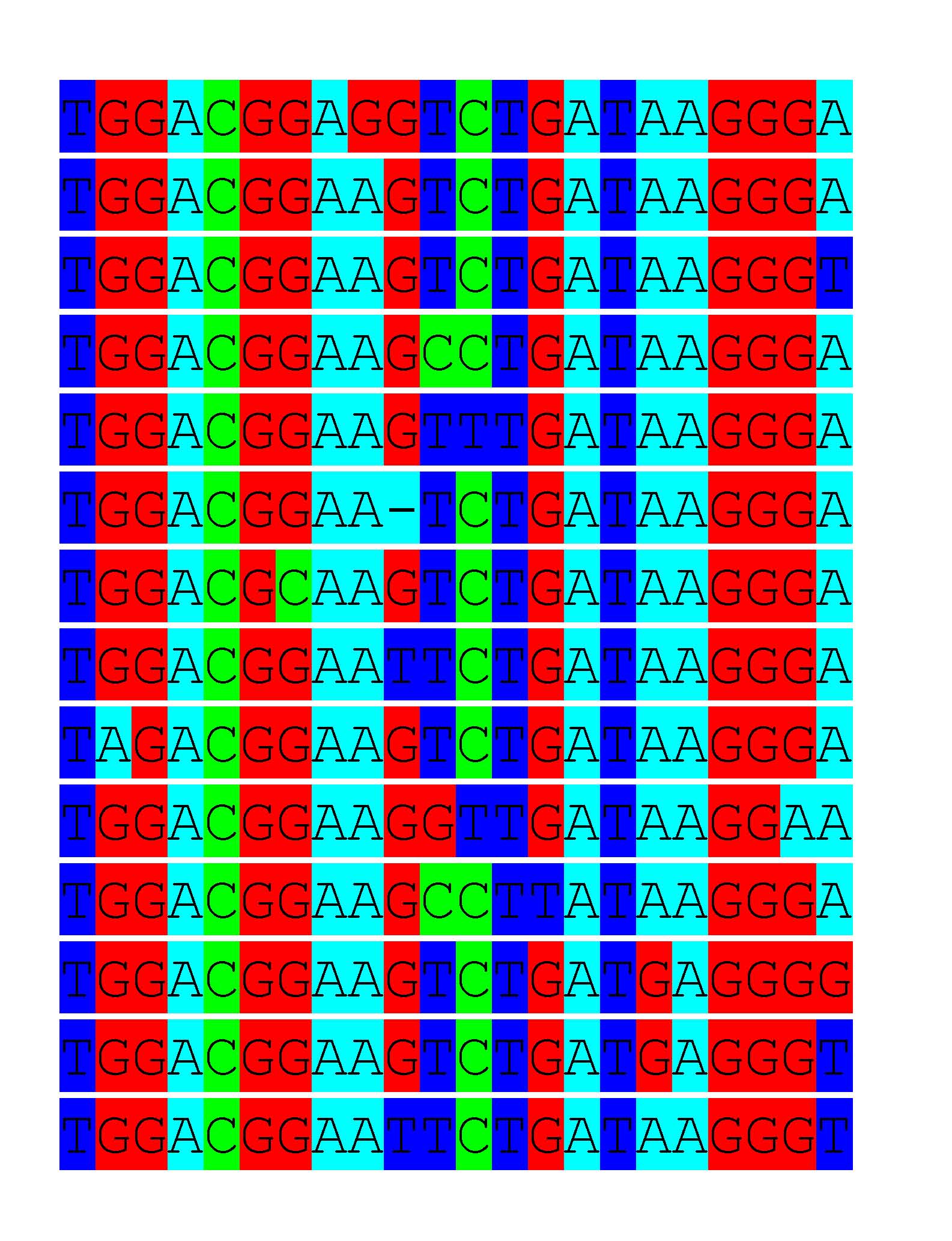


Figure S1 | The alignment of possible oyster mature *mir-184*.


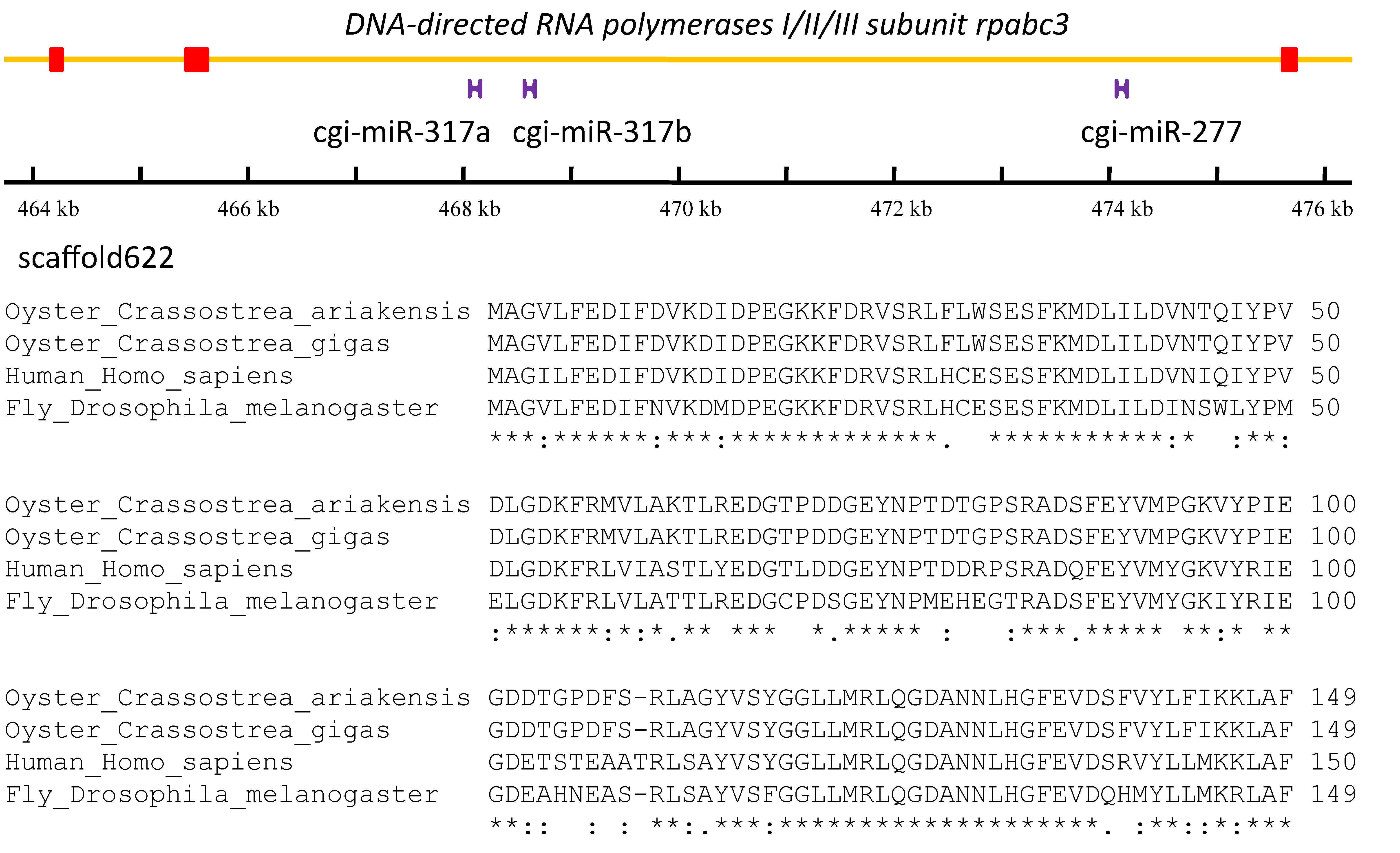


**Figure S2 | Manually adjusted model of the gene CGI_10018220 (*DNA-directed RNA polymerases I/II/III subunit rpabc3*) and the distribution of *cgi-miR-317a*, *cgi-miR-317b*, and *cgi-miR-277*.**

The purple bars indicate the miRNAs. The yellow lines show the introns whereas red blocks show the exon regions. The sequences below are the amino acid product sequences of the gene from the Pacific oyster *Crassostrea gigas* and homologs from the Jinjiang oyster *C. ariakensis*, Human *Homo sapiens*, and fruit fly *Drosophila melanogaster*. The alignment shows that this gene is highly conserved across the Bilateria, which suggests that the newly adjusted gene model is more credible. Thus, the miRNAs are intronic.


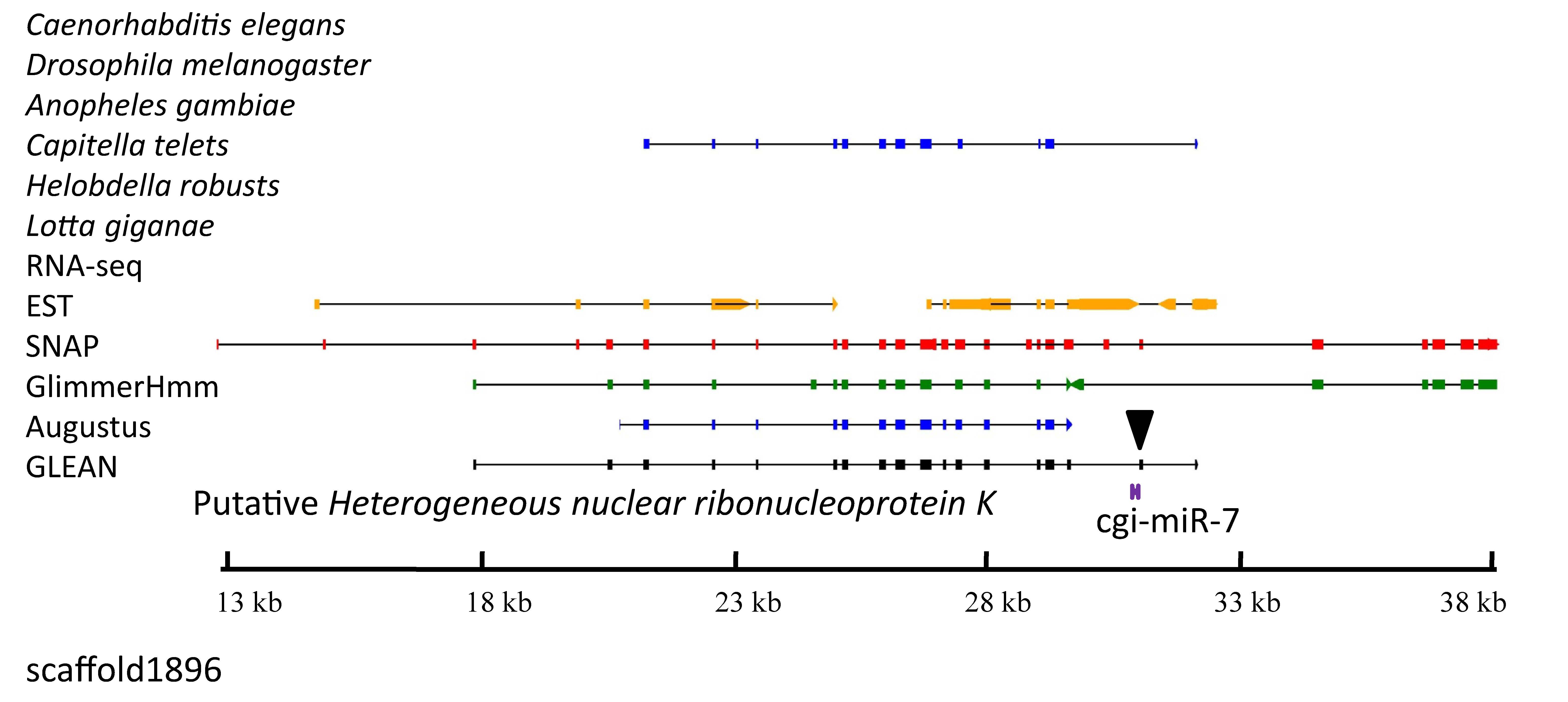


**Figure S3 | Gene model prediction for** **CGI_10019631 (putative *Heterogeneous nuclear ribonucleoprotein K*).**

The final consensus model integrated by the software GLEAN is shown. The penultimate exon (black arrow) was predicted only by the program SNAP (red blocks), whereas it was not supported by a homolog from the annelid *Capitella telets* (blue blocks at the top), ESTs (yellow blocks), or other *in silico* predictions (Glimmer Hmm prediction and Augustus prediction). Based on the most credible EST alignment result, we eliminated the penultimate exon from the gene model, which overlapped with miRNA *cgi-miR-7*. Thus, the miRNAs are intronic.


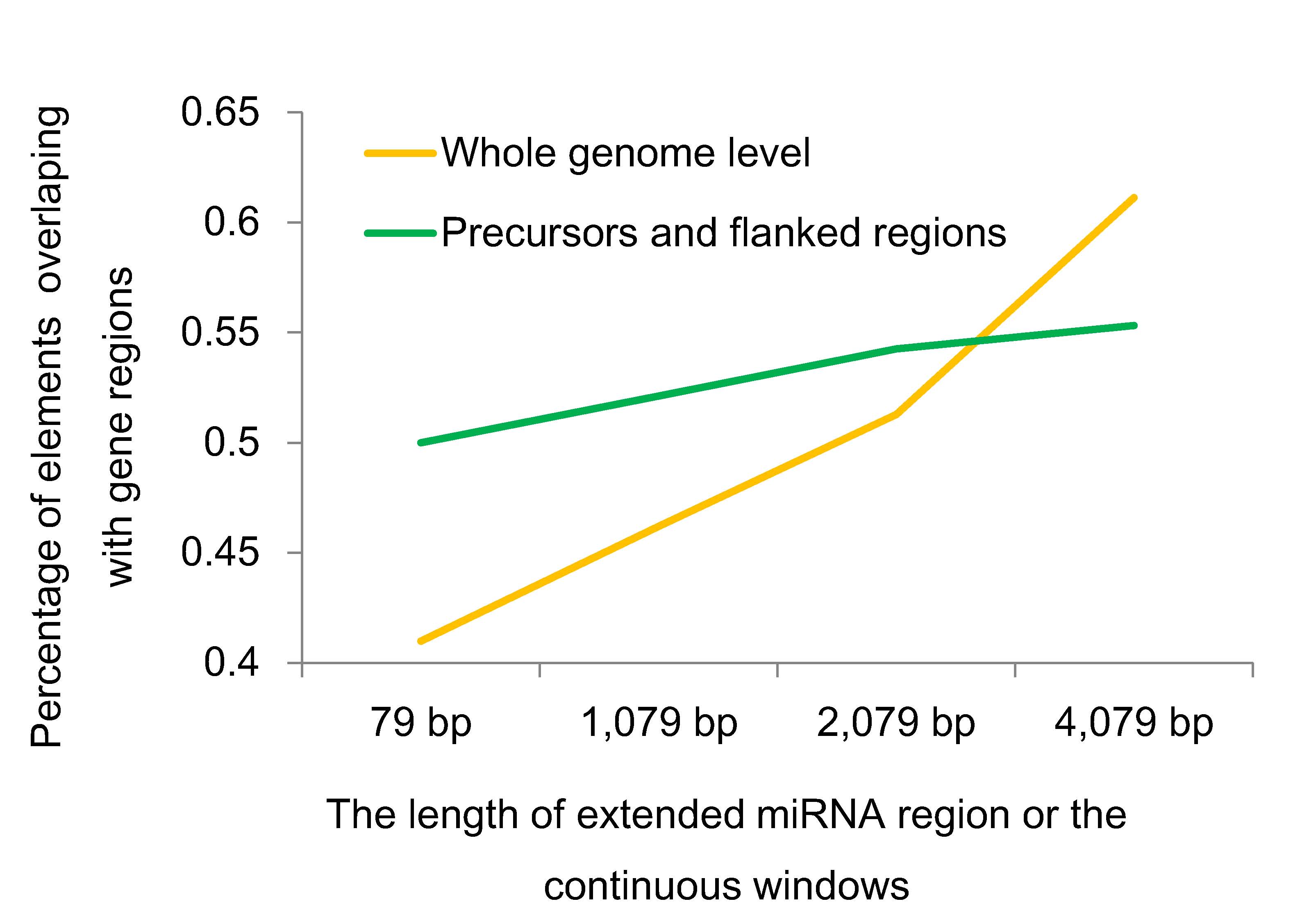


Figure S4 | The overlapping frequency of short sequence elements with gene regions.

The short sequence elements include: hairpin precursor miRNAs (total number = 100, average length = 79 bp) and the continuous windows produced by splicing the genome scaffolds into 79 bp lengths (total number = 7,064,881); the regions containing hairpin precursor miRNAs and their 500 bp upstream and downstream sequences (total number = 100, average length = 1,079 bp) and the continuous windows produced by splicing the genome scaffolds into 1,079 bp length (total number = 513,505); the regions containing hairpin precursor miRNAs and their 1,000 bp upstream and downstream sequences (total number = 100, average length = 2,079 bp) and the continuous windows produced by splicing the genome scaffolds into 2,079 bp lengths (total number = 265,011); the regions containing hairpin precursor miRNAs and their 2,000 bp upstream and downstream sequences (total number= 100, average length = 4,079 bp) and the continuous windows produced by splicing the genome scaffolds into 4,079 bp lengths (total number = 133,678).


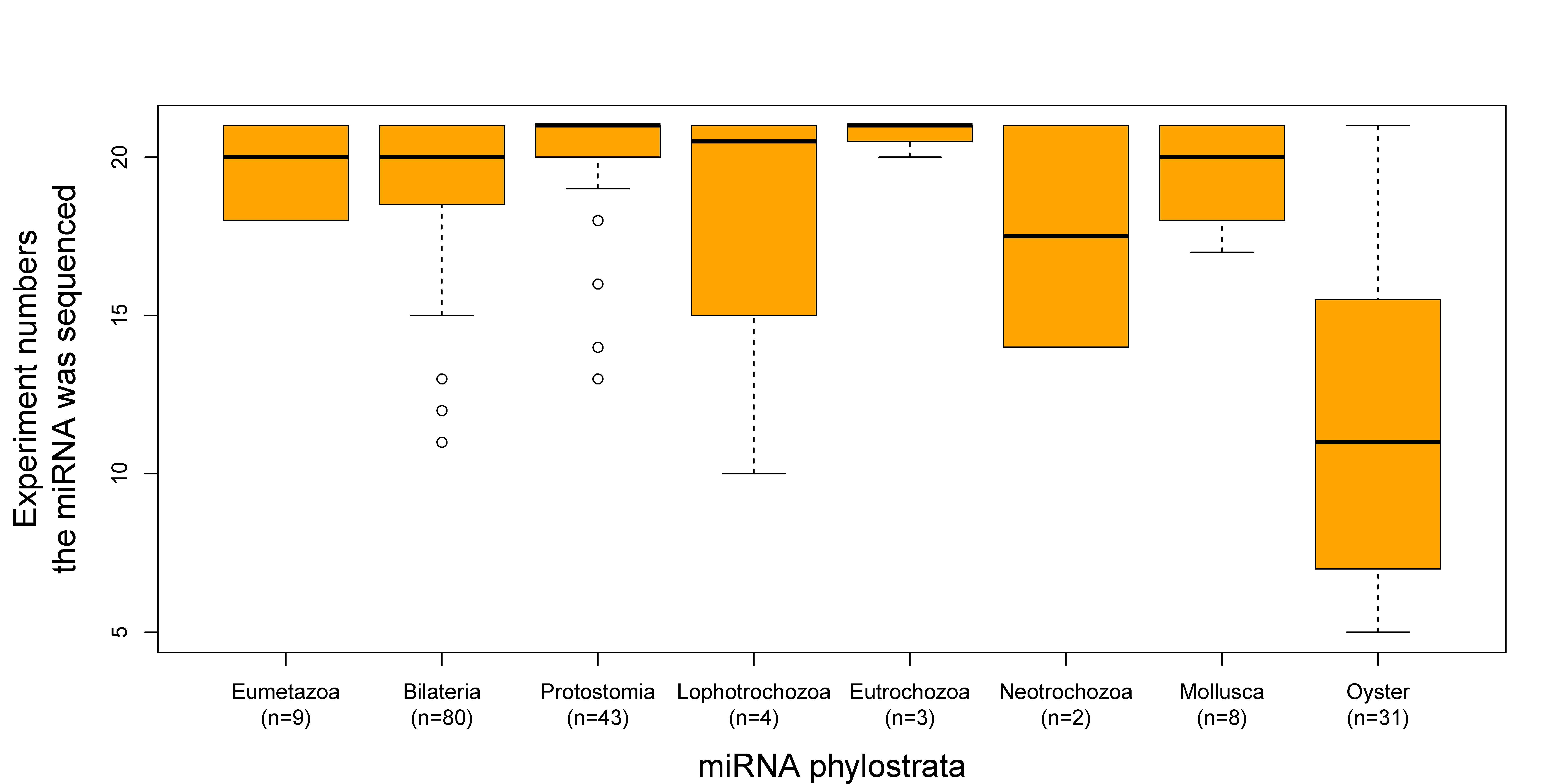


Figure S5 | The presence of miRNAs from different phylostrata in the 21 samples.

The numbers of experiments where the mature miRNA was sequenced were analyzed. The larger *y*-axis value indicates that more experiments sequenced the miRNA. The value of n under each phylostratum on the *x*-axis shows the miRNA number in the corresponding phylostratum.


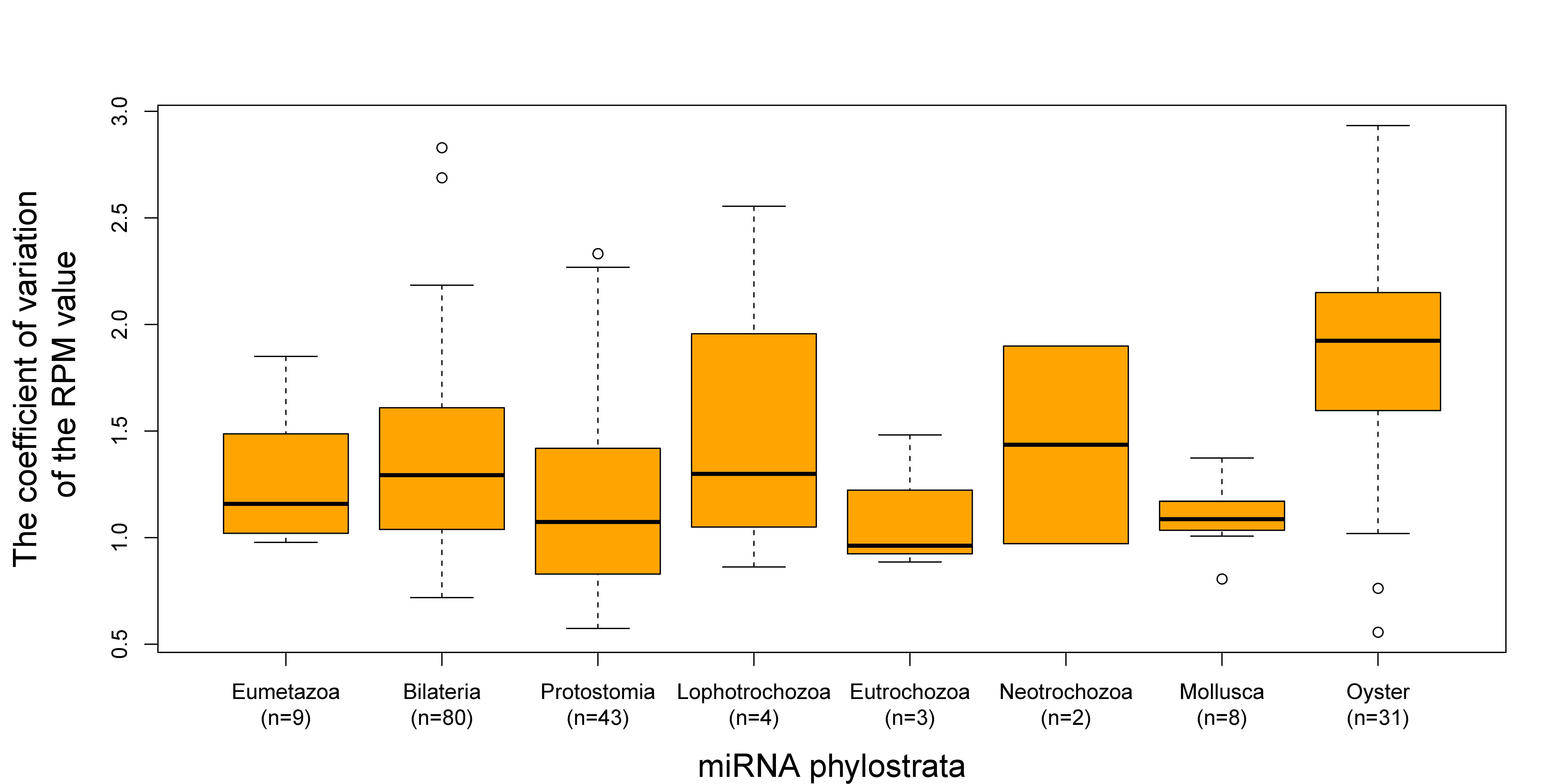


Figure S6 | The coefficients of variation for the RPM values of miRNAs from different phylostrata in the 21 samples.

The coefficients of variation were calculated using the RPM values in 21 samples for each miRNA. The larger *y*-axis value indicates that the expression level (RPM value) varied more.

Table S1 | Data for the developmental samples

| Developmental stages | Sampling time | Age | Mixed libraries^1^ | Developmental stages libraries | RIN value^2^ | Sea water temperature (°C) | Development stage description |
| --- | --- | --- | --- | --- | --- | --- | --- |
| Egg | 2010/6/22 13:25 | 0 h 00 min |  | s01.E |  | 26 | Egg |
| Embryo | 2010/6/22 14:00 | 0 h 35 min | m01.early |  |  | 26 | Second polar body ejected |
|  | 2010/6/22 14:57 | 1 h 32 min | m01.early |  |  | 26 | Four cells |
|  | 2010/6/22 16:55 | 3 h 30 min | m01.early |  |  | 26 | Morula stage |
|  | 2010/6/22 18:00 | 4 h 35 min |  | s02.B |  | 26 | Blastula stage |
|  | 2010/6/22 18:55 | 5 h 30 min | m01.early |  |  | 26 | Rotary movement |
|  | 2010/6/22 21:00 | 7 h 35 min | m01.early |  |  | 26 | Early gastrula stage |
| Trochophore | 2010/6/22 22:55 | 9 h 30 min | m01.early |  | RIN=3.0 | 26 | Trochophore |
|  | 2010/6/22 23:57 | 10 h 32 min |  | s03.T1 | RIN=1.3 | 26 | Trochophore |
|  | 2010/6/23 1:00 | 11 h 35 min | m01.early | s04.T2 |  | 26 | Trochophore |
|  | 2010/6/23 3:00 | 13 h 35 min | m01.early |  |  | 26 | Trochophore |
| D-shape larvae | 2010/6/23 4:55 | 15 h 30 min | m01.early |  |  | 26 | Early D-shape larvae |
|  | 2010/6/23 7:00 | 17 h 35 min | m01.early | s05.D |  | 26 | D-shape larvae |
|  | 2010/6/23 9:00 | 19 h 35 min | m01.early |  |  | 26 | D-shape larvae |
|  | 2010/6/23 11:00 | 21 h 35 min | m02.late |  | RIN=1.5 | 26 | D-shape larvae |
|  | 2010/6/24 7:30 | 1.75 d | m02.late |  |  | 26 | D-shape larvae |
|  | 2010/6/26 8:00 | 3.77 d | m02.late |  |  | 26 | D-shape larvae |
| Umbo larvae | 2010/6/28 7:00 | 5.73 d | m02.late |  | RIN=1.8 | 25 | Early umbo larvae |
|  | 2010/6/30 7:30 | 7.75 d | m02.late |  |  | 25 | Umbo larvae |
|  | 2010/7/1 7:30 | 8.75 d |  | s06.U |  | 25 | Umbo larvae |
|  | 2010/7/2 7:30 | 9.75 d | m02.late |  |  | 25 | Umbo larvae |
|  | 2010/7/4 7:30 | 11.75 d | m02.late |  |  | 25 | Umbo larvae |
|  | 2010/7/6 7:20 | 13.75 d | m02.late |  |  | 25 | Umbo larvae |
|  | 2010/7/8 7:00 | 15.73 d | m02.late |  |  | 25 | Later umbo larvae |
| Pediveliger | 2010/7/9 7:30 | 16.75 d | m02.late | s07.P1 | RIN=2.7 | 25 | Pediveliger |
|  | 2010/7/10 14:10 | 18.03 d |  | s08.P2 |  | 25 | Pediveliger |
| Spat | 2010/7/14 17:00 | 22.15 d | m02.late | s09.S |  | 25 | Spat |
| Juvenile | 2011/1/23 | 215 d |  | s10.J |  | Ambient, sea | Juvenile |
| Adult | 2010/5 | 2-3 years |  | m03.Adult |  | Ambient, sea | Organs mixture of an adult |

Note: 1, mixed libraries m01.early and m02.late were constructed by mixing RNA from the developmental stages marked as m01.early or m02.late. 2, The RNA integrity number (RIN) was used to assess the RNA quality. Some libraries with RIN values of <3.0 were noted.

Table S2 | Reads numbers for the 21 libraries

| **Library** | **RawData** | | | **Mapped_data** | | | | | |
| --- | --- | --- | --- | --- | --- | --- | --- | --- | --- |
|  | Base (G) | Reads (M) | Uniq_Reads (M) | Base (G) | % | Reads (M) | % | Mapped_Uniq_Reads (M) | % |
| m01.Early | 0.45 | 16.58 | 3.47 | 0.28 | 62.01% | 10.46 | 63.07% | 1.66 | 48.02% |
| m02.Late | 0.42 | 16.91 | 2.03 | 0.31 | 75.50% | 13.02 | 76.96% | 0.79 | 39.11% |
| m03.Adult | 0.33 | 13.18 | 1.73 | 0.25 | 75.07% | 10.18 | 77.21% | 0.84 | 48.59% |
| s01.E | 0.36 | 13.17 | 2.85 | 0.21 | 59.75% | 7.91 | 60.04% | 1.35 | 47.65% |
| s02.B | 0.35 | 13.57 | 2.80 | 0.22 | 61.44% | 8.40 | 61.96% | 1.36 | 48.74% |
| s03.T1 | 0.35 | 14.26 | 2.57 | 0.25 | 71.75% | 10.36 | 72.67% | 1.28 | 49.79% |
| s04.T2 | 0.48 | 18.63 | 3.03 | 0.34 | 69.81% | 13.27 | 71.22% | 1.40 | 46.30% |
| s05.D | 0.29 | 12.69 | 1.00 | 0.26 | 87.67% | 11.21 | 88.29% | 0.54 | 54.70% |
| s06.U | 0.31 | 12.92 | 1.06 | 0.26 | 82.90% | 10.91 | 84.46% | 0.50 | 47.23% |
| s07.P1 | 0.25 | 10.97 | 0.82 | 0.21 | 84.70% | 9.40 | 85.65% | 0.34 | 41.88% |
| s08.P2 | 0.51 | 21.82 | 1.21 | 0.44 | 86.05% | 18.99 | 87.04% | 0.45 | 37.33% |
| s09.S | 0.25 | 10.75 | 1.64 | 0.08 | 34.41% | 3.82 | 35.55% | 0.20 | 12.33% |
| s10.J | 0.27 | 12.06 | 1.52 | 0.23 | 83.46% | 10.22 | 84.75% | 0.79 | 52.02% |
| t01.Mao | 0.31 | 13.84 | 0.44 | 0.28 | 90.65% | 12.60 | 90.99% | 0.21 | 47.34% |
| t02.Mai | 0.31 | 13.70 | 0.34 | 0.28 | 90.61% | 12.47 | 91.00% | 0.16 | 46.19% |
| t03.Dgl | 0.30 | 12.92 | 1.51 | 0.25 | 82.77% | 10.87 | 84.09% | 0.79 | 52.46% |
| t04.Gil | 0.27 | 11.92 | 0.48 | 0.24 | 88.16% | 10.59 | 88.84% | 0.19 | 39.68% |
| t05.Amu | 0.34 | 15.08 | 0.53 | 0.32 | 91.65% | 13.90 | 92.18% | 0.27 | 51.33% |
| t06.Hem | 0.39 | 15.08 | 3.01 | 0.24 | 62.72% | 9.74 | 64.58% | 1.20 | 39.97% |
| t07.Lpa | 0.27 | 12.10 | 0.26 | 0.25 | 92.16% | 11.19 | 92.48% | 0.13 | 50.30% |
| t08.Fgo | 0.36 | 13.58 | 2.93 | 0.23 | 64.92% | 9.02 | 66.44% | 1.45 | 49.45% |

Note: The abbreviations of the developmental stages are the same as those in Table S1. Organs are abbreviated as: t01.Mao, outer edge of mantle along the margin of the shell; t02.Mai, inner pallial part covering the inner surface of the shell; t03.Dgl, digestive gland; t04.Gil, gills; t05.Amu, adductor muscle; t06.Hem, hemocyte; t07.Lpa, labial palp; t08.Fgo, female gonad.

Table S3 | Reads annotation results for different RNA categories

See Excel file.

**Table S4 | Details of the 81 conserved precursors and their mature miRNAs**

This table shows the following information for the 81 conserved precursors and the mature miRNAs: precursor ID; mature miRNA ID; homologs in the miRBase v20; miRNA family information; the predicted free energy of folding; sequence and secondary structure of the hairpin precursor; sequence of the predicted mature miRNA; physical location of the hairpin precursor based on the oyster genome version 1 (scaffold ID, start site, end site, and strand); miRNA cluster information (a cluster was defined as having a maximum gap of 10 Kbp between contiguous precursors); miRNA age and phylostratum (evolutionary acquisition, see the main text); the gene ID that overlapped with the miRNA and the strand information; read number and the RPM value for each miRNA in each sample.

See Excel file.

**Table S5 | Details of the 19 predicted novel oyster precursors and their mature miRNA products**

This table shows the following information for the 19 predicted novel oyster precursors and their mature miRNAs: precursor ID; mature miRNA ID; the predicted free energy of folding; sequence and secondary structure of the hairpin precursor; sequence of the predicted mature miRNA; physical location of the hairpin precursor based on the oyster genome version 1 (scaffold ID, start site, end site, and strand); miRNA cluster information (a cluster was defined as having a maximum gap of 10 Kbp between contiguous precursors); miRNA age and phylostratum (evolutionary acquisition, see the main text); the gene ID that overlapped with the miRNA and the strand information; read number and the RPM value for each miRNA in each sample.

See Excel file.

**Table S6 | The family distribution of conserved oyster miRNAs**

See Excel file.

**Table S7 |** **Details of the 27 potential oyster precursors and their mature miRNA products**

This table shows the following information for the 27 potential oyster precursors and their mature miRNAs: precursor ID; mature miRNA ID; the reason excluding the corresponding precursor from oyster miRNA gene set; the predicted free energy of folding; sequence and secondary structure of the hairpin precursor; sequence of the predicted mature miRNA; physical location of the hairpin precursor based on the oyster genome version 1 (scaffold ID, start site, end site, and strand); miRNA cluster information (a cluster was defined as having a maximum gap of 10 Kbp between contiguous precursors); the gene ID that overlapped with the miRNA and the strand information; read number and the RPM value for each miRNA in each sample.

See Excel file.

**Table S8 | The BLAST hits of *mir-184* in the oyster genome.**

See Excel file.

**Table S9 | Details of the oyster miRNA clusters**

| **Cluster** | **Precursor** | **family** | **Scaffold** | **Start** | **End** | **Strand** |
| --- | --- | --- | --- | --- | --- | --- |
| cluster1 | *cgi-miR-1* | mir-1 | scaffold1174 | 365473 | 365556 | + |
| cluster1 | *cgi-miR-133* | mir-133 | scaffold1174 | 373452 | 373524 | + |
| cluster2 | *cgi-miR-2001* | mir-2001 | scaffold1366 | 96719 | 96797 | + |
| cluster2 | *cgi-miR-252a* | mir-252 | scaffold1366 | 96950 | 97031 | + |
| cluster2 | *cgi-miR-252b* | mir-252 | scaffold1366 | 106392 | 106469 | + |
| cluster3 | *cgi-miR-29-1* | mir-29 | scaffold145 | 184789 | 184871 | + |
| cluster3 | *cgi-miR-29-2* | mir-29 | scaffold145 | 188902 | 188977 | + |
| cluster4 | *cgi-miR-67a* | mir-67 | scaffold146 | 102285 | 102361 | + |
| cluster4 | *cgi-miR-307* | mir-67 | scaffold146 | 102628 | 102708 | + |
| cluster5 | *cgi-miR-2a* | mir-2 | scaffold1503 | 13863 | 13941 | - |
| cluster5 | *cgi-miR-2b* | mir-2 | scaffold1503 | 15152 | 15231 | - |
| cluster6 | *cgi-miR-12* | mir-12 | scaffold1785 | 68044 | 68120 | - |
| cluster6 | *cgi-miR-216a* | mir-216 | scaffold1785 | 68800 | 68877 | - |
| cluster6 | *cgi-miR-216b* | mir-216 | scaffold1785 | 69351 | 69427 | - |
| cluster7 | *cgi-miR-1990* | mir-1990 | scaffold264 | 40650 | 40726 | + |
| cluster7 | *cgi-miR-1986* | mir-1986 | scaffold264 | 41056 | 41133 | + |
| cluster8 | *cgi-miR-1984* | mir-1984 | scaffold339 | 59448 | 59534 | + |
| cluster8 | *cgi-miR-1985* | mir-1985 | scaffold339 | 60087 | 60165 | + |
| cluster9 | *cgi-miR-9b* | mir-9 | scaffold42448 | 83572 | 83653 | - |
| cluster9 | *cgi-miR-9c* | mir-9 | scaffold42448 | 84454 | 84534 | - |
| cluster9 | *cgi-miR-9d* | mir-9 | scaffold42448 | 85400 | 85480 | - |
| cluster10 | *cgi-miR-125* | mir-10 | scaffold43364 | 40011 | 40082 | - |
| cluster10 | *cgi-let-7* | let-7 | scaffold43364 | 47249 | 47339 | - |
| cluster10 | *cgi-miR-100* | mir-10 | scaffold43364 | 47983 | 48053 | - |
| cluster11 | *cgi-miR-36* | mir-36 | scaffold43988 | 9119 | 9195 | - |
| cluster11 | *cgi-miR-279* | mir-279 | scaffold43988 | 13116 | 13191 | - |
| cluster12 | *cgi-miR-92d* | mir-25 | scaffold459 | 310875 | 310951 | - |
| cluster12 | *cgi-miR-92c* | mir-25 | scaffold459 | 311193 | 311272 | - |
| cluster12 | *cgi-miR-92b* | mir-25 | scaffold459 | 320184 | 320263 | - |
| cluster13 | *cgi-miR-1175* | mir-1175 | scaffold514 | 99882 | 99958 | - |
| cluster13 | *cgi-miR-750* | mir-750 | scaffold514 | 103015 | 103104 | - |
| cluster14 | *cgi-miR-71* | mir-71 | scaffold602 | 444830 | 444906 | + |
| cluster14 | *cgi-miR-2c* | mir-2 | scaffold602 | 445130 | 445206 | + |
| cluster14 | *cgi-miR-2d-1* | mir-2 | scaffold602 | 445404 | 445484 | + |
| cluster14 | *cgi-miR-2e* | mir-2 | scaffold602 | 445713 | 445790 | + |
| cluster14 | *cgi-miR-2f* | mir-2 | scaffold602 | 445832 | 445909 | + |
| cluster14 | *cgi-miR-2d-2* | mir-2 | scaffold602 | 446009 | 446083 | + |
| cluster14 | *cgi-miR-2g* | mir-2 | scaffold602 | 446524 | 446602 | + |
| cluster15 | *cgi-miR-317a* | mir-317 | scaffold622 | 468066 | 468144 | + |
| cluster15 | *cgi-miR-317b* | mir-317 | scaffold622 | 468573 | 468650 | + |
| cluster15 | *cgi-miR-277* | mir-277 | scaffold622 | 474074 | 474149 | + |
| cluster15 | *cgi-miR-34* | mir-34 | scaffold622 | 474782 | 474865 | + |
| cluster16 | *cgi-miR-183* | mir-263 | scaffold626 | 18341 | 18418 | + |
| cluster16 | *cgi-miR-182* | mir-183 | scaffold626 | 26557 | 26641 | + |
| cluster17 | *cgi-miR-745b* | mir-745 | scaffold726 | 81932 | 82015 | - |
| cluster17 | *cgi-miR-745a* | mir-745 | scaffold726 | 82794 | 82871 | - |
| cluster18 | *cgi-miR-1994b* | mir-1994 | scaffold950 | 63798 | 63876 | - |
| cluster18 | *cgi-miR-1994a* | mir-1994 | scaffold950 | 63983 | 64059 | - |
| cluster19 | *m0146* |  | scaffold150 | 1133587 | 1133670 | + |
| cluster19 | *m0148* |  | scaffold150 | 1133978 | 1134055 | + |
| cluster20 | *m0314* |  | scaffold370 | 642749 | 642827 | + |
| cluster20 | *m0315* |  | scaffold370 | 643274 | 643354 | + |
| cluster20 | *m0317* |  | scaffold370 | 643640 | 643718 | + |

Table S10 | The Pearson correlation coefficient between miRNA expression and host genes

The expression level of the host genes were from the literature and were measured with RPKM (Zhang et. al. 2012). The RNAs used for miRNA sequencing in this study and those used in the RNA sequencing in the previous report were from the same samples. The correspondence of the sample names in this study and the previous report are as follows: s01.E_RPM - E_RPKM, s02.B_RPM - B_RPKM, s04.T2_RPM - T2_RPKM, s05.D_RPM - D1_RPKM, s06.U_RPM - U2_RPKM, s08.P2_RPM - P1_RPKM, s09.S_RPM - S_RPKM, s10.J_RPM - J_RPKM, t01.Mao_RPM - Man1_RPKM, t02.Mai_RPM - Man2_RPKM, t03.Dgl_RPM - Dgl_RPKM, t04.Gil_RPM - Gil_RPKM, t05.Amu_RPM - Amu_RPKM, t06.Hem_RPM - Hem_RPKM, t07.Lpa_RPM - Lpa_RPKM, t08.Fgo_RPM - Fgo_RPKM.

See Excel file.

Table S11 | The 180 miRNAs chosen for analyzing the expression patterns.

See Excel file.

Table S12 | Specifically highly expressed miRNAs and specifically lowly expressed miRNAs in each organs

| **Organs** | **Specifically highly expressed miRNAs** | **Specifically lowly expressed miRNAs** |
| --- | --- | --- |
| Mantle | *cgi-miR-1990-3p; cgi-miR-1986-3p; cgi-miR-219-5p;* ***cgi-miR-9c-5p; cgi-miR-9d-5p*** |  |
| t03.Dgl | ***m0043_5p****;* ***m0043_3p****;* ***cgi-miR-9a-5p****; cgi-miR-1175-5p; cgi-miR-1175-3p; m0442_5p; m0442_3p; cgi-miR-750* |  |
| T04.Gil | *cgi-miR-252b-3p* |  |
| t05.Amu | *cgi-miR-1991-5p; cgi-miR-1991-3p;* | *cgi-miR-92a-3p; m0078_3p; cgi-miR-2722-5p; cgi-miR-252a-5p; cgi-miR-252a-3p; cgi-miR-29-1-3p; cgi-miR-29-2-3p; cgi-miR-10a-5p; cgi-miR-67a-5p; cgi-miR-67a-3p; cgi-miR-216a-5p; cgi-miR-216a-3p; cgi-miR-216b-5p; cgi-miR-278-3p; cgi-miR-7-5p; cgi-miR-8-5p; cgi-miR-8-3p; m0360_3p; cgi-miR-1992-3p; cgi-miR-279-3p; cgi-miR-92d-5p; cgi-miR-92d-3p; cgi-miR-92c-3p; cgi-miR-92b-3p; cgi-miR-981-3p; cgi-miR-96a-5p; cgi-miR-182-5p; cgi-miR-745b-3p; cgi-miR-96b-5p; cgi-miR-10b-5p; cgi-miR-10b-3p; cgi-miR-210-3p* |
| t06.Hem | *m0078_3p; cgi-miR-67a-5p; cgi-miR-307-3p; cgi-miR-67b-5p; cgi-miR-182-5p; cgi-miR-182-3p; m0489_3p; cgi-miR-281-5p; cgi-miR-281-3p; cgi-bantam-3p* | *cgi-miR-745b-3p* |
| t07.Lpa | ***cgi-miR-9c-5p; cgi-miR-9d-5p*** |  |
| t08.Fgo | ***m0043_5p****;* ***m0043_3p****;* *m0189_5p;* ***cgi-miR-9a-5p*** |  |

Note: the organs t01.Mao and t02.Mai were combined as “Mantle” by averaging the RPM values of the two samples for each miRNA. The families IDs were listed in the bracket. The bold IDs showed the miRNAs which specifically highly expressed in two organs.
