# Supplementary material for: Identification of Conserved and Novel MicroRNAs in the Pacific Oyster Crassostrea gigas by Deep Sequencing
Source: PLoS One. 2014 Aug 19;9(8):e104371. doi: 10.1371/journal.pone.0104371 (PMC4138081; doi:10.1371/journal.pone.0104371)
Supplement: File S2 — The compressed/ZIP file archive for the predicted precursors' secondary structures and reads alignment. (ZIP) [file pone.0104371.s010.zip › second structure and reads alignment for oyster miRNAs/conserved in table S4/cgi-bantam.pdf]

miRBase precursor : cgi-bantam
Total read count : 387598
cgi-bantam-5p read count : 113
cgi-bantam-3p read count : 387481
remaining reads : 4

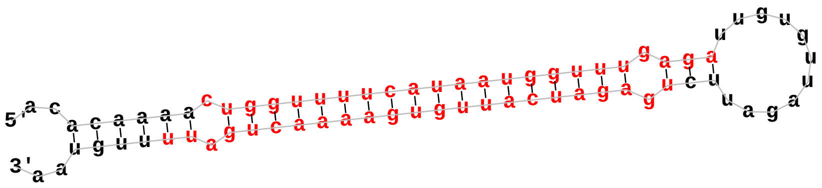

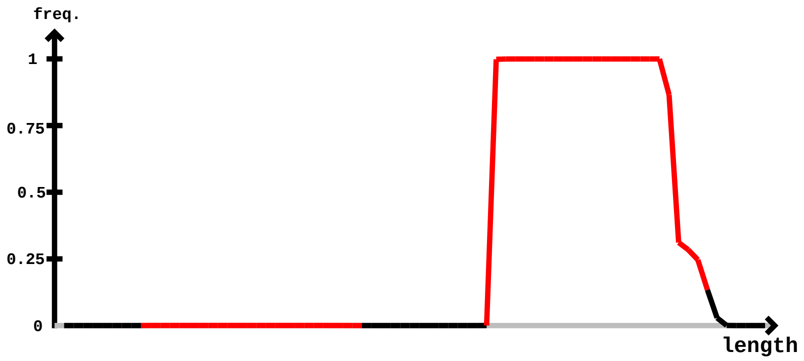


```
        cgi-bantam-5p
                                              cgi-bantam-3p
5'- acacaaaacugguuuucauaaugguuugagauuguguuagauucugagaucauugugaaaacugauuuuguaa  -3'    exp
    ..((((((.((((((((((((((((((.(((...........))).)))))))))))))))))).))))))..   reads    mm      sample
    .......acugguuuucauaaugguuuga............................................   2        0       seq
    ........cugguuuucauaaugguu...............................................   6        0       seq
    ........cugguuuucauaaugguuu..............................................   7        0       seq
    ........cugguuuucauaaugguuug.............................................   24       0       seq
    ........cugguuuucauaaugguuuga............................................   24       0       seq
    ........cugguuuucauaaugguuugag...........................................   21       0       seq
    ........cugguuuucauaaugguuugaga..........................................   13       0       seq
    .........ugguuuucauaaugguuu..............................................   4        0       seq
    .........ugguuuucauaaugguuug.............................................   3        0       seq
    .........ugguuuucauaaugguuuga............................................   5        0       seq
    .........ugguuuucauaaugguuugag...........................................   2        0       seq
    .........ugguuuucauaaugguuugaga..........................................   2        0       seq
    ..............................uuguguuagauucugagaucauugu..................   1        0       seq
    ..............................uuguguuagauucugagaucauugugaa...............   2        0       seq
    ..............................uuguguuagauucugagaucauugugaaa..............   1        0       seq
    ..........................................cugagaucauugugaaaa.............   2        0       seq
    ..........................................cugagaucauugugaaaac............   13       0       seq
    ..........................................cugagaucauugugaaaacu...........   44       0       seq
    ..........................................cugagaucauugugaaaacug..........   1        0       seq
    ..........................................cugagaucauugugaaaacuga.........   2        0       seq
    ..........................................cugagaucauugugaaaacugau........   2        0       seq
    ..........................................cugagaucauugugaaaacugauu.......   3        0       seq
    ..........................................cugagaucauugugaaaacugauuu......   1        0       seq
    ...........................................ugagaucauugugaaaac............   51924    0       seq
    ...........................................ugagaucauugugaaaacu...........   214670   0       seq
    ...........................................ugagaucauugugaaaacug..........   10577    0       seq
    ...........................................ugagaucauugugaaaacuga.........   14585    0       seq
    ...........................................ugagaucauugugaaaacugau........   43613    0       seq
    ...........................................ugagaucauugugaaaacugauu.......   40411    0       seq
    ...........................................ugagaucauugugaaaacugauuu......   10993    0       seq
    ...........................................ugagaucauugugaaaacugauuuu.....   141      0       seq
    ............................................gagaucauugugaaaacu...........   121      0       seq
    ............................................gagaucauugugaaaacug..........   11       0       seq
    ............................................gagaucauugugaaaacuga.........   23       0       seq
```

```
        cgi-bantam-5p
                                    cgi-bantam-3p
acacaaaacugguuuucauaaugguuugagauuguguuagauucugagaucauugugaaaacugauuuuguaa

............................................gagaucauugugaaaacugau.......    87     0     seq
............................................gagaucauugugaaaacugauu......    118    0     seq
............................................gagaucauugugaaaacugauuu.....    60     0     seq
.............................................agaucauugugaaaacug.........    11     0     seq
.............................................agaucauugugaaaacuga........    6      0     seq
.............................................agaucauugugaaaacugau.......    19     0     seq
.............................................agaucauugugaaaacugauu......    28     0     seq
.............................................agaucauugugaaaacugauuu.....    9      0     seq
...............................................aucauugugaaaacugau.......    1      0     seq
...............................................aucauugugaaaacugauu......    5      0     seq
```
