# Supplementary material for: Identification of Conserved and Novel MicroRNAs in the Pacific Oyster Crassostrea gigas by Deep Sequencing
Source: PLoS One. 2014 Aug 19;9(8):e104371. doi: 10.1371/journal.pone.0104371 (PMC4138081; doi:10.1371/journal.pone.0104371)
Supplement: File S2 — The compressed/ZIP file archive for the predicted precursors' secondary structures and reads alignment. (ZIP) [file pone.0104371.s010.zip › second structure and reads alignment for oyster miRNAs/conserved in table S4/cgi-let-7.pdf]

miRBase precursor : cgi-let-7
Total read count : 25286236
cgi-let-7-5p read count : 25285600
cgi-let-7-3p read count : 228
remaining reads : 408

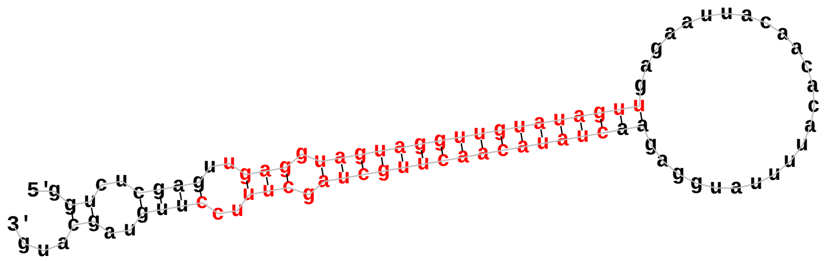

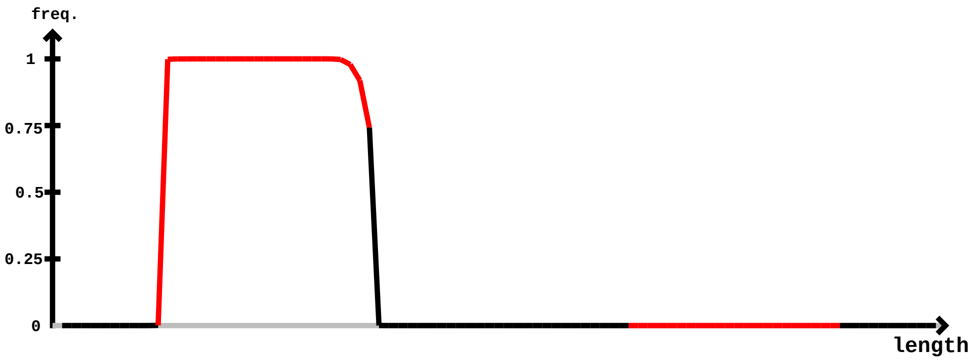


```
                 cgi-let-7-5p
                                                                     cgi-let-7-3p
5'- ggucucgaguugagguaguagguuguauaguugagaauuacaacacauuuuauggagaacuauacaacuugcuagcuuuccuuguagcaug   -3'    exp
    .((..((((..(((.(((((((((((((((((..........................))))))))))))))))).)))..))))..))...    reads    mm    sample
    .......aguugagguaguagguug..................................................................    52       0     seq
    .......aguugagguaguagguugu.................................................................    23       0     seq
    .......aguugagguaguagguugua................................................................    59       0     seq
    .......aguugagguaguagguuguau...............................................................    219      0     seq
    .......aguugagguaguagguuguaua..............................................................    2        0     seq
    ........guugagguaguagguugu.................................................................    4        0     seq
    ........guugagguaguagguugua................................................................    1        0     seq
    ........guugagguaguagguuguau...............................................................    1        0     seq
    ........guugagguaguagguuguaua..............................................................    164      0     seq
    ........guugagguaguagguuguauaguu...........................................................    1        0     seq
    .........uugagguaguagguugua................................................................    31       0     seq
    .........uugagguaguagguuguau...............................................................    135      0     seq
    .........uugagguaguagguuguaua..............................................................    2390     0     seq
    .........uugagguaguagguuguauag.............................................................    6090     0     seq
    .........uugagguaguagguuguauagu............................................................    10076    0     seq
    .........uugagguaguagguuguauaguu...........................................................    209      0     seq
    ..........ugagguaguagguuguau...............................................................    55069    0     seq
    ..........ugagguaguagguuguaua..............................................................    475283   0     seq
    ..........ugagguaguagguuguauag.............................................................    1495978  0     seq
    ..........ugagguaguagguuguauagu............................................................    4459364  0     seq
    ..........ugagguaguagguuguauaguu...........................................................    18742565 0     seq
    ..........ugagguaguagguuguauaguug..........................................................    3668     0     seq
    ..........ugagguaguagguuguauaguuga.........................................................    121      0     seq
    ..........ugagguaguagguuguauaguugag........................................................    30       0     seq
    ..........ugagguaguagguuguauaguugaga.......................................................    12       0     seq
    ..........ugagguaguagguuguauaguugagaa......................................................    19       0     seq
    ..........ugagguaguagguuguauaguugagaau.....................................................    5        0     seq
    ..........ugagguaguagguuguauaguugagaauu....................................................    3        0     seq
    ...........gagguaguagguuguaua..............................................................    994      0     seq
    ...........gagguaguagguuguauag.............................................................    1728     0     seq
    ...........gagguaguagguuguauagu............................................................    4387     0     seq
    ...........gagguaguagguuguauaguu...........................................................    22998    0     seq
    ...........gagguaguagguuguauaguug..........................................................    13       0     seq
    ...........gagguaguagguuguauaguugag........................................................    1        0     seq
```

cgi-let-7-5p

cgi-let-7-3p

```
ggucucgaguugagguaguagguuguauaguugagaauuacaacacauuuuauggagaacuauacaacuugcuagcuuuccuuguagcaug

............agguaguagguuguauag.............................................................    179    0    seq
............agguaguagguuguauagu............................................................    505    0    seq
............agguaguagguuguauaguu...........................................................    2469   0    seq
............agguaguagguuguauaguug..........................................................    52     0    seq
.............gguaguagguuguauagu............................................................    100    0    seq
.............gguaguagguuguauaguu...........................................................    444    0    seq
.............gguaguagguuguauaguug..........................................................    92     0    seq
.............gguaguagguuguauaguuga.........................................................    248    0    seq
..............guaguagguuguauaguu...........................................................    111    0    seq
..............guaguagguuguauaguug..........................................................    13     0    seq
..............guaguagguuguauaguuga.........................................................    15     0    seq
..............guaguagguuguauaguugag........................................................    15     0    seq
...............uaguagguuguauaguug..........................................................    6      0    seq
...............uaguagguuguauaguuga.........................................................    2      0    seq
...............uaguagguuguauaguugag........................................................    9      0    seq
...............uaguagguuguauaguugagaau.....................................................    1      0    seq
................aguagguuguauaguuga.........................................................    5      0    seq
................aguagguuguauaguugag........................................................    3      0    seq
..................uagguuguauaguugagaau.....................................................    2      0    seq
..................uagguuguauaguugagaauu....................................................    2      0    seq
...............................ugagaauuacaacacauuuuau......................................    1      0    seq
...............................ugagaauuacaacacauuuuauggaga.................................    1      0    seq
...............................ugagaauuacaacacauuuuauggagaac...............................    1      0    seq
................................gagaauuacaacacauuuuaugg....................................    2      0    seq
................................gagaauuacaacacauuuuauggaga.................................    1      0    seq
................................gagaauuacaacacauuuuauggagaa................................    8      0    seq
.................................agaauuacaacacauuuua.......................................    1      0    seq
................................................uuuauggagaacuauacaacuugcu..................    1      0    seq
..................................................uauggagaacuauacaacuug....................    1      0    seq
..................................................uauggagaacuauacaacuugcu..................    1      0    seq
...................................................auggagaacuauacaacuug....................    2      0    seq
...................................................auggagaacuauacaacuugc...................    1      0    seq
...................................................auggagaacuauacaacuugcu..................    6      0    seq
....................................................uggagaacuauacaacuugcuagcuuu............    3      0    seq
.....................................................ggagaacuauacaacuugcuagcuuu............    1      0    seq
.....................................................ggagaacuauacaacuugcuagcuuuc...........    1      0    seq
......................................................gagaacuauacaacuugcua.................    2      0    seq
......................................................gagaacuauacaacuugcuag................    1      0    seq
......................................................gagaacuauacaacuugcuagc...............    5      0    seq
.........................................................aacuauacaacuugcuagcuuucc..........    1      0    seq
..........................................................acuauacaacuugcuagcuuucc..........    1      0    seq
...........................................................cuauacaacuugcuagcu..............    1      0    seq
...........................................................cuauacaacuugcuagcuu.............    5      0    seq
...........................................................cuauacaacuugcuagcuuu............    10     0    seq
...........................................................cuauacaacuugcuagcuuuc...........    30     0    seq
...........................................................cuauacaacuugcuagcuuucc..........    92     0    seq
...........................................................cuauacaacuugcuagcuuuccu.........    1      0    seq
............................................................uauacaacuugcuagcuu.............    1      0    seq
............................................................uauacaacuugcuagcuuu............    13     0    seq
............................................................uauacaacuugcuagcuuuc...........    21     0    seq
............................................................uauacaacuugcuagcuuucc..........    26     0    seq
............................................................uauacaacuugcuagcuuuccu.........    24     0    seq
.............................................................auacaacuugcuagcuuuc...........    1      0    seq
..............................................................uacaacuugcuagcuuucc..........    1      0    seq
```
