# Supplementary material for: Identification of Conserved and Novel MicroRNAs in the Pacific Oyster Crassostrea gigas by Deep Sequencing
Source: PLoS One. 2014 Aug 19;9(8):e104371. doi: 10.1371/journal.pone.0104371 (PMC4138081; doi:10.1371/journal.pone.0104371)
Supplement: File S2 — The compressed/ZIP file archive for the predicted precursors' secondary structures and reads alignment. (ZIP) [file pone.0104371.s010.zip › second structure and reads alignment for oyster miRNAs/conserved in table S4/cgi-miR-1.pdf]

miRBase precursor : cgi-miR-1
Total read count : 54893716
cgi-miR-1-5p read count : 1622
cgi-miR-1-3p read count : 54891888
remaining reads : 206

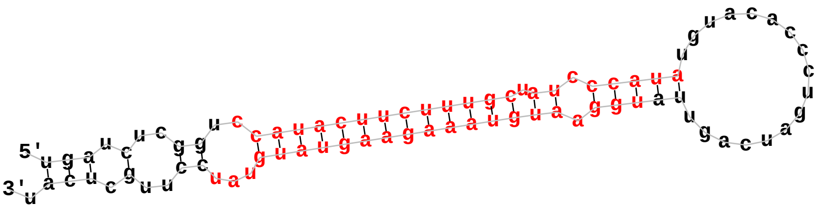

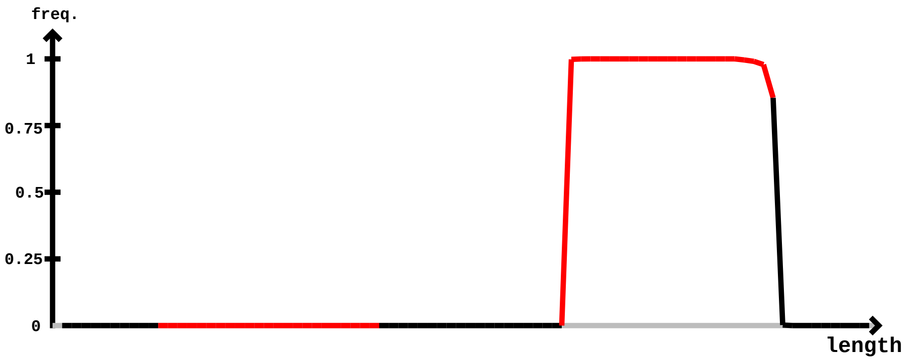


```
                                   cgi-miR-1-5p
                                                                               cgi-miR-1-3p
5'- ugaucucgguccauacuucuuugcuaucccauauguacacccugaucaguuauggaauguaaagaaguauguauccuugcucau   -3'   exp
    (((.(..((..(((((((((((((.((.(((((.................))))).)))))))))))))))...))..).))).   reads   mm   sample
    .........uccauacuucuuugcuaucc.......................................................   2       0    seq
    .........uccauacuucuuugcuauccc......................................................   1       0    seq
    .........uccauacuucuuugcuauccca.....................................................   6       0    seq
    .........uccauacuucuuugcuaucccau....................................................   14      0    seq
    ..........ccauacuucuuugcuauc........................................................   16      0    seq
    ..........ccauacuucuuugcuaucc.......................................................   21      0    seq
    ..........ccauacuucuuugcuauccc......................................................   19      0    seq
    ..........ccauacuucuuugcuauccca.....................................................   131     0    seq
    ..........ccauacuucuuugcuaucccau....................................................   515     0    seq
    ..........ccauacuucuuugcuaucccaua...................................................   865     0    seq
    ..........ccauacuucuuugcuaucccauau..................................................   12      0    seq
    ..........ccauacuucuuugcuaucccauaug.................................................   1       0    seq
    ...........cauacuucuuugcuauccca.....................................................   1       0    seq
    ...........cauacuucuuugcuaucccau....................................................   2       0    seq
    ...........cauacuucuuugcuaucccaua...................................................   5       0    seq
    ............auacuucuuugcuauccca.....................................................   1       0    seq
    ............auacuucuuugcuaucccau....................................................   1       0    seq
    ............auacuucuuugcuaucccaua...................................................   9       0    seq
    ................................auguacacccugaucaguu.................................   1       0    seq
    .................................uguacacccugaucaguua................................   12      0    seq
    .................................uguacacccugaucaguuau...............................   2       0    seq
    .................................uguacacccugaucaguuauggaa...........................   1       0    seq
    .................................uguacacccugaucaguuauggaau..........................   2       0    seq
    .................................uguacacccugaucaguuauggaaug.........................   1       0    seq
    .................................uguacacccugaucaguuauggaaugua.......................   10      0    seq
    .................................uguacacccugaucaguuauggaauguaa......................   24      0    seq
    .................................uguacacccugaucaguuauggaauguaaa.....................   3       0    seq
    ......................................acccugaucaguuauggaau..........................   1       0    seq
    ............................................aucaguuauggaauguaaagaaguaugu............   1       0    seq
    ............................................aucaguuauggaauguaaagaaguaugua...........   1       0    seq
    ..............................................caguuauggaauguaaagaaguau..............   2       0    seq
    ..............................................caguuauggaauguaaagaaguaugua...........   1       0    seq
    ...............................................aguuauggaauguaaagaaguau..............   1       0    seq
    ...............................................aguuauggaauguaaagaaguaugua...........   1       0    seq
```

cgi-miR-1-5p

cgi-miR-1-3p

ugaucucgguccauacuucuuugcuaucccauauguacacccugaucaguuauggaauguaaagaaguauguauccuugcucau

....aguuauggaauguaaagaaguauguau..........	3	0	seq
....guuauggaauguaaagaaguaugu............	1	0	seq
....guuauggaauguaaagaaguaugua...........	1	0	seq
....guuauggaauguaaagaaguauguau..........	2	0	seq
....uuauggaauguaaagaagua...............	3	0	seq
....uuauggaauguaaagaaguau..............	34	0	seq
....uuauggaauguaaagaaguaug.............	18	0	seq
....uuauggaauguaaagaaguaugu............	20	0	seq
....uuauggaauguaaagaaguaugua...........	16	0	seq
....uuauggaauguaaagaaguauguau..........	44	0	seq
....uauggaauguaaagaaguau..............	17	0	seq
....uauggaauguaaagaaguaug.............	12	0	seq
....uauggaauguaaagaaguaugu............	7	0	seq
....uauggaauguaaagaaguaugua...........	7	0	seq
....uauggaauguaaagaaguauguau..........	37	0	seq
....auggaauguaaagaagua...............	2	0	seq
....auggaauguaaagaaguau..............	71	0	seq
....auggaauguaaagaaguaug.............	33	0	seq
....auggaauguaaagaaguaugu............	121	0	seq
....auggaauguaaagaaguaugua...........	111	0	seq
....auggaauguaaagaaguauguau..........	559	0	seq
....auggaauguaaagaaguauguauc.........	2	0	seq
....uggaauguaaagaaguau..............	220703	0	seq
....uggaauguaaagaaguaug.............	286310	0	seq
....uggaauguaaagaaguaugu............	664539	0	seq
....uggaauguaaagaaguaugua...........	6821264	0	seq
....uggaauguaaagaaguauguau..........	46686132	0	seq
....uggaauguaaagaaguauguauc.........	112111	0	seq
....uggaauguaaagaaguauguaucc........	32	0	seq
....uggaauguaaagaaguauguauccu.......	1	0	seq
....ggaauguaaagaaguaug.............	362	0	seq
....ggaauguaaagaaguaugu............	1038	0	seq
....ggaauguaaagaaguaugua...........	9676	0	seq
....ggaauguaaagaaguauguau..........	76105	0	seq
....ggaauguaaagaaguauguauc.........	148	0	seq
....gaauguaaagaaguaugu............	165	0	seq
....gaauguaaagaaguaugua...........	1572	0	seq
....gaauguaaagaaguauguau..........	10473	0	seq
....gaauguaaagaaguauguauc.........	26	0	seq
....aauguaaagaaguaugua...........	31	0	seq
....aauguaaagaaguauguau..........	200	0	seq
....aauguaaagaaguauguauccu.......	1	0	seq
....auguaaagaaguauguau..........	18	0	seq
....auguaaagaaguauguauc.........	1	0	seq
....uguaaagaaguauguauc.........	1	0	seq
