# Supplementary material for: Identification of Conserved and Novel MicroRNAs in the Pacific Oyster Crassostrea gigas by Deep Sequencing
Source: PLoS One. 2014 Aug 19;9(8):e104371. doi: 10.1371/journal.pone.0104371 (PMC4138081; doi:10.1371/journal.pone.0104371)
Supplement: File S2 — The compressed/ZIP file archive for the predicted precursors' secondary structures and reads alignment. (ZIP) [file pone.0104371.s010.zip › second structure and reads alignment for oyster miRNAs/conserved in table S4/cgi-miR-100.pdf]

miRBase precursor : cgi-miR-100
Total read count : 347780
cgi-miR-100-5p read count : 346196
cgi-miR-100-3p read count : 1526
remaining reads : 58

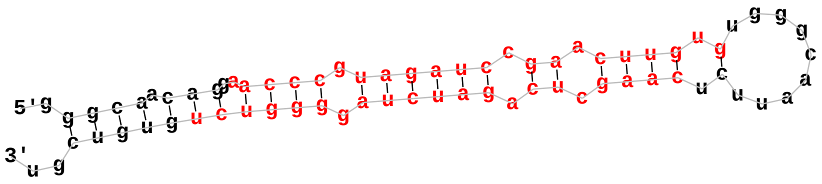

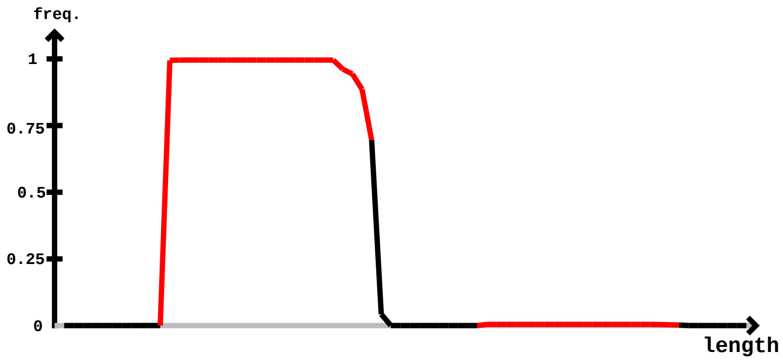


```
                                   cgi-miR-100-3p
             cgi-miR-100-5p
5'- gggcaacaggaacccguagauccgaacuugugugggcaauucucaagcucagaucuaggggucugugucgu   -3'    exp
    .((((.(((..((((.((((((.((.((((.(.........).)))).)).)))))).))))))))))..    reads      mm         sample
    .......aggaacccguagauccgaa.............................................    9          0          seq
    .......aggaacccguagauccgaac............................................    2          0          seq
    .......aggaacccguagauccgaacu...........................................    4          0          seq
    .......aggaacccguagauccgaacuu..........................................    19         0          seq
    .......aggaacccguagauccgaacuug.........................................    11         0          seq
    .........gaacccguagauccgaacuugu........................................    2          0          seq
    ..........aacccguagauccgaacu...........................................    11799      0          seq
    ..........aacccguagauccgaacuu..........................................    6383       0          seq
    ..........aacccguagauccgaacuug.........................................    19827      0          seq
    ..........aacccguagauccgaacuugu........................................    66018      0          seq
    ..........aacccguagauccgaacuugug.......................................    226985     0          seq
    ..........aacccguagauccgaacuugugu......................................    14747      0          seq
    ..........aacccguagauccgaacuugugug.....................................    5          0          seq
    ..........aacccguagauccgaacuugugugg....................................    1          0          seq
    ..........aacccguagauccgaacuugugugggca.................................    3          0          seq
    ..........aacccguagauccgaacuugugugggcaa................................    1          0          seq
    ..........aacccguagauccgaacuugugugggcaau...............................    2          0          seq
    ..........aacccguagauccgaacuugugugggcaauu..............................    1          0          seq
    ...........acccguagauccgaacuu..........................................    6          0          seq
    ...........acccguagauccgaacuug.........................................    26         0          seq
    ...........acccguagauccgaacuugu........................................    80         0          seq
    ...........acccguagauccgaacuugug.......................................    240        0          seq
    ...........acccguagauccgaacuugugu......................................    31         0          seq
    ............cccguagauccgaacuugu........................................    3          0          seq
    ............cccguagauccgaacuugug.......................................    8          0          seq
    ............cccguagauccgaacuugugu......................................    2          0          seq
    .............ccguagauccgaacuugu........................................    3          0          seq
    .............ccguagauccgaacuugug.......................................    21         0          seq
    .............ccguagauccgaacuugugu......................................    2          0          seq
    ..............cguagauccgaacuugug.......................................    7          0          seq
    ......................cgaacuugugugggcaauucucaagc.......................    1          0          seq
    ................................ugggcaauucucaagcucagaucuaggg...........    2          0          seq
    ................................ugggcaauucucaagcucagaucuagggg..........    2          0          seq
    ........................................ucucaagcucagaucuaggggu.........    1          0          seq
```

```
                                          cgi-miR-100-3p
          cgi-miR-100-5p
gggcaacaggaacccguagauccgaacuugugugggcaauucucaagcucagaucuaggggucugugucgu

...........................................caagcucagaucuagggg..........    171    0    seq
...........................................caagcucagaucuaggggu.........    261    0    seq
...........................................caagcucagaucuagggguc........    360    0    seq
...........................................caagcucagaucuaggggucu.......    700    0    seq
...........................................caagcucagaucuaggggucug......    16     0    seq
...........................................caagcucagaucuaggggucugu.....    15     0    seq
.............................................agcucagaucuagggguc........    2      0    seq
..............................................gcucagaucuaggggucu.......    1      0    seq
```
