# Supplementary material for: Identification of Conserved and Novel MicroRNAs in the Pacific Oyster Crassostrea gigas by Deep Sequencing
Source: PLoS One. 2014 Aug 19;9(8):e104371. doi: 10.1371/journal.pone.0104371 (PMC4138081; doi:10.1371/journal.pone.0104371)
Supplement: File S2 — The compressed/ZIP file archive for the predicted precursors' secondary structures and reads alignment. (ZIP) [file pone.0104371.s010.zip › second structure and reads alignment for oyster miRNAs/conserved in table S4/cgi-miR-10a.pdf]

miRBase precursor : cgi-miR-10a
Total read count : 216561
cgi-miR-10a-5p read count : 216523
cgi-miR-10a-3p read count : 34
remaining reads : 4

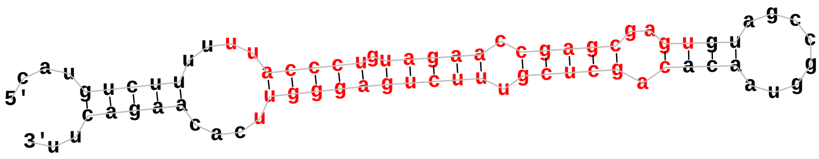

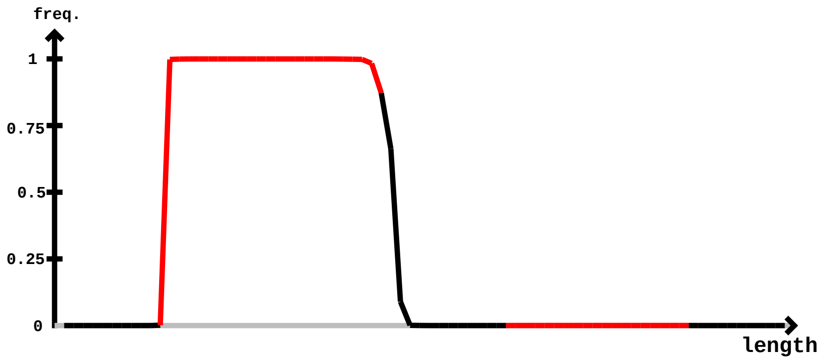


```
                                                 cgi-miR-10a-3p
            cgi-miR-10a-5p
5'- caugucuuuuuuacccuguagaaccgagcgaguguagccgguaacacagcucguuucugaggguucacaagacuu  -3'    exp
    ...(((((....(((((.(((((.(((((..((((........)))).))))).))))))))))....)))))..    reads      mm         sample
    .........uuuacccuguagaaccgagcg.............................................    1          0          seq
    .........uuuacccuguagaaccgagcga............................................    9          0          seq
    .........uuuacccuguagaaccgagcgag...........................................    47         0          seq
    .........uuuacccuguagaaccgagcgagu..........................................    78         0          seq
    .........uuuacccuguagaaccgagcgagug.........................................    141        0          seq
    .........uuuacccuguagaaccgagcgagugu........................................    26         0          seq
    .........uuuacccuguagaaccgagcgagugua.......................................    1          0          seq
    ..........uuacccuguagaaccgag...............................................    108        0          seq
    ..........uuacccuguagaaccgagc..............................................    119        0          seq
    ..........uuacccuguagaaccgagcg.............................................    147        0          seq
    ..........uuacccuguagaaccgagcga............................................    3231       0          seq
    ..........uuacccuguagaaccgagcgag...........................................    23887      0          seq
    ..........uuacccuguagaaccgagcgagu..........................................    45173      0          seq
    ..........uuacccuguagaaccgagcgagug.........................................    123805     0          seq
    ..........uuacccuguagaaccgagcgagugu........................................    19082      0          seq
    ..........uuacccuguagaaccgagcgagugua.......................................    151        0          seq
    ..........uuacccuguagaaccgagcgaguguag......................................    64         0          seq
    ..........uuacccuguagaaccgagcgaguguagcc....................................    2          0          seq
    ..........uuacccuguagaaccgagcgaguguagccg...................................    2          0          seq
    ...........uacccuguagaaccgagc..............................................    1          0          seq
    ...........uacccuguagaaccgagcga............................................    2          0          seq
    ...........uacccuguagaaccgagcgag...........................................    29         0          seq
    ...........uacccuguagaaccgagcgagu..........................................    56         0          seq
    ...........uacccuguagaaccgagcgagug.........................................    174        0          seq
    ...........uacccuguagaaccgagcgagugu........................................    62         0          seq
    ...........uacccuguagaaccgagcgagugua.......................................    1          0          seq
    ............acccuguagaaccgagcgag...........................................    12         0          seq
    ............acccuguagaaccgagcgagu..........................................    16         0          seq
    ............acccuguagaaccgagcgagug.........................................    60         0          seq
    ............acccuguagaaccgagcgagugu........................................    19         0          seq
    .............cccuguagaaccgagcgag...........................................    1          0          seq
    .............cccuguagaaccgagcgagu..........................................    2          0          seq
    .............cccuguagaaccgagcgagug.........................................    1          0          seq
    .............cccuguagaaccgagcgagugu........................................    1          0          seq
```

```
                                              cgi-miR-10a-3p
          cgi-miR-10a-5p
caugucuuuuuuacccuguagaaccgagcgaguguagccgguaacacagcucguuucugaggguucacaagacuu

..............ccuguagaaccgagcgagug.........................................    3    0    seq
...............cuguagaaccgagcgagu..........................................    1    0    seq
...............cuguagaaccgagcgagug.........................................    6    0    seq
...............cuguagaaccgagcgagugu........................................    1    0    seq
................uguagaaccgagcgagug.........................................    4    0    seq
................uguagaaccgagcgagugu........................................    1    0    seq
.............................................acagcucguuucugaggg............    1    0    seq
.............................................acagcucguuucugagggu...........    1    0    seq
.............................................acagcucguuucugaggguu..........    1    0    seq
..............................................cagcucguuucugagggu...........    6    0    seq
..............................................cagcucguuucugaggguu..........    15   0    seq
..............................................cagcucguuucugaggguuca........    1    0    seq
..............................................cagcucguuucugaggguucac.......    9    0    seq
```
