# Supplementary material for: Identification of Conserved and Novel MicroRNAs in the Pacific Oyster Crassostrea gigas by Deep Sequencing
Source: PLoS One. 2014 Aug 19;9(8):e104371. doi: 10.1371/journal.pone.0104371 (PMC4138081; doi:10.1371/journal.pone.0104371)
Supplement: File S2 — The compressed/ZIP file archive for the predicted precursors' secondary structures and reads alignment. (ZIP) [file pone.0104371.s010.zip › second structure and reads alignment for oyster miRNAs/conserved in table S4/cgi-miR-10b.pdf]

miRBase precursor : cgi-miR-10b
Total read count : 2013125
cgi-miR-10b-5p read count : 2011064
cgi-miR-10b-3p read count : 2061
remaining reads : 0

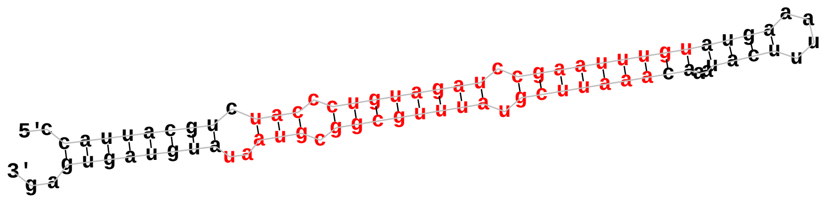

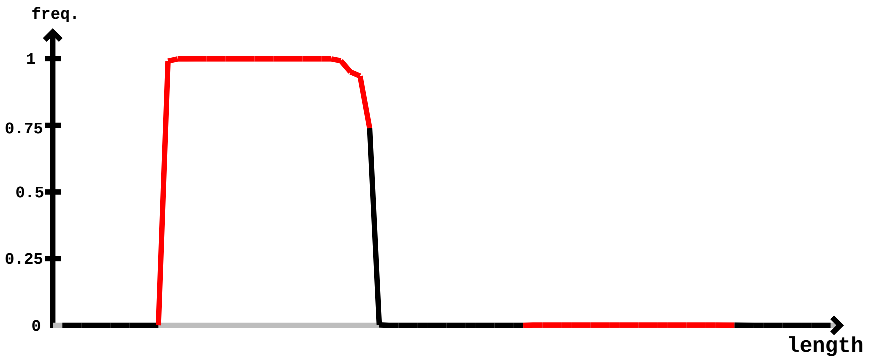


```
                   cgi-miR-10b-5p
                                                            cgi-miR-10b-3p
5'- ccauuacgucuacccuguagauccgaauuuguaugaaauuucauaaacaaauucguauuugcggcguaauauguagugag   -3'    exp
    .((((((((.(((.((((((((.(((((((((((((....))))..))))))))).)))))))).)))..)))))))))..    reads      mm         sample
    ........ucuacccuguagauccgaauuugu................................................    1          0          seq
    .........cuacccuguagauccgaauuug.................................................    4          0          seq
    .........cuacccuguagauccgaauuugu................................................    22         0          seq
    ..........uacccuguagauccgaau....................................................    14195      0          seq
    ..........uacccuguagauccgaauu...................................................    83941      0          seq
    ..........uacccuguagauccgaauuu..................................................    31286      0          seq
    ..........uacccuguagauccgaauuug.................................................    392923     0          seq
    ..........uacccuguagauccgaauuugu................................................    1468802    0          seq
    ..........uacccuguagauccgaauuugua...............................................    2938       0          seq
    ..........uacccuguagauccgaauuuguau..............................................    16         0          seq
    ..........uacccuguagauccgaauuuguaug.............................................    2          0          seq
    ...........acccuguagauccgaauu...................................................    20         0          seq
    ...........acccuguagauccgaauuu..................................................    45         0          seq
    ...........acccuguagauccgaauuug.................................................    957        0          seq
    ...........acccuguagauccgaauuugu................................................    15559      0          seq
    ...........acccuguagauccgaauuugua...............................................    275        0          seq
    ...........acccuguagauccgaauuuguau..............................................    15         0          seq
    ............cccuguagauccgaauuug.................................................    1          0          seq
    ............cccuguagauccgaauuugu................................................    4          0          seq
    .............ccuguagauccgaauuug.................................................    3          0          seq
    .............ccuguagauccgaauuugu................................................    32         0          seq
    .............ccuguagauccgaauuugua...............................................    1          0          seq
    ..............cuguagauccgaauuugu................................................    22         0          seq
    ..............................................acaaauucguauuugcggcgu.............    1          0          seq
    ...............................................caaauucguauuugcggcg..............    1          0          seq
    ...............................................caaauucguauuugcggcgu.............    2          0          seq
    ...............................................caaauucguauuugcggcgua............    3          0          seq
    ...............................................caaauucguauuugcggcguaa...........    5          0          seq
    ...............................................caaauucguauuugcggcguaau..........    8          0          seq
    ................................................aaauucguauuugcggcg..............    31         0          seq
    ................................................aaauucguauuugcggcgu.............    98         0          seq
    ................................................aaauucguauuugcggcgua............    204        0          seq
    ................................................aaauucguauuugcggcguaa...........    240        0          seq
    ................................................aaauucguauuugcggcguaau..........    731        0          seq
```

```
          cgi-miR-10b-5p
                                                cgi-miR-10b-3p
ccauuacgucuacccuguagauccgaauuuguaugaaauuucauaaacaaauucguauuugcggcguaauauguagugag

................................................aaauucguauuugcggcguaaua.........    621    0    seq
................................................aaauucguauuugcggcguaauau........    9      0    seq
.................................................aauucguauuugcggcgu.............    2      0    seq
.................................................aauucguauuugcggcgua............    7      0    seq
.................................................aauucguauuugcggcguaa...........    5      0    seq
.................................................aauucguauuugcggcguaau..........    23     0    seq
.................................................aauucguauuugcggcguaaua.........    40     0    seq
..................................................auucguauuugcggcgua............    1      0    seq
..................................................auucguauuugcggcguaa...........    1      0    seq
..................................................auucguauuugcggcguaau..........    3      0    seq
..................................................auucguauuugcggcguaaua.........    13     0    seq
..................................................auucguauuugcggcguaauau........    6      0    seq
...................................................uucguauuugcggcguaaua.........    4      0    seq
...................................................uucguauuugcggcguaauau........    2      0    seq
```
