# Supplementary material for: Identification of Conserved and Novel MicroRNAs in the Pacific Oyster Crassostrea gigas by Deep Sequencing
Source: PLoS One. 2014 Aug 19;9(8):e104371. doi: 10.1371/journal.pone.0104371 (PMC4138081; doi:10.1371/journal.pone.0104371)
Supplement: File S2 — The compressed/ZIP file archive for the predicted precursors' secondary structures and reads alignment. (ZIP) [file pone.0104371.s010.zip › second structure and reads alignment for oyster miRNAs/conserved in table S4/cgi-miR-1175.pdf]

miRBase precursor : cgi-miR-1175
Total read count : 2893
cgi-miR-1175-5p read count: 1090
cgi-miR-1175-3p read count: 1802
remaining reads : 1

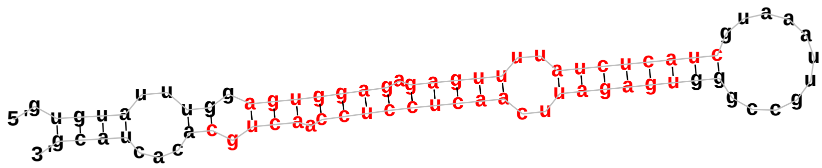

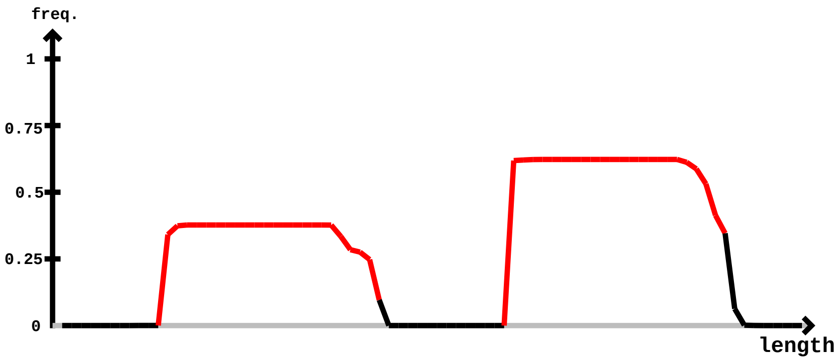


```
                                 cgi-miR-1175-5p
                                                                  cgi-miR-1175-3p
5'- guguauuuggaguggagagaguuuuaucucaucguaaauugccgggugagauucaacuccuccaacugcacacuacg   -3'    exp
    ((((....(.(((((((.(((((..((((((((...........))))))))..))))))))).))).)))))....    reads      mm        sample
    .......uggaguggagagaguuuuau..................................................    1          0         seq
    ..........aguggagagaguuuuauc.................................................    123        0         seq
    ..........aguggagagaguuuuaucu................................................    142        0         seq
    ..........aguggagagaguuuuaucuc...............................................    26         0         seq
    ..........aguggagagaguuuuaucuca..............................................    70         0         seq
    ..........aguggagagaguuuuaucucau.............................................    387        0         seq
    ..........aguggagagaguuuuaucucauc............................................    238        0         seq
    ...........guggagagaguuuuaucu................................................    2          0         seq
    ...........guggagagaguuuuaucuca..............................................    11         0         seq
    ...........guggagagaguuuuaucucau.............................................    46         0         seq
    ...........guggagagaguuuuaucucauc............................................    36         0         seq
    ............uggagagaguuuuaucucau.............................................    5          0         seq
    ............uggagagaguuuuaucucauc............................................    4          0         seq
    ..............................................ugagauucaacuccucca.............    31         0         seq
    ..............................................ugagauucaacuccuccaa............    71         0         seq
    ..............................................ugagauucaacuccuccaac...........    162        0         seq
    ..............................................ugagauucaacuccuccaacu..........    340        0         seq
    ..............................................ugagauucaacuccuccaacug.........    193        0         seq
    ..............................................ugagauucaacuccuccaacugc........    816        0         seq
    ..............................................ugagauucaacuccuccaacugca.......    177        0         seq
    ...............................................gagauucaacuccuccaac...........    1          0         seq
    ...............................................gagauucaacuccuccaacu..........    1          0         seq
    ...............................................gagauucaacuccuccaacug.........    1          0         seq
    ...............................................gagauucaacuccuccaacugc........    2          0         seq
    ...............................................gagauucaacuccuccaacugca.......    1          0         seq
    ................................................agauucaacuccuccaacug.........    1          0         seq
    ................................................agauucaacuccuccaacugcac......    3          0         seq
    ................................................agauucaacuccuccaacugcaca.....    1          0         seq
    .................................................gauucaacuccuccaacugc........    1          0         seq
```
