# Supplementary material for: Identification of Conserved and Novel MicroRNAs in the Pacific Oyster Crassostrea gigas by Deep Sequencing
Source: PLoS One. 2014 Aug 19;9(8):e104371. doi: 10.1371/journal.pone.0104371 (PMC4138081; doi:10.1371/journal.pone.0104371)
Supplement: File S2 — The compressed/ZIP file archive for the predicted precursors' secondary structures and reads alignment. (ZIP) [file pone.0104371.s010.zip › second structure and reads alignment for oyster miRNAs/conserved in table S4/cgi-miR-12.pdf]

miRBase precursor	: cgi-miR-12
Total read count	: 107131
cgi-miR-12-5p read count	: 106382
cgi-miR-12-3p read count	: 748
remaining reads	: 1

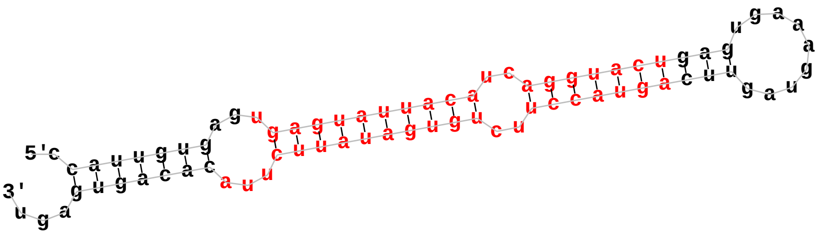

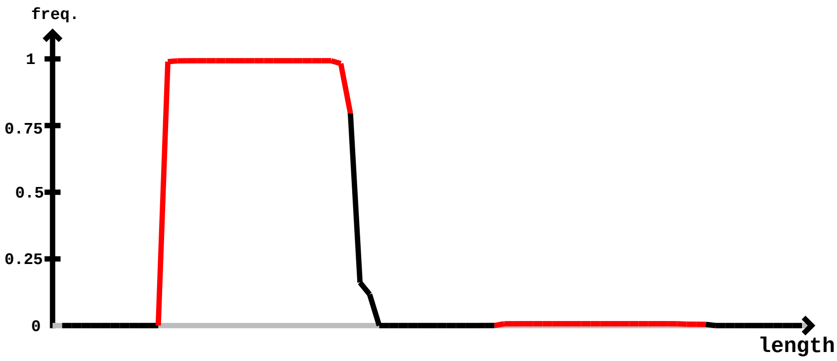


```
                cgi-miR-12-5p
                                  cgi-miR-12-3p
5'- ccauugugagugaguauuacaucagguacugagugaaaguaguucaguaccuucugugauauucuuacacagugagu   -3'    exp
    .(((((((...((((((((((..((((((((((.........))))))))))..))))))))))...)))))))...    reads      mm         sample
    ........agugaguauuacaucaggua.................................................    1          0          seq
    .........gugaguauuacaucagguacu...............................................    1          0          seq
    .........gugaguauuacaucagguacug..............................................    1          0          seq
    .........gugaguauuacaucagguacuga.............................................    1          0          seq
    ..........ugaguauuacaucaggua.................................................    1131       0          seq
    ..........ugaguauuacaucagguac................................................    19925      0          seq
    ..........ugaguauuacaucagguacu...............................................    67755      0          seq
    ..........ugaguauuacaucagguacug..............................................    4722       0          seq
    ..........ugaguauuacaucagguacuga.............................................    12478      0          seq
    ..........ugaguauuacaucagguacugag............................................    1          0          seq
    ...........gaguauuacaucagguac................................................    79         0          seq
    ...........gaguauuacaucagguacu...............................................    175        0          seq
    ...........gaguauuacaucagguacug..............................................    11         0          seq
    ...........gaguauuacaucagguacuga.............................................    47         0          seq
    ............aguauuacaucagguacu...............................................    30         0          seq
    ............aguauuacaucagguacug..............................................    5          0          seq
    ............aguauuacaucagguacuga.............................................    11         0          seq
    .............guauuacaucagguacuga.............................................    8          0          seq
    .....................ucagguacugagugaaaguaguuc................................    1          0          seq
    ...........................................ucaguaccuucugugauauu..............    1          0          seq
    ...........................................ucaguaccuucugugauauuc.............    13         0          seq
    ...........................................ucaguaccuucugugauauucu............    6          0          seq
    ...........................................ucaguaccuucugugauauucuu...........    2          0          seq
    .............................................aguaccuucugugauauu..............    20         0          seq
    .............................................aguaccuucugugauauuc.............    152        0          seq
    .............................................aguaccuucugugauauucu............    24         0          seq
    .............................................aguaccuucugugauauucuu...........    51         0          seq
    .............................................aguaccuucugugauauucuua..........    453        0          seq
    ..............................................guaccuucugugauauuc.............    2          0          seq
    ..............................................guaccuucugugauauucuua..........    14         0          seq
    ...............................................uaccuucugugauauucuua..........    8          0          seq
    ................................................accuucugugauauucuua..........    2          0          seq
```
