# Supplementary material for: Identification of Conserved and Novel MicroRNAs in the Pacific Oyster Crassostrea gigas by Deep Sequencing
Source: PLoS One. 2014 Aug 19;9(8):e104371. doi: 10.1371/journal.pone.0104371 (PMC4138081; doi:10.1371/journal.pone.0104371)
Supplement: File S2 — The compressed/ZIP file archive for the predicted precursors' secondary structures and reads alignment. (ZIP) [file pone.0104371.s010.zip › second structure and reads alignment for oyster miRNAs/conserved in table S4/cgi-miR-124.pdf]

miRBase precursor : cgi-miR-124
Total read count : 28856
cgi-miR-124-5p read count : 33
cgi-miR-124-3p read count : 28815
remaining reads : 8

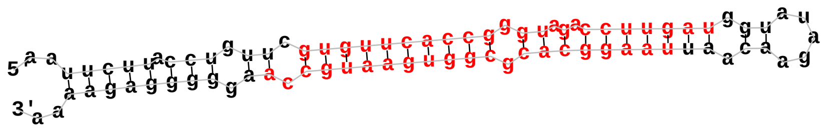

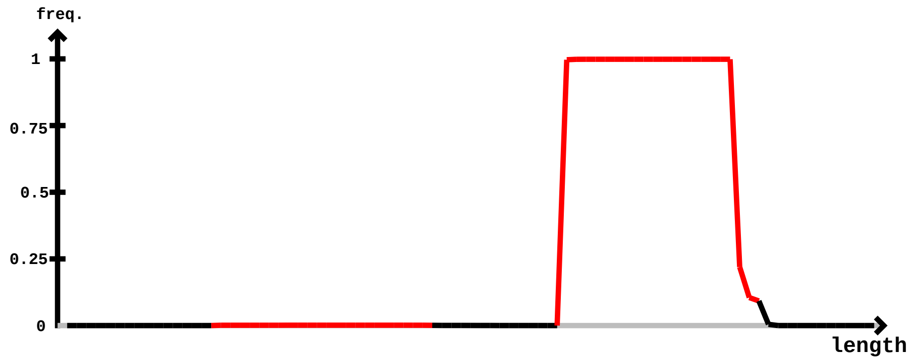


```
                                                   cgi-miR-124-3p
               cgi-miR-124-5p
5'- aauucuuaccuguucguguucaccggguagaccuugauggguauagaacaauuaaggcacgcggugaaugccaagggggagaaaa  -3'   exp
    ..(((((.(((.((.((((((((((.((.(.(((((((.((.....)).)))))))))).))))))))))).)).))))))))..    reads    mm    sample
    ...............guguucaccggguagaccu..................................................    2        0     seq
    ...............guguucaccggguagaccuu.................................................    1        0     seq
    ...............guguucaccggguagaccuug................................................    1        0     seq
    ...............guguucaccggguagaccuuga...............................................    11       0     seq
    ...............guguucaccggguagaccuugau..............................................    13       0     seq
    ...............guguucaccggguagaccuugauggu...........................................    1        0     seq
    ...............guguucaccggguagaccuugauggua..........................................    3        0     seq
    ...............guguucaccggguagaccuugaugguau.........................................    1        0     seq
    ...............guguucaccggguagaccuugaugguaua........................................    2        0     seq
    ......................ccggguagaccuugaugguaua........................................    3        0     seq
    ......................ccggguagaccuugaugguauagaaca...................................    2        0     seq
    ..................................ugaugguauagaacaauuaaggcac.........................    1        0     seq
    ...................................................uaaggcacgcggugaaug...............    22473    0     seq
    ...................................................uaaggcacgcggugaaugc..............    3288     0     seq
    ...................................................uaaggcacgcggugaaugcc.............    364      0     seq
    ...................................................uaaggcacgcggugaaugcca............    2508     0     seq
    ...................................................uaaggcacgcggugaaugccaa...........    131      0     seq
    ....................................................aaggcacgcggugaaugc..............    24       0     seq
    ....................................................aaggcacgcggugaaugcc.............    2        0     seq
    ....................................................aaggcacgcggugaaugcca............    16       0     seq
    ....................................................aaggcacgcggugaaugccaa...........    3        0     seq
    .....................................................aggcacgcggugaaugcca............    5        0     seq
    .....................................................aggcacgcggugaaugccaa...........    1        0     seq
```
