# Supplementary material for: Identification of Conserved and Novel MicroRNAs in the Pacific Oyster Crassostrea gigas by Deep Sequencing
Source: PLoS One. 2014 Aug 19;9(8):e104371. doi: 10.1371/journal.pone.0104371 (PMC4138081; doi:10.1371/journal.pone.0104371)
Supplement: File S2 — The compressed/ZIP file archive for the predicted precursors' secondary structures and reads alignment. (ZIP) [file pone.0104371.s010.zip › second structure and reads alignment for oyster miRNAs/conserved in table S4/cgi-miR-125.pdf]

miRBase precursor : cgi-miR-125
Total read count : 2758901
cgi-miR-125-5p read count : 2758024
cgi-miR-125-3p read count : 874
remaining reads : 3

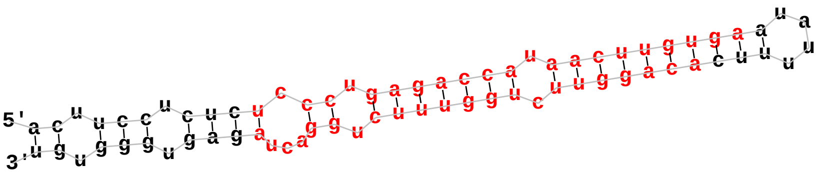

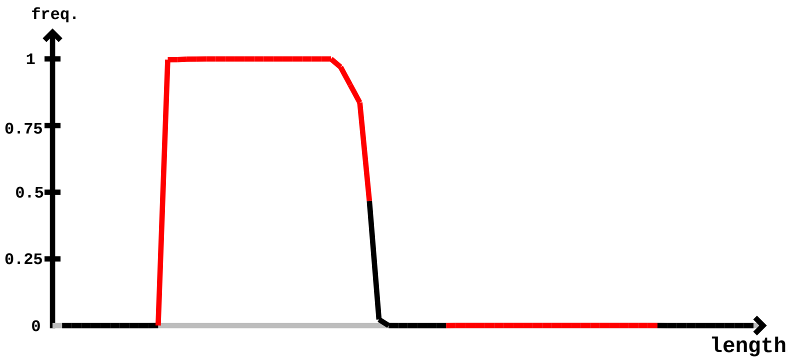


```
                                                      cgi-miR-125-3p
                cgi-miR-125-5p
5'- acuuccucucucccugagaccauaacuugugaauauuuucacagguucugguuucuggacuagagugggugu   -3'    exp
    ((.(((.((((.((.(((((((.((((((((((....)))))))))).))))))).))...)))).))).))    reads      mm         sample
    ........ucucccugagaccauaacu.............................................    1          0          seq
    ........ucucccugagaccauaacuug...........................................    1          0          seq
    ........ucucccugagaccauaacuugu..........................................    1          0          seq
    ........ucucccugagaccauaacuugug.........................................    3          0          seq
    ........ucucccugagaccauaacuuguga........................................    8          0          seq
    .........cucccugagaccauaacu.............................................    66         0          seq
    .........cucccugagaccauaacuu............................................    20         0          seq
    .........cucccugagaccauaacuug...........................................    39         0          seq
    .........cucccugagaccauaacuugu..........................................    46         0          seq
    .........cucccugagaccauaacuugug.........................................    235        0          seq
    .........cucccugagaccauaacuuguga........................................    191        0          seq
    .........cucccugagaccauaacuugugaa.......................................    10         0          seq
    ..........ucccugagaccauaacuu............................................    83692      0          seq
    ..........ucccugagaccauaacuug...........................................    183194     0          seq
    ..........ucccugagaccauaacuugu..........................................    183255     0          seq
    ..........ucccugagaccauaacuugug.........................................    1016497    0          seq
    ..........ucccugagaccauaacuuguga........................................    1221012    0          seq
    ..........ucccugagaccauaacuugugaa.......................................    62149      0          seq
    ..........ucccugagaccauaacuugugaau......................................    140        0          seq
    ..........ucccugagaccauaacuugugaauauuu..................................    1          0          seq
    ...........cccugagaccauaacuug...........................................    39         0          seq
    ...........cccugagaccauaacuugu..........................................    39         0          seq
    ...........cccugagaccauaacuugug.........................................    318        0          seq
    ...........cccugagaccauaacuuguga........................................    263        0          seq
    ...........cccugagaccauaacuugugaa.......................................    22         0          seq
    ...........cccugagaccauaacuugugaau......................................    8          0          seq
    ............ccugagaccauaacuugu..........................................    463        0          seq
    ............ccugagaccauaacuugug.........................................    1608       0          seq
    ............ccugagaccauaacuuguga........................................    2556       0          seq
    ............ccugagaccauaacuugugaa.......................................    146        0          seq
    .............cugagaccauaacuugug.........................................    162        0          seq
    .............cugagaccauaacuuguga........................................    377        0          seq
    .............cugagaccauaacuugugaa.......................................    19         0          seq
    ..............ugagaccauaacuuguga........................................    1359       0          seq
```

```
                                        cgi-miR-125-3p
          cgi-miR-125-5p
acuuccucucucccugagaccauaacuugugaauauuuucacagguucugguuucuggacuagagugggugu

..............ugagaccauaacuugugaa.......................................    85    0    seq
................................auauuuucacagguucugguuuc.................    1     0    seq
.................................uauuuucacagguucugguuuc.................    1     0    seq
......................................ucacagguucugguuucu................    1     0    seq
........................................acagguucugguuucugg..............    33    0    seq
........................................acagguucugguuucugga.............    42    0    seq
........................................acagguucugguuucuggac............    86    0    seq
........................................acagguucugguuucuggacu...........    26    0    seq
........................................acagguucugguuucuggacua..........    681   0    seq
.........................................cagguucugguuucuggacu...........    1     0    seq
.........................................cagguucugguuucuggacua..........    4     0    seq
```
