# Supplementary material for: Identification of Conserved and Novel MicroRNAs in the Pacific Oyster Crassostrea gigas by Deep Sequencing
Source: PLoS One. 2014 Aug 19;9(8):e104371. doi: 10.1371/journal.pone.0104371 (PMC4138081; doi:10.1371/journal.pone.0104371)
Supplement: File S2 — The compressed/ZIP file archive for the predicted precursors' secondary structures and reads alignment. (ZIP) [file pone.0104371.s010.zip › second structure and reads alignment for oyster miRNAs/conserved in table S4/cgi-miR-133.pdf]

miRBase precursor : cgi-miR-133
Total read count : 51366
cgi-miR-133-5p read count : 13270
cgi-miR-133-3p read count : 38091
remaining reads : 5

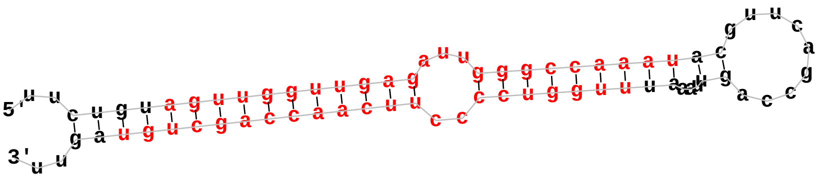

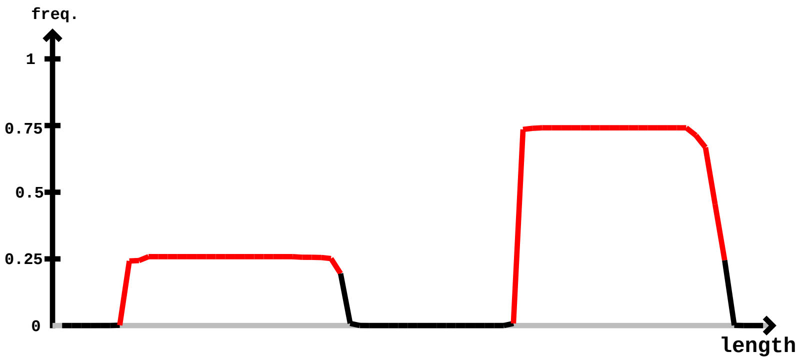

cgi-miR-133-5p

cgi-miR-133-3p

5'- uucuguaguugguugagauugggccaaauacguucagccaguuaaauuugguccccuucaaccagcuguaguu -3'	exp		
..(((((((((((((((...(((((((((((.........))...)))))))))..))))))))))))))))..	reads	mm	sample
...uguaguugguugagauugg...	1	0	seq
...uguaguugguugagauugggcc..	2	0	seq
.....uaguugguugagauuggg..	6	0	seq
.....uaguugguugagauugggc...	6	0	seq
.....uaguugguugagauugggcc..	1	0	seq
.....uaguugguugagauugggcca...	1	0	seq
.....uaguugguugagauugggccaa..	2	0	seq
.....uaguugguugagauugggccaaa...	22	0	seq
.....uaguugguugagauugggccaaau..	19	0	seq
......aguugguugagauugggc...	100	0	seq
......aguugguugagauugggcc..	7	0	seq
......aguugguugagauugggcca...	24	0	seq
......aguugguugagauugggccaa..	180	0	seq
......aguugguugagauugggccaaa...	2806	0	seq
......aguugguugagauugggccaaau..	8977	0	seq
......aguugguugagauugggccaaaua...	289	0	seq
.......guugguugagauugggccaa..	2	0	seq
.......guugguugagauugggccaaa...	12	0	seq
.......guugguugagauugggccaaau..	55	0	seq
.......guugguugagauugggccaaaua...	11	0	seq
........uugguugagauugggcca...	23	0	seq
........uugguugagauugggccaa..	10	0	seq
........uugguugagauugggccaaa...	47	0	seq
........uugguugagauugggccaaau..	551	0	seq
........uugguugagauugggccaaaua...	105	0	seq
........uugguugagauugggccaaauac..	13	0	seq
.........ugguugagauugggccaaau..	1	0	seq
....aauuugguccccuucaacca.........	1	0	seq
....aauuugguccccuucaaccag........	1	0	seq
....auuugguccccuucaaccagc.......	1	0	seq
....auuugguccccuucaaccagcu......	1	0	seq
....uuugguccccuucaacca.........	5	0	seq
....uuugguccccuucaaccag........	9	0	seq
....uuugguccccuucaaccagc.......	8	0	seq

```
      cgi-miR-133-5p
                                          cgi-miR-133-3p
uucuguaguugguugagauugggccaaauacguucagccaguuaaauuugguccccuucaaccagcuguaguu

..............................................uuugguccccuucaaccagcu......    42      0      seq
..............................................uuugguccccuucaaccagcug.....    149     0      seq
..............................................uuugguccccuucaaccagcugu....    191     0      seq
...............................................uugguccccuucaaccag........    1449    0      seq
...............................................uugguccccuucaaccagc.......    2299    0      seq
...............................................uugguccccuucaaccagcu......    10723   0      seq
...............................................uugguccccuucaaccagcug.....    10610   0      seq
...............................................uugguccccuucaaccagcugu....    12249   0      seq
...............................................uugguccccuucaaccagcugua...    42      0      seq
................................................ugguccccuucaaccagc.......    8       0      seq
................................................ugguccccuucaaccagcu......    123     0      seq
................................................ugguccccuucaaccagcug.....    31      0      seq
................................................ugguccccuucaaccagcugu....    53      0      seq
................................................ugguccccuucaaccagcugua...    1       0      seq
.................................................gguccccuucaaccagcu......    37      0      seq
.................................................gguccccuucaaccagcug.....    20      0      seq
.................................................gguccccuucaaccagcugu....    39      0      seq
.................................................gguccccuucaaccagcugua...    1       0      seq
```
