# Supplementary material for: Identification of Conserved and Novel MicroRNAs in the Pacific Oyster Crassostrea gigas by Deep Sequencing
Source: PLoS One. 2014 Aug 19;9(8):e104371. doi: 10.1371/journal.pone.0104371 (PMC4138081; doi:10.1371/journal.pone.0104371)
Supplement: File S2 — The compressed/ZIP file archive for the predicted precursors' secondary structures and reads alignment. (ZIP) [file pone.0104371.s010.zip › second structure and reads alignment for oyster miRNAs/conserved in table S4/cgi-miR-137.pdf]

miRBase precursor : cgi-miR-137
Total read count : 1971
cgi-miR-137-5p read count : 5
cgi-miR-137-3p read count : 1966
remaining reads : 0

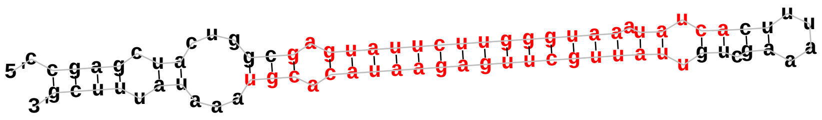

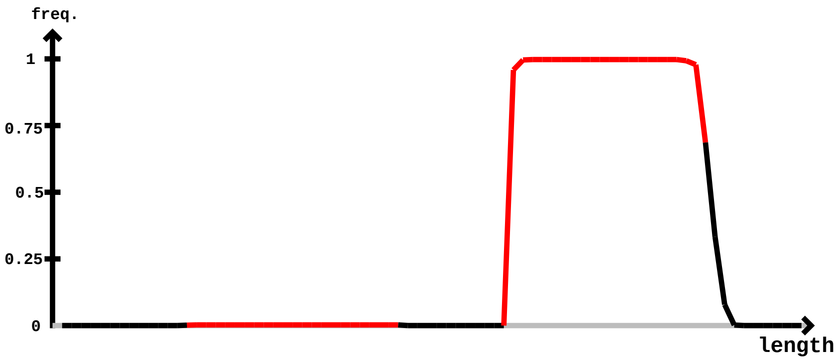

cgi-miR-137-3p

cgi-miR-137-5p

```
5'- ccgagcuacuggcgaguauucuuggguaaaauaucacuuuaaagcuguuauugcuugagaauacacguaaauauuucg   -3'    exp
    .((((.((...(((.((((((((((((((.((.((((....)).)).)))))))))))))))).)))...)).))))   reads      mm         sample
    ............cgaguauucuuggguaaaauauca..........................................   3          0          seq
    .............gaguauucuuggguaaaauauca..........................................   2          0          seq
    ...............................................uuauugcuugagaauaca.............   9          0          seq
    ...............................................uuauugcuugagaauacac............   29         0          seq
    ...............................................uuauugcuugagaauacacg...........   560        0          seq
    ...............................................uuauugcuugagaauacacgu..........   690        0          seq
    ...............................................uuauugcuugagaauacacgua.........   491        0          seq
    ...............................................uuauugcuugagaauacacguaa........   106        0          seq
    ...............................................uuauugcuugagaauacacguaaa.......   4          0          seq
    ................................................uauugcuugagaauacacg...........   14         0          seq
    ................................................uauugcuugagaauacacgu..........   7          0          seq
    ................................................uauugcuugagaauacacgua.........   8          0          seq
    ................................................uauugcuugagaauacacguaa........   45         0          seq
    .................................................auugcuugagaauacacg...........   1          0          seq
    .................................................auugcuugagaauacacgua.........   2          0          seq
```
