# Supplementary material for: Identification of Conserved and Novel MicroRNAs in the Pacific Oyster Crassostrea gigas by Deep Sequencing
Source: PLoS One. 2014 Aug 19;9(8):e104371. doi: 10.1371/journal.pone.0104371 (PMC4138081; doi:10.1371/journal.pone.0104371)
Supplement: File S2 — The compressed/ZIP file archive for the predicted precursors' secondary structures and reads alignment. (ZIP) [file pone.0104371.s010.zip › second structure and reads alignment for oyster miRNAs/conserved in table S4/cgi-miR-153.pdf]

miRBase precursor : cgi-miR-153
Total read count : 1093
cgi-miR-153-5p read count : 90
cgi-miR-153-3p read count : 1003
remaining reads : 0

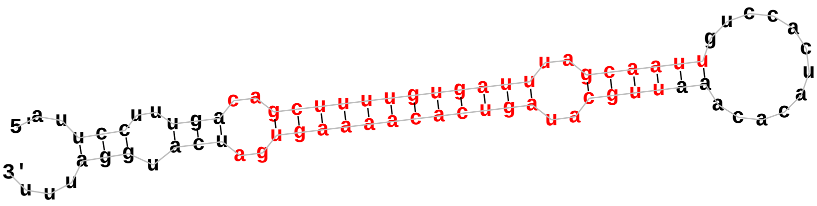

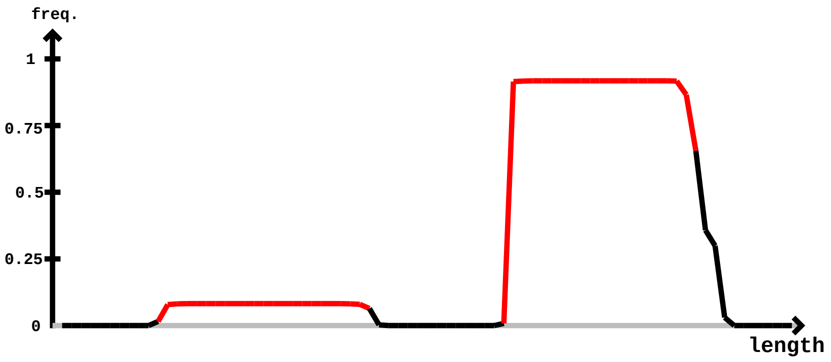


```
                                                    cgi-miR-153-3p
          cgi-miR-153-5p
5'- auuccuugacagcuuuugugauuuagcaauuguccacuacacaaauugcauagucacaaaagugaucauggauuu   -3'    exp
    ..(((..(((..((((((((((((..((((((............))))))..))))))))))))..))).)))...    reads      mm         sample
    .........acagcuuuugugauuuagcaau.............................................    5          0          seq
    .........acagcuuuugugauuuagcaauu............................................    12         0          seq
    ..........cagcuuuugugauuuagca...............................................    1          0          seq
    ..........cagcuuuugugauuuagcaa..............................................    2          0          seq
    ..........cagcuuuugugauuuagcaau.............................................    12         0          seq
    ..........cagcuuuugugauuuagcaauu............................................    52         0          seq
    ..........cagcuuuugugauuuagcaauug...........................................    2          0          seq
    ...........agcuuuugugauuuagcaauu............................................    2          0          seq
    ...........agcuuuugugauuuagcaauug...........................................    1          0          seq
    ............gcuuuugugauuuagcaauu............................................    1          0          seq
    .............................................auugcauagucacaaaag.............    1          0          seq
    .............................................auugcauagucacaaaaguga..........    2          0          seq
    .............................................auugcauagucacaaaagugau.........    3          0          seq
    .............................................auugcauagucacaaaagugauc........    2          0          seq
    .............................................auugcauagucacaaaagugauca.......    1          0          seq
    ..............................................uugcauagucacaaaagu............    56         0          seq
    ..............................................uugcauagucacaaaagug...........    230        0          seq
    ..............................................uugcauagucacaaaaguga..........    321        0          seq
    ..............................................uugcauagucacaaaagugau.........    61         0          seq
    ..............................................uugcauagucacaaaagugauc........    292        0          seq
    ..............................................uugcauagucacaaaagugauca.......    31         0          seq
    ...............................................ugcauagucacaaaaguga..........    1          0          seq
    ...............................................ugcauagucacaaaagugauca.......    1          0          seq
    ................................................gcauagucacaaaaguga..........    1          0          seq
```
