# Supplementary material for: Identification of Conserved and Novel MicroRNAs in the Pacific Oyster Crassostrea gigas by Deep Sequencing
Source: PLoS One. 2014 Aug 19;9(8):e104371. doi: 10.1371/journal.pone.0104371 (PMC4138081; doi:10.1371/journal.pone.0104371)
Supplement: File S2 — The compressed/ZIP file archive for the predicted precursors' secondary structures and reads alignment. (ZIP) [file pone.0104371.s010.zip › second structure and reads alignment for oyster miRNAs/conserved in table S4/cgi-miR-1692-1.pdf]

miRBase precursor : cgi-miR-1692-1
Total read count : 4182
cgi-miR-1692-2-5p read count : 2090
cgi-miR-1692-2-35p read count : 1
cgi-miR-1692-1-5p read count : 2090
cgi-miR-1692-1-35p read count : 1
remaining reads : 0

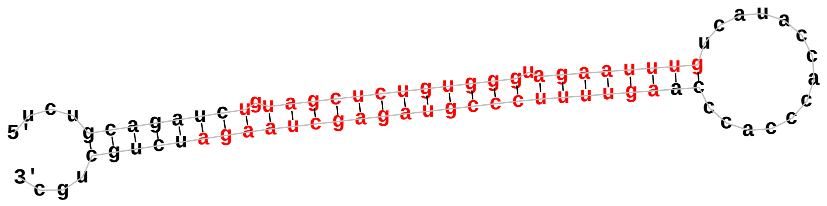

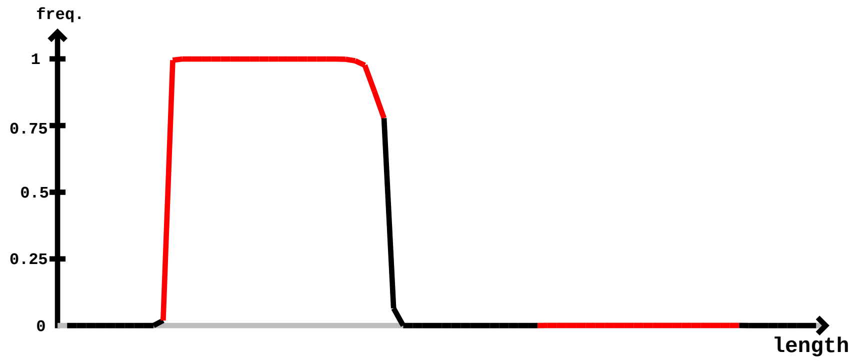

cgi-miR-1692-2-5p
cgi-miR-1692-1-35p
cgi-miR-1692-1-5p
cgi-miR-1692-2-35p

```
5'- ucugcagaucuguagcucuguggguagaauuugucauaccacccacccaaguuuucccguagagcuaagaucugcugc   -3'    exp
    .(.((((((((.((((((((((((.((((((((..............)))))))))))))))))))))))))))).).    reads      mm         sample
    .........cuguagcucuguggguagaa.................................................    1          0          seq
    .........cuguagcucuguggguagaauu...............................................    3          0          seq
    .........cuguagcucuguggguagaauuu..............................................    7          0          seq
    .........cuguagcucuguggguagaauuug.............................................    26         0          seq
    .........cuguagcucuguggguagaauuugu............................................    3          0          seq
    ..........uguagcucuguggguaga..................................................    2          0          seq
    ..........uguagcucuguggguagaa.................................................    11         0          seq
    ..........uguagcucuguggguagaau................................................    35         0          seq
    ..........uguagcucuguggguagaauu...............................................    201        0          seq
    ..........uguagcucuguggguagaauuu..............................................    204        0          seq
    ..........uguagcucuguggguagaauuug.............................................    1459       0          seq
    ..........uguagcucuguggguagaauuugu............................................    129        0          seq
    ...........guagcucuguggguagaauuug.............................................    6          0          seq
    ...........guagcucuguggguagaauuugu............................................    3          0          seq
    .................................................aguuuucccguagagcuaaga........    1          0          seq
```
