# Supplementary material for: Identification of Conserved and Novel MicroRNAs in the Pacific Oyster Crassostrea gigas by Deep Sequencing
Source: PLoS One. 2014 Aug 19;9(8):e104371. doi: 10.1371/journal.pone.0104371 (PMC4138081; doi:10.1371/journal.pone.0104371)
Supplement: File S2 — The compressed/ZIP file archive for the predicted precursors' secondary structures and reads alignment. (ZIP) [file pone.0104371.s010.zip › second structure and reads alignment for oyster miRNAs/conserved in table S4/cgi-miR-1692-2.pdf]

miRBase precursor	: cgi-miR-1692-2
Total read count	: 4182
cgi-miR-1692-2-5p read count	: 2090
cgi-miR-1692-2-35p read count	: 1
cgi-miR-1692-1-5p read count	: 2090
cgi-miR-1692-1-35p read count	: 1
remaining reads	: 0

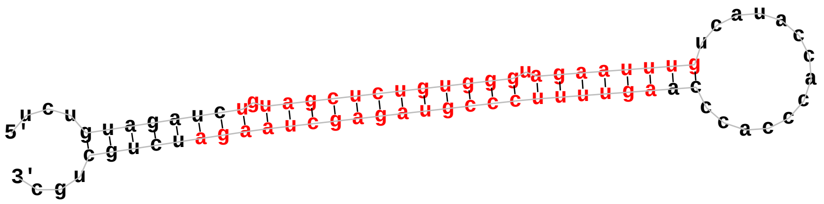

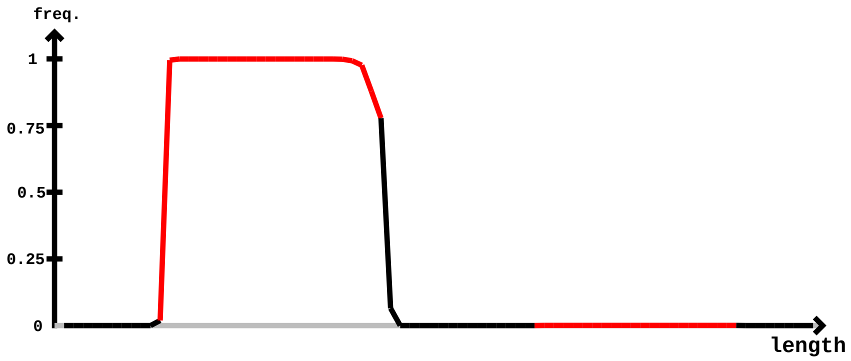


```
           cgi-miR-1692-2-5p
                                                     cgi-miR-1692-1-35p
           cgi-miR-1692-1-5p
                                                     cgi-miR-1692-2-35p
5'- ucuguagaucuguagcucugugggguagaauuugucauaccacccacccaaguuuucccguagagcuaagaucugcugc   -3'    exp
    .(.((((((((.((((((((((((.((((((((.............)))))))))))))))))))))))))))).).   reads      mm         sample
    .........cuguagcucugugggguagaa.............................................     1          0          seq
    .........cuguagcucugugggguagaauu...........................................     3          0          seq
    .........cuguagcucugugggguagaauuu..........................................     7          0          seq
    .........cuguagcucugugggguagaauuug.........................................     26         0          seq
    .........cuguagcucugugggguagaauuugu........................................     3          0          seq
    ..........uguagcucugugggguaga..............................................     2          0          seq
    ..........uguagcucugugggguagaa.............................................     11         0          seq
    ..........uguagcucugugggguagaau............................................     35         0          seq
    ..........uguagcucugugggguagaauu...........................................     201        0          seq
    ..........uguagcucugugggguagaauuu..........................................     204        0          seq
    ..........uguagcucugugggguagaauuug.........................................     1459       0          seq
    ..........uguagcucugugggguagaauuugu........................................     129        0          seq
    ............guagcucugugggguagaauuug........................................     6          0          seq
    ............guagcucugugggguagaauuugu.......................................     3          0          seq
    ..................................................aguuuucccguagagcuaaga........     1          0          seq
```
