# Supplementary material for: Identification of Conserved and Novel MicroRNAs in the Pacific Oyster Crassostrea gigas by Deep Sequencing
Source: PLoS One. 2014 Aug 19;9(8):e104371. doi: 10.1371/journal.pone.0104371 (PMC4138081; doi:10.1371/journal.pone.0104371)
Supplement: File S2 — The compressed/ZIP file archive for the predicted precursors' secondary structures and reads alignment. (ZIP) [file pone.0104371.s010.zip › second structure and reads alignment for oyster miRNAs/conserved in table S4/cgi-miR-182.pdf]

miRBase precursor : cgi-miR-182
Total read count : 121345
cgi-miR-182-5p read count : 109660
cgi-miR-182-3p read count : 11670
remaining reads : 15

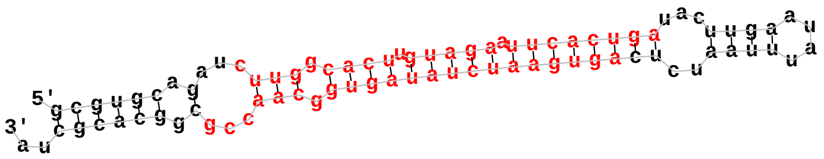

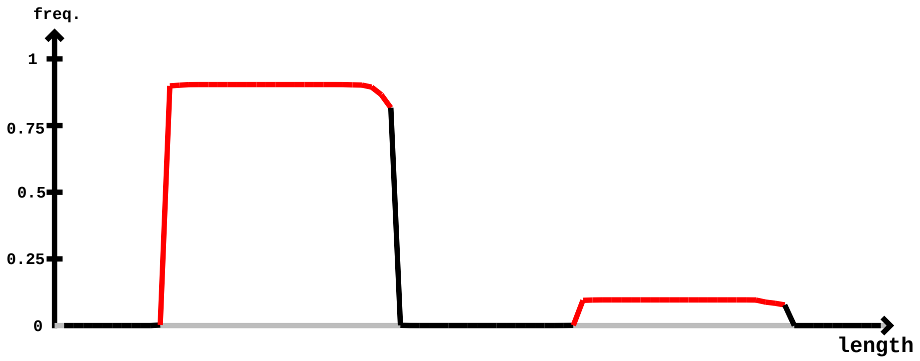


```
                                                    cgi-miR-182-3p
              cgi-miR-182-5p
5'- gcgugcagaucuuggcacuuguagaauucacugauacuugaauauuuaaaucucagugaaucuauaguggcaaccgcggcacgcua  -3'    exp
    ((((((.(...(((.((((.(((((.((((((((...((((....))))..)))))))))))))))).)))...).))))))..    reads      mm        sample
    ........aucuuggcacuuguagaauucac.......................................................    1          0         seq
    .........ucuuggcacuuguagaau...........................................................    1          0         seq
    .........ucuuggcacuuguagaauu..........................................................    2          0         seq
    .........ucuuggcacuuguagaauuca........................................................    2          0         seq
    .........ucuuggcacuuguagaauucac.......................................................    13         0         seq
    .........ucuuggcacuuguagaauucacu......................................................    53         0         seq
    .........ucuuggcacuuguagaauucacug.....................................................    135        0         seq
    .........ucuuggcacuuguagaauucacuga....................................................    51         0         seq
    ..........cuuggcacuuguagaauu..........................................................    12         0         seq
    ..........cuuggcacuuguagaauuc.........................................................    120        0         seq
    ..........cuuggcacuuguagaauuca........................................................    89         0         seq
    ..........cuuggcacuuguagaauucac.......................................................    912        0         seq
    ..........cuuggcacuuguagaauucacu......................................................    3326       0         seq
    ..........cuuggcacuuguagaauucacug.....................................................    5825       0         seq
    ..........cuuggcacuuguagaauucacuga....................................................    98431      0         seq
    ..........cuuggcacuuguagaauucacugau...................................................    75         0         seq
    ..........cuuggcacuuguagaauucacugaua..................................................    1          0         seq
    ..........cuuggcacuuguagaauucacugauac.................................................    2          0         seq
    ...........uuggcacuuguagaauucac.......................................................    3          0         seq
    ...........uuggcacuuguagaauucacu......................................................    10         0         seq
    ...........uuggcacuuguagaauucacug.....................................................    23         0         seq
    ...........uuggcacuuguagaauucacuga....................................................    284        0         seq
    ...........uuggcacuuguagaauucacugaua..................................................    1          0         seq
    ............uggcacuuguagaauuca........................................................    1          0         seq
    ............uggcacuuguagaauucac.......................................................    2          0         seq
    ............uggcacuuguagaauucacu......................................................    11         0         seq
    ............uggcacuuguagaauucacug.....................................................    13         0         seq
    ............uggcacuuguagaauucacuga....................................................    226        0         seq
    ............uggcacuuguagaauucacugau...................................................    3          0         seq
    ............uggcacuuguagaauucacugaua..................................................    9          0         seq
    ............uggcacuuguagaauucacugauacu................................................    1          0         seq
    .............ggcacuuguagaauucacu......................................................    1          0         seq
    .............ggcacuuguagaauucacug.....................................................    1          0         seq
    .............ggcacuuguagaauucacuga....................................................    14         0         seq
```

```
                                                    cgi-miR-182-3p
          cgi-miR-182-5p
gcgugcagaucuuggcacuuguagaauucacugauacuugaauauuuaaucucagugaaucuauaguggcaaccgcggcacgcua

..............gcacuuguagaauucacuga...................................................    1      0      seq
...............cacuuguagaauucacuga...................................................    2      0      seq
................acuuguagaauucacuga...................................................    3      0      seq
..................................uacuugaauauuuaaucuc................................    15     0      seq
...................................................ucagugaaucuauaguggca..............    1      0      seq
...................................................ucagugaaucuauaguggcaa.............    2      0      seq
...................................................ucagugaaucuauaguggcaac............    7      0      seq
...................................................ucagugaaucuauaguggcaacc...........    5      0      seq
...................................................ucagugaaucuauaguggcaaccg..........    5      0      seq
....................................................cagugaaucuauaguggca..............    1      0      seq
....................................................cagugaaucuauaguggcaa.............    4      0      seq
....................................................cagugaaucuauaguggcaac............    1      0      seq
....................................................cagugaaucuauaguggcaaccg..........    45     0      seq
.....................................................agugaaucuauaguggca..............    42     0      seq
.....................................................agugaaucuauaguggcaa.............    938    0      seq
.....................................................agugaaucuauaguggcaac............    507    0      seq
.....................................................agugaaucuauaguggcaacc...........    712    0      seq
.....................................................agugaaucuauaguggcaaccg..........    9224   0      seq
.....................................................agugaaucuauaguggcaaccgc.........    2      0      seq
......................................................gugaaucuauaguggcaa.............    9      0      seq
......................................................gugaaucuauaguggcaac............    12     0      seq
......................................................gugaaucuauaguggcaacc...........    17     0      seq
......................................................gugaaucuauaguggcaaccg..........    95     0      seq
.......................................................ugaaucuauaguggcaac............    1      0      seq
.......................................................ugaaucuauaguggcaacc...........    1      0      seq
.......................................................ugaaucuauaguggcaaccg..........    38     0      seq
........................................................gaaucuauaguggcaaccg..........    1      0      seq
```
