# Supplementary material for: Identification of Conserved and Novel MicroRNAs in the Pacific Oyster Crassostrea gigas by Deep Sequencing
Source: PLoS One. 2014 Aug 19;9(8):e104371. doi: 10.1371/journal.pone.0104371 (PMC4138081; doi:10.1371/journal.pone.0104371)
Supplement: File S2 — The compressed/ZIP file archive for the predicted precursors' secondary structures and reads alignment. (ZIP) [file pone.0104371.s010.zip › second structure and reads alignment for oyster miRNAs/conserved in table S4/cgi-miR-183.pdf]

miRBase precursor : cgi-miR-183
Total read count : 14744239
cgi-miR-183-5p read count : 14743535
cgi-miR-183-3p read count : 698
remaining reads : 6

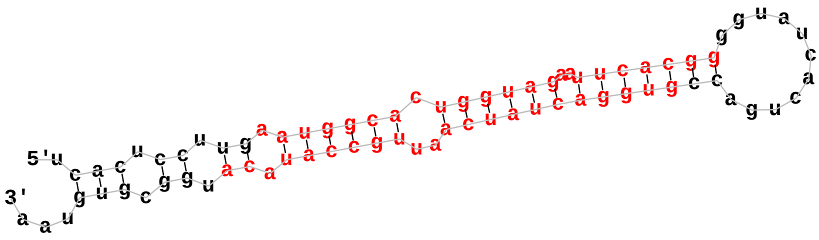

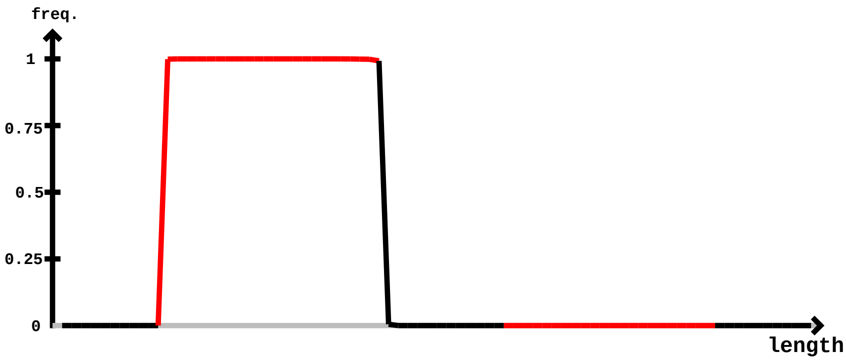


```
                cgi-miR-183-5p
                                           cgi-miR-183-3p
5'- ucacuccuugaauggcacugguagaauucacgggguaucacugaccguggacuaucaauugccauacauggcguguaa   -3'    exp
    .(((.((.((.((((((.((((((..(((((((...........)))))))))))))..)))))).)).)).)))...    reads      mm          sample
    .........gaauggcacugguagaauuca................................................    1          0           seq
    .........gaauggcacugguagaauucacg..............................................    1          0           seq
    .........gaauggcacugguagaauucacgg.............................................    54         0           seq
    ..........aauggcacugguagaauu..................................................    488        0           seq
    ..........aauggcacugguagaauuc.................................................    2161       0           seq
    ..........aauggcacugguagaauuca................................................    9916       0           seq
    ..........aauggcacugguagaauucac...............................................    8014       0           seq
    ..........aauggcacugguagaauucacg..............................................    89120      0           seq
    ..........aauggcacugguagaauucacgg.............................................    14544978   0           seq
    ..........aauggcacugguagaauucacggg............................................    73609      0           seq
    ..........aauggcacugguagaauucacgggg...........................................    58         0           seq
    ..........aauggcacugguagaauucacggggu..........................................    15         0           seq
    ..........aauggcacugguagaauucacgggguа.........................................    22         0           seq
    ..........aauggcacugguagaauucacgggguau........................................    9          0           seq
    ...........auggcacugguagaauuc.................................................    3          0           seq
    ...........auggcacugguagaauuca................................................    15         0           seq
    ...........auggcacugguagaauucac...............................................    11         0           seq
    ...........auggcacugguagaauucacg..............................................    99         0           seq
    ...........auggcacugguagaauucacgg.............................................    14124      0           seq
    ...........auggcacugguagaauucacggg............................................    86         0           seq
    ...........auggcacugguagaauucacgggguа.........................................    1          0           seq
    ............uggcacugguagaauucacg..............................................    4          0           seq
    ............uggcacugguagaauucacgg.............................................    407        0           seq
    ............uggcacugguagaauucacggg............................................    5          0           seq
    .............ggcacugguagaauucacgg.............................................    81         0           seq
    .............ggcacugguagaauucacggg............................................    1          0           seq
    ..............gcacugguagaauucacg..............................................    1          0           seq
    ..............gcacugguagaauucacgg.............................................    147        0           seq
    ..............gcacugguagaauucacggg............................................    3          0           seq
    ..............gcacugguagaauucacgggguauc.......................................    1          0           seq
    ...............cacugguagaauucacgg.............................................    100        0           seq
    ..................ugguagaauucacggggu..........................................    1          0           seq
    ...............................gggguaucacugaccguggacuauca.....................    1          0           seq
    ................................ggguaucacugaccgugg............................    1          0           seq
```

```
          cgi-miR-183-5p
                                              cgi-miR-183-3p
ucacuccuugaauggcacugguagaauucacgggguaucacugaccguggacuaucaauugccauacauggcguguaa

....................................aucacugaccguggacuaucaauu..................    1      0      seq
......................................cacugaccguggacuaucaauu..................    1      0      seq
........................................cugaccguggacuaucaauugc................    1      0      seq
.............................................cguggacuaucaauugccauac...........    1      0      seq
.............................................cguggacuaucaauugccauaca..........    8      0      seq
..............................................guggacuaucaauugcca..............    43     0      seq
..............................................guggacuaucaauugccau.............    6      0      seq
..............................................guggacuaucaauugccaua............    30     0      seq
..............................................guggacuaucaauugccauac...........    63     0      seq
..............................................guggacuaucaauugccauaca..........    535    0      seq
..............................................guggacuaucaauugccauacau........    2      0      seq
...............................................uggacuaucaauugccaua............    2      0      seq
...............................................uggacuaucaauugccauac...........    3      0      seq
...............................................uggacuaucaauugccauaca..........    5      0      seq
```
