# Supplementary material for: Identification of Conserved and Novel MicroRNAs in the Pacific Oyster Crassostrea gigas by Deep Sequencing
Source: PLoS One. 2014 Aug 19;9(8):e104371. doi: 10.1371/journal.pone.0104371 (PMC4138081; doi:10.1371/journal.pone.0104371)
Supplement: File S2 — The compressed/ZIP file archive for the predicted precursors' secondary structures and reads alignment. (ZIP) [file pone.0104371.s010.zip › second structure and reads alignment for oyster miRNAs/conserved in table S4/cgi-miR-190.pdf]

miRBase precursor : cgi-miR-190
Total read count : 43200
cgi-miR-190-5p read count : 42496
cgi-miR-190-3p read count : 701
remaining reads : 3

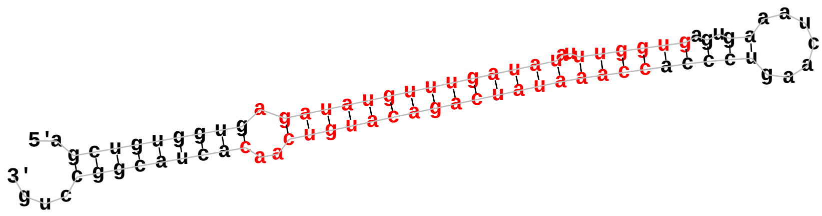

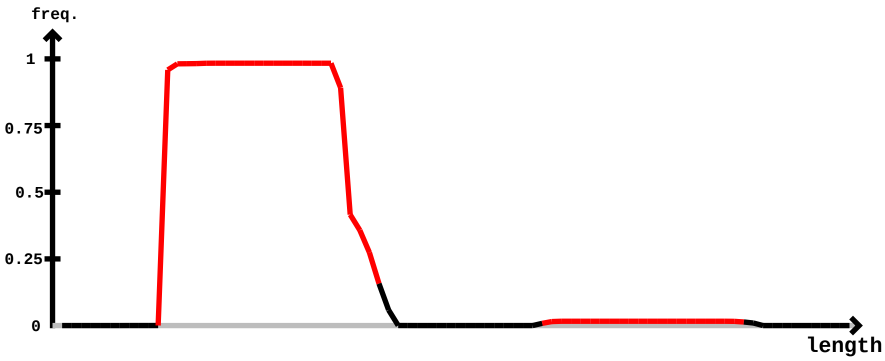


```
                cgi-miR-190-5p
                                      cgi-miR-190-3p
5'- agcuguggugagauauguuugauauauuuggugagugaaaucaagucccaccaaauaucagacaugucaacacuacggccug   -3'    exp
    .(((((((((.((((((((((((((..((((((.(.((.......)))))))))))))))))))))))..)))))))))...   reads      mm         sample
    .....uggugagauauguuugauau.........................................................   1          0          seq
    ......ggugagauauguuugauau.........................................................   1          0          seq
    ......ggugagauauguuugauauauuu.....................................................   1          0          seq
    ........ugagauauguuugauauauuugg...................................................   1          0          seq
    .........gagauauguuugauauau.......................................................   1          0          seq
    .........gagauauguuugauauauu......................................................   1          0          seq
    .........gagauauguuugauauauuugg...................................................   2          0          seq
    ..........agauauguuugauauauu......................................................   3973       0          seq
    ..........agauauguuugauauauuu.....................................................   20389      0          seq
    ..........agauauguuugauauauuug....................................................   2270       0          seq
    ..........agauauguuugauauauuugg...................................................   3448       0          seq
    ..........agauauguuugauauauuuggu..................................................   4814       0          seq
    ..........agauauguuugauauauuuggug.................................................   4038       0          seq
    ..........agauauguuugauauauuuggugа................................................   2467       0          seq
    ..........agauauguuugauauauuuggugag...............................................   3          0          seq
    ..........agauauguuugauauauuuggugagu..............................................   2          0          seq
    ..........agauauguuugauauauuuggugaguga............................................   1          0          seq
    ...........gauauguuugauauauuu.....................................................   160        0          seq
    ...........gauauguuugauauauuug....................................................   245        0          seq
    ...........gauauguuugauauauuugg...................................................   156        0          seq
    ...........gauauguuugauauauuuggu..................................................   181        0          seq
    ...........gauauguuugauauauuuggug.................................................   209        0          seq
    ...........gauauguuugauauauuuggugа................................................   33         0          seq
    ............auauguuugauauauuug....................................................   4          0          seq
    ............auauguuugauauauuuggu..................................................   3          0          seq
    ............auauguuugauauauuuggug.................................................   3          0          seq
    ............auauguuugauauauuuggugа................................................   4          0          seq
    ............auauguuugauauauuuggugag...............................................   2          0          seq
    .............uauguuugauauauuugg...................................................   9          0          seq
    .............uauguuugauauauuuggu..................................................   6          0          seq
    .............uauguuugauauauuuggug.................................................   1          0          seq
    .............uauguuugauauauuuggugа................................................   6          0          seq
    ..............auguuugauauauuuggu..................................................   42         0          seq
    ..............auguuugauauauuuggug.................................................   10         0          seq
```

```
          cgi-miR-190-5p
                                                    cgi-miR-190-3p
agcugugguggagauauguuugauauauuuggugagugaaaucaagucccaccaaauaucagacaugucaacacuacggccug

..............auguuugauauauuuggugа.................................................    1      0      seq
..............auguuugauauauuuggugag................................................    2      0      seq
...............uguuugauauauuuggug..................................................    6      0      seq
...............uguuugauauauuuggugа.................................................    2      0      seq
................guuugauauauuuggugа.................................................    1      0      seq
..................................................accaaauaucagacaugu...............    1      0      seq
..................................................accaaauaucagacauguca.............    12     0      seq
..................................................accaaauaucagacaugucaa............    59     0      seq
..................................................accaaauaucagacaugucaac...........    77     0      seq
..................................................accaaauaucagacaugucaaca..........    209    0      seq
...................................................ccaaauaucagacauguca.............    4      0      seq
...................................................ccaaauaucagacaugucaa............    34     0      seq
...................................................ccaaauaucagacaugucaac...........    83     0      seq
...................................................ccaaauaucagacaugucaaca..........    171    0      seq
....................................................caaauaucagacaugucaa............    9      0      seq
....................................................caaauaucagacaugucaac...........    11     0      seq
....................................................caaauaucagacaugucaaca..........    28     0      seq
.....................................................aaauaucagacaugucaa............    2      0      seq
.....................................................aaauaucagacaugucaaca..........    1      0      seq
```
