# Supplementary material for: Identification of Conserved and Novel MicroRNAs in the Pacific Oyster Crassostrea gigas by Deep Sequencing
Source: PLoS One. 2014 Aug 19;9(8):e104371. doi: 10.1371/journal.pone.0104371 (PMC4138081; doi:10.1371/journal.pone.0104371)
Supplement: File S2 — The compressed/ZIP file archive for the predicted precursors' secondary structures and reads alignment. (ZIP) [file pone.0104371.s010.zip › second structure and reads alignment for oyster miRNAs/conserved in table S4/cgi-miR-193.pdf]

miRBase precursor : cgi-miR-193
Total read count : 333636
cgi-miR-193-5p read count : 331
cgi-miR-193-3p read count : 333292
remaining reads : 13

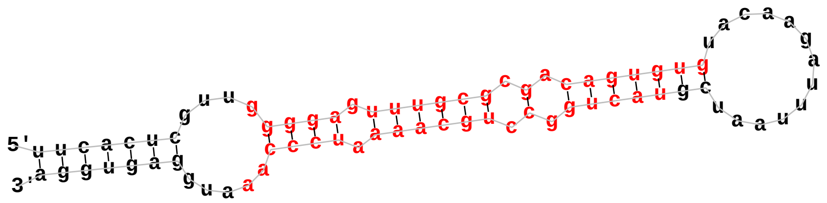

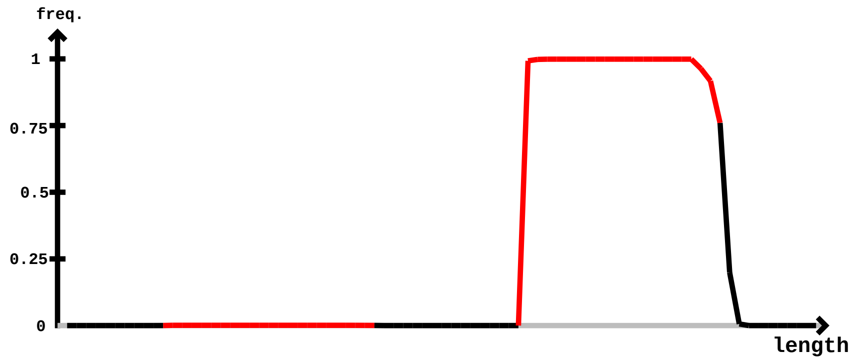


```
                                    cgi-miR-193-5p
                                                      cgi-miR-193-3p
5'- uucacucguuggggaguuugcgcgacagugugguacaagauuuaaaucguacuggccugcaaaaucccaaauggagugga   -3'    exp
    (((((((....((((.((((((.(.(((((((.............))))))).).)))))).)))).....)))))))    reads      mm        sample
    .........uggggaguuugcgcgacagu..................................................    2          0         seq
    .........uggggaguuugcgcgacagug.................................................    3          0         seq
    .........uggggaguuugcgcgacagugu................................................    33         0         seq
    .........uggggaguuugcgcgacagugug...............................................    6          0         seq
    ..........ggggaguuugcgcgacag...................................................    6          0         seq
    ..........ggggaguuugcgcgacagu..................................................    31         0         seq
    ..........ggggaguuugcgcgacagug.................................................    38         0         seq
    ..........ggggaguuugcgcgacagugu................................................    37         0         seq
    ..........ggggaguuugcgcgacagugug...............................................    158        0         seq
    ..........ggggaguuugcgcgacagugugu..............................................    4          0         seq
    ..........ggggaguuugcgcgacaguguguacaa..........................................    1          0         seq
    ...........gggaguuugcgcgacagu..................................................    2          0         seq
    ...........gggaguuugcgcgacagugu................................................    2          0         seq
    ...........gggaguuugcgcgacagugug...............................................    7          0         seq
    ...........gggaguuugcgcgacagugugu..............................................    1          0         seq
    ................................uacaagauuuaaaucguacuggccu......................    7          0         seq
    ...................................aagauuuaaaucguacuggccugcaa..................    1          0         seq
    ........................................uuaaaucguacuggccugcaaaaucccaa..........    1          0         seq
    .........................................uaaaucguacuggccugcaaaauccca...........    1          0         seq
    .........................................uaaaucguacuggccugcaaaaucccaa..........    1          0         seq
    ............................................aucguacuggccugcaaaaucccaa..........    1          0         seq
    .............................................ucguacuggccugcaaaauccca...........    1          0         seq
    ................................................uacuggccugcaaaaucc.............    11802      0         seq
    ................................................uacuggccugcaaaauccc............    15455      0         seq
    ................................................uacuggccugcaaaauccca...........    52174      0         seq
    ................................................uacuggccugcaaaaucccaa..........    185785     0         seq
    ................................................uacuggccugcaaaaucccaaa.........    64008      0         seq
    ................................................uacuggccugcaaaaucccaaau........    1975       0         seq
    ................................................uacuggccugcaaaaucccaaaug.......    1          0         seq
    ................................................uacuggccugcaaaaucccaaaugga.....    1          0         seq
    .................................................acuggccugcaaaauccc............    45         0         seq
    .................................................acuggccugcaaaauccca...........    300        0         seq
    .................................................acuggccugcaaaaucccaa..........    1145       0         seq
    .................................................acuggccugcaaaaucccaaa.........    206        0         seq
```

```
          cgi-miR-193-5p
                                              cgi-miR-193-3p
uucacucguuggggaguuugcgcgacaguguguacaagauuuaaucguacuggccugcaaaaucccaaauggagugga

................................................acuggccugcaaaaucccaaau........   68     0     seq
.................................................cuggccugcaaaauccca...........   47     0     seq
.................................................cuggccugcaaaaucccaa..........   208    0     seq
.................................................cuggccugcaaaaucccaaa.........   43     0     seq
.................................................cuggccugcaaaaucccaaau........   2      0     seq
..................................................uggccugcaaaaucccaa..........   19     0     seq
..................................................uggccugcaaaaucccaaa.........   6      0     seq
...................................................ggccugcaaaaucccaaa.........   2      0     seq
```
