# Supplementary material for: Identification of Conserved and Novel MicroRNAs in the Pacific Oyster Crassostrea gigas by Deep Sequencing
Source: PLoS One. 2014 Aug 19;9(8):e104371. doi: 10.1371/journal.pone.0104371 (PMC4138081; doi:10.1371/journal.pone.0104371)
Supplement: File S2 — The compressed/ZIP file archive for the predicted precursors' secondary structures and reads alignment. (ZIP) [file pone.0104371.s010.zip › second structure and reads alignment for oyster miRNAs/conserved in table S4/cgi-miR-1984.pdf]

miRBase precursor : cgi-miR-1984
Total read count : 422137
cgi-miR-1984-5p read count: 411806
cgi-miR-1984-3p read count: 10323
remaining reads : 8

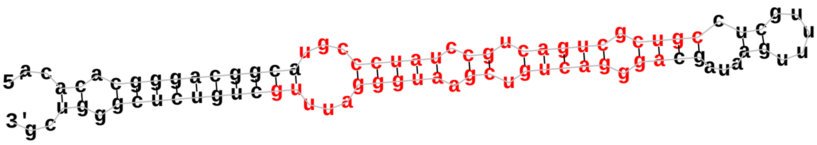

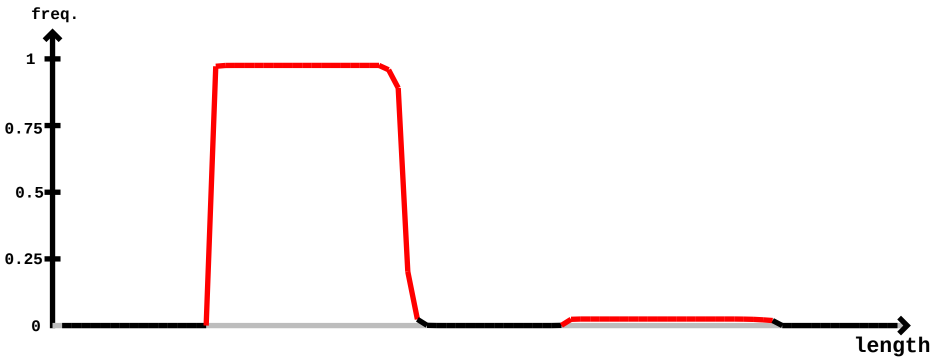


```
                                cgi-miR-1984-5p
                                                                    cgi-miR-1984-3p
5'- acacacgggacggcaugcccuauccgucagucgcugccucguuuugaauagcagggacugucgaaugggauuugcugucucgggucg -3'   exp
    ..((.((((((((((...(((((.((.(((((.((((.((.....))...)))).))))).)).)))))...)))))))))).))..    reads   mm   sample
    .............caugcccuauccgucagucgcu....................................................    1       0    seq
    ..............augcccuauccgucagucgc.....................................................    1       0    seq
    ..............augcccuauccgucagucgcu....................................................    7       0    seq
    ..............augcccuauccgucagucgcug...................................................    1       0    seq
    ..............augcccuauccgucagucgcugc..................................................    1       0    seq
    ...............ugcccuauccgucagucg......................................................    6954    0    seq
    ...............ugcccuauccgucagucgc.....................................................    28700   0    seq
    ...............ugcccuauccgucagucgcu....................................................    290122  0    seq
    ...............ugcccuauccgucagucgcug...................................................    74836   0    seq
    ...............ugcccuauccgucagucgcugc..................................................    9410    0    seq
    ...............ugcccuauccgucagucgcugcc.................................................    320     0    seq
    ...............ugcccuauccgucagucgcugccu................................................    34      0    seq
    ................gcccuauccgucagucgc.....................................................    56      0    seq
    ................gcccuauccgucagucgcu....................................................    643     0    seq
    ................gcccuauccgucagucgcug...................................................    498     0    seq
    ................gcccuauccgucagucgcugc..................................................    118     0    seq
    ................gcccuauccgucagucgcugcc.................................................    7       0    seq
    .................cccuauccgucagucgcu....................................................    63      0    seq
    .................cccuauccgucagucgcug...................................................    15      0    seq
    .................cccuauccgucagucgcugc..................................................    3       0    seq
    ..................ccuauccgucagucgcug...................................................    13      0    seq
    ..................ccuauccgucagucgcugc..................................................    2       0    seq
    .....................auccgucagucgcugccu................................................    1       0    seq
    ............................................ugaauagcagggacugucgaauggg..................    1       0    seq
    ............................................ugaauagcagggacugucgaaugggau................    1       0    seq
    ............................................ugaauagcagggacugucgaaugggauu...............    2       0    seq
    .............................................gaauagcagggacugucgaauggg..................    1       0    seq
    .............................................gaauagcagggacugucgaaugggauu...............    1       0    seq
    .................................................agcagggacugucgaaugggau................    1       0    seq
    .................................................agcagggacugucgaaugggauu...............    1       0    seq
    ...................................................cagggacugucgaauggga.................    4       0    seq
    ...................................................cagggacugucgaaugggau................    16      0    seq
    ...................................................cagggacugucgaaugggauu...............    51      0    seq
    ...................................................cagggacugucgaaugggauuu..............    58      0    seq
```

```
               cgi-miR-1984-5p
                                                    cgi-miR-1984-3p
acacacgggacggcaugcccuauccgucagucgcugccucguuuugaauagcagggacugucgaaugggauuugcugucucgggucg

...................................................cagggacugucgaaugggauuug.............    192     0     seq
....................................................agggacugucgaauggga.................    133     0     seq
....................................................agggacugucgaaugggau................    314     0     seq
....................................................agggacugucgaaugggauu...............    714     0     seq
....................................................agggacugucgaaugggauuu..............    1021    0     seq
....................................................agggacugucgaaugggauuug.............    7356    0     seq
....................................................agggacugucgaaugggauuugc............    1       0     seq
.....................................................gggacugucgaaugggau................    10      0     seq
.....................................................gggacugucgaaugggauu...............    27      0     seq
.....................................................gggacugucgaaugggauuu..............    27      0     seq
.....................................................gggacugucgaaugggauuug.............    388     0     seq
.....................................................gggacugucgaaugggauuugc............    11      0     seq
```
