# Supplementary material for: Identification of Conserved and Novel MicroRNAs in the Pacific Oyster Crassostrea gigas by Deep Sequencing
Source: PLoS One. 2014 Aug 19;9(8):e104371. doi: 10.1371/journal.pone.0104371 (PMC4138081; doi:10.1371/journal.pone.0104371)
Supplement: File S2 — The compressed/ZIP file archive for the predicted precursors' secondary structures and reads alignment. (ZIP) [file pone.0104371.s010.zip › second structure and reads alignment for oyster miRNAs/conserved in table S4/cgi-miR-1985.pdf]

miRBase precursor : cgi-miR-1985
Total read count : 2872045
cgi-miR-1985-5p read count: 2322123
cgi-miR-1985-3p read count: 549821
remaining reads : 101

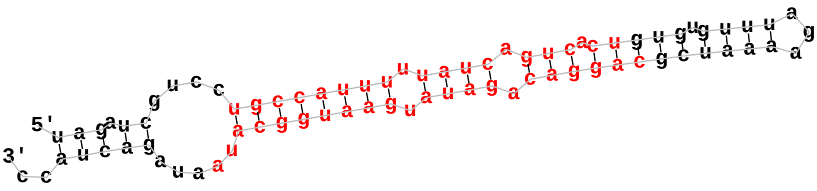

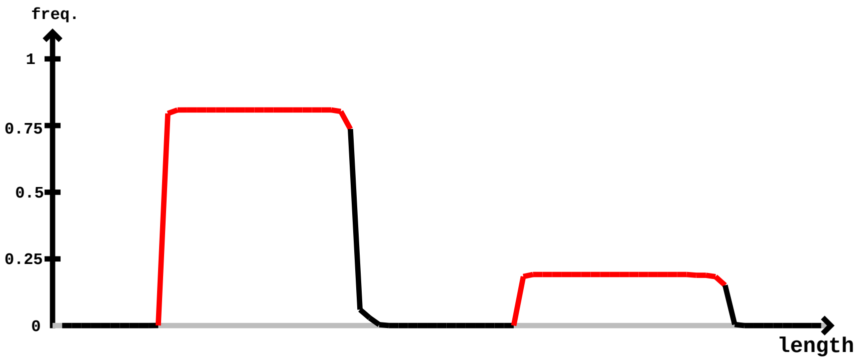


```
                                                                   cgi-miR-1985-3p
                  cgi-miR-1985-5p
5'- uagaucguccugccauuuuuaucagucacugugugguuuagaaaaucgcaggacagauaugaauggcauaauagacuacc   -3'    exp
    (((.((....((((((((.((((.(((.(((((.((((...)))))))))))).)))).)))))))).....)))))..    reads      mm         sample
    .........cugccauuuuuaucaguc....................................................    10         0          seq
    .........cugccauuuuuaucaguca...................................................    13         0          seq
    .........cugccauuuuuaucagucac..................................................    156        0          seq
    .........cugccauuuuuaucagucacu.................................................    818        0          seq
    .........cugccauuuuuaucagucacug................................................    75         0          seq
    .........cugccauuuuuaucagucacugu...............................................    36         0          seq
    .........cugccauuuuuaucagucacugug..............................................    3          0          seq
    ..........ugccauuuuuaucaguca...................................................    16392      0          seq
    ..........ugccauuuuuaucagucac..................................................    185444     0          seq
    ..........ugccauuuuuaucagucacu.................................................    1911568    0          seq
    ..........ugccauuuuuaucagucacug................................................    87831      0          seq
    ..........ugccauuuuuaucagucacugu...............................................    72295      0          seq
    ..........ugccauuuuuaucagucacugug..............................................    9490       0          seq
    ..........ugccauuuuuaucagucacugugu.............................................    320        0          seq
    ..........ugccauuuuuaucagucacugugug............................................    2          0          seq
    ...........gccauuuuuaucagucac..................................................    3781       0          seq
    ...........gccauuuuuaucagucacu.................................................    31659      0          seq
    ...........gccauuuuuaucagucacug................................................    1056       0          seq
    ...........gccauuuuuaucagucacugu...............................................    584        0          seq
    ...........gccauuuuuaucagucacugug..............................................    52         0          seq
    ...........gccauuuuuaucagucacugugu.............................................    2          0          seq
    ............ccauuuuuaucagucacu.................................................    428        0          seq
    ............ccauuuuuaucagucacug................................................    19         0          seq
    ............ccauuuuuaucagucacugu...............................................    17         0          seq
    ............ccauuuuuaucagucacugug..............................................    2          0          seq
    .............cauuuuuaucagucacug................................................    29         0          seq
    .............cauuuuuaucagucacugu...............................................    22         0          seq
    .............cauuuuuaucagucacugug..............................................    5          0          seq
    ..............auuuuuaucagucacugu...............................................    11         0          seq
    ..............auuuuuaucagucacugug..............................................    1          0          seq
    ...............uuuuuaucagucacugug..............................................    2          0          seq
    .................................uguuuagaaaaucgcagga...........................    1          0          seq
    .................................uguuuagaaaaucgcaggaca.........................    1          0          seq
    .................................uguuuagaaaaucgcaggacaga.......................    1          0          seq
```

```
                                               cgi-miR-1985-3p
          cgi-miR-1985-5p
uagaucguccugccauuuuuaucagucacugugugiuuagaaaaucgcaggacagauaugaauggcauaauagacuacc

.................................uguuuagaaaaucgcaggacagau......................    3          0          seq
.................................uguuuagaaaaucgcaggacagaua.....................    2          0          seq
.................................uguuuagaaaaucgcaggacagauau....................    3          0          seq
.................................uguuuagaaaaucgcaggacagauauga..................    2          0          seq
...................................uuuagaaaaucgcaggacag........................    1          0          seq
....................................uuagaaaaucgcaggacagau......................    1          0          seq
.........................................aaaucgcaggacagauaugaauggcau...........    1          0          seq
..........................................aaucgcaggacagauaugaauggcau...........    3          0          seq
...........................................aucgcaggacagauaugaauggca............    2          0          seq
...........................................aucgcaggacagauaugaauggcau...........    3          0          seq
...........................................aucgcaggacagauaugaauggcaua..........    4          0          seq
............................................ucgcaggacagauaugaa.................    2          0          seq
............................................ucgcaggacagauaugaau................    1          0          seq
............................................ucgcaggacagauaugaaug...............    2          0          seq
............................................ucgcaggacagauaugaaugg..............    28         0          seq
............................................ucgcaggacagauaugaauggc.............    24         0          seq
............................................ucgcaggacagauaugaauggca............    10         0          seq
............................................ucgcaggacagauaugaauggcau...........    3          0          seq
............................................ucgcaggacagauaugaauggcaua..........    3          0          seq
.............................................cgcaggacagauaugaaug...............    1          0          seq
.............................................cgcaggacagauaugaauggcaua..........    3          0          seq
..............................................gcaggacagauaugaauggca............    1          0          seq
..............................................gcaggacagauaugaauggcau...........    3          0          seq
..............................................gcaggacagauaugaauggcaua..........    6          0          seq
...............................................caggacagauaugaaugg..............    8335       0          seq
...............................................caggacagauaugaauggc.............    168        0          seq
...............................................caggacagauaugaauggca............    14412      0          seq
...............................................caggacagauaugaauggcau...........    89404      0          seq
...............................................caggacagauaugaauggcaua..........    411691     0          seq
...............................................caggacagauaugaauggcauaa.........    4110       0          seq
...............................................caggacagauaugaauggcauaau........    8          0          seq
................................................aggacagauaugaauggc.............    2          0          seq
................................................aggacagauaugaauggca............    517        0          seq
................................................aggacagauaugaauggcau...........    2881       0          seq
................................................aggacagauaugaauggcaua..........    10536      0          seq
................................................aggacagauaugaauggcauaa.........    7681       0          seq
................................................aggacagauaugaauggcauaau........    17         0          seq
.................................................ggacagauaugaauggcau...........    7          0          seq
.................................................ggacagauaugaauggcaua..........    22         0          seq
.................................................ggacagauaugaauggcauaa.........    15         0          seq
..................................................gacagauaugaauggcauaa.........    1          0          seq
```
