# Supplementary material for: Identification of Conserved and Novel MicroRNAs in the Pacific Oyster Crassostrea gigas by Deep Sequencing
Source: PLoS One. 2014 Aug 19;9(8):e104371. doi: 10.1371/journal.pone.0104371 (PMC4138081; doi:10.1371/journal.pone.0104371)
Supplement: File S2 — The compressed/ZIP file archive for the predicted precursors' secondary structures and reads alignment. (ZIP) [file pone.0104371.s010.zip › second structure and reads alignment for oyster miRNAs/conserved in table S4/cgi-miR-1986.pdf]

miRBase precursor : cgi-miR-1986
Total read count : 18064
cgi-miR-1986-5p read count : 3354
cgi-miR-1986-3p read count : 14709
remaining reads : 1

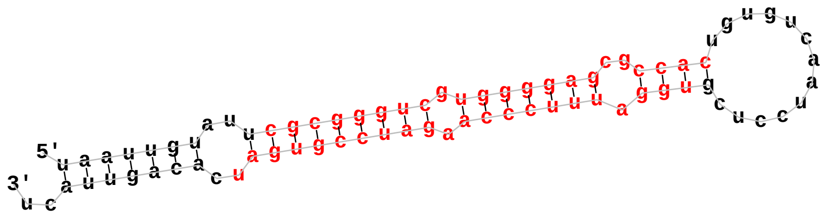

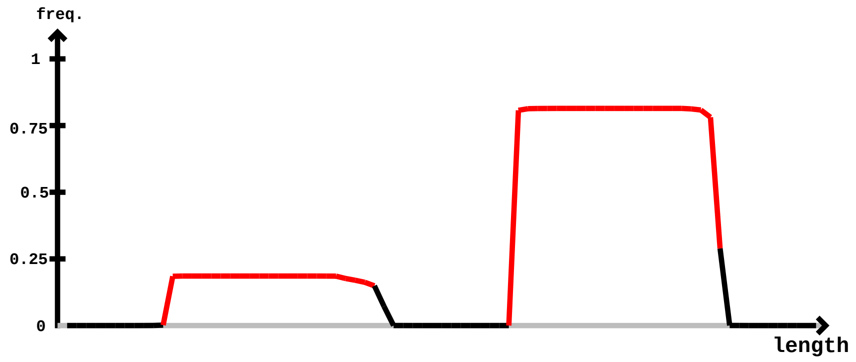

cgi-miR-1986-3p
cgi-miR-1986-5p

5'- uaauuguauucgcgggucguggggagcgccacugugucaauccucguggauuucccaagauccgugaucacaguuacu -3'	exp		
(((((((..(((((((((.(((((((..((((.............)))).))))))).)))))))))..))))))))..	reads	mm	sample
.......auucgcgggucguggggag..	1	0	seq
........uucgcgggucguggggag..	3	0	seq
........uucgcgggucguggggagcg..	1	0	seq
.........ucgcgggucguggggagc...	4	0	seq
.........ucgcgggucguggggagcg..	4	0	seq
.........ucgcgggucguggggagcgc...	4	0	seq
.........ucgcgggucguggggagcgcc..	2	0	seq
.........ucgcgggucguggggagcgcca...	3	0	seq
.........ucgcgggucguggggagcgccac..	6	0	seq
.........ucgcgggucguggggagcgccacu...	2	0	seq
..........cgcgggucguggggagcg..	154	0	seq
..........cgcgggucguggggagcgc...	115	0	seq
..........cgcgggucguggggagcgcc..	137	0	seq
..........cgcgggucguggggagcgcca...	232	0	seq
..........cgcgggucguggggagcgccac..	1381	0	seq
..........cgcgggucguggggagcgccacu...	1287	0	seq
..........cgcgggucguggggagcgccacug..	1	0	seq
..........cgcgggucguggggagcgccacugu...	1	0	seq
...........gcgggucguggggagcgcca...	1	0	seq
...........gcgggucguggggagcgccac..	4	0	seq
...........gcgggucguggggagcgccacu...	12	0	seq
....uggauuucccaagauccg..............	33	0	seq
....uggauuucccaagauccgu.............	73	0	seq
....uggauuucccaagauccgug............	484	0	seq
....uggauuucccaagauccguga...........	8818	0	seq
....uggauuucccaagauccgugau..........	5167	0	seq
....uggauuucccaagauccgugauc.........	2	0	seq
....uggauuucccaagauccgugauca........	2	0	seq
....ggauuucccaagauccgu.............	1	0	seq
....ggauuucccaagauccgug............	8	0	seq
....ggauuucccaagauccguga...........	67	0	seq
....ggauuucccaagauccgugau..........	35	0	seq
....gauuucccaagauccguga...........	5	0	seq
....gauuucccaagauccgugau..........	6	0	seq

```
                                              cgi-miR-1986-3p
          cgi-miR-1986-5p
uaauuguauucgcgggucguggggagcgccacugugucaauccucguggauuucccaagauccgugaucacaguuacu

................................................gauuucccaagauccgugauca........    1    0    seq
.................................................auuucccaagauccgugau..........    4    0    seq
..................................................uuucccaagauccgugau..........    3    0    seq
```
