# Supplementary material for: Identification of Conserved and Novel MicroRNAs in the Pacific Oyster Crassostrea gigas by Deep Sequencing
Source: PLoS One. 2014 Aug 19;9(8):e104371. doi: 10.1371/journal.pone.0104371 (PMC4138081; doi:10.1371/journal.pone.0104371)
Supplement: File S2 — The compressed/ZIP file archive for the predicted precursors' secondary structures and reads alignment. (ZIP) [file pone.0104371.s010.zip › second structure and reads alignment for oyster miRNAs/conserved in table S4/cgi-miR-1989.pdf]

miRBase precursor : cgi-miR-1989
Total read count : 7313
cgi-miR-1989-5p read count: 6726
cgi-miR-1989-3p read count: 587
remaining reads : 0

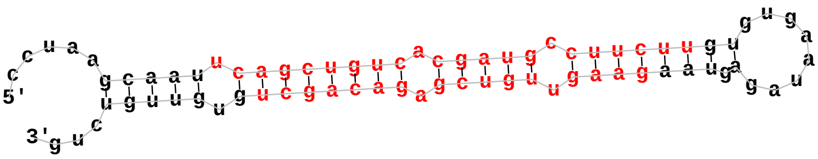

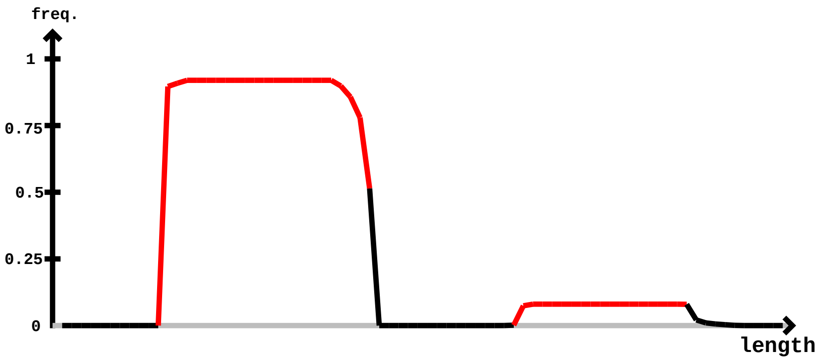


```
               cgi-miR-1989-5p
                                             cgi-miR-1989-3p
5'- ccuaagcaauucagcugucacgaugccuucuugugugaauagaguaagaaguugucgagacagcuguguugucug   -3'    exp
    .....(((((.((((((((.(((((.((((((((........).))))))).))))).)))))))).))))).. .   reads   mm   sample
    ..........ucagcugucacgaugccu...............................................    156     0    seq
    ..........ucagcugucacgaugccuu..............................................    288     0    seq
    ..........ucagcugucacgaugccuuc.............................................    549     0    seq
    ..........ucagcugucacgaugccuucu............................................    1898    0    seq
    ..........ucagcugucacgaugccuucuu...........................................    3665    0    seq
    ...........cagcugucacgaugccuu..............................................    13      0    seq
    ...........cagcugucacgaugccuuc.............................................    6       0    seq
    ...........cagcugucacgaugccuucu............................................    27      0    seq
    ...........cagcugucacgaugccuucuu...........................................    44      0    seq
    ............agcugucacgaugccuuc.............................................    10      0    seq
    ............agcugucacgaugccuucu............................................    20      0    seq
    ............agcugucacgaugccuucuu...........................................    50      0    seq
    .............................................aagaaguugucgagacagcu..........    1       0    seq
    ..............................................agaaguugucgagacagc...........    4       0    seq
    ..............................................agaaguugucgagacagcu..........    6       0    seq
    ...............................................gaaguugucgagacagcu..........    422     0    seq
    ...............................................gaaguugucgagacagcug.........    65      0    seq
    ...............................................gaaguugucgagacagcugu........    20      0    seq
    ...............................................gaaguugucgagacagcugug.......    8       0    seq
    ...............................................gaaguugucgagacagcugugu......    12      0    seq
    ...............................................gaaguugucgagacagcuguguu.....    4       0    seq
    ................................................aaguugucgagacagcug.........    16      0    seq
    ................................................aaguugucgagacagcugu........    10      0    seq
    ................................................aaguugucgagacagcugug.......    11      0    seq
    ................................................aaguugucgagacagcugugu......    6       0    seq
    ................................................aaguugucgagacagcuguguu.....    2       0    seq
```
