# Supplementary material for: Identification of Conserved and Novel MicroRNAs in the Pacific Oyster Crassostrea gigas by Deep Sequencing
Source: PLoS One. 2014 Aug 19;9(8):e104371. doi: 10.1371/journal.pone.0104371 (PMC4138081; doi:10.1371/journal.pone.0104371)
Supplement: File S2 — The compressed/ZIP file archive for the predicted precursors' secondary structures and reads alignment. (ZIP) [file pone.0104371.s010.zip › second structure and reads alignment for oyster miRNAs/conserved in table S4/cgi-miR-1990.pdf]

miRBase precursor : cgi-miR-1990
Total read count : 2438525
cgi-miR-1990-5p read count: 21264
cgi-miR-1990-3p read count: 2417220
remaining reads : 41

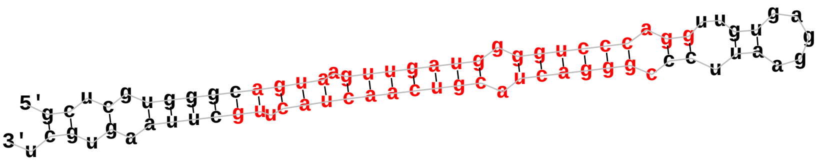

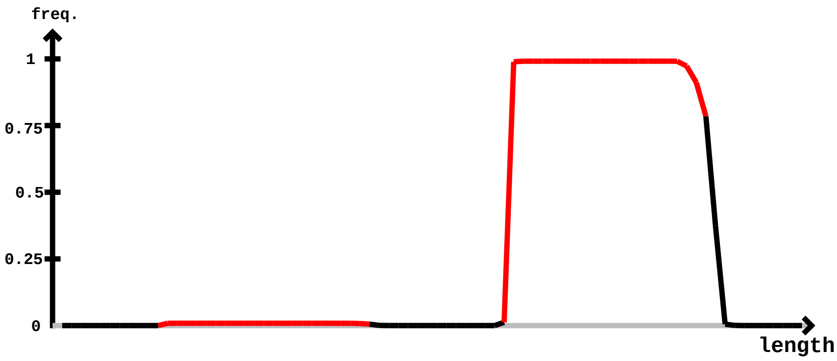

cgi-miR-1990-5p

cgi-miR-1990-3p

```
5'- gcucgugggcaguaaguugauggggucccagguugugaggaauucccgggacuacgucaacuacuugcuuaagugcu   -3'    exp
    ((.(.(((((((((.(((((((.((((((.((..((.....)).)).)))))).)))))))))).)))))).).)).    reads      mm         sample
    .........caguaaguugaugggguccca...............................................    2          0          seq
    .........caguaaguugauggggucccag..............................................    4          0          seq
    .........caguaaguugauggggucccagg.............................................    4          0          seq
    ..........aguaaguugauggggucc.................................................    214        0          seq
    ..........aguaaguugaugggguccc................................................    285        0          seq
    ..........aguaaguugaugggguccca...............................................    2159       0          seq
    ..........aguaaguugauggggucccag..............................................    5329       0          seq
    ..........aguaaguugauggggucccagg.............................................    11733      0          seq
    ..........aguaaguugauggggucccaggu............................................    1211       0          seq
    ..........aguaaguugauggggucccagguu...........................................    142        0          seq
    ...........guaaguugaugggguccc................................................    1          0          seq
    ...........guaaguugaugggguccca...............................................    9          0          seq
    ...........guaaguugauggggucccag..............................................    32         0          seq
    ...........guaaguugauggggucccagg.............................................    103        0          seq
    ...........guaaguugauggggucccaggu............................................    10         0          seq
    ...........guaaguugauggggucccagguu...........................................    4          0          seq
    ............uaaguugauggggucccag..............................................    1          0          seq
    ............uaaguugauggggucccagg.............................................    11         0          seq
    ............uaaguugauggggucccaggu............................................    1          0          seq
    ............uaaguugauggggucccagguu...........................................    1          0          seq
    .............aaguugauggggucccag..............................................    1          0          seq
    .............aaguugauggggucccagg.............................................    3          0          seq
    .............aaguugauggggucccaggu............................................    1          0          seq
    ..............aguugauggggucccagg.............................................    2          0          seq
    ..............aguugauggggucccaggu............................................    1          0          seq
    ................................uugugaggaauucccgggacuacguc...................    1          0          seq
    ................................uugugaggaauucccgggacuacguca..................    2          0          seq
    ................................uugugaggaauucccgggacuacgucaac................    2          0          seq
    .................................ugugaggaauucccgggacuacgu....................    4          0          seq
    .................................ugugaggaauucccgggacuacguca..................    6          0          seq
    .................................ugugaggaauucccgggacuacgucaa.................    1          0          seq
    .................................ugugaggaauucccgggacuacgucaac................    3          0          seq
    .................................ugugaggaauucccgggacuacgucaacu...............    9          0          seq
    ........................................aauucccgggacuacgucaacuacuugc.........    1          0          seq
```

```
          cgi-miR-1990-5p
                                              cgi-miR-1990-3p
gcucgugggcaguaaguugauggggucccagguugugaggaauucccgggacuacgucaacuacuugcuuaagugcu

.........................................auucccgggacuacgucaacu...............    1       0    seq
...........................................ucccgggacuacgucaac................    1       0    seq
...........................................ucccgggacuacgucaacuac.............    2       0    seq
...........................................ucccgggacuacgucaacuacu............    5       0    seq
...........................................ucccgggacuacgucaacuacuu...........    1       0    seq
...........................................ucccgggacuacgucaacuacuugc.........    2       0    seq
............................................cccgggacuacgucaacuacu............    2       0    seq
............................................cccgggacuacgucaacuacuug..........    4       0    seq
............................................cccgggacuacgucaacuacuugc.........    3       0    seq
.............................................ccgggacuacgucaacua..............    104     0    seq
.............................................ccgggacuacgucaacuac.............    1154    0    seq
.............................................ccgggacuacgucaacuacu............    3332    0    seq
.............................................ccgggacuacgucaacuacuu...........    6663    0    seq
.............................................ccgggacuacgucaacuacuug..........    13939   0    seq
.............................................ccgggacuacgucaacuacuugc.........    3898    0    seq
.............................................ccgggacuacgucaacuacuugcu........    18      0    seq
.............................................ccgggacuacgucaacuacuugcuu.......    1       0    seq
..............................................cgggacuacgucaacuac.............    43469   0    seq
..............................................cgggacuacgucaacuacu............    145788  0    seq
..............................................cgggacuacgucaacuacuu...........    302358  0    seq
..............................................cgggacuacgucaacuacuug..........    985426  0    seq
..............................................cgggacuacgucaacuacuugc.........    893142  0    seq
..............................................cgggacuacgucaacuacuugcu........    11275   0    seq
..............................................cgggacuacgucaacuacuugcuu.......    1342    0    seq
..............................................cgggacuacgucaacuacuugcuua......    6       0    seq
...............................................gggacuacgucaacuacu............    272     0    seq
...............................................gggacuacgucaacuacuu...........    588     0    seq
...............................................gggacuacgucaacuacuug..........    2242    0    seq
...............................................gggacuacgucaacuacuugc.........    2109    0    seq
...............................................gggacuacgucaacuacuugcu........    33      0    seq
...............................................gggacuacgucaacuacuugcuu.......    5       0    seq
................................................ggacuacgucaacuacuu...........    4       0    seq
................................................ggacuacgucaacuacuug..........    17      0    seq
................................................ggacuacgucaacuacuugc.........    21      0    seq
................................................ggacuacgucaacuacuugcu........    1       0    seq
................................................ggacuacgucaacuacuugcuu.......    1       0    seq
.................................................gacuacgucaacuacuug..........    1       0    seq
.................................................gacuacgucaacuacuugcu........    1       0    seq
.................................................gacuacgucaacuacuugcuu.......    1       0    seq
```
