# Supplementary material for: Identification of Conserved and Novel MicroRNAs in the Pacific Oyster Crassostrea gigas by Deep Sequencing
Source: PLoS One. 2014 Aug 19;9(8):e104371. doi: 10.1371/journal.pone.0104371 (PMC4138081; doi:10.1371/journal.pone.0104371)
Supplement: File S2 — The compressed/ZIP file archive for the predicted precursors' secondary structures and reads alignment. (ZIP) [file pone.0104371.s010.zip › second structure and reads alignment for oyster miRNAs/conserved in table S4/cgi-miR-1991.pdf]

miRBase precursor : cgi-miR-1991
Total read count : 5110
cgi-miR-1991-5p read count : 2249
cgi-miR-1991-3p read count : 2861
remaining reads : 0

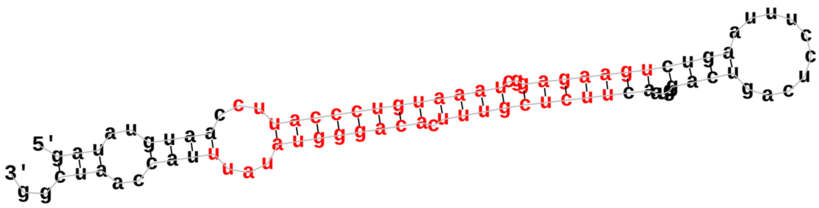

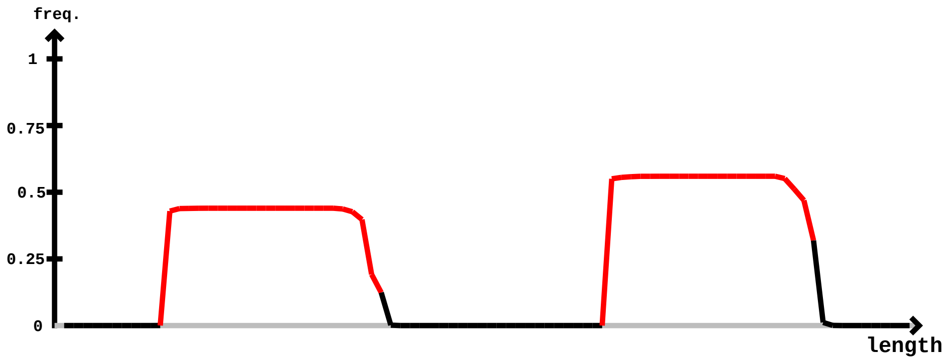

cgi-miR-1991-3p

cgi-miR-1991-5p

5'- gauauguaaccuuacccuguaaaucggagaagucugaauuuccucagucagguaacuucucguuucacaggguauauuuaccaaucgg -3' exp

(((..((((...((((((((((((..(((((((((((..........))))...))))))))))).))))))))...))))..)))..	reads	mm	sample
.........ccuuacccuguaaaucggagaagu...	1	0	seq
..........cuuacccuguaaaucgga..	15	0	seq
..........cuuacccuguaaaucggag...	50	0	seq
..........cuuacccuguaaaucggaga..	146	0	seq
..........cuuacccuguaaaucggagaa...	1039	0	seq
..........cuuacccuguaaaucggagaag..	332	0	seq
..........cuuacccuguaaaucggagaagu...	609	0	seq
..........cuuacccuguaaaucggagaaguc..	1	0	seq
...........uuacccuguaaaucggag...	3	0	seq
...........uuacccuguaaaucggaga..	3	0	seq
...........uuacccuguaaaucggagaa...	10	0	seq
...........uuacccuguaaaucggagaag..	13	0	seq
...........uuacccuguaaaucggagaagu...	13	0	seq
...........uuacccuguaaaucggagaaguc..	7	0	seq
............uacccuguaaaucggaga..	1	0	seq
............uacccuguaaaucggagaag..	2	0	seq
.............acccuguaaaucggagaa...	1	0	seq
.............acccuguaaaucggagaagu...	2	0	seq
..............cccuguaaaucggagaag..	1	0	seq
....uucucguuucacagggua..............	44	0	seq
....uucucguuucacaggguau.............	203	0	seq
....uucucguuucacaggguaua............	211	0	seq
....uucucguuucacaggguauau...........	759	0	seq
....uucucguuucacaggguauauu..........	1538	0	seq
....uucucguuucacaggguauauuu.........	55	0	seq
....uucucguuucacaggguauauuua........	2	0	seq
....ucucguuucacaggguau.............	1	0	seq
....ucucguuucacaggguaua............	3	0	seq
....ucucguuucacaggguauau...........	5	0	seq
....ucucguuucacaggguauauu..........	17	0	seq
....ucucguuucacaggguauauuua........	1	0	seq
....cucguuucacaggguauau...........	1	0	seq
....cucguuucacaggguauauu..........	12	0	seq
....ucguuucacaggguauau...........	4	0	seq

```
                                                        cgi-miR-1991-3p
          cgi-miR-1991-5p
gauauguaaccuuacccuguaaaucggagaagucugaauuuccucagucagguaacuucucguuucacaggguauauuuaccaaucgg

...........................................................ucguuucacaggguauauu..........    3          0          seq
...........................................................ucguuucacaggguauauuu.........    1          0          seq
.............................................................guuucacaggguauauuu.........    1          0          seq
```
