# Supplementary material for: Identification of Conserved and Novel MicroRNAs in the Pacific Oyster Crassostrea gigas by Deep Sequencing
Source: PLoS One. 2014 Aug 19;9(8):e104371. doi: 10.1371/journal.pone.0104371 (PMC4138081; doi:10.1371/journal.pone.0104371)
Supplement: File S2 — The compressed/ZIP file archive for the predicted precursors' secondary structures and reads alignment. (ZIP) [file pone.0104371.s010.zip › second structure and reads alignment for oyster miRNAs/conserved in table S4/cgi-miR-1992.pdf]

miRBase precursor : cgi-miR-1992
Total read count : 79206
cgi-miR-1992-5p read count : 1495
cgi-miR-1992-3p read count : 77708
remaining reads : 3

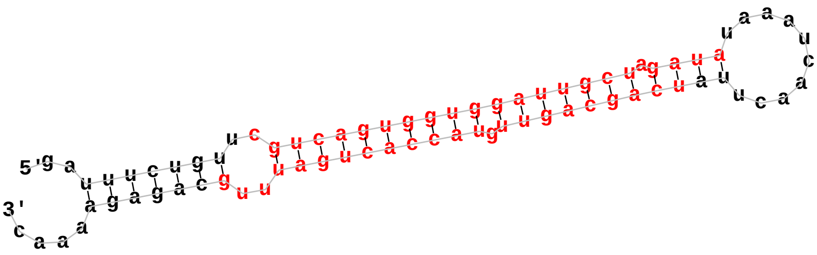

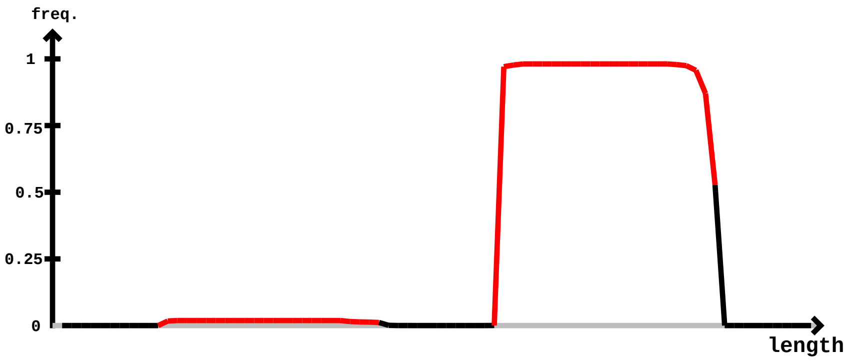


```
                                         cgi-miR-1992-3p
            cgi-miR-1992-5p
5'- gauuucuguucgucagugguggauugcuagauauaaaucaacuuaucagcaguuguaccacugauuugcagagaaaac  -3'   exp
    ..(((((((..(((((((((((((((((.((((.........)))))))))))).))))))))))..)))))))....   reads   mm   sample
    ....ucuguucgucagugguggauug...................................................   2       0    seq
    ..........cgucagugguggauugcu.................................................   6       0    seq
    ..........cgucagugguggauugcua................................................   262     0    seq
    ..........cgucagugguggauugcuag...............................................   97      0    seq
    ..........cgucagugguggauugcuaga..............................................   58      0    seq
    ..........cgucagugguggauugcuagau.............................................   113     0    seq
    ..........cgucagugguggauugcuagaua............................................   749     0    seq
    ..........cgucagugguggauugcuagauau...........................................   58      0    seq
    ..........cgucagugguggauugcuagauaua..........................................   4       0    seq
    ..........cgucagugguggauugcuagauauaa.........................................   3       0    seq
    ..........cgucagugguggauugcuagauauaaa........................................   1       0    seq
    ...........gucagugguggauugcua................................................   35      0    seq
    ...........gucagugguggauugcuag...............................................   10      0    seq
    ...........gucagugguggauugcuaga..............................................   2       0    seq
    ...........gucagugguggauugcuagau.............................................   3       0    seq
    ...........gucagugguggauugcuagaua............................................   90      0    seq
    ...........gucagugguggauugcuagauau...........................................   3       0    seq
    ..............agugguggauugcuagaua............................................   1       0    seq
    ......................................aacuuaucagcaguuguaccacugauu............   1       0    seq
    ...........................................aucagcaguuguaccacugau.............   2       0    seq
    ...........................................aucagcaguuguaccacugauuu...........   6       0    seq
    ...........................................aucagcaguuguaccacugauuug..........   20      0    seq
    ............................................ucagcaguuguaccacug................   197     0    seq
    ............................................ucagcaguuguaccacuga...............   314     0    seq
    ............................................ucagcaguuguaccacugau..............   1375    0    seq
    ............................................ucagcaguuguaccacugauu.............   6797    0    seq
    ............................................ucagcaguuguaccacugauuu............   26810   0    seq
    ............................................ucagcaguuguaccacugauuug...........   41364   0    seq
    ............................................ucagcaguuguaccacugauuugc..........   1       0    seq
    ............................................ucagcaguuguaccacugauuugca.........   3       0    seq
    .............................................cagcaguuguaccacuga...............   2       0    seq
    .............................................cagcaguuguaccacugau..............   5       0    seq
    .............................................cagcaguuguaccacugauu.............   52      0    seq
    .............................................cagcaguuguaccacugauuu............   228     0    seq
```

cgi-miR-1992-3p

cgi-miR-1992-5p

gauuucuguucgucaguggugggauugcuagauauaaaucaacuuaucagcaguuguaccacugauuugcagagaaaac

```
..............................................cagcaguuguaccacugauuug..........    198    0    seq
..............................................cagcaguuguaccacugauuugca........    1      0    seq
...............................................agcaguuguaccacugau.............    4      0    seq
...............................................agcaguuguaccacugauu............    23     0    seq
...............................................agcaguuguaccacugauuu...........    149    0    seq
...............................................agcaguuguaccacugauuug..........    151    0    seq
................................................gcaguuguaccacugauuug..........    3      0    seq
..................................................aguuguaccacugauuug..........    3      0    seq
```
