# Supplementary material for: Identification of Conserved and Novel MicroRNAs in the Pacific Oyster Crassostrea gigas by Deep Sequencing
Source: PLoS One. 2014 Aug 19;9(8):e104371. doi: 10.1371/journal.pone.0104371 (PMC4138081; doi:10.1371/journal.pone.0104371)
Supplement: File S2 — The compressed/ZIP file archive for the predicted precursors' secondary structures and reads alignment. (ZIP) [file pone.0104371.s010.zip › second structure and reads alignment for oyster miRNAs/conserved in table S4/cgi-miR-1993.pdf]

miRBase precursor : cgi-miR-1993
Total read count : 79054
cgi-miR-1993-5p read count : 4113
cgi-miR-1993-3p read count : 74940
remaining reads : 1

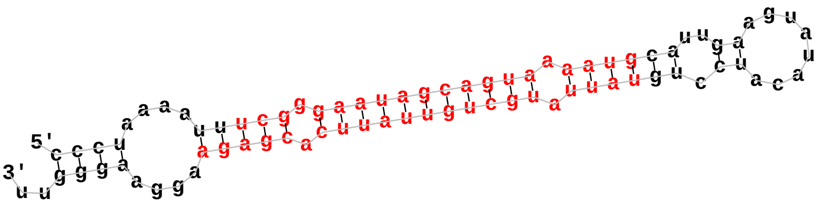

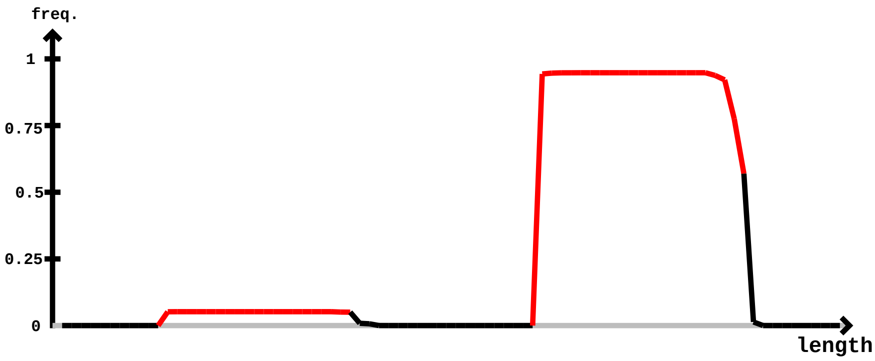


```
                                            cgi-miR-1993-3p
              cgi-miR-1993-5p
5'- cccuaaaauuucgggaauagcaguaaaaugcauugaaguauacauccuguauuaugcuguuauucacgagaaggaaggguu   -3'    exp
    ((((....(((((.(((((((((((.((((((..((........)).)))))).))))))))))).)))))....))))..   reads    mm    sample
    ........uuucgggaauagcaguaaaaug...................................................   1        0     seq
    .........uucgggaauagcaguaaaaug...................................................   2        0     seq
    ..........ucgggaauagcaguaaaa.....................................................   110      0     seq
    ..........ucgggaauagcaguaaaau....................................................   33       0     seq
    ..........ucgggaauagcaguaaaaug...................................................   3278     0     seq
    ..........ucgggaauagcaguaaaaugc..................................................   148      0     seq
    ..........ucgggaauagcaguaaaaugca.................................................   514      0     seq
    ..........ucgggaauagcaguaaaaugcau................................................   2        0     seq
    ..........ucgggaauagcaguaaaaugcauugaagua.........................................   1        0     seq
    ...........cgggaauagcaguaaaaug...................................................   13       0     seq
    ...........cgggaauagcaguaaaaugc..................................................   1        0     seq
    ...........cgggaauagcaguaaaaugca.................................................   5        0     seq
    ...........cgggaauagcaguaaaaugcau................................................   1        0     seq
    ............gggaauagcaguaaaaug...................................................   4        0     seq
    ............gggaauagcaguaaaaugca.................................................   1        0     seq
    ...............................................uguauuaugcuguuauucacgaga..........   1        0     seq
    ................................................guauuaugcuguuauucacga............   2        0     seq
    .................................................uauuaugcuguuauucac..............   790      0     seq
    .................................................uauuaugcuguuauucacg.............   1313     0     seq
    .................................................uauuaugcuguuauucacga............   11551    0     seq
    .................................................uauuaugcuguuauucacgag...........   16161    0     seq
    .................................................uauuaugcuguuauucacgaga..........   43738    0     seq
    .................................................uauuaugcuguuauucacgagaa.........   1022     0     seq
    .................................................uauuaugcuguuauucacgagaag........   8        0     seq
    .................................................uauuaugcuguuauucacgagaagga......   2        0     seq
    ..................................................auuaugcuguuauucacg.............   6        0     seq
    ..................................................auuaugcuguuauucacga............   18       0     seq
    ..................................................auuaugcuguuauucacgag...........   37       0     seq
    ..................................................auuaugcuguuauucacgaga..........   159      0     seq
    ..................................................auuaugcuguuauucacgagaa.........   22       0     seq
    ...................................................uuaugcuguuauucacga............   17       0     seq
    ...................................................uuaugcuguuauucacgag...........   10       0     seq
    ...................................................uuaugcuguuauucacgaga..........   51       0     seq
    ...................................................uuaugcuguuauucacgagaa.........   11       0     seq
```

```
                                                 cgi-miR-1993-3p
          cgi-miR-1993-5p
cccuaaaauuucgggaauagcaguaaaaugcauugaaguauacauccuguauuaugcuguuauucacgagaaggaagggguu

....................................................uaugcuguuauucacgag...........     1           0           seq
....................................................uaugcuguuauucacgaga..........     9           0           seq
....................................................uaugcuguuauucacgagaa.........     1           0           seq
.....................................................augcuguuauucacgaga..........     8           0           seq
.....................................................augcuguuauucacgagaa.........     2           0           seq
```
