# Supplementary material for: Identification of Conserved and Novel MicroRNAs in the Pacific Oyster Crassostrea gigas by Deep Sequencing
Source: PLoS One. 2014 Aug 19;9(8):e104371. doi: 10.1371/journal.pone.0104371 (PMC4138081; doi:10.1371/journal.pone.0104371)
Supplement: File S2 — The compressed/ZIP file archive for the predicted precursors' secondary structures and reads alignment. (ZIP) [file pone.0104371.s010.zip › second structure and reads alignment for oyster miRNAs/conserved in table S4/cgi-miR-1994a.pdf]

miRBase precursor : cgi-miR-1994a
Total read count : 112895
cgi-miR-1994a-5p read count : 2126
cgi-miR-1994a-3p read count : 110768
remaining reads : 1

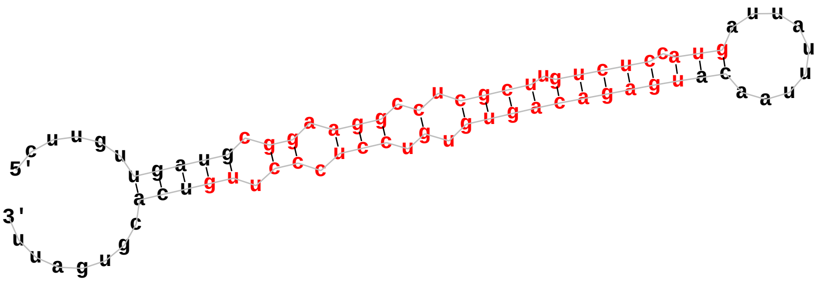

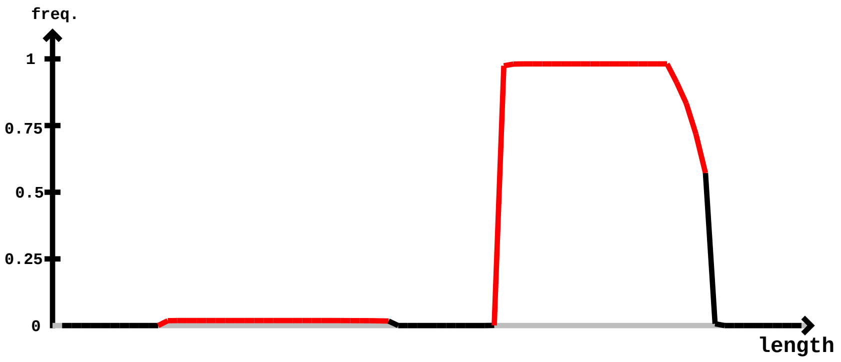

cgi-miR-1994a-3p

cgi-miR-1994a-5p

```
5'- cuuguugaugcggaaggccucgcuugucuccaugauuauuuaacaugagacaguguguccucccuugucacgugauu   -3'    exp
    (.(((.((((.((.(((.(.((((.(((((.(((.........)))))))))))).).))).)).))))))).)...    reads    mm    sample
    ..........cggaaggccucgcuuguc.................................................    17       0     seq
    ..........cggaaggccucgcuugucu................................................    23       0     seq
    ..........cggaaggccucgcuugucuc...............................................    18       0     seq
    ..........cggaaggccucgcuugucucc..............................................    18       0     seq
    ..........cggaaggccucgcuugucucca.............................................    52       0     seq
    ..........cggaaggccucgcuugucuccau............................................    79       0     seq
    ..........cggaaggccucgcuugucuccaug...........................................    1842     0     seq
    ..........cggaaggccucgcuugucuccauga..........................................    2        0     seq
    ...........ggaaggccucgcuugucucca.............................................    1        0     seq
    ...........ggaaggccucgcuugucuccau............................................    4        0     seq
    ...........ggaaggccucgcuugucuccaug...........................................    69       0     seq
    ............gaaggccucgcuugucucca.............................................    1        0     seq
    .........................................aacaugagacaguguguccucccuug..........    1        0     seq
    ...........................................caugagacaguguguccucccuug..........    3        0     seq
    ............................................augagacaguguguccuc...............    2        0     seq
    ............................................augagacaguguguccuccc.............    7        0     seq
    ............................................augagacaguguguccucccu............    12       0     seq
    ............................................augagacaguguguccucccuu...........    5        0     seq
    ............................................augagacaguguguccucccuug..........    32       0     seq
    ............................................augagacaguguguccucccuugu.........    1        0     seq
    .............................................ugagacaguguguccucc..............    7855     0     seq
    .............................................ugagacaguguguccuccc.............    8798     0     seq
    .............................................ugagacaguguguccucccu............    12814    0     seq
    .............................................ugagacaguguguccucccuu...........    16490    0     seq
    .............................................ugagacaguguguccucccuug..........    63210    0     seq
    .............................................ugagacaguguguccucccuugu.........    747      0     seq
    .............................................ugagacaguguguccucccuuguc........    1        0     seq
    ..............................................gagacaguguguccuccc.............    66       0     seq
    ..............................................gagacaguguguccucccu............    62       0     seq
    ..............................................gagacaguguguccucccuu...........    81       0     seq
    ..............................................gagacaguguguccucccuug..........    489      0     seq
    ..............................................gagacaguguguccucccuugu.........    12       0     seq
    ...............................................agacaguguguccucccu............    6        0     seq
    ...............................................agacaguguguccucccuu...........    7        0     seq
```

```
                                             cgi-miR-1994a-3p
          cgi-miR-1994a-5p
cuuguugaugcggaaggccucgcuugucuccaugauuauuuaacaugagacagugguguccucccuugucacgugauu

..............................................agacagugguguccucccuug..........    66         0          seq
..............................................agacagugguguccucccuugu.........    1          0          seq
................................................acagugguguccucccuug..........    1          0          seq
```
