# Supplementary material for: Identification of Conserved and Novel MicroRNAs in the Pacific Oyster Crassostrea gigas by Deep Sequencing
Source: PLoS One. 2014 Aug 19;9(8):e104371. doi: 10.1371/journal.pone.0104371 (PMC4138081; doi:10.1371/journal.pone.0104371)
Supplement: File S2 — The compressed/ZIP file archive for the predicted precursors' secondary structures and reads alignment. (ZIP) [file pone.0104371.s010.zip › second structure and reads alignment for oyster miRNAs/conserved in table S4/cgi-miR-2001.pdf]

miRBase precursor : cgi-miR-2001
Total read count : 13417
cgi-miR-2001-5p read count : 11607
cgi-miR-2001-3p read count : 23
remaining reads : 1787

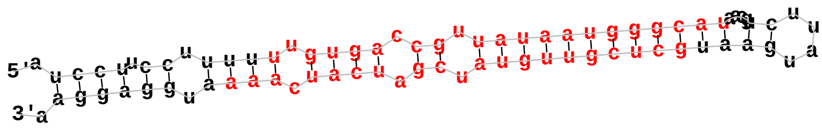

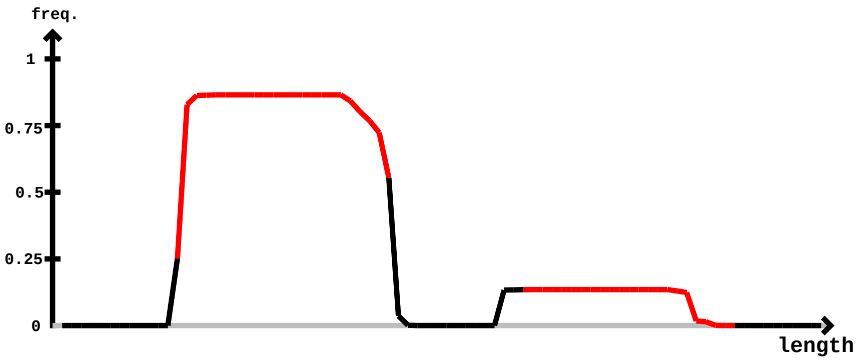


```
                    cgi-miR-2001-5p
                                          cgi-miR-2001-3p
5'- auccuuccuuuuuugugaccguuauaaugggcauaacgucuuaugaaugcucguuguaucgaucaucaaaauggaggaa   -3'    exp
    .((((.((.((((.((((.((.((((((((((((....((....)))))))))))))).)).)))).)))).)))))).    reads      mm         sample
    ...........uuugugaccguuauaaug..................................................    309        0          seq
    ...........uuugugaccguuauaaugg.................................................    170        0          seq
    ...........uuugugaccguuauaauggg................................................    125        0          seq
    ...........uuugugaccguuauaaugggc...............................................    393        0          seq
    ...........uuugugaccguuauaaugggca..............................................    1523       0          seq
    ...........uuugugaccguuauaaugggcau.............................................    855        0          seq
    ...........uuugugaccguuauaaugggcaua............................................    6          0          seq
    ...........uuugugaccguuauaaugggcauaa...........................................    2          0          seq
    ............uugugaccguuauaaugg.................................................    368        0          seq
    ............uugugaccguuauaauggg................................................    297        0          seq
    ............uugugaccguuauaaugggc...............................................    196        0          seq
    ............uugugaccguuauaaugggca..............................................    681        0          seq
    ............uugugaccguuauaaugggcau.............................................    5884       0          seq
    ............uugugaccguuauaaugggcaua............................................    296        0          seq
    ............uugugaccguuauaaugggcauaa...........................................    10         0          seq
    .............ugugaccguuauaauggg................................................    33         0          seq
    .............ugugaccguuauaaugggc...............................................    10         0          seq
    .............ugugaccguuauaaugggca..............................................    64         0          seq
    .............ugugaccguuauaaugggcau.............................................    197        0          seq
    .............ugugaccguuauaaugggcaua............................................    152        0          seq
    .............ugugaccguuauaaugggcauaa...........................................    5          0          seq
    ..............gugaccguuauaaugggc...............................................    1          0          seq
    ..............gugaccguuauaaugggca..............................................    5          0          seq
    ..............gugaccguuauaaugggcau.............................................    5          0          seq
    ..............gugaccguuauaaugggcaua............................................    1          0          seq
    ...............ugaccguuauaaugggcau.............................................    4          0          seq
    ...............ugaccguuauaaugggcaua............................................    15         0          seq
    .....................................gucuuaugaaugcucguugu......................    1          0          seq
    .....................................gucuuaugaaugcucguuguaucgauc...............    1          0          seq
    .............................................aaugcucguuguaucgau................    68         0          seq
    .............................................aaugcucguuguaucgauc...............    77         0          seq
    .............................................aaugcucguuguaucgauca..............    1425       0          seq
    .............................................aaugcucguuguaucgaucau.............    37         0          seq
    .............................................aaugcucguuguaucgaucauc............    173        0          seq
```

```
            cgi-miR-2001-5p
                                                cgi-miR-2001-3p
auccuucuuuuuugugaccguuauaaaugggcauaacgucuuaugaaugcucguuguaucgaucaucaaaauggaggaa

.............................................aaugcucguuguaucgaucauca...........    5    0    seq
..............................................augcucguuguaucgauc...............    1    0    seq
..............................................augcucguuguaucgauca..............    6    0    seq
..............................................augcucguuguaucgaucau.............    1    0    seq
...............................................ugcucguuguaucgaucauc............    6    0    seq
...............................................ugcucguuguaucgaucauca...........    5    0    seq
................................................gcucguuguaucgaucauca...........    2    0    seq
................................................gcucguuguaucgaucaucaa..........    1    0    seq
.................................................cucguuguaucgaucaucaaaa........    1    0    seq
```
