# Supplementary material for: Identification of Conserved and Novel MicroRNAs in the Pacific Oyster Crassostrea gigas by Deep Sequencing
Source: PLoS One. 2014 Aug 19;9(8):e104371. doi: 10.1371/journal.pone.0104371 (PMC4138081; doi:10.1371/journal.pone.0104371)
Supplement: File S2 — The compressed/ZIP file archive for the predicted precursors' secondary structures and reads alignment. (ZIP) [file pone.0104371.s010.zip › second structure and reads alignment for oyster miRNAs/conserved in table S4/cgi-miR-210.pdf]

miRBase precursor : cgi-miR-210
Total read count : 11912
cgi-miR-210-5p read count : 476
cgi-miR-210-3p read count : 11436
remaining reads : 0

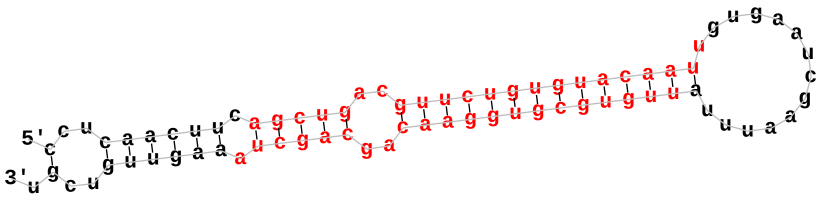

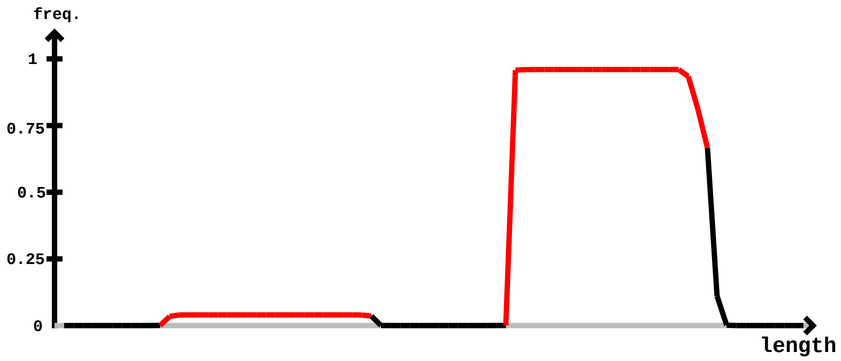

cgi-miR-210-3p

cgi-miR-210-5p

```
5'- ccucaacuucagcugacguucuguguacaauugugaaucgaauuuauugugcguggaacagcagcuaaaguugucgu   -3'    exp
    (..((((((.(((((..((((((((((((((..............))))))))))))))..))))).))))))..).    reads      mm         sample
    ........ucagcugacguucuguguacaa...............................................    1          0          seq
    .........cagcugacguucuguguacaau..............................................    3          0          seq
    .........cagcugacguucuguguacaauu.............................................    3          0          seq
    ..........agcugacguucuguguacaa...............................................    3          0          seq
    ..........agcugacguucuguguacaau..............................................    36         0          seq
    ..........agcugacguucuguguacaauu.............................................    363        0          seq
    ...........gcugacguucuguguacaa...............................................    1          0          seq
    ...........gcugacguucuguguacaau..............................................    8          0          seq
    ...........gcugacguucuguguacaauu.............................................    55         0          seq
    ...........gcugacguucuguguacaauug............................................    1          0          seq
    ...........gcugacguucuguguacaauugu...........................................    1          0          seq
    .............ugacguucuguguacaauu.............................................    1          0          seq
    ............................................uauugugcguggaacagca..............    1          0          seq
    ............................................uauugugcguggaacagcagc............    1          0          seq
    ............................................uauugugcguggaacagcagcu...........    1          0          seq
    .............................................auugugcguggaacagcagc............    1          0          seq
    ..............................................uugugcguggaacagcag.............    307        0          seq
    ..............................................uugugcguggaacagcagc............    1447       0          seq
    ..............................................uugugcguggaacagcagcu...........    1737       0          seq
    ..............................................uugugcguggaacagcagcua..........    6600       0          seq
    ..............................................uugugcguggaacagcagcuaa.........    1299       0          seq
    ..............................................uugugcguggaacagcagcuaaa........    16         0          seq
    ..............................................uugugcguggaacagcagcuaaag.......    1          0          seq
    ...............................................ugugcguggaacagcagc............    2          0          seq
    ...............................................ugugcguggaacagcagcu...........    6          0          seq
    ...............................................ugugcguggaacagcagcua..........    8          0          seq
    ...............................................ugugcguggaacagcagcuaa.........    3          0          seq
    ................................................gugcguggaacagcagcu...........    4          0          seq
    ................................................gugcguggaacagcagcua..........    1          0          seq
    ................................................gugcguggaacagcagcuaa.........    1          0          seq
```
