# Supplementary material for: Identification of Conserved and Novel MicroRNAs in the Pacific Oyster Crassostrea gigas by Deep Sequencing
Source: PLoS One. 2014 Aug 19;9(8):e104371. doi: 10.1371/journal.pone.0104371 (PMC4138081; doi:10.1371/journal.pone.0104371)
Supplement: File S2 — The compressed/ZIP file archive for the predicted precursors' secondary structures and reads alignment. (ZIP) [file pone.0104371.s010.zip › second structure and reads alignment for oyster miRNAs/conserved in table S4/cgi-miR-216a.pdf]

miRBase precursor : cgi-miR-216a
Total read count : 55914
cgi-miR-216a-5p read count: 46430
cgi-miR-216a-3p read count: 9349
remaining reads : 135

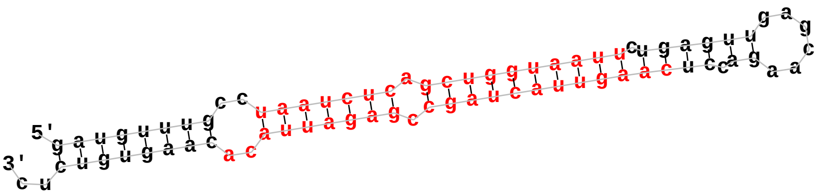

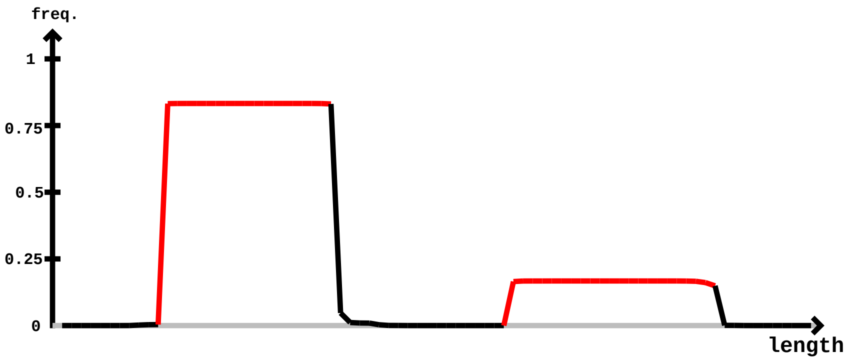


```
                                                cgi-miR-216a-3p
              cgi-miR-216a-5p
5'- gauguuugccuaaucucagcugguaauucugaguugagcaagaccucaaguuacuagccgagauuacacaagugucuc   -3'    exp
    ((((((((..(((((((.((((((((((.((((((......)).))))))))))))))).)))))))..))))))))..    reads      mm         sample
    ......ugccuaaucucagcugguaau...................................................    5          0          seq
    ......ugccuaaucucagcugguaauu..................................................    3          0          seq
    .......gccuaaucucagcuggua.....................................................    2          0          seq
    .......gccuaaucucagcugguaa....................................................    16         0          seq
    .......gccuaaucucagcugguaau...................................................    61         0          seq
    .......gccuaaucucagcugguaauu..................................................    18         0          seq
    .......gccuaaucucagcugguaauuc.................................................    2          0          seq
    ........ccuaaucucagcugguaa....................................................    1          0          seq
    ........ccuaaucucagcugguaau...................................................    12         0          seq
    ........ccuaaucucagcugguaauu..................................................    60         0          seq
    ........ccuaaucucagcugguaauuc.................................................    7          0          seq
    .........cuaaucucagcugguaau...................................................    1          0          seq
    .........cuaaucucagcugguaauu..................................................    17         0          seq
    ..........uaaucucagcugguaauu..................................................    43738      0          seq
    ..........uaaucucagcugguaauuc.................................................    1968       0          seq
    ..........uaaucucagcugguaauucu................................................    95         0          seq
    ..........uaaucucagcugguaauucug...............................................    23         0          seq
    ..........uaaucucagcugguaauucuga..............................................    336        0          seq
    ..........uaaucucagcugguaauucugag.............................................    142        0          seq
    ..........uaaucucagcugguaauucugagu............................................    10         0          seq
    ..........uaaucucagcugguaauucugaguu...........................................    14         0          seq
    ..........uaaucucagcugguaauucugaguug..........................................    2          0          seq
    ...........aaucucagcugguaauuc.................................................    19         0          seq
    ...........aaucucagcugguaauucu................................................    2          0          seq
    ...........aaucucagcugguaauucuga..............................................    5          0          seq
    ...........aaucucagcugguaauucugag.............................................    2          0          seq
    ...........aaucucagcugguaauucugagu............................................    1          0          seq
    ............aucucagcugguaauucugagu............................................    1          0          seq
    ...............ucagcugguaauucugag.............................................    2          0          seq
    .............................................ucaaguuacuagccgagauuaca..........    2          0          seq
    ..............................................caaguuacuagccgagau...............    17         0          seq
    ..............................................caaguuacuagccgagauu..............    49         0          seq
    ..............................................caaguuacuagccgagauua.............    274        0          seq
    ..............................................caaguuacuagccgagauuac............    665        0          seq
```

```
                                              cgi-miR-216a-3p
          cgi-miR-216a-5p
gauguuugccuaaucucagcugguaauucugaguugagcaagaccucaaguuacuagccgagauuacacaagugucuc

..............................................caaguuacuagccgagauuaca..........    8214    0    seq
..............................................caaguuacuagccgagauuacac.........    1       0    seq
..............................................caaguuacuagccgagauuacaca........    2       0    seq
..............................................caaguuacuagccgagauuacacaa.......    1       0    seq
...............................................aaguuacuagccgagauua............    3       0    seq
...............................................aaguuacuagccgagauuac...........    5       0    seq
...............................................aaguuacuagccgagauuaca..........    72      0    seq
...............................................aaguuacuagccgagauuacac.........    6       0    seq
...............................................aaguuacuagccgagauuacaca........    29      0    seq
................................................aguuacuagccgagauuaca..........    1       0    seq
................................................aguuacuagccgagauuacacaa.......    2       0    seq
.................................................guuacuagccgagauuaca..........    3       0    seq
..................................................uuacuagccgagauuaca..........    1       0    seq
..................................................uuacuagccgagauuacacaag......    2       0    seq
```
