# Supplementary material for: Identification of Conserved and Novel MicroRNAs in the Pacific Oyster Crassostrea gigas by Deep Sequencing
Source: PLoS One. 2014 Aug 19;9(8):e104371. doi: 10.1371/journal.pone.0104371 (PMC4138081; doi:10.1371/journal.pone.0104371)
Supplement: File S2 — The compressed/ZIP file archive for the predicted precursors' secondary structures and reads alignment. (ZIP) [file pone.0104371.s010.zip › second structure and reads alignment for oyster miRNAs/conserved in table S4/cgi-miR-216b.pdf]

miRBase precursor : cgi-miR-216b
Total read count : 63688
cgi-miR-216b-5p read count : 63560
cgi-miR-216b-3p read count : 127
remaining reads : 1

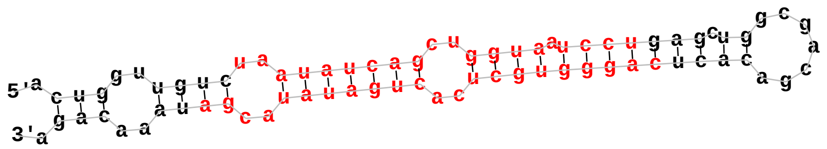

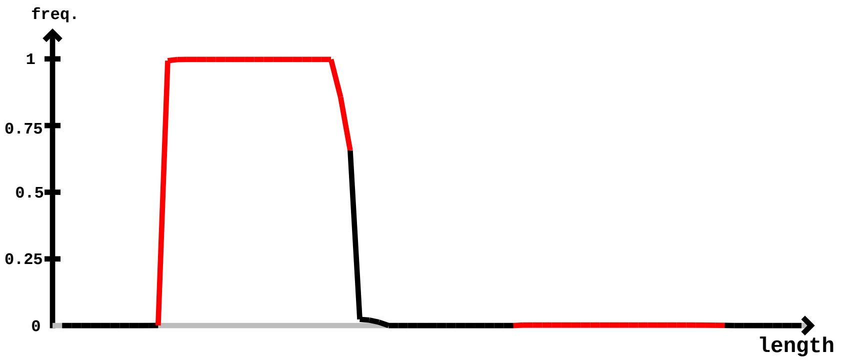


```
                                                  cgi-miR-216b-3p
              cgi-miR-216b-5p
5'- acugguugucuaauaucagcugguaauccugagcuggcgacgacacucagggugcucacugauauacgauaaacaga   -3'    exp
    .(((..((((..(((((((..((((.(((((((.((.......)))))))))))))..)))))))..))))..))).    reads      mm         sample
    ........ucuaauaucagcugguaauc.................................................    1          0          seq
    ........ucuaauaucagcugguaaucc................................................    1          0          seq
    ........ucuaauaucagcugguaauccu...............................................    3          0          seq
    ........ucuaauaucagcugguaauccug..............................................    1          0          seq
    .........cuaauaucagcugguaauc.................................................    2          0          seq
    .........cuaauaucagcugguaaucc................................................    4          0          seq
    .........cuaauaucagcugguaauccu...............................................    10         0          seq
    .........cuaauaucagcugguaauccuga.............................................    1          0          seq
    ..........uaauaucagcugguaauc.................................................    8989       0          seq
    ..........uaauaucagcugguaaucc................................................    12686      0          seq
    ..........uaauaucagcugguaauccu...............................................    40102      0          seq
    ..........uaauaucagcugguaauccug..............................................    180        0          seq
    ..........uaauaucagcugguaauccuga.............................................    493        0          seq
    ..........uaauaucagcugguaauccugag............................................    790        0          seq
    ...........aauaucagcugguaaucc................................................    53         0          seq
    ...........aauaucagcugguaauccu...............................................    196        0          seq
    ...........aauaucagcugguaauccug..............................................    2          0          seq
    ...........aauaucagcugguaauccuga.............................................    3          0          seq
    ...........aauaucagcugguaauccugag............................................    9          0          seq
    ............auaucagcugguaauccu...............................................    28         0          seq
    ............auaucagcugguaauccug..............................................    1          0          seq
    ............auaucagcugguaauccuga.............................................    1          0          seq
    ............auaucagcugguaauccugag............................................    3          0          seq
    ..............aucagcugguaauccugag............................................    1          0          seq
    ..........................uccugagcuggcgacgacacu..............................    1          0          seq
    ..............................................ucagggugcucacugauauacg.........    1          0          seq
    ...............................................cagggugcucacugauau............    10         0          seq
    ...............................................cagggugcucacugauaua...........    11         0          seq
    ...............................................cagggugcucacugauauac..........    25         0          seq
    ...............................................cagggugcucacugauauacg.........    36         0          seq
    ...............................................cagggugcucacugauauacga........    41         0          seq
    ................................................agggugcucacugauaua...........    1          0          seq
    ................................................agggugcucacugauauac..........    1          0          seq
    ................................................agggugcucacugauauacga........    1          0          seq
```

cgi-miR-216b-3p

cgi-miR-216b-5p

acugguugucuaauaucagcugguaauccugagcuggcgacgacacucagggugcucacugauauacgauaaacaga
