# Supplementary material for: Identification of Conserved and Novel MicroRNAs in the Pacific Oyster Crassostrea gigas by Deep Sequencing
Source: PLoS One. 2014 Aug 19;9(8):e104371. doi: 10.1371/journal.pone.0104371 (PMC4138081; doi:10.1371/journal.pone.0104371)
Supplement: File S2 — The compressed/ZIP file archive for the predicted precursors' secondary structures and reads alignment. (ZIP) [file pone.0104371.s010.zip › second structure and reads alignment for oyster miRNAs/conserved in table S4/cgi-miR-219.pdf]

miRBase precursor	: cgi-miR-219
Total read count	: 11587
cgi-miR-219-5p read count	: 9253
cgi-miR-219-3p read count	: 2334
remaining reads	: 0

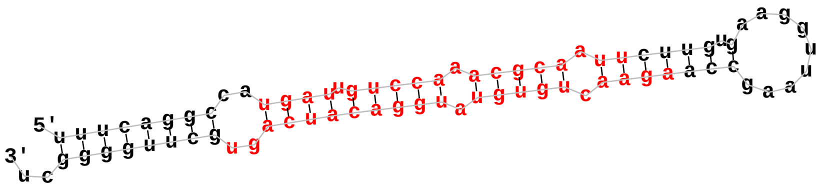

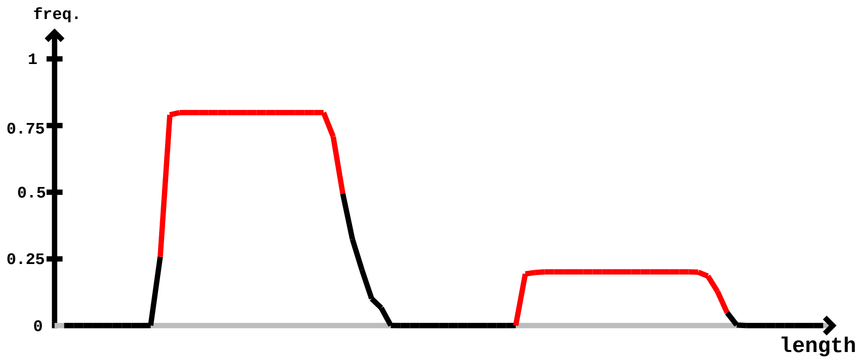


```
                 cgi-miR-219-5p
                                         cgi-miR-219-3p
5'- uuucaggccaugauuguccaaacgcaauucuugugaagguuaagccaagaacuguguauggacaucagugcuuggggcu  -3'   exp
    .(((((((..((((.(((((.(((((.((((((.(.........))))))).))))).)))))))))..)))))))...   reads      mm         sample
    ........caugauuguccaaacgcaa....................................................   1          0          seq
    ........caugauuguccaaacgcaau...................................................   1          0          seq
    .........augauuguccaaacgcaa....................................................   1040       0          seq
    .........augauuguccaaacgcaau...................................................   1057       0          seq
    .........augauuguccaaacgcaauu..................................................   396        0          seq
    .........augauuguccaaacgcaauuc.................................................   228        0          seq
    .........augauuguccaaacgcaauucu................................................   142        0          seq
    .........augauuguccaaacgcaauucuu...............................................   94         0          seq
    .........augauuguccaaacgcaauucuug..............................................   30         0          seq
    ..........ugauuguccaaacgcaau...................................................   1422       0          seq
    ..........ugauuguccaaacgcaauu..................................................   1561       0          seq
    ..........ugauuguccaaacgcaauuc.................................................   1093       0          seq
    ..........ugauuguccaaacgcaauucu................................................   1071       0          seq
    ..........ugauuguccaaacgcaauucuu...............................................   299        0          seq
    ..........ugauuguccaaacgcaauucuug..............................................   718        0          seq
    ..........ugauuguccaaacgcaauucuugu.............................................   3          0          seq
    ...........gauuguccaaacgcaauu..................................................   17         0          seq
    ...........gauuguccaaacgcaauuc.................................................   27         0          seq
    ...........gauuguccaaacgcaauucu................................................   27         0          seq
    ...........gauuguccaaacgcaauucuu...............................................   8          0          seq
    ...........gauuguccaaacgcaauucuug..............................................   17         0          seq
    ............auuguccaaacgcaauucuug..............................................   1          0          seq
    ..............................................aagaacuguguauggacaucag...........   2          0          seq
    ...............................................agaacuguguauggacau..............   14         0          seq
    ...............................................agaacuguguauggacauc.............   165        0          seq
    ...............................................agaacuguguauggacauca............   635        0          seq
    ...............................................agaacuguguauggacaucag...........   882        0          seq
    ...............................................agaacuguguauggacaucagu..........   528        0          seq
    ...............................................agaacuguguauggacaucagug.........   18         0          seq
    ................................................gaacuguguauggacauc.............   7          0          seq
    ................................................gaacuguguauggacauca............   17         0          seq
    ................................................gaacuguguauggacaucag...........   25         0          seq
    ................................................gaacuguguauggacaucagu..........   9          0          seq
    ................................................gaacuguguauggacaucagug.........   3          0          seq
```

```
          cgi-miR-219-5p
                                             cgi-miR-219-3p
uuucaggccaugauuguccaaacgcaauucuugugaagguuaagccaagaacuguguauggacaucagugcuuggggcu

................................................aacuguguauggacauca............    13          0           seq
................................................aacuguguauggacaucag...........    11          0           seq
................................................aacuguguauggacaucagu..........    1           0           seq
................................................aacuguguauggacaucagug.........    3           0           seq
.................................................acuguguauggacaucag...........    1           0           seq
```
