# Supplementary material for: Identification of Conserved and Novel MicroRNAs in the Pacific Oyster Crassostrea gigas by Deep Sequencing
Source: PLoS One. 2014 Aug 19;9(8):e104371. doi: 10.1371/journal.pone.0104371 (PMC4138081; doi:10.1371/journal.pone.0104371)
Supplement: File S2 — The compressed/ZIP file archive for the predicted precursors' secondary structures and reads alignment. (ZIP) [file pone.0104371.s010.zip › second structure and reads alignment for oyster miRNAs/conserved in table S4/cgi-miR-242.pdf]

miRBase precursor : cgi-miR-242
Total read count : 542755
cgi-miR-242-5p read count : 542728
cgi-miR-242-3p read count : 25
remaining reads : 2

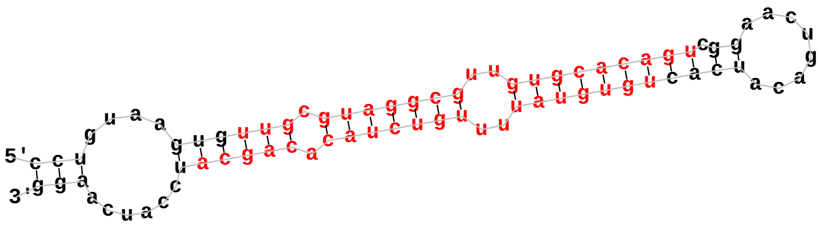

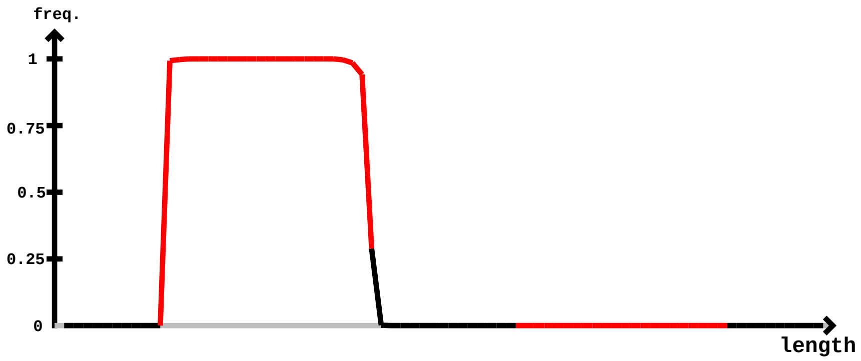


```
                                                   cgi-miR-242-3p
          cgi-miR-242-5p
5'- ccuguaaguguugcguaggcguugugcacagucggaacugacaucacuguguauuuugucuacacagcauccaucaagg  -3'   exp
    (((....((((((.(((((((..(((((((((.((........)))))))))))..))))))).))))))......)))   reads      mm         sample
    ..........uugcguaggcguugugca...................................................   1863       0          seq
    ..........uugcguaggcguugugcac..................................................   6221       0          seq
    ..........uugcguaggcguugugcaca.................................................   23395      0          seq
    ..........uugcguaggcguugugcacag................................................   352819     0          seq
    ..........uugcguaggcguugugcacagu...............................................   154395     0          seq
    ..........uugcguaggcguugugcacaguc..............................................   320        0          seq
    ..........uugcguaggcguugugcacagucg.............................................   4          0          seq
    ..........uugcguaggcguugugcacagucgg............................................   1          0          seq
    ..........uugcguaggcguugugcacagucgga...........................................   1          0          seq
    ..........uugcguaggcguugugcacagucggaa..........................................   1          0          seq
    ..........uugcguaggcguugugcacagucggaac.........................................   2          0          seq
    ...........ugcguaggcguugugcac..................................................   31         0          seq
    ...........ugcguaggcguugugcaca.................................................   65         0          seq
    ...........ugcguaggcguugugcacag................................................   948        0          seq
    ...........ugcguaggcguugugcacagu...............................................   826        0          seq
    ...........ugcguaggcguugugcacaguc..............................................   382        0          seq
    ............gcguaggcguugugcaca.................................................   26         0          seq
    ............gcguaggcguugugcacag................................................   698        0          seq
    ............gcguaggcguugugcacagu...............................................   702        0          seq
    ............gcguaggcguugugcacaguc..............................................   6          0          seq
    .............cguaggcguugugcacag................................................   13         0          seq
    .............cguaggcguugugcacagu...............................................   4          0          seq
    .............cguaggcguugugcacagucg.............................................   1          0          seq
    ..............guaggcguugugcacagu...............................................   6          0          seq
    ...............................................uguguauuuugucuacacagca..........   22         0          seq
    ................................................guguauuuugucuacacagca..........   3          0          seq
```
