# Supplementary material for: Identification of Conserved and Novel MicroRNAs in the Pacific Oyster Crassostrea gigas by Deep Sequencing
Source: PLoS One. 2014 Aug 19;9(8):e104371. doi: 10.1371/journal.pone.0104371 (PMC4138081; doi:10.1371/journal.pone.0104371)
Supplement: File S2 — The compressed/ZIP file archive for the predicted precursors' secondary structures and reads alignment. (ZIP) [file pone.0104371.s010.zip › second structure and reads alignment for oyster miRNAs/conserved in table S4/cgi-miR-252a.pdf]

miRBase precursor : cgi-miR-252a
Total read count : 20908
cgi-miR-252a-5p read count: 19163
cgi-miR-252a-3p read count: 1718
remaining reads : 27

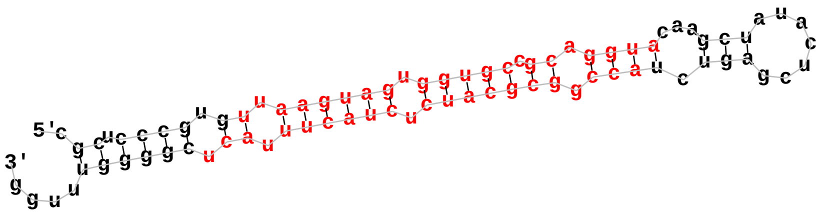

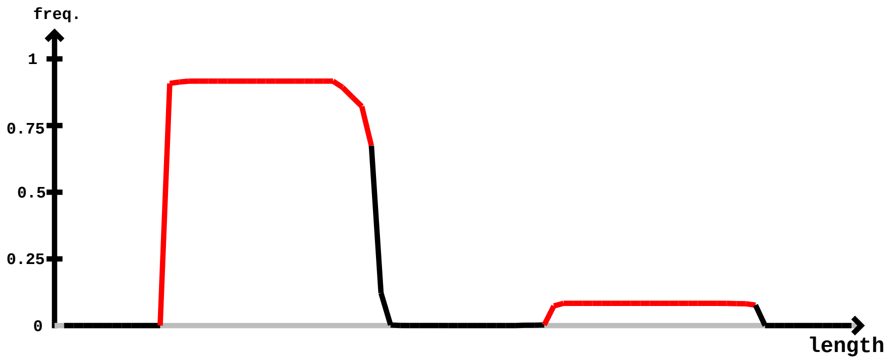


```
                                                              cgi-miR-252a-3p
           cgi-miR-252a-5p
5'- cgcucccguguuaaguaguggugccgcagguacaagcuauacucgagucuaccggcgcaucucuacuuuacucgggguuugg   -3'    exp
    .((.((((.((.((((((.(((((.((.((((...(((.......))).)))).))))))).)))))).)).))))))....    reads      mm         sample
    ..........uuaaguaguggugccgca......................................................    493        0          seq
    ..........uuaaguaguggugccgcag.....................................................    744        0          seq
    ..........uuaaguaguggugccgcagg....................................................    732        0          seq
    ..........uuaaguaguggugccgcaggu...................................................    3071       0          seq
    ..........uuaaguaguggugccgcaggua..................................................    11420      0          seq
    ..........uuaaguaguggugccgcagguac.................................................    2484       0          seq
    ..........uuaaguaguggugccgcagguaca................................................    36         0          seq
    ..........uuaaguaguggugccgcagguacaa...............................................    5          0          seq
    ..........uuaaguaguggugccgcagguacaagcua...........................................    1          0          seq
    ...........uaaguaguggugccgcag.....................................................    5          0          seq
    ...........uaaguaguggugccgcagg....................................................    3          0          seq
    ...........uaaguaguggugccgcaggu...................................................    13         0          seq
    ...........uaaguaguggugccgcaggua..................................................    70         0          seq
    ...........uaaguaguggugccgcagguac.................................................    17         0          seq
    ............aaguaguggugccgcagg....................................................    3          0          seq
    ............aaguaguggugccgcaggu...................................................    9          0          seq
    ............aaguaguggugccgcaggua..................................................    43         0          seq
    ............aaguaguggugccgcagguac.................................................    13         0          seq
    ............aaguaguggugccgcagguaca................................................    1          0          seq
    .............aguaguggugccgcaggua..................................................    1          0          seq
    ................................caagcuauacucgagucua...............................    1          0          seq
    ...............................................ucuaccggcgcaucucuacu...............    1          0          seq
    ...............................................ucuaccggcgcaucucuacuuu.............    13         0          seq
    ...............................................ucuaccggcgcaucucuacuuua............    1          0          seq
    ...............................................ucuaccggcgcaucucuacuuuac...........    3          0          seq
    ...............................................ucuaccggcgcaucucuacuuuacu..........    7          0          seq
    ................................................cuaccggcgcaucucuacuuuacu..........    1          0          seq
    .................................................uaccggcgcaucucuacuuu.............    1          0          seq
    .................................................uaccggcgcaucucuacuuuac...........    12         0          seq
    .................................................uaccggcgcaucucuacuuuacu..........    9          0          seq
    ..................................................accggcgcaucucuacu...............    3          0          seq
    ..................................................accggcgcaucucuacuuu.............    5          0          seq
    ..................................................accggcgcaucucuacuuua............    6          0          seq
    ..................................................accggcgcaucucuacuuuac...........    78         0          seq
```

```
                                                  cgi-miR-252a-3p
          cgi-miR-252a-5p
cgcucccguguuaaguaguggugccgcagguacaagcuauacucgagucuaccggcgcaucucuacuuuacucggggguuugg

..................................................accggcgcaucucuacuuuacu..........    1396      0         seq
...................................................ccggcgcaucucuacuuu.............    2         0         seq
...................................................ccggcgcaucucuacuuuac...........    15        0         seq
...................................................ccggcgcaucucuacuuuacu..........    190       0         seq
```
