# Supplementary material for: Identification of Conserved and Novel MicroRNAs in the Pacific Oyster Crassostrea gigas by Deep Sequencing
Source: PLoS One. 2014 Aug 19;9(8):e104371. doi: 10.1371/journal.pone.0104371 (PMC4138081; doi:10.1371/journal.pone.0104371)
Supplement: File S2 — The compressed/ZIP file archive for the predicted precursors' secondary structures and reads alignment. (ZIP) [file pone.0104371.s010.zip › second structure and reads alignment for oyster miRNAs/conserved in table S4/cgi-miR-252b.pdf]

miRBase precursor : cgi-miR-252b
Total read count : 10734
cgi-miR-252b-5p read count : 10658
cgi-miR-252b-3p read count : 76
remaining reads : 0

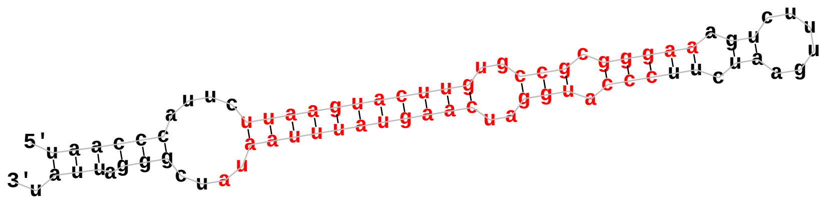

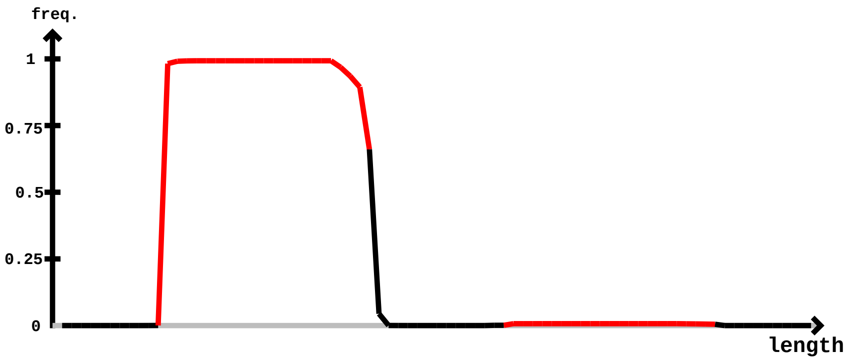


```
                                             cgi-miR-252b-3p
             cgi-miR-252b-5p
5'- uaacccauucuuaaguacuugugccgcgggaaagucuuugaaucuucccauggaucaaguauuuaauaucgggauuau  -3'   exp
    ...(((....((((((((((..(((.(((((.((......)).))))).)))..)))))))))))....))).....   reads     mm        sample
    ........ucuuaaguacuugugccgcgggaa..............................................   1         0         seq
    .........cuuaaguacuugugccgcgggaa..............................................   3         0         seq
    ..........uuaaguacuugugccgcg..................................................   262       0         seq
    ..........uuaaguacuugugccgcgg.................................................   355       0         seq
    ..........uuaaguacuugugccgcggg................................................   438       0         seq
    ..........uuaaguacuugugccgcggga...............................................   2471      0         seq
    ..........uuaaguacuugugccgcgggaa..............................................   6542      0         seq
    ..........uuaaguacuugugccgcgggaaa.............................................   469       0         seq
    ..........uuaaguacuugugccgcgggaaag............................................   1         0         seq
    ..........uuaaguacuugugccgcgggaaagu...........................................   1         0         seq
    ...........uaaguacuugugccgcggg................................................   5         0         seq
    ...........uaaguacuugugccgcggga...............................................   28        0         seq
    ...........uaaguacuugugccgcgggaa..............................................   60        0         seq
    ...........uaaguacuugugccgcgggaaa.............................................   3         0         seq
    ............aaguacuugugccgcggg................................................   1         0         seq
    ............aaguacuugugccgcggga...............................................   3         0         seq
    ............aaguacuugugccgcgggaa..............................................   10        0         seq
    ............aaguacuugugccgcgggaaag............................................   1         0         seq
    .............aguacuugugccgcggga...............................................   1         0         seq
    .............aguacuugugccgcgggaa..............................................   3         0         seq
    ............................................uucccauggaucaaguauuu..............   4         0         seq
    ............................................uucccauggaucaaguauuua.............   4         0         seq
    ............................................uucccauggaucaaguauuuaa............   3         0         seq
    .............................................ucccauggaucaaguauuuaa............   1         0         seq
    ..............................................cccauggaucaaguauuu..............   1         0         seq
    ..............................................cccauggaucaaguauuuaa............   3         0         seq
    ..............................................cccauggaucaaguauuuaau...........   7         0         seq
    ..............................................cccauggaucaaguauuuaaua..........   52        0         seq
    .................................................auggaucaaguauuuaaua..........   1         0         seq
```
