# Supplementary material for: Identification of Conserved and Novel MicroRNAs in the Pacific Oyster Crassostrea gigas by Deep Sequencing
Source: PLoS One. 2014 Aug 19;9(8):e104371. doi: 10.1371/journal.pone.0104371 (PMC4138081; doi:10.1371/journal.pone.0104371)
Supplement: File S2 — The compressed/ZIP file archive for the predicted precursors' secondary structures and reads alignment. (ZIP) [file pone.0104371.s010.zip › second structure and reads alignment for oyster miRNAs/conserved in table S4/cgi-miR-2722.pdf]

miRBase precursor : cgi-miR-2722
Total read count : 3246
cgi-miR-2722-5p read count : 2506
cgi-miR-2722-3p read count : 738
remaining reads : 2

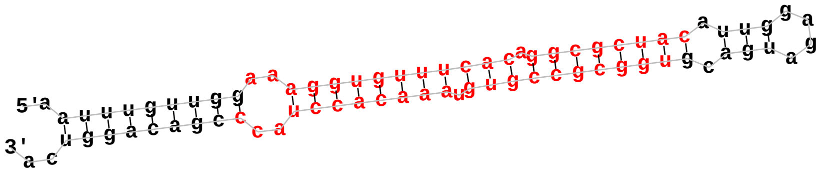

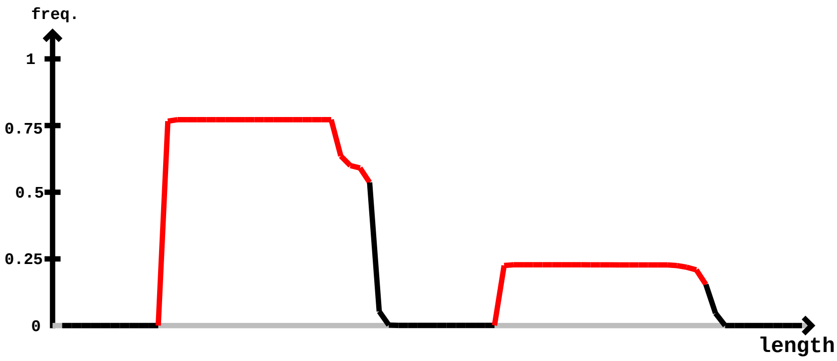


```
                                             cgi-miR-2722-3p
          cgi-miR-2722-5p
5'- aauuuguuggaaagguguuucacaggcgcuacauuggagaugacguggcgccguguaaacaccuacccgacagguca   -3'    exp
    .(((((((((..(((((((((((.((((((((.(((....))).))))))))))).))))))))..)))))))))..    reads      mm         sample
    ..........aaagguguuucacaggcg.................................................    442        0          seq
    ..........aaagguguuucacaggcgc................................................    117        0          seq
    ..........aaagguguuucacaggcgcu...............................................    29         0          seq
    ..........aaagguguuucacaggcgcua..............................................    173        0          seq
    ..........aaagguguuucacaggcgcuac.............................................    1557       0          seq
    ..........aaagguguuucacaggcgcuaca............................................    166        0          seq
    ..........aaagguguuucacaggcgcuacau...........................................    4          0          seq
    ...........aagguguuucacaggcgcua..............................................    3          0          seq
    ...........aagguguuucacaggcgcuac.............................................    13         0          seq
    ...........aagguguuucacaggcgcuaca............................................    2          0          seq
    .................................uuggagaugacguggcgccgu.......................    1          0          seq
    .................................uuggagaugacguggcgccgugua....................    1          0          seq
    .............................................uggcgccguguaaacacc..............    8          0          seq
    .............................................uggcgccguguaaacaccu.............    20         0          seq
    .............................................uggcgccguguaaacaccua............    32         0          seq
    .............................................uggcgccguguaaacaccuac...........    174        0          seq
    .............................................uggcgccguguaaacaccuacc..........    348        0          seq
    .............................................uggcgccguguaaacaccuaccc.........    147        0          seq
    ..............................................ggcgccguguaaacaccuac...........    4          0          seq
    ..............................................ggcgccguguaaacaccuacc..........    2          0          seq
    ..............................................ggcgccguguaaacaccuaccc.........    3          0          seq
```
