# Supplementary material for: Identification of Conserved and Novel MicroRNAs in the Pacific Oyster Crassostrea gigas by Deep Sequencing
Source: PLoS One. 2014 Aug 19;9(8):e104371. doi: 10.1371/journal.pone.0104371 (PMC4138081; doi:10.1371/journal.pone.0104371)
Supplement: File S2 — The compressed/ZIP file archive for the predicted precursors' secondary structures and reads alignment. (ZIP) [file pone.0104371.s010.zip › second structure and reads alignment for oyster miRNAs/conserved in table S4/cgi-miR-277.pdf]

miRBase precursor : cgi-miR-277
Total read count : 1162
cgi-miR-277-5p read count : 64
cgi-miR-277-3p read count : 1098
remaining reads : 0

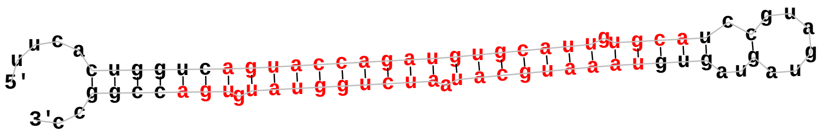

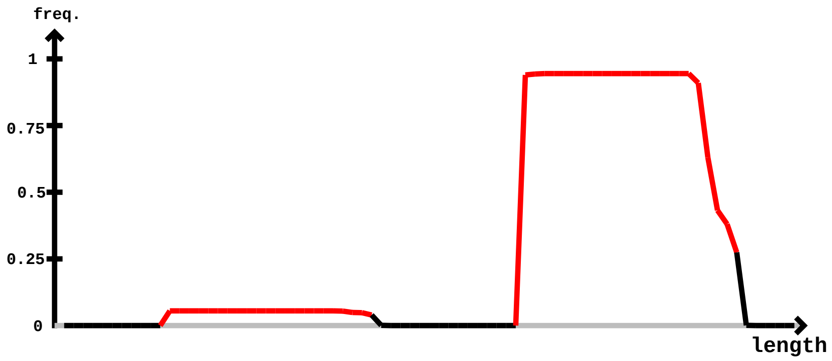


```
                 cgi-miR-277-5p
                                                     cgi-miR-277-3p
5'- uucacuggucaguaccagaugugcauugugcauccguaguaguaguguaaaaugcauaaucugguaugugaccggcc   -3'    exp
    ....(((((((((((((((((((((((.(((((.(......)..)))))))))))).))))))))).))))))).. reads      mm         sample
    ..........aguaccagaugugcauug................................................ 1          0          seq
    ..........aguaccagaugugcauugu............................................... 6          0          seq
    ..........aguaccagaugugcauugug.............................................. 1          0          seq
    ..........aguaccagaugugcauugugc............................................. 10         0          seq
    ..........aguaccagaugugcauugugca............................................ 45         0          seq
    ..........aguaccagaugugcauugugcau........................................... 1          0          seq
    ...............................................uaaaugcauaaucuggua........... 41         0          seq
    ...............................................uaaaugcauaaucugguau.......... 325        0          seq
    ...............................................uaaaugcauaaucugguaug......... 230        0          seq
    ...............................................uaaaugcauaaucugguaugu........ 59         0          seq
    ...............................................uaaaugcauaaucugguaugug....... 120        0          seq
    ...............................................uaaaugcauaaucugguauguga...... 316        0          seq
    ...............................................uaaaugcauaaucugguaugugac..... 1          0          seq
    ................................................aaaugcauaaucugguaugu........ 1          0          seq
    ................................................aaaugcauaaucugguaugug....... 1          0          seq
    ................................................aaaugcauaaucugguauguga...... 2          0          seq
    .................................................aaugcauaaucugguaugug....... 2          0          seq
```
