# Supplementary material for: Identification of Conserved and Novel MicroRNAs in the Pacific Oyster Crassostrea gigas by Deep Sequencing
Source: PLoS One. 2014 Aug 19;9(8):e104371. doi: 10.1371/journal.pone.0104371 (PMC4138081; doi:10.1371/journal.pone.0104371)
Supplement: File S2 — The compressed/ZIP file archive for the predicted precursors' secondary structures and reads alignment. (ZIP) [file pone.0104371.s010.zip › second structure and reads alignment for oyster miRNAs/conserved in table S4/cgi-miR-279.pdf]

miRBase precursor : cgi-miR-279
Total read count : 504869
cgi-miR-279-5p read count : 2833
cgi-miR-279-3p read count : 502027
remaining reads : 9

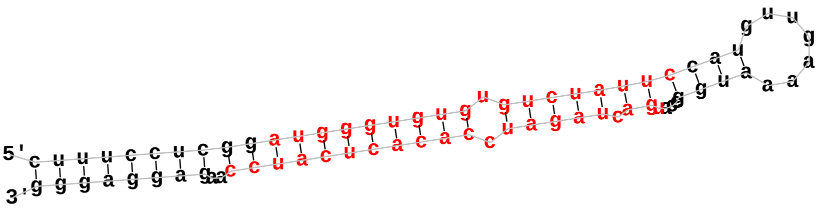

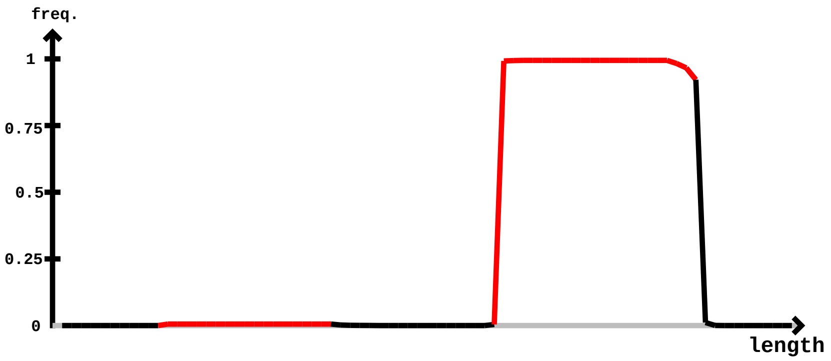


```
                    cgi-miR-279-5p
                                                  cgi-miR-279-3p
5'- cuuuccucggaugggugugugucuauuccauguugaaaauggccaugacuagauccacacucauccaagaggaggg   -3'    exp
    ((((((((((((((((((((.(((((((((((.......))))....)).))))).)))))))))))..))))))))    reads      mm         sample
    .....cucggauggguguguguc.....................................................    1          0          seq
    ......ucggaugggugugugucuau..................................................    4          0          seq
    ......ucggaugggugugugucuauu.................................................    2          0          seq
    ......ucggaugggugugugucuauuc................................................    1          0          seq
    ........gaugggugugugucuauuc.................................................    1          0          seq
    .........augggugugugucuauuc.................................................    1719       0          seq
    .........augggugugugucuauucc................................................    587        0          seq
    .........augggugugugucuauucca...............................................    212        0          seq
    .........augggugugugucuauuccau..............................................    99         0          seq
    .........augggugugugucuauuccaug.............................................    191        0          seq
    .........augggugugugucuauuccaugu............................................    17         0          seq
    .........augggugugugucuauuccauguug..........................................    1          0          seq
    ..........ugggugugugucuauucc................................................    2          0          seq
    ..........ugggugugugucuauucca...............................................    1          0          seq
    ..........ugggugugugucuauuccau..............................................    1          0          seq
    ..........ugggugugugucuauuccaug.............................................    3          0          seq
    ............................................augacuagauccacacuc..............    3          0          seq
    ............................................augacuagauccacacuca.............    50         0          seq
    ............................................augacuagauccacacucau............    46         0          seq
    ............................................augacuagauccacacucauc...........    187        0          seq
    ............................................augacuagauccacacucaucc..........    1783       0          seq
    ............................................augacuagauccacacucaucca.........    26         0          seq
    ............................................augacuagauccacacucauccaa........    7          0          seq
    .............................................ugacuagauccacacuca.............    5898       0          seq
    .............................................ugacuagauccacacucau............    8193       0          seq
    .............................................ugacuagauccacacucauc...........    22237      0          seq
    .............................................ugacuagauccacacucaucc..........    456931     0          seq
    .............................................ugacuagauccacacucaucca.........    5421       0          seq
    .............................................ugacuagauccacacucauccaa........    219        0          seq
    .............................................ugacuagauccacacucauccaag.......    1          0          seq
    .............................................ugacuagauccacacucauccaaga......    1          0          seq
    ..............................................gacuagauccacacucau............    17         0          seq
    ..............................................gacuagauccacacucauc...........    30         0          seq
    ..............................................gacuagauccacacucaucc..........    565        0          seq
```

```
          cgi-miR-279-5p
                                                   cgi-miR-279-3p
cuuuccucggaugggugugugucuauuccauguugaaaauggccaugacuagauccacacucauccaagaggaggg

..............................................gacuagauccacacucaucca.........    14    0    seq
..............................................gacuagauccacacucauccaa........    1     0    seq
...............................................acuagauccacacucauc...........    28    0    seq
...............................................acuagauccacacucaucc..........    361   0    seq
...............................................acuagauccacacucaucca.........    5     0    seq
...............................................acuagauccacacucauccaa........    1     0    seq
................................................cuagauccacacucaucca.........    2     0    seq
```
