# Supplementary material for: Identification of Conserved and Novel MicroRNAs in the Pacific Oyster Crassostrea gigas by Deep Sequencing
Source: PLoS One. 2014 Aug 19;9(8):e104371. doi: 10.1371/journal.pone.0104371 (PMC4138081; doi:10.1371/journal.pone.0104371)
Supplement: File S2 — The compressed/ZIP file archive for the predicted precursors' secondary structures and reads alignment. (ZIP) [file pone.0104371.s010.zip › second structure and reads alignment for oyster miRNAs/conserved in table S4/cgi-miR-29-1.pdf]

miRBase precursor : cgi-miR-29-1
Total read count : 53599
cgi-miR-29-1-5p read count: 794
cgi-miR-29-1-3p read count: 26364
cgi-miR-29-2-3p read count: 26364
remaining reads : 77

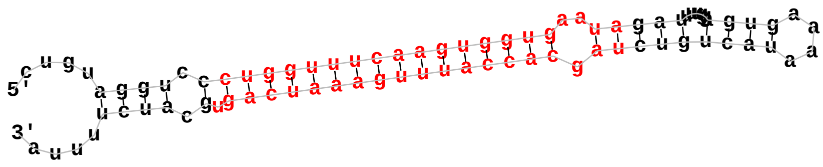

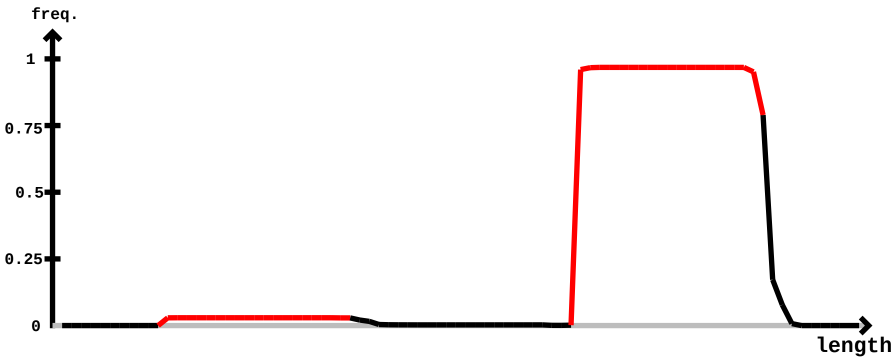


```
                 cgi-miR-29-1-5p
                                                    cgi-miR-29-1-3p
                                                    cgi-miR-29-2-3p
5'- cuguaggucccugguuucaaguggugaauagauuuuggagugaaaauacugucuagcaccauuugaaaucagugcaucuuuua  -3'   exp
    ....((((.(((((((((((((((((..(((((.....((((....))))))))).))))))))))))))))).).))))....   reads     mm         sample
    .........ccugguuucaaguggugaauag....................................................   1         0          seq
    .........ccugguuucaaguggugaauaga...................................................   2         0          seq
    ..........cugguuucaaguggugaa.......................................................   14        0          seq
    ..........cugguuucaaguggugaau......................................................   2         0          seq
    ..........cugguuucaaguggugaaua.....................................................   217       0          seq
    ..........cugguuucaaguggugaauag....................................................   138       0          seq
    ..........cugguuucaaguggugaauaga...................................................   380       0          seq
    ..........cugguuucaaguggugaauagau..................................................   22        0          seq
    ..........cugguuucaaguggugaauagauu.................................................   6         0          seq
    ..........cugguuucaaguggugaauagauuu................................................   3         0          seq
    ..........cugguuucaaguggugaauagauuuu...............................................   4         0          seq
    ...........ugguuucaaguggugaaua.....................................................   2         0          seq
    ...........ugguuucaaguggugaauag....................................................   1         0          seq
    ...........ugguuucaaguggugaauaga...................................................   3         0          seq
    ...........ugguuucaaguggugaauagau..................................................   2         0          seq
    ...........ugguuucaaguggugaauagauu.................................................   1         0          seq
    ...............................auuuuggagugaaaauac..................................   1         0          seq
    ................................uuuuggagugaaaauacu.................................   51        0          seq
    ................................uuuuggagugaaaauacug................................   5         0          seq
    ................................uuuuggagugaaaauacugu...............................   1         0          seq
    ................................uuuuggagugaaaauacuguc..............................   11        0          seq
    ................................uuuuggagugaaaauacugucu.............................   1         0          seq
    .............................................auacugucuagcaccauuugaaa...............   1         0          seq
    ..............................................uacugucuagcaccauuugaaauc.............   1         0          seq
    .................................................ugucuagcaccauuugaaau..............   1         0          seq
    ...................................................ucuagcaccauuugaaaucagu..........   2         0          seq
    ....................................................cuagcaccauuugaaauc.............   1         0          seq
    ....................................................cuagcaccauuugaaaucag...........   1         0          seq
    ....................................................cuagcaccauuugaaaucagu..........   11        0          seq
    ....................................................cuagcaccauuugaaaucagug.........   6         0          seq
    ....................................................cuagcaccauuugaaaucagugc........   6         0          seq
    .....................................................uagcaccauuugaaauca............   455       0          seq
    .....................................................uagcaccauuugaaaucag...........   4375      0          seq
```

cgi-miR-29-1-5p

cgi-miR-29-1-3p

cgi-miR-29-2-3p

cuguagguccugguuucaaguggugaauagauuuuggagugaaaauacuguc[illegible]ucagugcaucuuuua

....uagcaccauuugaaaucagu..........	16645	0	seq
....uagcaccauuugaaaucagug.........	2539	0	seq
....uagcaccauuugaaaucagugc........	1897	0	seq
....uagcaccauuugaaaucagugca.......	198	0	seq
....uagcaccauuugaaaucagugcau......	1	0	seq
....agcaccauuugaaaucag...........	37	0	seq
....agcaccauuugaaaucagu..........	130	0	seq
....agcaccauuugaaaucagug.........	17	0	seq
....agcaccauuugaaaucagugc........	11	0	seq
....gcaccauuugaaaucagu..........	27	0	seq
....gcaccauuugaaaucagug.........	4	0	seq
....gcaccauuugaaaucagugc........	1	0	seq
