# Supplementary material for: Identification of Conserved and Novel MicroRNAs in the Pacific Oyster Crassostrea gigas by Deep Sequencing
Source: PLoS One. 2014 Aug 19;9(8):e104371. doi: 10.1371/journal.pone.0104371 (PMC4138081; doi:10.1371/journal.pone.0104371)
Supplement: File S2 — The compressed/ZIP file archive for the predicted precursors' secondary structures and reads alignment. (ZIP) [file pone.0104371.s010.zip › second structure and reads alignment for oyster miRNAs/conserved in table S4/cgi-miR-29-2.pdf]

miRBase precursor : cgi-miR-29-2
Total read count : 98503
cgi-miR-29-1-3p read count: 48953
cgi-miR-29-2-5p read count: 597
cgi-miR-29-2-3p read count: 48953
remaining reads : 0

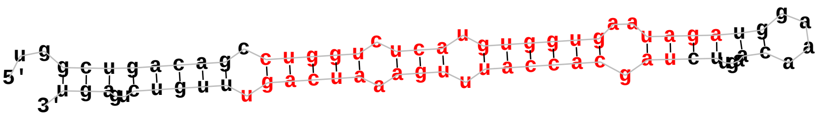

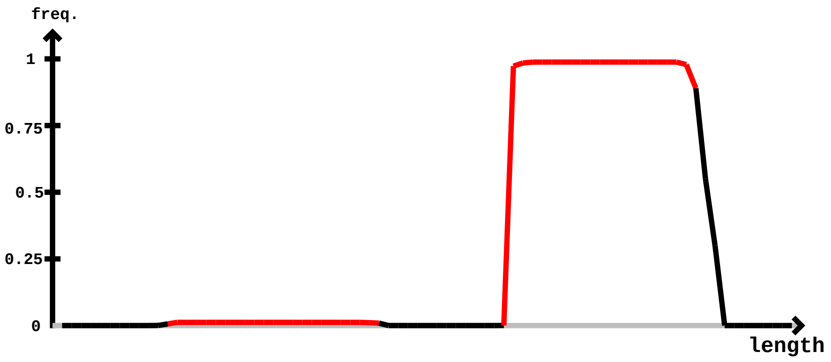


```
                                             cgi-miR-29-1-3p
                                             cgi-miR-29-2-3p
              cgi-miR-29-2-5p
5'- uggcugacagccuggucucauguggugaauagauggaaacaaguucuagcaccauuugaaaucaguuugucugagu  -3'   exp
    ...(.(((((.(((((.(((.((((((..((((((....))...)))).)))))).))).))))).))))).)...   reads   mm   sample
    .........gccuggucucaugugguga................................................   1       0    seq
    .........gccuggucucauguggugaa...............................................   2       0    seq
    .........gccuggucucauguggugaaua.............................................   4       0    seq
    .........gccuggucucauguggugaauag............................................   14      0    seq
    ..........ccuggucucauguggugaa...............................................   4       0    seq
    ..........ccuggucucauguggugaaua.............................................   19      0    seq
    ..........ccuggucucauguggugaauag............................................   38      0    seq
    ..........ccuggucucauguggugaauaga...........................................   224     0    seq
    ...........cuggucucauguggugaa...............................................   2       0    seq
    ...........cuggucucauguggugaaua.............................................   28      0    seq
    ...........cuggucucauguggugaauag............................................   32      0    seq
    ...........cuggucucauguggugaauaga...........................................   225     0    seq
    ...........cuggucucauguggugaauagaugg.......................................   1       0    seq
    ............uggucucauguggugaauaga...........................................   1       0    seq
    ............uggucucauguggugaauagaug.........................................   1       0    seq
    ............uggucucauguggugaauagauggaa......................................   1       0    seq
    ............................................ucuagcaccauuugaaaucagu..........   2       0    seq
    .............................................cuagcaccauuugaaauc.............   1       0    seq
    .............................................cuagcaccauuugaaaucag...........   1       0    seq
    .............................................cuagcaccauuugaaaucagu..........   11      0    seq
    .............................................cuagcaccauuugaaaucaguu.........   5       0    seq
    .............................................cuagcaccauuugaaaucaguuu........   2       0    seq
    ..............................................uagcaccauuugaaauca............   455     0    seq
    ..............................................uagcaccauuugaaaucag...........   4375    0    seq
    ..............................................uagcaccauuugaaaucagu..........   16645   0    seq
    ..............................................uagcaccauuugaaaucaguu.........   12243   0    seq
    ..............................................uagcaccauuugaaaucaguuu........   14457   0    seq
    ..............................................uagcaccauuugaaaucaguuug.......   3       0    seq
    ...............................................agcaccauuugaaaucag...........   37      0    seq
    ...............................................agcaccauuugaaaucagu..........   130     0    seq
    ...............................................agcaccauuugaaaucaguu.........   167     0    seq
    ...............................................agcaccauuugaaaucaguuu........   258     0    seq
    ................................................gcaccauuugaaaucagu..........   27      0    seq
```

cgi-miR-29-1-3p
cgi-miR-29-2-3p
cgi-miR-29-2-5p
uggcugacagc[illegible]gaauagauggaaacaaguucuagcaccauuugaaaucaguuugucugagu

....gcaccauuugaaaucaguu.........	49	0	seq
....gcaccauuugaaaucaguuu........	79	0	seq
....caccauuugaaaucaguuu........	2	0	seq
....accauuugaaaucaguuu........	4	0	seq
