# Supplementary material for: Identification of Conserved and Novel MicroRNAs in the Pacific Oyster Crassostrea gigas by Deep Sequencing
Source: PLoS One. 2014 Aug 19;9(8):e104371. doi: 10.1371/journal.pone.0104371 (PMC4138081; doi:10.1371/journal.pone.0104371)
Supplement: File S2 — The compressed/ZIP file archive for the predicted precursors' secondary structures and reads alignment. (ZIP) [file pone.0104371.s010.zip › second structure and reads alignment for oyster miRNAs/conserved in table S4/cgi-miR-2c.pdf]

miRBase precursor : cgi-miR-2c
Total read count : 35703
cgi-miR-2c-5p read count : 13832
cgi-miR-2c-3p read count : 21871
remaining reads : 0

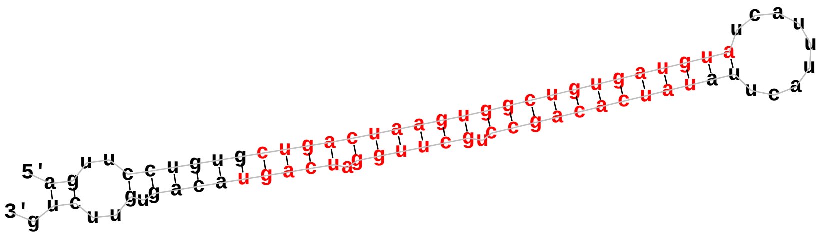

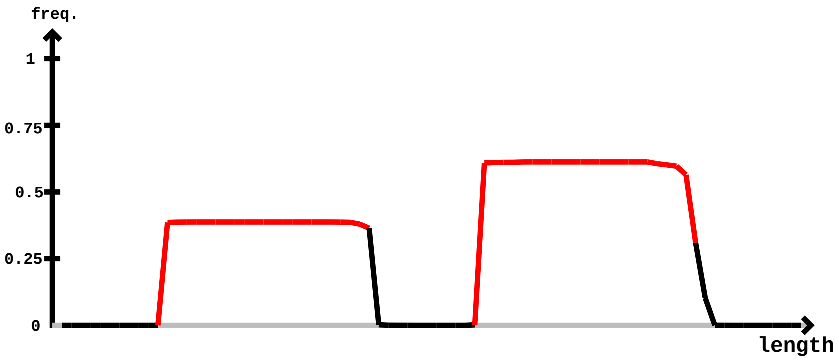


```
                                     cgi-miR-2c-3p
              cgi-miR-2c-5p
5'- aguuccugugcugacuaaguggcugugauguaucauuuacuuauaucacagccugcuuggaucaguacaguguucug   -3'    exp
    ((..((((((((((((((((((((((((((((.........)))))))))))).)))))).))))))))).)..)).    reads    mm    sample
    ..........cugacuaaguggcuguga.................................................    12       0     seq
    ..........cugacuaaguggcugugau................................................    23       0     seq
    ..........cugacuaaguggcugugaug...............................................    242      0     seq
    ..........cugacuaaguggcugugaugu..............................................    548      0     seq
    ..........cugacuaaguggcugugaugua.............................................    12885    0     seq
    ..........cugacuaaguggcugugauguau............................................    54       0     seq
    ..........cugacuaaguggcugugauguauc...........................................    5        0     seq
    ..........cugacuaaguggcugugauguauca..........................................    5        0     seq
    ..........cugacuaaguggcugugauguaucau.........................................    2        0     seq
    ...........ugacuaaguggcugugaugu..............................................    3        0     seq
    ...........ugacuaaguggcugugaugua.............................................    35       0     seq
    ...........ugacuaaguggcugugauguau............................................    3        0     seq
    ...........ugacuaaguggcugugauguauca..........................................    1        0     seq
    ............gacuaaguggcugugaug...............................................    1        0     seq
    ............gacuaaguggcugugaugua.............................................    9        0     seq
    .............acuaaguggcugugaugua.............................................    1        0     seq
    .............acuaaguggcugugauguau............................................    3        0     seq
    ..........................................auaucacagccugcuuggauc..............    1        0     seq
    ..........................................auaucacagccugcuuggaucag............    4        0     seq
    ..........................................auaucacagccugcuuggaucagu...........    16       0     seq
    ..........................................auaucacagccugcuuggaucagua..........    45       0     seq
    ...........................................uaucacagccugcuugga................    238      0     seq
    ...........................................uaucacagccugcuuggau...............    154      0     seq
    ...........................................uaucacagccugcuuggauc..............    169      0     seq
    ...........................................uaucacagccugcuuggauca.............    1148     0     seq
    ...........................................uaucacagccugcuuggaucag............    9065     0     seq
    ...........................................uaucacagccugcuuggaucagu...........    7317     0     seq
    ...........................................uaucacagccugcuuggaucagua..........    3573     0     seq
    ...........................................uaucacagccugcuuggaucaguac.........    6        0     seq
    ...........................................uaucacagccugcuuggaucaguaca........    2        0     seq
    ............................................aucacagccugcuuggau...............    1        0     seq
    ............................................aucacagccugcuuggauca.............    6        0     seq
    ............................................aucacagccugcuuggaucag............    10       0     seq
    ............................................aucacagccugcuuggaucagu...........    11       0     seq
```

```
                                           cgi-miR-2c-3p
          cgi-miR-2c-5p
aguuccugugcugacuaaguggcugugauguaucauuuacuuauaucacagccugcuuggaucaguacaguguucug

............................................aucacagccugcuuggaucagua..........   13   0   seq
.............................................ucacagccugcuuggauc..............   1    0   seq
.............................................ucacagccugcuuggauca.............   7    0   seq
.............................................ucacagccugcuuggaucag............   3    0   seq
.............................................ucacagccugcuuggaucagu...........   19   0   seq
.............................................ucacagccugcuuggaucagua..........   14   0   seq
..............................................cacagccugcuuggaucag............   1    0   seq
..............................................cacagccugcuuggaucagu...........   3    0   seq
..............................................cacagccugcuuggaucagua..........   3    0   seq
...............................................acagccugcuuggaucag............   12   0   seq
...............................................acagccugcuuggaucagu...........   18   0   seq
...............................................acagccugcuuggaucagua..........   9    0   seq
................................................cagccugcuuggaucagu...........   1    0   seq
................................................cagccugcuuggaucagua..........   1    0   seq
```
