# Supplementary material for: Identification of Conserved and Novel MicroRNAs in the Pacific Oyster Crassostrea gigas by Deep Sequencing
Source: PLoS One. 2014 Aug 19;9(8):e104371. doi: 10.1371/journal.pone.0104371 (PMC4138081; doi:10.1371/journal.pone.0104371)
Supplement: File S2 — The compressed/ZIP file archive for the predicted precursors' secondary structures and reads alignment. (ZIP) [file pone.0104371.s010.zip › second structure and reads alignment for oyster miRNAs/conserved in table S4/cgi-miR-2d-1.pdf]

miRBase precursor : cgi-miR-2d-1
Total read count : 97254
cgi-miR-2d-1-5p read count: 2828
cgi-miR-2d-1-3p read count: 47213
cgi-miR-2d-2-3p read count: 47213
remaining reads : 0

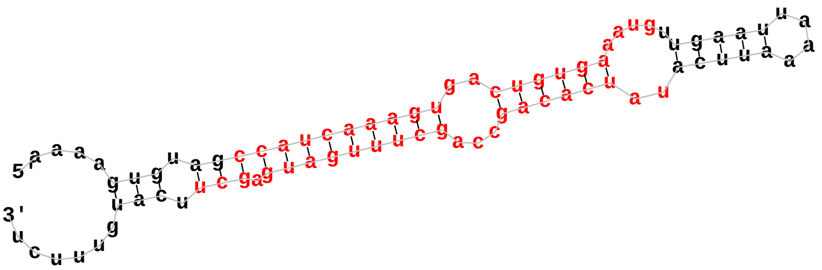

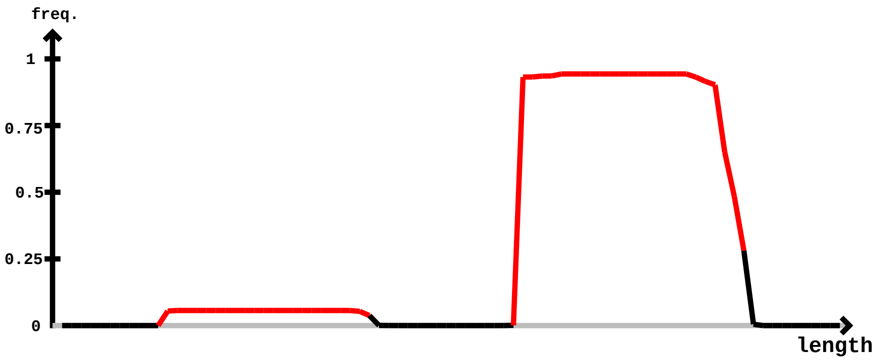

cgi-miR-2d-1-5p

cgi-miR-2d-1-3p

cgi-miR-2d-2-3p

```
5'- aaaaguguagccaucaaagugacugugaaauguugaauuaaaauucauaucacagccagcuuugaugagcuucauguuucu   -3'    exp
    ....(((.((((((((((((..((((((.....(((((....)))))..))))))...))))))))).))).)))......    reads      mm         sample
    ..........ccaucaaagugacuguga.....................................................    3          0          seq
    ..........ccaucaaagugacugugaa....................................................    4          0          seq
    ..........ccaucaaagugacugugaaa...................................................    146        0          seq
    ..........ccaucaaagugacugugaaau..................................................    766        0          seq
    ..........ccaucaaagugacugugaaaug.................................................    1784       0          seq
    ..........ccaucaaagugacugugaaaugu................................................    13         0          seq
    ..........ccaucaaagugacugugaaauguu...............................................    9          0          seq
    ..........ccaucaaagugacugugaaauguug..............................................    1          0          seq
    ..........ccaucaaagugacugugaaauguuga.............................................    2          0          seq
    ...........caucaaagugacugugaaa...................................................    6          0          seq
    ...........caucaaagugacugugaaau..................................................    27         0          seq
    ...........caucaaagugacugugaaaug.................................................    64         0          seq
    ............aucaaagugacugugaaau..................................................    1          0          seq
    ............aucaaagugacugugaaaug.................................................    1          0          seq
    .............ucaaagugacugugaaaug.................................................    1          0          seq
    .............................................cauaucacagccagcuuugaug..............    1          0          seq
    .............................................cauaucacagccagcuuugauga.............    5          0          seq
    .............................................cauaucacagccagcuuugaugagcu..........    1          0          seq
    ..............................................auaucacagccagcuuugau...............    1          0          seq
    ..............................................auaucacagccagcuuugaug..............    4          0          seq
    ..............................................auaucacagccagcuuugauga.............    4          0          seq
    ..............................................auaucacagccagcuuugaugag............    4          0          seq
    ..............................................auaucacagccagcuuugaugagc...........    6          0          seq
    ..............................................auaucacagccagcuuugaugagcu..........    17         0          seq
    ...............................................uaucacagccagcuuuga................    594        0          seq
    ...............................................uaucacagccagcuuugau...............    808        0          seq
    ...............................................uaucacagccagcuuugaug..............    630        0          seq
    ...............................................uaucacagccagcuuugauga.............    12492      0          seq
    ...............................................uaucacagccagcuuugaugag............    8117       0          seq
    ...............................................uaucacagccagcuuugaugagc...........    10121      0          seq
    ...............................................uaucacagccagcuuugaugagcu..........    13592      0          seq
    ...............................................uaucacagccagcuuugaugagcuu.........    236        0          seq
    ................................................aucacagccagcuuugau...............    3          0          seq
```

cgi-miR-2d-1-5p

cgi-miR-2d-1-3p

aaaaguguagccaucaaagugacugugaaauguugaauuaaaauuca[illegible]ugaugagcuucauguuucu

....aucacagccagcuuugaugag............	3	0	seq
....aucacagccagcuuugaugagc...........	4	0	seq
....aucacagccagcuuugaugagcu..........	5	0	seq
....aucacagccagcuuugaugagcuu.........	2	0	seq
....ucacagccagcuuugaug..............	7	0	seq
....ucacagccagcuuugauga.............	72	0	seq
....ucacagccagcuuugaugag............	18	0	seq
....ucacagccagcuuugaugagc...........	22	0	seq
....ucacagccagcuuugaugagcu..........	55	0	seq
....ucacagccagcuuugaugagcuu.........	1	0	seq
....cacagccagcuuugaugag............	1	0	seq
....cacagccagcuuugaugagc...........	1	0	seq
....cacagccagcuuugaugagcu..........	9	0	seq
....acagccagcuuugaugag............	179	0	seq
....acagccagcuuugaugagc...........	65	0	seq
....acagccagcuuugaugagcu..........	126	0	seq
....acagccagcuuugaugagcuu.........	5	0	seq
....cagccagcuuugaugagc...........	1	0	seq
....agccagcuuugaugagcu..........	1	0	seq
