# Supplementary material for: Identification of Conserved and Novel MicroRNAs in the Pacific Oyster Crassostrea gigas by Deep Sequencing
Source: PLoS One. 2014 Aug 19;9(8):e104371. doi: 10.1371/journal.pone.0104371 (PMC4138081; doi:10.1371/journal.pone.0104371)
Supplement: File S2 — The compressed/ZIP file archive for the predicted precursors' secondary structures and reads alignment. (ZIP) [file pone.0104371.s010.zip › second structure and reads alignment for oyster miRNAs/conserved in table S4/cgi-miR-2d-2.pdf]

miRBase precursor : cgi-miR-2d-2
Total read count : 96725
cgi-miR-2d-1-3p read count: 47172
cgi-miR-2d-2-5p read count: 2376
cgi-miR-2d-2-3p read count: 47172
remaining reads : 5

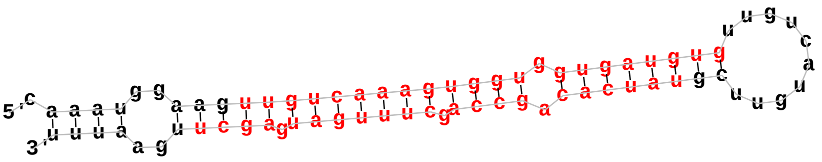

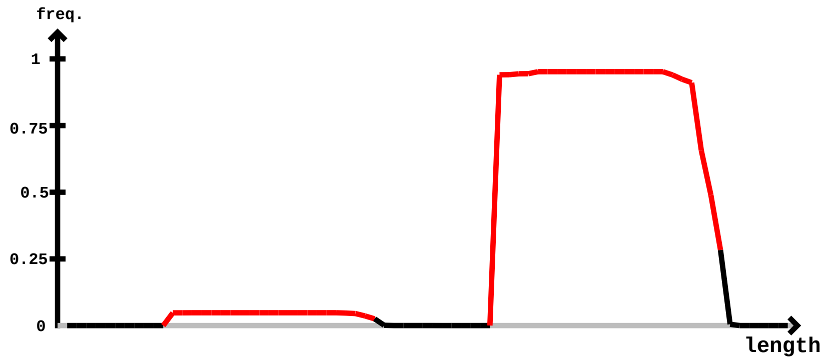


```
                                            cgi-miR-2d-1-3p
                                            cgi-miR-2d-2-3p
          cgi-miR-2d-2-5p
5'- caaauggaaguugucaaagugguggugaugugguugucauguucguaucacagccagcuuugaugagcuugaauuu   -3'    exp
    ..(((..((((((((((((((((.((((((((..........)))))))).)))).))))))).)))))..))).    reads      mm         sample
    ..........uugucaaaguggugguga...............................................    55         0          seq
    ..........uugucaaagugguggugau..............................................    94         0          seq
    ..........uugucaaagugguggugaug.............................................    442        0          seq
    ..........uugucaaagugguggugaugu............................................    536        0          seq
    ..........uugucaaagugguggugaugug...........................................    1189       0          seq
    ..........uugucaaagugguggugaugugu..........................................    42         0          seq
    ..........uugucaaagugguggugauguguu.........................................    2          0          seq
    ..........uugucaaagugguggugauguguuguca.....................................    4          0          seq
    ...........ugucaaagugguggugaugug...........................................    5          0          seq
    ............gucaaagugguggugaug.............................................    2          0          seq
    ............gucaaagugguggugaugug...........................................    8          0          seq
    ............gucaaagugguggugaugugu..........................................    1          0          seq
    .................................ugucauguucguaucacagcca....................    1          0          seq
    ...........................................guaucacagccagcuuugauga..........    1          0          seq
    ...........................................guaucacagccagcuuugaugagcu.......    1          0          seq
    ............................................uaucacagccagcuuuga.............    594        0          seq
    ............................................uaucacagccagcuuugau............    808        0          seq
    ............................................uaucacagccagcuuugaug...........    630        0          seq
    ............................................uaucacagccagcuuugauga..........    12492      0          seq
    ............................................uaucacagccagcuuugaugag.........    8117       0          seq
    ............................................uaucacagccagcuuugaugagc........    10121      0          seq
    ............................................uaucacagccagcuuugaugagcu.......    13592      0          seq
    ............................................uaucacagccagcuuugaugagcuu......    236        0          seq
    .............................................aucacagccagcuuugau............    3          0          seq
    .............................................aucacagccagcuuugaugag.........    3          0          seq
    .............................................aucacagccagcuuugaugagc........    4          0          seq
    .............................................aucacagccagcuuugaugagcu.......    5          0          seq
    .............................................aucacagccagcuuugaugagcuu......    2          0          seq
    ..............................................ucacagccagcuuugaug...........    7          0          seq
    ..............................................ucacagccagcuuugauga..........    72         0          seq
    ..............................................ucacagccagcuuugaugag.........    18         0          seq
    ..............................................ucacagccagcuuugaugagc........    22         0          seq
    ..............................................ucacagccagcuuugaugagcu.......    55         0          seq
```

```
                                            cgi-miR-2d-1-3p
                                            cgi-miR-2d-2-3p
caaauggaag[illegible]ugaugug uugucauguucguaucacagccagcuuugaugagcuugaauuu
          cgi-miR-2d-2-5p
```

....ucacagccagcuuugaugagcuu......	1	0	seq
....cacagccagcuuugaugag.........	1	0	seq
....cacagccagcuuugaugagc........	1	0	seq
....cacagccagcuuugaugagcu.......	9	0	seq
....acagccagcuuugaugag.........	179	0	seq
....acagccagcuuugaugagc........	65	0	seq
....acagccagcuuugaugagcu.......	126	0	seq
....acagccagcuuugaugagcuu......	5	0	seq
....cagccagcuuugaugagc........	1	0	seq
....agccagcuuugaugagcu.......	1	0	seq
