# Supplementary material for: Identification of Conserved and Novel MicroRNAs in the Pacific Oyster Crassostrea gigas by Deep Sequencing
Source: PLoS One. 2014 Aug 19;9(8):e104371. doi: 10.1371/journal.pone.0104371 (PMC4138081; doi:10.1371/journal.pone.0104371)
Supplement: File S2 — The compressed/ZIP file archive for the predicted precursors' secondary structures and reads alignment. (ZIP) [file pone.0104371.s010.zip › second structure and reads alignment for oyster miRNAs/conserved in table S4/cgi-miR-2e.pdf]

miRBase precursor : cgi-miR-2e
Total read count : 11833
cgi-miR-2e-5p read count : 4
cgi-miR-2e-3p read count : 11800
remaining reads : 29

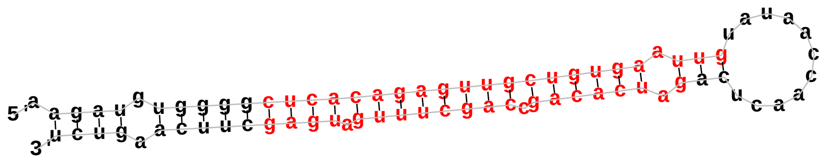

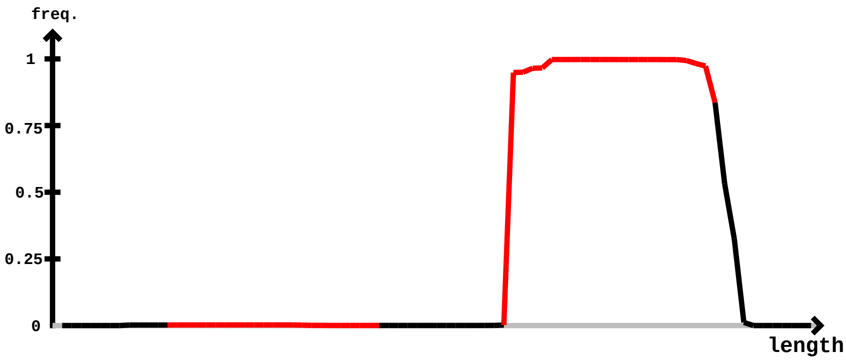


```
                                                          cgi-miR-2e-3p
              cgi-miR-2e-5p
5'- aagaugugggggcucacagaguugcugugaauuguauaaccaacucagaucacagccagcuuugaugagcuucaagucu   -3'    exp
    .((((.(((((((((((((((((((((((.(((...........))).))))))).)))))))).))))))))).))))   reads   mm   sample
    ......uggggcucacagaguugc......................................................   8       0    seq
    ......uggggcucacagaguugcu.....................................................   12      0    seq
    ......uggggcucacagaguugcug....................................................   1       0    seq
    ......uggggcucacagaguugcugu...................................................   3       0    seq
    ...........cucacagaguugcugugaauug.............................................   3       0    seq
    ..............acagaguugcugugaauugu............................................   1       0    seq
    .................................uauaaccaacucagaucacagcca.....................   1       0    seq
    .................................uauaaccaacucagaucacagccag....................   1       0    seq
    .................................uauaaccaacucagaucacagccagcuuu................   2       0    seq
    ...........................................ucagaucacagccagcuuugaugagcu........   1       0    seq
    ............................................cagaucacagccagcuuugaugag..........   1       0    seq
    .............................................agaucacagccagcuuugauga...........   3       0    seq
    .............................................agaucacagccagcuuugaugag..........   2       0    seq
    .............................................agaucacagccagcuuugaugagc.........   4       0    seq
    .............................................agaucacagccagcuuugaugagcu........   9       0    seq
    ..............................................gaucacagccagcuuuga..............   39      0    seq
    ..............................................gaucacagccagcuuugau.............   131     0    seq
    ..............................................gaucacagccagcuuugaug............   104     0    seq
    ..............................................gaucacagccagcuuugauga...........   1550    0    seq
    ..............................................gaucacagccagcuuugaugag..........   3398    0    seq
    ..............................................gaucacagccagcuuugaugagc.........   2341    0    seq
    ..............................................gaucacagccagcuuugaugagcu........   3507    0    seq
    ..............................................gaucacagccagcuuugaugagcuu.......   131     0    seq
    ...............................................aucacagccagcuuugau.............   3       0    seq
    ...............................................aucacagccagcuuugaugag..........   3       0    seq
    ...............................................aucacagccagcuuugaugagc.........   4       0    seq
    ...............................................aucacagccagcuuugaugagcu........   5       0    seq
    ...............................................aucacagccagcuuugaugagcuu.......   2       0    seq
    ................................................ucacagccagcuuugaug............   7       0    seq
    ................................................ucacagccagcuuugauga...........   72      0    seq
    ................................................ucacagccagcuuugaugag..........   18      0    seq
    ................................................ucacagccagcuuugaugagc.........   22      0    seq
    ................................................ucacagccagcuuugaugagcu........   55      0    seq
    ................................................ucacagccagcuuugaugagcuu.......   1       0    seq
```

```
                                              cgi-miR-2e-3p
           cgi-miR-2e-5p
aagauguggggcucacagaguugcugugaauuguauaaccaacucagaucacagccagcuuugaugagcuucaagucu

.................................................cacagccagcuuugaugag..........    1      0      seq
.................................................cacagccagcuuugaugagc.........    1      0      seq
.................................................cacagccagcuuugaugagcu........    9      0      seq
..................................................acagccagcuuugaugag..........    179    0      seq
..................................................acagccagcuuugaugagc.........    65     0      seq
..................................................acagccagcuuugaugagcu........    126    0      seq
..................................................acagccagcuuugaugagcuu.......    5      0      seq
...................................................cagccagcuuugaugagc.........    1      0      seq
....................................................agccagcuuugaugagcu........    1      0      seq
```
