# Supplementary material for: Identification of Conserved and Novel MicroRNAs in the Pacific Oyster Crassostrea gigas by Deep Sequencing
Source: PLoS One. 2014 Aug 19;9(8):e104371. doi: 10.1371/journal.pone.0104371 (PMC4138081; doi:10.1371/journal.pone.0104371)
Supplement: File S2 — The compressed/ZIP file archive for the predicted precursors' secondary structures and reads alignment. (ZIP) [file pone.0104371.s010.zip › second structure and reads alignment for oyster miRNAs/conserved in table S4/cgi-miR-2f.pdf]

miRBase precursor : cgi-miR-2f
Total read count : 14270
cgi-miR-2f-5p read count : 140
cgi-miR-2f-3p read count : 14130
remaining reads : 0

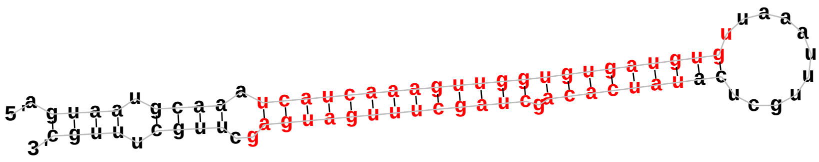

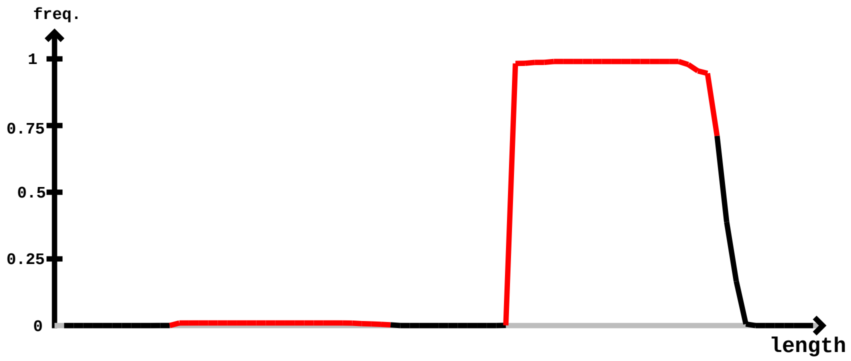


```
                    cgi-miR-2f-5p
                                                cgi-miR-2f-3p
5'- aguaaugcaaaucaucaaaguuggugugauguguuaaauuugcucauaucacagcuagcuuugaugagcuugcuuugc  -3'   exp
    .((((.((((.((((((((((((((((((((((...........))))))))).)))))))))))))..)))).))))   reads   mm   sample
    .........aaucaucaaaguuggugug..................................................   1       0    seq
    .........aaucaucaaaguuggugugau................................................   1       0    seq
    .........aaucaucaaaguuggugugaug...............................................   1       0    seq
    ...........ucaucaaaguuggugugа.................................................   8       0    seq
    ...........ucaucaaaguuggugugau................................................   27      0    seq
    ...........ucaucaaaguuggugugaug...............................................   15      0    seq
    ...........ucaucaaaguuggugugaugu..............................................   22      0    seq
    ...........ucaucaaaguuggugugaugug.............................................   23      0    seq
    ...........ucaucaaaguuggugugaugugu............................................   37      0    seq
    ...........ucaucaaaguuggugugauguguu...........................................   2       0    seq
    ............caucaaaguuggugugaugug.............................................   2       0    seq
    .............aucaaaguuggugugaugug.............................................   1       0    seq
    .............................................auaucacagcuagcuuugaugag..........   4       0    seq
    .............................................auaucacagcuagcuuugaugagc.........   2       0    seq
    .............................................auaucacagcuagcuuugaugagcu........   5       0    seq
    .............................................auaucacagcuagcuuugaugagcuu.......   1       0    seq
    ..............................................uaucacagcuagcuuuga..............   162     0    seq
    ..............................................uaucacagcuagcuuugau.............   343     0    seq
    ..............................................uaucacagcuagcuuugaug............   125     0    seq
    ..............................................uaucacagcuagcuuugauga...........   3334    0    seq
    ..............................................uaucacagcuagcuuugaugag..........   4557    0    seq
    ..............................................uaucacagcuagcuuugaugagc.........   3154    0    seq
    ..............................................uaucacagcuagcuuugaugagcu........   2270    0    seq
    ..............................................uaucacagcuagcuuugaugagcuu.......   69      0    seq
    ...............................................aucacagcuagcuuugauga...........   1       0    seq
    ...............................................aucacagcuagcuuugaugag..........   5       0    seq
    ...............................................aucacagcuagcuuugaugagcu........   2       0    seq
    ...............................................aucacagcuagcuuugaugagcuu.......   1       0    seq
    ................................................ucacagcuagcuuugaug............   4       0    seq
    ................................................ucacagcuagcuuugauga...........   13      0    seq
    ................................................ucacagcuagcuuugaugag..........   13      0    seq
    ................................................ucacagcuagcuuugaugagc.........   2       0    seq
    ................................................ucacagcuagcuuugaugagcu........   3       0    seq
    ................................................ucacagcuagcuuugaugagcuu.......   10      0    seq
```

```
          cgi-miR-2f-5p
                                   cgi-miR-2f-3p
aguaaugcaaaucaucaaaguugguguga ugugu uaaauuugcucauaucacagcuagcuuugaugagcuugcuuugc
```

```
................................................cacagcuagcuuugaugag..........    1          0          seq
................................................cacagcuagcuuugaugagc.........    1          0          seq
................................................cacagcuagcuuugaugagcu........    1          0          seq
.................................................acagcuagcuuugaugag..........    24         0          seq
.................................................acagcuagcuuugaugagc.........    9          0          seq
.................................................acagcuagcuuugaugagcu........    13         0          seq
.................................................acagcuagcuuugaugagcuu.......    1          0          seq
```
