# Supplementary material for: Identification of Conserved and Novel MicroRNAs in the Pacific Oyster Crassostrea gigas by Deep Sequencing
Source: PLoS One. 2014 Aug 19;9(8):e104371. doi: 10.1371/journal.pone.0104371 (PMC4138081; doi:10.1371/journal.pone.0104371)
Supplement: File S2 — The compressed/ZIP file archive for the predicted precursors' secondary structures and reads alignment. (ZIP) [file pone.0104371.s010.zip › second structure and reads alignment for oyster miRNAs/conserved in table S4/cgi-miR-2g.pdf]

miRBase precursor : cgi-miR-2g
Total read count : 156601
cgi-miR-2g-5p read count : 584
cgi-miR-2g-3p read count : 156016
remaining reads : 1

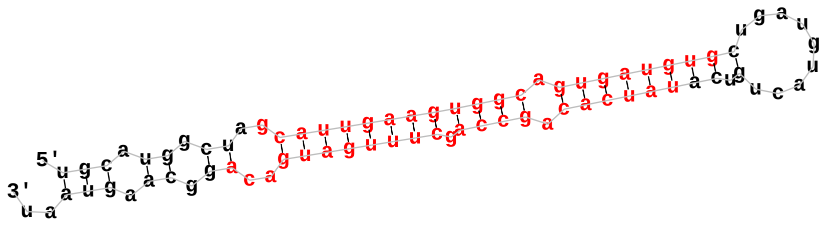

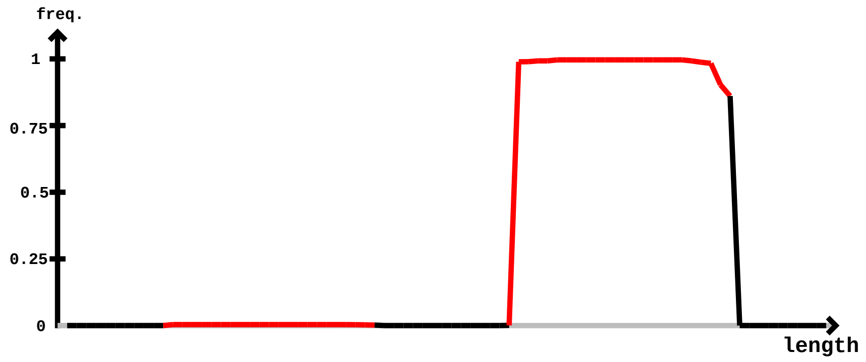

cgi-miR-2g-5p

cgi-miR-2g-3p

```
5'- ugcauggcuagcauugaaguggcagugaugugcugauguacugucauaucacagccagcuuugaugacaggcaaguaau   -3'    exp
    (((.((.((..((((((((((((.(((((((((.........).)))))))).)))).))))))))..)).)).)))..    reads      mm         sample
    ....uggcuagcauugaaguggcagu.....................................................    1          0          seq
    .........agcauugaaguggcagugaugu................................................    1          0          seq
    .........agcauugaaguggcagugaugug...............................................    1          0          seq
    ..........gcauugaaguggcaguga...................................................    20         0          seq
    ..........gcauugaaguggcagugau..................................................    50         0          seq
    ..........gcauugaaguggcagugaug.................................................    92         0          seq
    ..........gcauugaaguggcagugaugu................................................    142        0          seq
    ..........gcauugaaguggcagugaugug...............................................    275        0          seq
    ..........gcauugaaguggcagugaugugcuga...........................................    2          0          seq
    ..........gcauugaaguggcagugaugugcugau..........................................    1          0          seq
    ............................................cauaucacagccagcuuugaug.............    1          0          seq
    ............................................cauaucacagccagcuuugauga............    5          0          seq
    .............................................auaucacagccagcuuugau..............    1          0          seq
    .............................................auaucacagccagcuuugaug.............    4          0          seq
    .............................................auaucacagccagcuuugauga............    4          0          seq
    .............................................auaucacagccagcuuugaugac...........    37         0          seq
    .............................................auaucacagccagcuuugaugaca..........    53         0          seq
    ..............................................uaucacagccagcuuuga...............    594        0          seq
    ..............................................uaucacagccagcuuugau..............    808        0          seq
    ..............................................uaucacagccagcuuugaug.............    630        0          seq
    ..............................................uaucacagccagcuuugauga............    12492      0          seq
    ..............................................uaucacagccagcuuugaugac...........    6525       0          seq
    ..............................................uaucacagccagcuuugaugaca..........    133713     0          seq
    ..............................................uaucacagccagcuuugaugacag.........    9          0          seq
    ..............................................uaucacagccagcuuugaugacagg........    1          0          seq
    ...............................................aucacagccagcuuugau..............    3          0          seq
    ...............................................aucacagccagcuuugaugac...........    2          0          seq
    ...............................................aucacagccagcuuugaugaca..........    67         0          seq
    ................................................ucacagccagcuuugaug.............    7          0          seq
    ................................................ucacagccagcuuugauga............    72         0          seq
    ................................................ucacagccagcuuugaugac...........    24         0          seq
    ................................................ucacagccagcuuugaugaca..........    368        0          seq
    .................................................cacagccagcuuugaugaca..........    8          0          seq
    ..................................................acagccagcuuugaugac...........    28         0          seq
```

```
          cgi-miR-2g-5p
                                    cgi-miR-2g-3p
ugcauggcuagcauugaaguggcagugaugugcugauguacugucauaucacagccagcuuugaugacaggcaaguaau

..................................................acagccagcuuugaugaca..........    554        0          seq
...................................................cagccagcuuugaugaca..........    6          0          seq
```
