# Supplementary material for: Identification of Conserved and Novel MicroRNAs in the Pacific Oyster Crassostrea gigas by Deep Sequencing
Source: PLoS One. 2014 Aug 19;9(8):e104371. doi: 10.1371/journal.pone.0104371 (PMC4138081; doi:10.1371/journal.pone.0104371)
Supplement: File S2 — The compressed/ZIP file archive for the predicted precursors' secondary structures and reads alignment. (ZIP) [file pone.0104371.s010.zip › second structure and reads alignment for oyster miRNAs/conserved in table S4/cgi-miR-307.pdf]

miRBase precursor : cgi-miR-307
Total read count : 4573
cgi-miR-307-5p read count : 590
cgi-miR-307-3p read count : 3983
remaining reads : 0

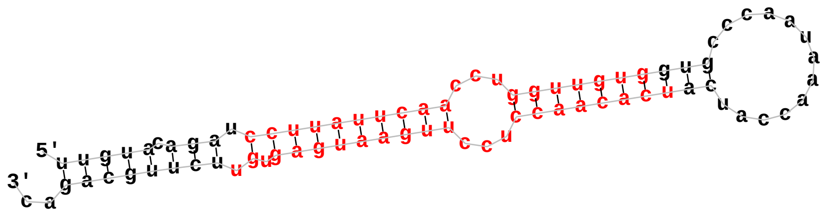

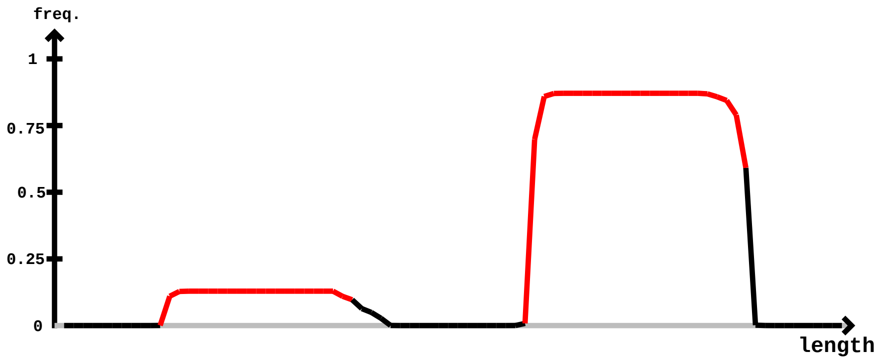


```
                                                cgi-miR-307-3p
          cgi-miR-307-5p
5'- uuguacagauccuuauucaaccugguuguggugcccaauaaaccaucaucacaaccuccuugaaugagugugucuugcagac   -3'    exp
    (((((.(((.((((((((((...((((((((((.............))))))))))...))))))))).).))))))))..     reads      mm         sample
    .........uccuuauucaaccugguugu....................................................     1          0          seq
    .........uccuuauucaaccugguugugg..................................................     1          0          seq
    ..........ccuuauucaaccugguug.....................................................     89         0          seq
    ..........ccuuauucaaccugguugu....................................................     54         0          seq
    ..........ccuuauucaaccugguugug...................................................     126        0          seq
    ..........ccuuauucaaccugguugugg..................................................     58         0          seq
    ..........ccuuauucaaccugguuguggu.................................................     96         0          seq
    ..........ccuuauucaaccugguuguggug................................................     78         0          seq
    ...........cuuauucaaccugguugu....................................................     4          0          seq
    ...........cuuauucaaccugguugug...................................................     25         0          seq
    ...........cuuauucaaccugguugugg..................................................     7          0          seq
    ...........cuuauucaaccugguuguggu.................................................     2          0          seq
    ...........cuuauucaaccugguuguggug................................................     44         0          seq
    ............uuauucaaccugguuguggu.................................................     2          0          seq
    ............uuauucaaccugguuguggug................................................     1          0          seq
    ............uuauucaaccugguuguggugc...............................................     2          0          seq
    ..............................................caucacaaccuccuugaaugagugu..........     1          0          seq
    ...............................................aucacaaccuccuugaaugagug...........     3          0          seq
    ...............................................aucacaaccuccuugaaugagugu..........     33         0          seq
    ................................................ucacaaccuccuugaaug...............     10         0          seq
    ................................................ucacaaccuccuugaauga..............     42         0          seq
    ................................................ucacaaccuccuugaaugag.............     48         0          seq
    ................................................ucacaaccuccuugaaugagu............     123        0          seq
    ................................................ucacaaccuccuugaaugagug...........     704        0          seq
    ................................................ucacaaccuccuugaaugagugu..........     2227       0          seq
    ................................................ucacaaccuccuugaaugaguguu.........     3          0          seq
    .................................................cacaaccuccuugaauga..............     8          0          seq
    .................................................cacaaccuccuugaaugag.............     9          0          seq
    .................................................cacaaccuccuugaaugagu............     122        0          seq
    .................................................cacaaccuccuugaaugagug...........     189        0          seq
    .................................................cacaaccuccuugaaugagugu..........     403        0          seq
    .................................................cacaaccuccuugaaugaguguu.........     3          0          seq
    ..................................................acaaccuccuugaaugag.............     6          0          seq
    ..................................................acaaccuccuugaaugagu............     4          0          seq
```

cgi-miR-307-3p

cgi-miR-307-5p

```
uuguacagauccuuauucaaccugguuguggugcccaauaaaccaucaucacaaccuccuugaaugaguguucuugcagac

..................................................acaaccuccuugaaugagug...........	10	0	seq
..................................................acaaccuccuugaaugagugu..........	31	0	seq
..................................................acaaccuccuugaaugaguguuc........	1	0	seq
...................................................caaccuccuugaaugagug...........	2	0	seq
...................................................caaccuccuugaaugagugu..........	1	0	seq
```
