# Supplementary material for: Identification of Conserved and Novel MicroRNAs in the Pacific Oyster Crassostrea gigas by Deep Sequencing
Source: PLoS One. 2014 Aug 19;9(8):e104371. doi: 10.1371/journal.pone.0104371 (PMC4138081; doi:10.1371/journal.pone.0104371)
Supplement: File S2 — The compressed/ZIP file archive for the predicted precursors' secondary structures and reads alignment. (ZIP) [file pone.0104371.s010.zip › second structure and reads alignment for oyster miRNAs/conserved in table S4/cgi-miR-315.pdf]

miRBase precursor	: cgi-miR-315
Total read count	: 143465
cgi-miR-315-5p read count	: 143381
cgi-miR-315-3p read count	: 72
remaining reads	: 12

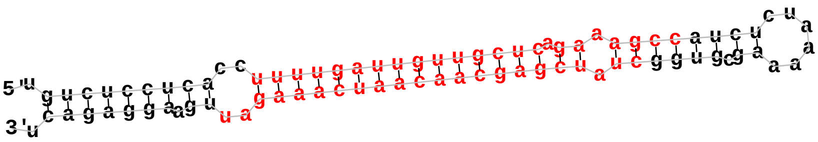

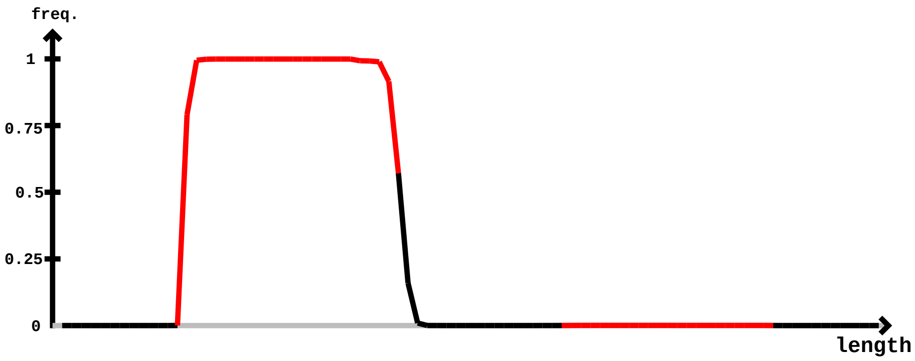


```
                 cgi-miR-315-5p
                                         cgi-miR-315-3p
5'- ugucuccucaccuuuugauuguugcucagaaagccaucucuaaaaagcguggcuaucgagcaacaaucaaagauugaaggagacu   -3'    exp
    .(((((((((..(((((((((((((((.((.((((((((......)).)))))).)))))))))))))))))..)).))))))).    reads      mm          sample
    ....uccucaccuuuugauuguugcu...........................................................    1          0           seq
    ..........ccuuuugauuguugcucagaaag....................................................    1          0           seq
    ...........cuuuugauuguugcucag........................................................    4          0           seq
    ...........cuuuugauuguugcucagaaagc...................................................    27         0           seq
    ...........cuuuugauuguugcucagaaagcc..................................................    8          0           seq
    ............uuuugauuguugcucaga.......................................................    920        0           seq
    ............uuuugauuguugcucagaa......................................................    108        0           seq
    ............uuuugauuguugcucagaaa.....................................................    410        0           seq
    ............uuuugauuguugcucagaaag....................................................    9424       0           seq
    ............uuuugauuguugcucagaaagc...................................................    46883      0           seq
    ............uuuugauuguugcucagaaagcc..................................................    53816      0           seq
    ............uuuugauuguugcucagaaagcca.................................................    1843       0           seq
    ............uuuugauuguugcucagaaagccau................................................    92         0           seq
    .............uuugauuguugcucagaa......................................................    11         0           seq
    .............uuugauuguugcucagaaa.....................................................    58         0           seq
    .............uuugauuguugcucagaaag....................................................    1039       0           seq
    .............uuugauuguugcucagaaagc...................................................    2408       0           seq
    .............uuugauuguugcucagaaagcc..................................................    5023       0           seq
    .............uuugauuguugcucagaaagcca.................................................    19466      0           seq
    .............uuugauuguugcucagaaagccau................................................    1206       0           seq
    .............uuugauuguugcucagaaagccauc...............................................    2          0           seq
    ..............uugauuguugcucagaaag....................................................    16         0           seq
    ..............uugauuguugcucagaaagc...................................................    89         0           seq
    ..............uugauuguugcucagaaagcc..................................................    201        0           seq
    ..............uugauuguugcucagaaagcca.................................................    198        0           seq
    ..............uugauuguugcucagaaagccau................................................    25         0           seq
    ...............ugauuguugcucagaaag....................................................    3          0           seq
    ...............ugauuguugcucagaaagc...................................................    8          0           seq
    ...............ugauuguugcucagaaagcc..................................................    25         0           seq
    ...............ugauuguugcucagaaagcca.................................................    28         0           seq
    ...............ugauuguugcucagaaagccau................................................    7          0           seq
    ...............ugauuguugcucagaaagccauc...............................................    25         0           seq
    ...............ugauuguugcucagaaagccaucu..............................................    1          0           seq
    ................gauuguugcucagaaagc...................................................    2          0           seq
```

```
            cgi-miR-315-5p
                                                    cgi-miR-315-3p
ugucuccucaccuuuugauuguugcucagaaagccaucucuaaaaagcguggcuaucgagcaacaaucaaagauugaaggagacu

................gauuguugcucagaaagcc..................................................   1   0   seq
................gauuguugcucagaaagcca.................................................   1   0   seq
................gauuguugcucagaaagccauc...............................................   1   0   seq
.................auuguugcucagaaagcc..................................................   1   0   seq
......................................ucuaaaaagcguggcuaucga..........................   1   0   seq
......................................ucuaaaaagcguggcuaucgagcaa......................   5   0   seq
......................................ucuaaaaagcguggcuaucgagcaac.....................   1   0   seq
.................................................uggcuaucgagcaacaaucaa...............   2   0   seq
.................................................uggcuaucgagcaacaaucaaa..............   2   0   seq
...................................................gcuaucgagcaacaaucaaag.............   1   0   seq
...................................................gcuaucgagcaacaaucaaaga............   22  0   seq
...................................................gcuaucgagcaacaaucaaagau...........   1   0   seq
....................................................cuaucgagcaacaaucaaa..............   1   0   seq
....................................................cuaucgagcaacaaucaaag.............   3   0   seq
....................................................cuaucgagcaacaaucaaaga............   5   0   seq
....................................................cuaucgagcaacaaucaaagau...........   17  0   seq
.....................................................uaucgagcaacaaucaaag.............   2   0   seq
.....................................................uaucgagcaacaaucaaaga............   8   0   seq
.....................................................uaucgagcaacaaucaaagau...........   6   0   seq
.....................................................uaucgagcaacaaucaaagauu..........   3   0   seq
......................................................aucgagcaacaaucaaaga............   2   0   seq
......................................................aucgagcaacaaucaaagau...........   1   0   seq
```
