# Supplementary material for: Identification of Conserved and Novel MicroRNAs in the Pacific Oyster Crassostrea gigas by Deep Sequencing
Source: PLoS One. 2014 Aug 19;9(8):e104371. doi: 10.1371/journal.pone.0104371 (PMC4138081; doi:10.1371/journal.pone.0104371)
Supplement: File S2 — The compressed/ZIP file archive for the predicted precursors' secondary structures and reads alignment. (ZIP) [file pone.0104371.s010.zip › second structure and reads alignment for oyster miRNAs/conserved in table S4/cgi-miR-317a.pdf]

miRBase precursor : cgi-miR-317a
Total read count : 146971
cgi-miR-317a-5p read count: 377
cgi-miR-317a-3p read count: 146588
remaining reads : 6

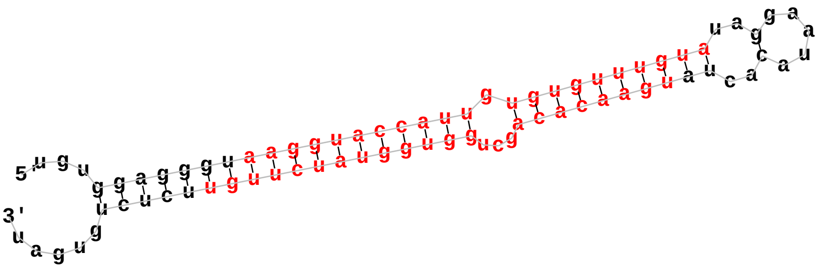

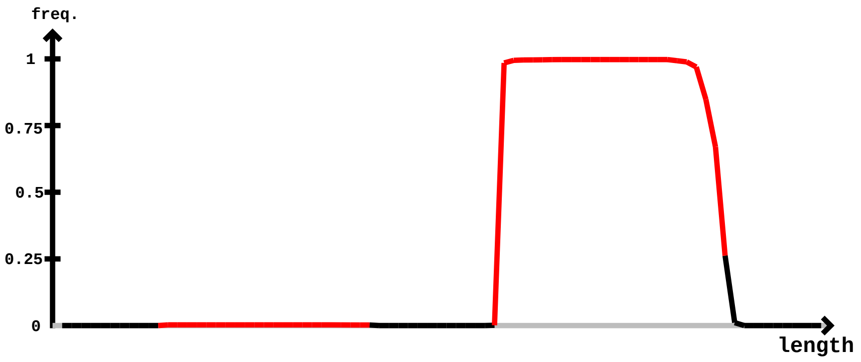


```
                                                                  cgi-miR-317a-3p
           cgi-miR-317a-5p
5'- uguggagguaagguaccauuguguguuuguauaggaauacacuaugaacacagcuggugguaucuuguucucugugau   -3'    exp
    ...((((((((((((((((((.((((((((((..(.....)..))))))))))...))))))))))))))))))).....    reads      mm         sample
    ..........aagguaccauuguguguu...................................................    28         0          seq
    ..........aagguaccauuguguguuu..................................................    23         0          seq
    ..........aagguaccauuguguguuug.................................................    12         0          seq
    ..........aagguaccauuguguguuugu................................................    1          0          seq
    ..........aagguaccauuguguguuugua...............................................    310        0          seq
    ..........aagguaccauuguguguuuguaua.............................................    1          0          seq
    ..........aagguaccauuguguguuuguauag............................................    1          0          seq
    ..........aagguaccauuguguguuuguauaggaa.........................................    1          0          seq
    ...........agguaccauuguguguuugua...............................................    1          0          seq
    ................................uaggaauacacuaugaacaca..........................    1          0          seq
    ................................uaggaauacacuaugaacacagcu.......................    1          0          seq
    ................................uaggaauacacuaugaacacagcug......................    1          0          seq
    ................................uaggaauacacuaugaacacagcuggugg..................    1          0          seq
    .......................................acacuaugaacacagcuggugguaucuug...........    1          0          seq
    ...........................................uaugaacacagcuggugguaucuugu..........    1          0          seq
    ............................................augaacacagcugguggu.................    1          0          seq
    ............................................augaacacagcuggugguа................    2          0          seq
    ............................................augaacacagcuggugguau...............    4          0          seq
    ............................................augaacacagcuggugguauc..............    3          0          seq
    ............................................augaacacagcuggugguaucu.............    16         0          seq
    ............................................augaacacagcuggugguaucuu............    16         0          seq
    ............................................augaacacagcuggugguaucuug...........    32         0          seq
    ............................................augaacacagcuggugguaucuugu..........    81         0          seq
    .............................................ugaacacagcugguggua................    625        0          seq
    .............................................ugaacacagcuggugguau...............    634        0          seq
    .............................................ugaacacagcuggugguauc..............    2768       0          seq
    .............................................ugaacacagcuggugguaucu.............    17692      0          seq
    .............................................ugaacacagcuggugguaucuu............    25826      0          seq
    .............................................ugaacacagcuggugguaucuug...........    59288      0          seq
    .............................................ugaacacagcuggugguaucuugu..........    36211      0          seq
    .............................................ugaacacagcuggugguaucuuguu.........    1524       0          seq
    .............................................ugaacacagcuggugguaucuuguuc........    3          0          seq
    .............................................ugaacacagcuggugguaucuuguucu.......    2          0          seq
    ..............................................gaacacagcuggugguau...............    6          0          seq
```

```
                                             cgi-miR-317a-3p
          cgi-miR-317a-5p
uguggaggguaagguaccauuguguguuuguauaggaauacacuaugaacacagcuggugguaucuuguucucugugau

..............................................gaacacagcuggugguauc..............    35    0    seq
..............................................gaacacagcuggugguaucu.............    131   0    seq
..............................................gaacacagcuggugguaucuu............    284   0    seq
..............................................gaacacagcuggugguaucuug...........    522   0    seq
..............................................gaacacagcuggugguaucuugu..........    424   0    seq
..............................................gaacacagcuggugguaucuuguu.........    21    0    seq
...............................................aacacagcuggugguauc..............    3     0    seq
...............................................aacacagcuggugguaucu.............    28    0    seq
...............................................aacacagcuggugguaucuu............    47    0    seq
...............................................aacacagcuggugguaucuug...........    63    0    seq
...............................................aacacagcuggugguaucuugu..........    65    0    seq
...............................................aacacagcuggugguaucuuguu.........    1     0    seq
................................................acacagcuggugguaucu.............    15    0    seq
................................................acacagcuggugguaucuu............    5     0    seq
................................................acacagcuggugguaucuug...........    12    0    seq
................................................acacagcuggugguaucuugu..........    8     0    seq
................................................acacagcuggugguaucuuguu.........    1     0    seq
.................................................cacagcuggugguaucuu............    9     0    seq
.................................................cacagcuggugguaucuug...........    14    0    seq
.................................................cacagcuggugguaucuugu..........    19    0    seq
.................................................cacagcuggugguaucuuguu.........    7     0    seq
..................................................acagcuggugguaucuug...........    32    0    seq
..................................................acagcuggugguaucuugu..........    49    0    seq
..................................................acagcuggugguaucuuguu.........    29    0    seq
...................................................cagcuggugguaucuugu..........    19    0    seq
...................................................cagcuggugguaucuuguu.........    9     0    seq
....................................................agcuggugguaucuuguu.........    1     0    seq
```
