# Supplementary material for: Identification of Conserved and Novel MicroRNAs in the Pacific Oyster Crassostrea gigas by Deep Sequencing
Source: PLoS One. 2014 Aug 19;9(8):e104371. doi: 10.1371/journal.pone.0104371 (PMC4138081; doi:10.1371/journal.pone.0104371)
Supplement: File S2 — The compressed/ZIP file archive for the predicted precursors' secondary structures and reads alignment. (ZIP) [file pone.0104371.s010.zip › second structure and reads alignment for oyster miRNAs/conserved in table S4/cgi-miR-317b.pdf]

miRBase precursor : cgi-miR-317b
Total read count : 159989
cgi-miR-317b-5p read count : 508
cgi-miR-317b-3p read count : 159478
remaining reads : 3

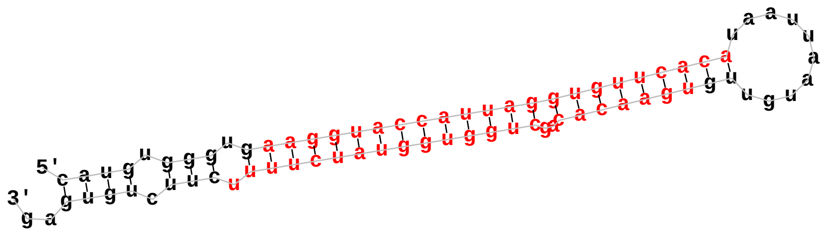

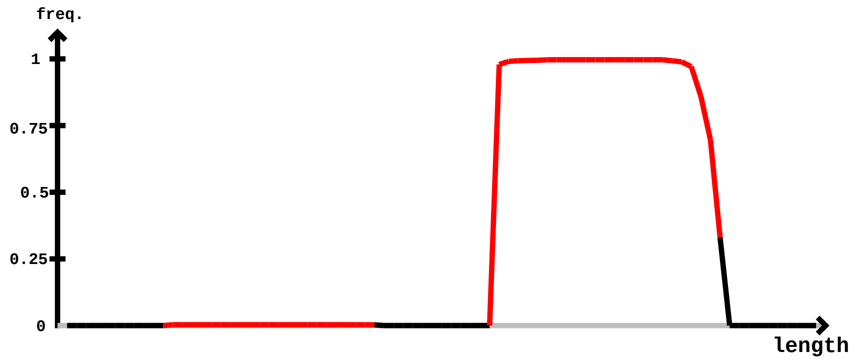

cgi-miR-317b-3p

cgi-miR-317b-5p

5'- caugugggugaagguaccauuagguguucacauaauuaauguugugaacacagcuggugguaucuuuucuucugugag -3'	exp		
((((.(((.(((((((((((((((((((((((..........)))))))))..)))))))))))))).))).))))..	reads	mm	sample
..........aagguaccauuagguguu..	3	0	seq
..........aagguaccauuagguguuc...	4	0	seq
..........aagguaccauuagguguuca..	27	0	seq
..........aagguaccauuagguguucac...	25	0	seq
..........aagguaccauuagguguucaca..	444	0	seq
...........agguaccauuagguguucac...	1	0	seq
...........agguaccauuagguguucaca..	3	0	seq
..............uaccauuagguguucaca..	1	0	seq
................................uaauuaauguugugaacacagcuggugg..................	1	0	seq
....uugugaacacagcuggugguauc..............	1	0	seq
....uugugaacacagcuggugguaucu.............	1	0	seq
....ugugaacacagcugguggua................	1	0	seq
....ugugaacacagcuggugguau...............	1	0	seq
....ugugaacacagcuggugguauc..............	1	0	seq
....gugaacacagcuggugguaucuu............	1	0	seq
....gugaacacagcuggugguaucuuu...........	1	0	seq
....ugaacacagcugguggua................	625	0	seq
....ugaacacagcuggugguau...............	634	0	seq
....ugaacacagcuggugguauc..............	2768	0	seq
....ugaacacagcuggugguaucu.............	17692	0	seq
....ugaacacagcuggugguaucuu............	25826	0	seq
....ugaacacagcuggugguaucuuu...........	57241	0	seq
....ugaacacagcuggugguaucuuuu..........	51861	0	seq
....ugaacacagcuggugguaucuuuuc.........	9	0	seq
....gaacacagcuggugguau...............	6	0	seq
....gaacacagcuggugguauc..............	35	0	seq
....gaacacagcuggugguaucu.............	131	0	seq
....gaacacagcuggugguaucuu............	284	0	seq
....gaacacagcuggugguaucuuu...........	710	0	seq
....gaacacagcuggugguaucuuuu..........	674	0	seq
....gaacacagcuggugguaucuuuuc.........	1	0	seq
....aacacagcuggugguauc..............	3	0	seq
....aacacagcuggugguaucu.............	28	0	seq
....aacacagcuggugguaucuu............	47	0	seq

```
                                            cgi-miR-317b-3p
          cgi-miR-317b-5p
cauguggugaagguaccauuagguguucacauaauuaauguugugaacacagcuggugguaucuuuucuucugugag

..............................................aacacagcuggugguaucuuu...........    150    0    seq
..............................................aacacagcuggugguaucuuuu..........    150    0    seq
..............................................aacacagcuggugguaucuuuucu........    1      0    seq
...............................................acacagcuggugguaucu.............    15     0    seq
...............................................acacagcuggugguaucuu............    5      0    seq
...............................................acacagcuggugguaucuuu...........    91     0    seq
...............................................acacagcuggugguaucuuuu..........    29     0    seq
................................................cacagcuggugguaucuu............    9      0    seq
................................................cacagcuggugguaucuuu...........    56     0    seq
................................................cacagcuggugguaucuuuu..........    46     0    seq
.................................................acagcuggugguaucuuu...........    177    0    seq
.................................................acagcuggugguaucuuuu..........    112    0    seq
..................................................cagcuggugguaucuuuu..........    57     0    seq
```
