# Supplementary material for: Identification of Conserved and Novel MicroRNAs in the Pacific Oyster Crassostrea gigas by Deep Sequencing
Source: PLoS One. 2014 Aug 19;9(8):e104371. doi: 10.1371/journal.pone.0104371 (PMC4138081; doi:10.1371/journal.pone.0104371)
Supplement: File S2 — The compressed/ZIP file archive for the predicted precursors' secondary structures and reads alignment. (ZIP) [file pone.0104371.s010.zip › second structure and reads alignment for oyster miRNAs/conserved in table S4/cgi-miR-33.pdf]

miRBase precursor : cgi-miR-33
Total read count : 68514
cgi-miR-33-5p read count : 68375
cgi-miR-33-3p read count : 138
remaining reads : 1

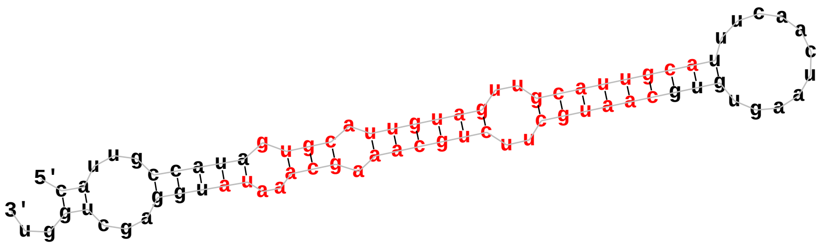

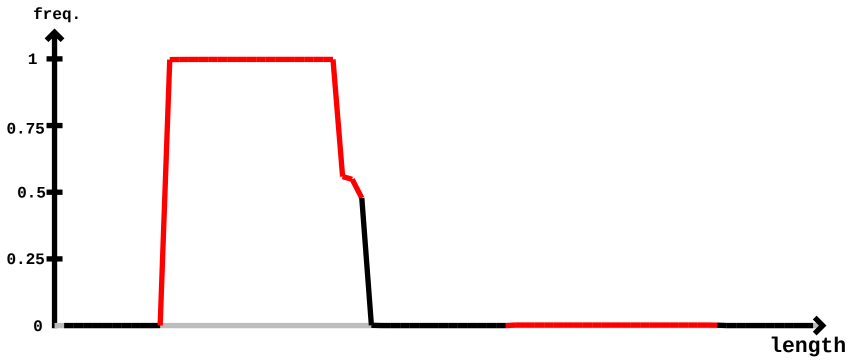


```
                                                         cgi-miR-33-3p
          cgi-miR-33-5p
5'- cauugccauagugcauuguaguugcauugcauuucaacuaagugugcaaugcuucugcaaagcaaauauggagcuggu  -3'   exp
    ((...(((((.(((.((((((..(((((((((...........)))))))))..)))))).)))..)))))...))..   reads      mm         sample
    ........uagugcauuguaguugcau...................................................   2          0          seq
    ........uagugcauuguaguugcauu..................................................   1          0          seq
    .........agugcauuguaguugcauugca...............................................   10         0          seq
    ..........gugcauuguaguugcauu..................................................   30102      0          seq
    ..........gugcauuguaguugcauug.................................................   688        0          seq
    ..........gugcauuguaguugcauugc................................................   4802       0          seq
    ..........gugcauuguaguugcauugca...............................................   32652      0          seq
    ..........gugcauuguaguugcauugcau..............................................   68         0          seq
    ..........gugcauuguaguugcauugcauu.............................................   6          0          seq
    ...........ugcauuguaguugcauug.................................................   6          0          seq
    ...........ugcauuguaguugcauugc................................................   6          0          seq
    ...........ugcauuguaguugcauugca...............................................   20         0          seq
    ............gcauuguaguugcauugc................................................   1          0          seq
    ............gcauuguaguugcauugca...............................................   6          0          seq
    .............cauuguaguugcauugca...............................................   5          0          seq
    ...............................uuucaacuaagugugcaaugcuuc.......................   1          0          seq
    ..............................................caaugcuucugcaaagcaaa............   10         0          seq
    ..............................................caaugcuucugcaaagcaaau...........   24         0          seq
    ..............................................caaugcuucugcaaagcaaaua..........   102        0          seq
    ...............................................aaugcuucugcaaagcaaaua..........   2          0          seq
```
