# Supplementary material for: Identification of Conserved and Novel MicroRNAs in the Pacific Oyster Crassostrea gigas by Deep Sequencing
Source: PLoS One. 2014 Aug 19;9(8):e104371. doi: 10.1371/journal.pone.0104371 (PMC4138081; doi:10.1371/journal.pone.0104371)
Supplement: File S2 — The compressed/ZIP file archive for the predicted precursors' secondary structures and reads alignment. (ZIP) [file pone.0104371.s010.zip › second structure and reads alignment for oyster miRNAs/conserved in table S4/cgi-miR-34.pdf]

miRBase precursor : cgi-miR-34
Total read count : 201264
cgi-miR-34-5p read count : 200928
cgi-miR-34-3p read count : 230
remaining reads : 106

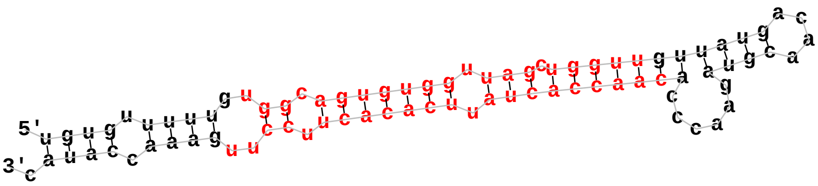

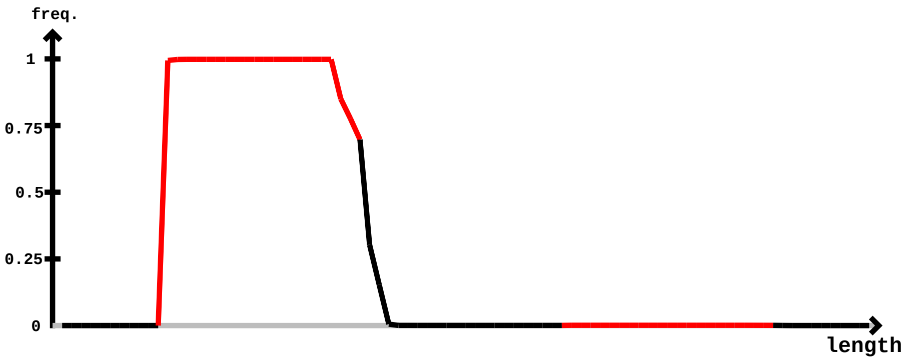


```
                                                                        cgi-miR-34-3p
             cgi-miR-34-5p
5'- uguguuuuuguggcagugugguuagcugguuguuaugacaacguagaacccacaaccacuauucacacuuccuugaaaccauac   -3'    exp
    ((((.((((..((.(((((((.(((.(((((((((((....))))......)))))))))).))))))).))..)))).)))).   reads      mm         sample
    ........uguggcagugugguuagcu.........................................................   1          0          seq
    ........uguggcagugugguuagcugguu.....................................................   1          0          seq
    .........guggcagugugguuagcu.........................................................   2          0          seq
    ..........uggcagugugguuagcug........................................................   29831      0          seq
    ..........uggcagugugguuagcugg.......................................................   14791      0          seq
    ..........uggcagugugguuagcuggu......................................................   15812      0          seq
    ..........uggcagugugguuagcugguu.....................................................   79038      0          seq
    ..........uggcagugugguuagcugguug....................................................   29995      0          seq
    ..........uggcagugugguuagcugguugu...................................................   29680      0          seq
    ..........uggcagugugguuagcugguuguu..................................................   904        0          seq
    ..........uggcagugugguuagcugguuguua.................................................   14         0          seq
    ..........uggcagugugguuagcugguuguuau................................................   5          0          seq
    ...........ggcagugugguuagcugg.......................................................   51         0          seq
    ...........ggcagugugguuagcuggu......................................................   68         0          seq
    ...........ggcagugugguuagcugguu.....................................................   313        0          seq
    ...........ggcagugugguuagcugguug....................................................   100        0          seq
    ...........ggcagugugguuagcugguugu...................................................   163        0          seq
    ...........ggcagugugguuagcugguuguu..................................................   45         0          seq
    ...........ggcagugugguuagcugguuguua.................................................   1          0          seq
    ...........ggcagugugguuagcugguuguuau................................................   1          0          seq
    ...........ggcagugugguuagcugguuguuaug...............................................   1          0          seq
    ............gcagugugguuagcuggu......................................................   11         0          seq
    ............gcagugugguuagcugguu.....................................................   48         0          seq
    ............gcagugugguuagcugguug....................................................   15         0          seq
    ............gcagugugguuagcugguugu...................................................   17         0          seq
    ............gcagugugguuagcugguuguu..................................................   8          0          seq
    ............gcagugugguuagcugguuguuau................................................   1          0          seq
    .............cagugugguuagcugguu.....................................................   1          0          seq
    .............cagugugguuagcugguug....................................................   1          0          seq
    ..............agugugguuagcugguug....................................................   1          0          seq
    ..............agugugguuagcugguugu...................................................   5          0          seq
    ..............agugugguuagcugguuguu..................................................   1          0          seq
    ...............gugugguuagcugguugu...................................................   3          0          seq
    .................................uaugacaacguagaaccca................................   17         0          seq
```

```
                                                            cgi-miR-34-3p
          cgi-miR-34-5p
uguguuuuuguggcagugugguuagcugguuguuaugacaacguagaacccacaaccacuauucacacuuccuugaaaccauac

.................................uaugacaacguagaacccac...............................  86  0  seq
.................................uaugacaacguagaacccaca..............................  1   0  seq
..................................augacaacguagaacccacaac............................  1   0  seq
....................................................caaccacuauucacacuu..............  4   0  seq
....................................................caaccacuauucacacuuc.............  11  0  seq
....................................................caaccacuauucacacuucc............  20  0  seq
....................................................caaccacuauucacacuuccu...........  26  0  seq
....................................................caaccacuauucacacuuccuu..........  74  0  seq
....................................................caaccacuauucacacuuccuug.........  2   0  seq
.....................................................aaccacuauucacacuuc.............  4   0  seq
.....................................................aaccacuauucacacuucc............  1   0  seq
.....................................................aaccacuauucacacuuccu...........  3   0  seq
.....................................................aaccacuauucacacuuccuu..........  17  0  seq
.....................................................aaccacuauucacacuuccuug.........  65  0  seq
.....................................................aaccacuauucacacuuccuuga........  1   0  seq
......................................................accacuauucacacuuccuu..........  2   0  seq
```
