# Supplementary material for: Identification of Conserved and Novel MicroRNAs in the Pacific Oyster Crassostrea gigas by Deep Sequencing
Source: PLoS One. 2014 Aug 19;9(8):e104371. doi: 10.1371/journal.pone.0104371 (PMC4138081; doi:10.1371/journal.pone.0104371)
Supplement: File S2 — The compressed/ZIP file archive for the predicted precursors' secondary structures and reads alignment. (ZIP) [file pone.0104371.s010.zip › second structure and reads alignment for oyster miRNAs/conserved in table S4/cgi-miR-36.pdf]

miRBase precursor : cgi-miR-36
Total read count : 20456
cgi-miR-36-5p read count : 1959
cgi-miR-36-3p read count : 18496
remaining reads : 1

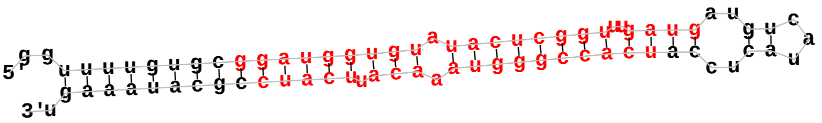

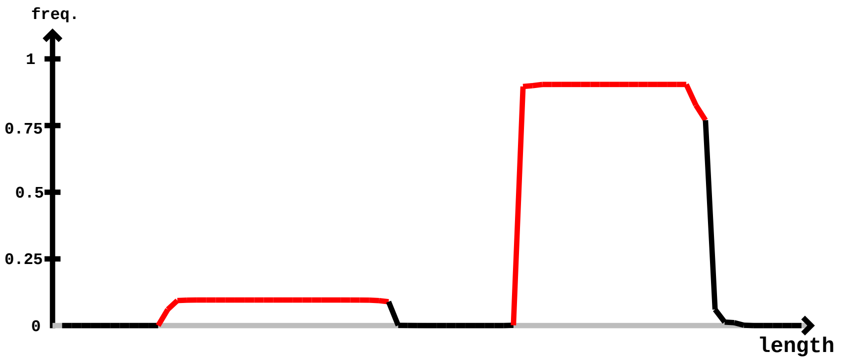


```
             cgi-miR-36-5p
                                          cgi-miR-36-3p
5'- gguuuugugcggaugguguauacucgguuugaugaugucauacuccaucaccggguaaacauucauccgcauaaagu   -3'    exp
    ..(((((((((((((((((.((((((((..((((..((...))..)))))))))))).))).)))))))))))))).    reads     mm       sample
    .....ugugcggaugguguauacu.....................................................    1         0        seq
    ..........ggaugguguauacucgguuu...............................................    1         0        seq
    ..........ggaugguguauacucgguuug..............................................    5         0        seq
    ..........ggaugguguauacucgguuuga.............................................    14        0        seq
    ..........ggaugguguauacucgguuugau............................................    30        0        seq
    ..........ggaugguguauacucgguuugaug...........................................    1180      0        seq
    ..........ggaugguguauacucgguuugauga..........................................    2         0        seq
    ..........ggaugguguauacucgguuugaugau.........................................    1         0        seq
    ..........ggaugguguauacucgguuugaugaug........................................    1         0        seq
    ...........gaugguguauacucgguu................................................    1         0        seq
    ...........gaugguguauacucgguuu...............................................    2         0        seq
    ...........gaugguguauacucgguuug..............................................    1         0        seq
    ...........gaugguguauacucgguuuga.............................................    21        0        seq
    ...........gaugguguauacucgguuugau............................................    49        0        seq
    ...........gaugguguauacucgguuugaug...........................................    609       0        seq
    ............augguguauacucgguuuga.............................................    1         0        seq
    ............augguguauacucgguuugau............................................    4         0        seq
    ............augguguauacucgguuugaug...........................................    21        0        seq
    ............augguguauacucgguuugauga..........................................    5         0        seq
    ............augguguauacucgguuugaugau.........................................    1         0        seq
    .............ugguguauacucgguuugau............................................    1         0        seq
    .............ugguguauacucgguuugaug...........................................    4         0        seq
    .............ugguguauacucgguuugaugau.........................................    3         0        seq
    .............ugguguauacucgguuugaugaug........................................    2         0        seq
    .............................................caucaccggguaaacauucauccgc.......    1         0        seq
    ..............................................aucaccggguaaacauucau...........    2         0        seq
    ..............................................aucaccggguaaacauucaucc.........    2         0        seq
    ..............................................aucaccggguaaacauucauccg........    1         0        seq
    ..............................................aucaccggguaaacauucauccgc.......    11        0        seq
    ..............................................aucaccggguaaacauucauccgca......    5         0        seq
    ...............................................ucaccggguaaacauuca............    1589      0        seq
    ...............................................ucaccggguaaacauucau...........    1148      0        seq
    ...............................................ucaccggguaaacauucauc..........    14421     0        seq
    ...............................................ucaccggguaaacauucaucc.........    941       0        seq
```

```
          cgi-miR-36-5p
                                               cgi-miR-36-3p
gguuuugugcggaugguguauacucgguuugaugaugucauacuccaucaccggguaaacauucauccgcauaaagu

...............................................ucaccggguaaacauucauccg........    38    0    seq
...............................................ucaccggguaaacauucauccgc.......    159   0    seq
...............................................ucaccggguaaacauucauccgca......    18    0    seq
................................................caccggguaaacauucau...........    6     0    seq
................................................caccggguaaacauucauc..........    35    0    seq
................................................caccggguaaacauucaucc.........    9     0    seq
................................................caccggguaaacauucauccg........    5     0    seq
................................................caccggguaaacauucauccgc.......    11    0    seq
.................................................accggguaaacauucauc..........    63    0    seq
.................................................accggguaaacauucaucc.........    12    0    seq
.................................................accggguaaacauucauccg........    4     0    seq
.................................................accggguaaacauucauccgc.......    10    0    seq
.................................................accggguaaacauucauccgca......    3     0    seq
..................................................ccggguaaacauucauccgc.......    1     0    seq
...................................................cggguaaacauucauccgc.......    1     0    seq
```
