# Supplementary material for: Identification of Conserved and Novel MicroRNAs in the Pacific Oyster Crassostrea gigas by Deep Sequencing
Source: PLoS One. 2014 Aug 19;9(8):e104371. doi: 10.1371/journal.pone.0104371 (PMC4138081; doi:10.1371/journal.pone.0104371)
Supplement: File S2 — The compressed/ZIP file archive for the predicted precursors' secondary structures and reads alignment. (ZIP) [file pone.0104371.s010.zip › second structure and reads alignment for oyster miRNAs/conserved in table S4/cgi-miR-375.pdf]

miRBase precursor : cgi-miR-375
Total read count : 506528
cgi-miR-375-5p read count : 1974
cgi-miR-375-3p read count : 504554
remaining reads : 0

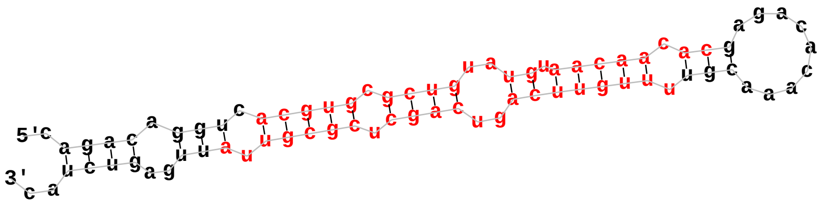

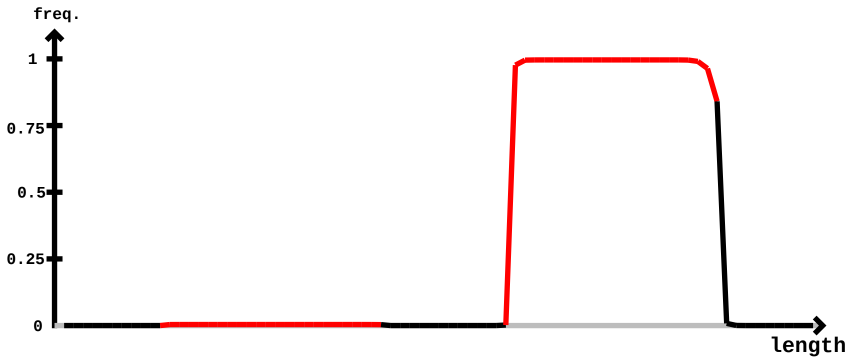


```
                                              cgi-miR-375-3p
            cgi-miR-375-5p
5'- cagacaggucacgugcgcuguauguaacaacacgagacacaaacguuuuguucagucagcucgcguuauugagucuac   -3'    exp
    .((((.(((.(((((.((((..((.(((((.(((.........))).)))))))..)))).))))).))).))))..    reads      mm         sample
    .........cacgugcgcuguauguaacaacac.............................................    1          0          seq
    ..........acgugcgcuguauguaacaa................................................    6          0          seq
    ..........acgugcgcuguauguaacaac...............................................    27         0          seq
    ..........acgugcgcuguauguaacaaca..............................................    175        0          seq
    ..........acgugcgcuguauguaacaacac.............................................    1725       0          seq
    ..........acgugcgcuguauguaacaacacg............................................    2          0          seq
    ..........acgugcgcuguauguaacaacacga...........................................    1          0          seq
    ..........acgugcgcuguauguaacaacacgag..........................................    4          0          seq
    ..........acgugcgcuguauguaacaacacgaga.........................................    2          0          seq
    ...........cgugcgcuguauguaacaaca..............................................    2          0          seq
    ...........cgugcgcuguauguaacaacac.............................................    25         0          seq
    ...........cgugcgcuguauguaacaacacg............................................    3          0          seq
    .............ugcgcuguauguaacaaca..............................................    1          0          seq
    .............................................uuuuguucagucagcucg...............    1          0          seq
    .............................................uuuuguucagucagcucgc..............    1          0          seq
    .............................................uuuuguucagucagcucgcg.............    14         0          seq
    .............................................uuuuguucagucagcucgcgu............    146        0          seq
    .............................................uuuuguucagucagcucgcguu...........    464        0          seq
    .............................................uuuuguucagucagcucgcguua..........    397        0          seq
    .............................................uuuuguucagucagcucgcguuau.........    14         0          seq
    ..............................................uuuguucagucagcucgc..............    323        0          seq
    ..............................................uuuguucagucagcucgcg.............    2459       0          seq
    ..............................................uuuguucagucagcucgcgu............    13111      0          seq
    ..............................................uuuguucagucagcucgcguu...........    61055      0          seq
    ..............................................uuuguucagucagcucgcguua..........    412700     0          seq
    ..............................................uuuguucagucagcucgcguuau.........    3807       0          seq
    ..............................................uuuguucagucagcucgcguuauu........    194        0          seq
    ...............................................uuguucagucagcucgcg.............    36         0          seq
    ...............................................uuguucagucagcucgcgu............    189        0          seq
    ...............................................uuguucagucagcucgcguu...........    869        0          seq
    ...............................................uuguucagucagcucgcguua..........    8213       0          seq
    ...............................................uuguucagucagcucgcguuau.........    128        0          seq
    ...............................................uuguucagucagcucgcguuauu........    6          0          seq
    ................................................uguucagucagcucgcgu............    3          0          seq
```

```
                                              cgi-miR-375-3p
          cgi-miR-375-5p
cagacaggucacgugcgcuguauguaacaacacgagacacaaacguuuuguucagucagcucgcguuauugagucuac

................................................uguucagucagcucgcguu...........    54      0       seq
................................................uguucagucagcucgcguua..........    331     0       seq
................................................uguucagucagcucgcguuau.........    1       0       seq
................................................uguucagucagcucgcguuauu........    9       0       seq
.................................................guucagucagcucgcguu...........    3       0       seq
.................................................guucagucagcucgcguua..........    6       0       seq
..................................................uucagucagcucgcguua..........    20      0       seq
```
