# Supplementary material for: Identification of Conserved and Novel MicroRNAs in the Pacific Oyster Crassostrea gigas by Deep Sequencing
Source: PLoS One. 2014 Aug 19;9(8):e104371. doi: 10.1371/journal.pone.0104371 (PMC4138081; doi:10.1371/journal.pone.0104371)
Supplement: File S2 — The compressed/ZIP file archive for the predicted precursors' secondary structures and reads alignment. (ZIP) [file pone.0104371.s010.zip › second structure and reads alignment for oyster miRNAs/conserved in table S4/cgi-miR-67a.pdf]

miRBase precursor : cgi-miR-67a
Total read count : 292414
cgi-miR-67a-5p read count : 3871
cgi-miR-67a-3p read count : 288540
remaining reads : 3

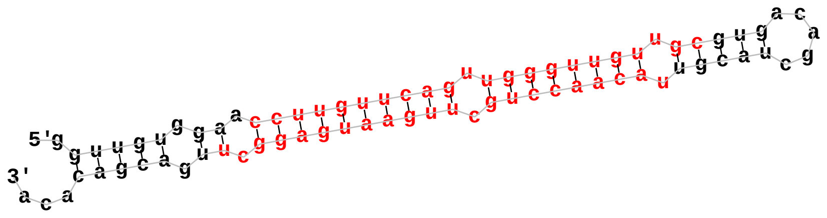

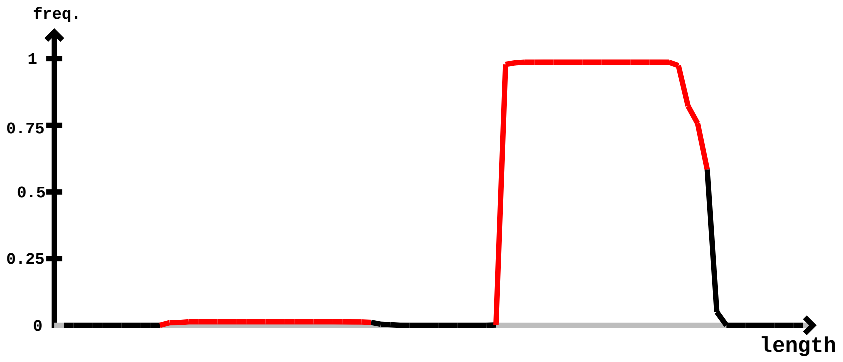


```
                                                   cgi-miR-67a-3p
             cgi-miR-67a-5p
5'- gguuguggaaccuuguucaguuggguuguugcgugacagcuacguuacaaccugcuugaaugaggcuugacgacaca -3'   exp
    .(((((.((.((((((((((.((((((((.(((((.....))))).)))))))).)))))))))).)).)))))...   reads   mm   sample
    ........aaccuuguucaguuggguu..................................................   2       0    seq
    .........ccuuguucaguuggguug..................................................   65      0    seq
    .........ccuuguucaguuggguugu.................................................   88      0    seq
    .........ccuuguucaguuggguuguu................................................   33      0    seq
    .........ccuuguucaguuggguuguug...............................................   435     0    seq
    .........ccuuguucaguuggguuguugc..............................................   1973    0    seq
    .........ccuuguucaguuggguuguugcg.............................................   272     0    seq
    .........ccuuguucaguuggguuguugcgu............................................   17      0    seq
    .........ccuuguucaguuggguuguugcgug...........................................   12      0    seq
    .........ccuuguucaguuggguuguugcguga..........................................   1       0    seq
    .........ccuuguucaguuggguuguugcgugac.........................................   3       0    seq
    .........ccuuguucaguuggguuguugcgugacagcuac...................................   1       0    seq
    ..........cuuguucaguuggguugu.................................................   1       0    seq
    ..........cuuguucaguuggguuguu................................................   1       0    seq
    ..........cuuguucaguuggguuguug...............................................   3       0    seq
    ..........cuuguucaguuggguuguugc..............................................   12      0    seq
    ..........cuuguucaguuggguuguugcg.............................................   96      0    seq
    ..........cuuguucaguuggguuguugcgug...........................................   1       0    seq
    ...........uuguucaguuggguuguu................................................   5       0    seq
    ...........uuguucaguuggguuguug...............................................   27      0    seq
    ...........uuguucaguuggguuguugc..............................................   39      0    seq
    ...........uuguucaguuggguuguugcg.............................................   136     0    seq
    ...........uuguucaguuggguuguugcgu............................................   632     0    seq
    ...........uuguucaguuggguuguugcgugacagc......................................   1       0    seq
    ............uguucaguuggguuguug...............................................   1       0    seq
    ............uguucaguuggguuguugc..............................................   3       0    seq
    ............uguucaguuggguuguugcgu............................................   3       0    seq
    ............uguucaguuggguuguugcgug...........................................   6       0    seq
    ............uguucaguuggguuguugcgugacagcua....................................   1       0    seq
    .............gucaguuggguuguugcgu.............................................   3       0    seq
    ................caguuggguuguugcgugac.........................................   1       0    seq
    ...........................................guuacaaccugcuugaauga..............   1       0    seq
    ...........................................guuacaaccugcuugaaugagg............   2       0    seq
    ............................................uuacaaccugcuugaaug...............   3       0    seq
```

```
                                              cgi-miR-67a-3p
          cgi-miR-67a-5p
gguuguggaaccuuguucaguuggguuguugcgugacagcuacguuacaaccugcuugaaugaggcuugacgacaca

............................................uuacaaccugcuugaauga..............    23      0     seq
............................................uuacaaccugcuugaaugag.............    106     0     seq
............................................uuacaaccugcuugaaugagg............    66      0     seq
............................................uuacaaccugcuugaaugaggc...........    52      0     seq
............................................uuacaaccugcuugaaugaggcu..........    110     0     seq
............................................uuacaaccugcuugaaugaggcuu.........    6       0     seq
.............................................uacaaccugcuugaauga..............    3698    0     seq
.............................................uacaaccugcuugaaugag.............    44165   0     seq
.............................................uacaaccugcuugaaugagg............    18928   0     seq
.............................................uacaaccugcuugaaugaggc...........    50204   0     seq
.............................................uacaaccugcuugaaugaggcu..........    154441  0     seq
.............................................uacaaccugcuugaaugaggcuu.........    14299   0     seq
.............................................uacaaccugcuugaaugaggcuug........    1       0     seq
..............................................acaaccugcuugaaugag.............    81      0     seq
..............................................acaaccugcuugaaugagg............    68      0     seq
..............................................acaaccugcuugaaugaggc...........    224     0     seq
..............................................acaaccugcuugaaugaggcu..........    1148    0     seq
..............................................acaaccugcuugaaugaggcuu.........    212     0     seq
...............................................caaccugcuugaaugagg............    23      0     seq
...............................................caaccugcuugaaugaggc...........    80      0     seq
...............................................caaccugcuugaaugaggcu..........    493     0     seq
...............................................caaccugcuugaaugaggcuu.........    78      0     seq
................................................aaccugcuugaaugaggc...........    1       0     seq
................................................aaccugcuugaaugaggcu..........    22      0     seq
................................................aaccugcuugaaugaggcuu.........    3       0     seq
.................................................accugcuugaaugaggcu..........    2       0     seq
```
