# Supplementary material for: Identification of Conserved and Novel MicroRNAs in the Pacific Oyster Crassostrea gigas by Deep Sequencing
Source: PLoS One. 2014 Aug 19;9(8):e104371. doi: 10.1371/journal.pone.0104371 (PMC4138081; doi:10.1371/journal.pone.0104371)
Supplement: File S2 — The compressed/ZIP file archive for the predicted precursors' secondary structures and reads alignment. (ZIP) [file pone.0104371.s010.zip › second structure and reads alignment for oyster miRNAs/conserved in table S4/cgi-miR-67b.pdf]

miRBase precursor : cgi-miR-67b
Total read count : 170176
cgi-miR-67b-5p read count : 16236
cgi-miR-67b-3p read count : 153938
remaining reads : 2

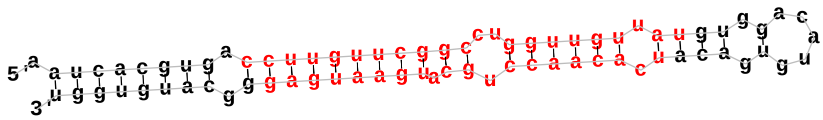

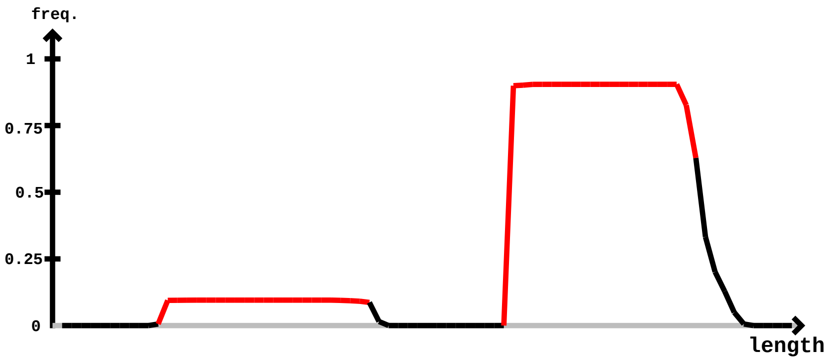


```
                    cgi-miR-67b-5p
                                                cgi-miR-67b-3p
5'- aaucacgugaccuuguucggccugguuguuauguggacaugugacaucacaaccugcaugaaugagggcauguggu   -3'   exp
    .((((((((.(((((((((((..((((((.((((.(.....).)))).)))))).)).))))))))).))))))))   reads     mm       sample
    .........accuuguucggccugguu.................................................   9         0        seq
    .........accuuguucggccugguug................................................   81        0        seq
    .........accuuguucggccugguugu...............................................   164       0        seq
    .........accuuguucggccugguuguu..............................................   291       0        seq
    .........accuuguucggccugguuguua.............................................   126       0        seq
    .........accuuguucggccugguuguuau............................................   286       0        seq
    .........accuuguucggccugguuguuaug...........................................   40        0        seq
    .........accuuguucggccugguuguuaugu..........................................   1         0        seq
    ..........ccuuguucggccugguug................................................   61        0        seq
    ..........ccuuguucggccugguugu...............................................   54        0        seq
    ..........ccuuguucggccugguuguu..............................................   48        0        seq
    ..........ccuuguucggccugguuguua.............................................   633       0        seq
    ..........ccuuguucggccugguuguuau............................................   11787     0        seq
    ..........ccuuguucggccugguuguuaug...........................................   2499      0        seq
    ..........ccuuguucggccugguuguuaugu..........................................   9         0        seq
    ..........ccuuguucggccugguuguuaugugga.......................................   1         0        seq
    ...........cuuguucggccugguuguua.............................................   2         0        seq
    ...........cuuguucggccugguuguuau............................................   38        0        seq
    ...........cuuguucggccugguuguuaug...........................................   8         0        seq
    ............uuguucggccugguuguua.............................................   1         0        seq
    ............uuguucggccugguuguuau............................................   43        0        seq
    ............uuguucggccugguuguuaug...........................................   17        0        seq
    .............uguucggccugguuguua.............................................   3         0        seq
    .............uguucggccugguuguuau............................................   17        0        seq
    .............uguucggccugguuguuaug...........................................   9         0        seq
    ..............guucggccugguuguuau............................................   6         0        seq
    ...............uucggccugguuguuaug...........................................   2         0        seq
    .................................uggacaugugacaucacaaccugcaugaau.............   1         0        seq
    .......................................ugugacaucacaaccugcaugaau.............   1         0        seq
    .............................................aucacaaccugcaugaaug............   1         0        seq
    .............................................aucacaaccugcaugaaugag..........   4         0        seq
    .............................................aucacaaccugcaugaaugagg.........   7         0        seq
    .............................................aucacaaccugcaugaaugaggg........   1         0        seq
    .............................................aucacaaccugcaugaaugagggc.......   5         0        seq
```

```
          cgi-miR-67b-5p
                                                cgi-miR-67b-3p
aaucacgugaccuuguucggccugguuguuauguggacaugugacaucacaaccugcaugaaugagggcauguggu

.............................................aucacaaccugcaugaaugagggca......    3       0     seq
..............................................ucacaaccugcaugaaug............    13286   0     seq
..............................................ucacaaccugcaugaauga...........    33690   0     seq
..............................................ucacaaccugcaugaaugag..........    50125   0     seq
..............................................ucacaaccugcaugaaugagg.........    22122   0     seq
..............................................ucacaaccugcaugaaugaggg........    12258   0     seq
..............................................ucacaaccugcaugaaugagggc.......    13347   0     seq
..............................................ucacaaccugcaugaaugagggca......    7221    0     seq
..............................................ucacaaccugcaugaaugagggcau.....    992     0     seq
...............................................cacaaccugcaugaauga...........    30      0     seq
...............................................cacaaccugcaugaaugag..........    130     0     seq
...............................................cacaaccugcaugaaugagg.........    66      0     seq
...............................................cacaaccugcaugaaugaggg........    44      0     seq
...............................................cacaaccugcaugaaugagggc.......    44      0     seq
...............................................cacaaccugcaugaaugagggca......    18      0     seq
...............................................cacaaccugcaugaaugagggcau.....    3       0     seq
................................................acaaccugcaugaaugag..........    110     0     seq
................................................acaaccugcaugaaugagg.........    110     0     seq
................................................acaaccugcaugaaugaggg........    78      0     seq
................................................acaaccugcaugaaugagggc.......    64      0     seq
................................................acaaccugcaugaaugagggca......    143     0     seq
................................................acaaccugcaugaaugagggcau.....    11      0     seq
.................................................caaccugcaugaaugagg.........    3       0     seq
.................................................caaccugcaugaaugaggg........    2       0     seq
.................................................caaccugcaugaaugagggc.......    5       0     seq
.................................................caaccugcaugaaugagggca......    3       0     seq
..................................................aaccugcaugaaugaggg........    3       0     seq
..................................................aaccugcaugaaugagggc.......    5       0     seq
..................................................aaccugcaugaaugagggca......    1       0     seq
..................................................aaccugcaugaaugagggcau.....    1       0     seq
...................................................accugcaugaaugagggc.......    1       0     seq
....................................................ccugcaugaaugagggca......    1       0     seq
```
