# Supplementary material for: Identification of Conserved and Novel MicroRNAs in the Pacific Oyster Crassostrea gigas by Deep Sequencing
Source: PLoS One. 2014 Aug 19;9(8):e104371. doi: 10.1371/journal.pone.0104371 (PMC4138081; doi:10.1371/journal.pone.0104371)
Supplement: File S2 — The compressed/ZIP file archive for the predicted precursors' secondary structures and reads alignment. (ZIP) [file pone.0104371.s010.zip › second structure and reads alignment for oyster miRNAs/conserved in table S4/cgi-miR-7.pdf]

miRBase precursor : cgi-miR-7
Total read count : 146143
cgi-miR-7-5p read count : 145956
cgi-miR-7-3p read count : 187
remaining reads : 0

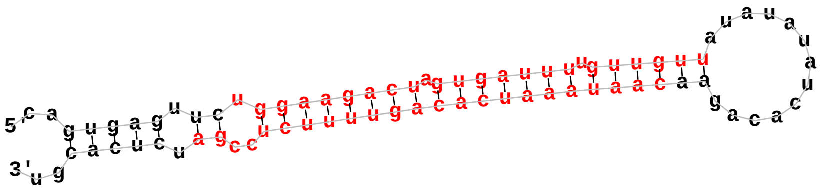

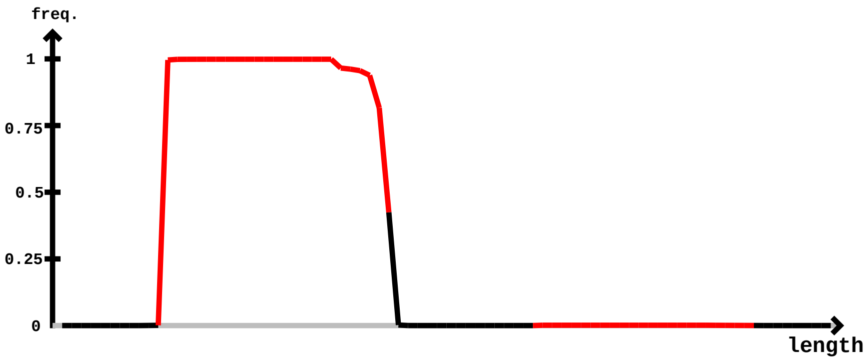


```
                                                 cgi-miR-7-3p
          cgi-miR-7-5p
5'- cagugaguucuggaagacuagugauuuuguuguuauauauaucacagaacaauaaaucacaguuuucuccgaucucacgu  -3'   exp
    ..(((((.((.((((((((.(((((((.((((((............))))))))))))))))))))..)).))))).. reads   mm   sample
    ........ucuggaagacuagugauuu.....................................................  5       0    seq
    ........ucuggaagacuagugauuuu....................................................  26      0    seq
    ........ucuggaagacuagugauuuug...................................................  4       0    seq
    ........ucuggaagacuagugauuuugu..................................................  6       0    seq
    ........ucuggaagacuagugauuuuguug................................................  9       0    seq
    ........ucuggaagacuagugauuuuguugu...............................................  2       0    seq
    ........ucuggaagacuagugauuuuguuguu..............................................  3       0    seq
    .........cuggaagacuagugauuu.....................................................  2       0    seq
    .........cuggaagacuagugauuuu....................................................  14      0    seq
    .........cuggaagacuagugauuuug...................................................  3       0    seq
    .........cuggaagacuagugauuuugu..................................................  1       0    seq
    .........cuggaagacuagugauuuuguu.................................................  3       0    seq
    .........cuggaagacuagugauuuuguug................................................  17      0    seq
    .........cuggaagacuagugauuuuguugu...............................................  25      0    seq
    .........cuggaagacuagugauuuuguuguu..............................................  20      0    seq
    ..........uggaagacuagugauuuu....................................................  4788    0    seq
    ..........uggaagacuagugauuuug...................................................  576     0    seq
    ..........uggaagacuagugauuuugu..................................................  838     0    seq
    ..........uggaagacuagugauuuuguu.................................................  2530    0    seq
    ..........uggaagacuagugauuuuguug................................................  17709   0    seq
    ..........uggaagacuagugauuuuguugu...............................................  57153   0    seq
    ..........uggaagacuagugauuuuguuguu..............................................  61510   0    seq
    ..........uggaagacuagugauuuuguuguua.............................................  299     0    seq
    ..........uggaagacuagugauuuuguuguuau............................................  7       0    seq
    ..........uggaagacuagugauuuuguuguuaua...........................................  1       0    seq
    ...........ggaagacuagugauuuug...................................................  4       0    seq
    ...........ggaagacuagugauuuugu..................................................  3       0    seq
    ...........ggaagacuagugauuuuguu.................................................  10      0    seq
    ...........ggaagacuagugauuuuguug................................................  50      0    seq
    ...........ggaagacuagugauuuuguugu...............................................  127     0    seq
    ...........ggaagacuagugauuuuguuguu..............................................  147     0    seq
    ............gaagacuagugauuuugu..................................................  1       0    seq
    ............gaagacuagugauuuuguu.................................................  1       0    seq
    ............gaagacuagugauuuuguug................................................  5       0    seq
```

```
                                        cgi-miR-7-3p
          cgi-miR-7-5p
cagugaguucuggaagacuagugauuuuguuguuauauauaucacagaacaauaaaucacaguuuucuccgaucucacgu

............gaagacuagugauuuuguugu...............................................    14    0    seq
............gaagacuagugauuuuguuguu..............................................    14    0    seq
.............aagacuagugauuuuguug................................................    1     0    seq
.............aagacuagugauuuuguugu...............................................    3     0    seq
.............aagacuagugauuuuguuguu..............................................    10    0    seq
..............agacuagugauuuuguugu...............................................    2     0    seq
..............agacuagugauuuuguuguu..............................................    8     0    seq
................acuagugauuuuguuguu..............................................    5     0    seq
.................................................caauaaaucacaguuuuc.............    44    0    seq
.................................................caauaaaucacaguuuucu............    33    0    seq
.................................................caauaaaucacaguuuucuc...........    23    0    seq
.................................................caauaaaucacaguuuucucc..........    32    0    seq
.................................................caauaaaucacaguuuucuccg.........    16    0    seq
.................................................caauaaaucacaguuuucuccga........    35    0    seq
..................................................aauaaaucacaguuuucuc...........    1     0    seq
..................................................aauaaaucacaguuuucucc..........    1     0    seq
..................................................aauaaaucacaguuuucuccg.........    1     0    seq
..................................................aauaaaucacaguuuucuccga........    1     0    seq
```
