# Supplementary material for: Identification of Conserved and Novel MicroRNAs in the Pacific Oyster Crassostrea gigas by Deep Sequencing
Source: PLoS One. 2014 Aug 19;9(8):e104371. doi: 10.1371/journal.pone.0104371 (PMC4138081; doi:10.1371/journal.pone.0104371)
Supplement: File S2 — The compressed/ZIP file archive for the predicted precursors' secondary structures and reads alignment. (ZIP) [file pone.0104371.s010.zip › second structure and reads alignment for oyster miRNAs/conserved in table S4/cgi-miR-71.pdf]

miRBase precursor : cgi-miR-71
Total read count : 998152
cgi-miR-71-5p read count : 996421
cgi-miR-71-3p read count : 1729
remaining reads : 2

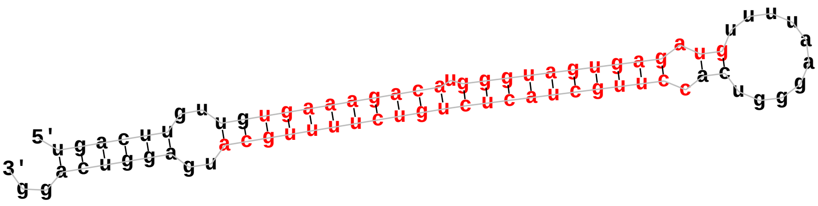

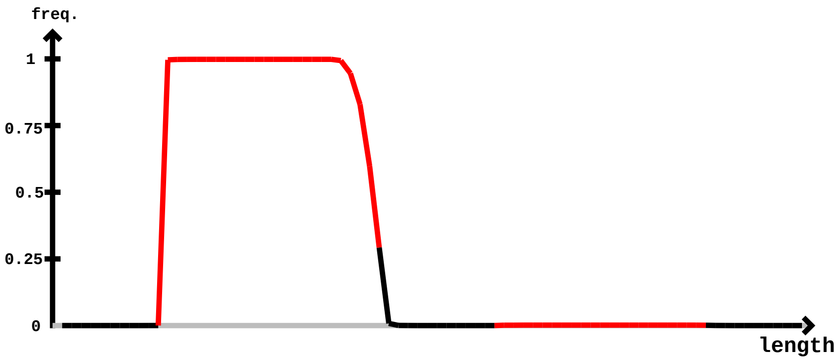


```
        cgi-miR-71-5p
                                          cgi-miR-71-3p
5'- ugacuuguugugaaagacaugguagugagauguuuuaaggguca ccuugcuacucugucuuuugcaugaggucagg   -3'    exp
```

```
5'- ugacuuguugugaaagacaugguagugagauguuuuagggucaccuugcuacucugucuuuugcaugaggucagg   -3'    exp
    ((((((..(((((((((((.((((((((((.((.........)).)))))))))))))))))))))..))))))..   reads    mm    sample
    .......uugugaaagacaugguagugag...............................................   2        0     seq
    ........ugugaaagacaugguag...................................................   3        0     seq
    ........ugugaaagacaugguagu..................................................   5        0     seq
    ........ugugaaagacaugguagug.................................................   2        0     seq
    ........ugugaaagacaugguaguga................................................   16       0     seq
    ........ugugaaagacaugguagugag...............................................   37       0     seq
    ........ugugaaagacaugguagugaga..............................................   50       0     seq
    .........gugaaagacaugguaguga................................................   5        0     seq
    .........gugaaagacaugguagugag...............................................   4        0     seq
    .........gugaaagacaugguagugaga..............................................   4        0     seq
    .........gugaaagacaugguagugagau.............................................   21       0     seq
    .........gugaaagacaugguagugagaug............................................   2        0     seq
    ..........ugaaagacaugguagug.................................................   4623     0     seq
    ..........ugaaagacaugguaguga................................................   47560    0     seq
    ..........ugaaagacaugguagugag...............................................   115852   0     seq
    ..........ugaaagacaugguagugaga..............................................   230896   0     seq
    ..........ugaaagacaugguagugagau.............................................   304302   0     seq
    ..........ugaaagacaugguagugagaug............................................   283115   0     seq
    ..........ugaaagacaugguagugagaugu...........................................   7612     0     seq
    ..........ugaaagacaugguagugagauguu..........................................   241      0     seq
    ..........ugaaagacaugguagugagauguuu.........................................   224      0     seq
    ..........ugaaagacaugguagugagauguuuu........................................   14       0     seq
    ...........gaaagacaugguaguga................................................   47       0     seq
    ...........gaaagacaugguagugag...............................................   125      0     seq
    ...........gaaagacaugguagugaga..............................................   317      0     seq
    ...........gaaagacaugguagugagau.............................................   441      0     seq
    ...........gaaagacaugguagugagaug............................................   496      0     seq
    ...........gaaagacaugguagugagaugu...........................................   17       0     seq
    ...........gaaagacaugguagugagauguu..........................................   1        0     seq
    ............aaagacaugguagugag...............................................   42       0     seq
    ............aaagacaugguagugaga..............................................   70       0     seq
    ............aaagacaugguagugagau.............................................   107      0     seq
    ............aaagacaugguagugagaug............................................   129      0     seq
    ............aaagacaugguagugagaugu...........................................   5        0     seq
```

```
          cgi-miR-71-5p
                                             cgi-miR-71-3p
ugacuuguugugaaagacauggguagugagauguuuuaagggucaccuugcuacucugucuuuugcaugaggucagg

............aaagacauggguagugagauguu..........................................    1     0    seq
............aaagacauggguagugagauguuu.........................................    1     0    seq
.............aagacauggguagugaga..............................................    6     0    seq
.............aagacauggguagugagau.............................................    7     0    seq
.............aagacauggguagugagaug............................................    11    0    seq
..............agacauggguagugagau.............................................    3     0    seq
..............agacauggguagugagaug............................................    5     0    seq
...............gacauggguagugagaug............................................    2     0    seq
.............................................ccuugcuacucugucuuu..............    2     0    seq
.............................................ccuugcuacucugucuuuu.............    16    0    seq
.............................................ccuugcuacucugucuuuug............    125   0    seq
.............................................ccuugcuacucugucuuuugc...........    420   0    seq
.............................................ccuugcuacucugucuuuugca..........    700   0    seq
.............................................ccuugcuacucugucuuuugcau.........    163   0    seq
.............................................ccuugcuacucugucuuuugcaug........    1     0    seq
..............................................cuugcuacucugucuuuugc...........    2     0    seq
..............................................cuugcuacucugucuuuugca..........    3     0    seq
..............................................cuugcuacucugucuuuugcau.........    2     0    seq
...............................................uugcuacucugucuuuugc...........    6     0    seq
...............................................uugcuacucugucuuuugca..........    21    0    seq
...............................................uugcuacucugucuuuugcau.........    105   0    seq
...............................................uugcuacucugucuuuugcaug........    156   0    seq
...............................................uugcuacucugucuuuugcauga.......    1     0    seq
................................................ugcuacucugucuuuugc...........    1     0    seq
................................................ugcuacucugucuuuugca..........    1     0    seq
................................................ugcuacucugucuuuugcaug........    3     0    seq
.................................................gcuacucugucuuuugcau.........    1     0    seq
```
