# Supplementary material for: Identification of Conserved and Novel MicroRNAs in the Pacific Oyster Crassostrea gigas by Deep Sequencing
Source: PLoS One. 2014 Aug 19;9(8):e104371. doi: 10.1371/journal.pone.0104371 (PMC4138081; doi:10.1371/journal.pone.0104371)
Supplement: File S2 — The compressed/ZIP file archive for the predicted precursors' secondary structures and reads alignment. (ZIP) [file pone.0104371.s010.zip › second structure and reads alignment for oyster miRNAs/conserved in table S4/cgi-miR-72.pdf]

miRBase precursor : cgi-miR-72
Total read count : 2633617
cgi-miR-72-5p read count : 2627124
cgi-miR-72-3p read count : 6488
remaining reads : 5

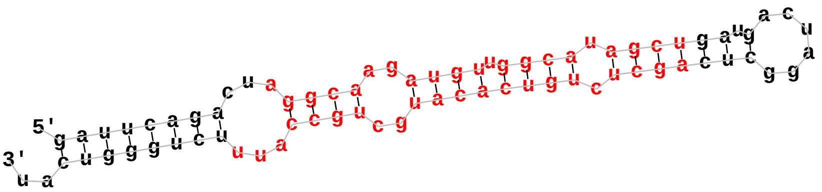

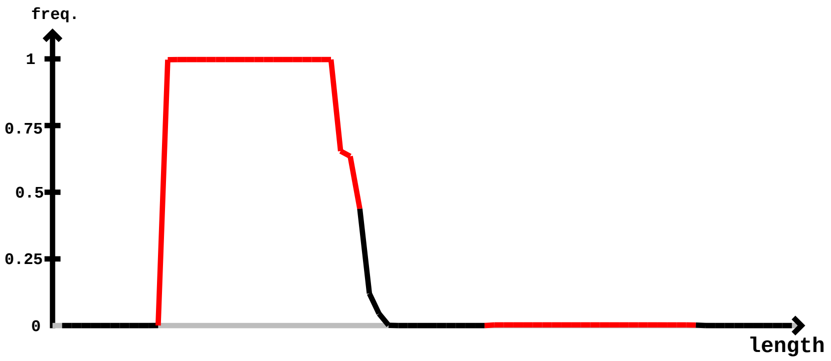

cgi-miR-72-5p

cgi-miR-72-3p

5'- gauucagacuaggcaagauguuggcauagcugaugacuaggcucagcucugucacaugcugccauuucugggucau -3'	exp		
((((((((...((((..((((.((((.((((((.(......))))))).))))))))..))))...))))))))..	reads	mm	sample
...ucagacuaggcaagaugu...	1	0	seq
.......acuaggcaagauguuggcaua..	1	0	seq
........cuaggcaagauguuggcau...	2	0	seq
........cuaggcaagauguuggcaua..	6	0	seq
........cuaggcaagauguuggcauagc..	1	0	seq
.........uaggcaagauguuggcau...	10	0	seq
.........uaggcaagauguuggcaua..	270	0	seq
.........uaggcaagauguuggcauag...	14	0	seq
.........uaggcaagauguuggcauagc..	131	0	seq
.........uaggcaagauguuggcauagcu...	165	0	seq
.........uaggcaagauguuggcauagcug..	72	0	seq
.........uaggcaagauguuggcauagcuga...	1	0	seq
..........aggcaagauguuggcaua..	904037	0	seq
..........aggcaagauguuggcauag...	50852	0	seq
..........aggcaagauguuggcauagc..	518282	0	seq
..........aggcaagauguuggcauagcu...	835512	0	seq
..........aggcaagauguuggcauagcug..	198559	0	seq
..........aggcaagauguuggcauagcuga...	114900	0	seq
..........aggcaagauguuggcauagcugau..	2575	0	seq
..........aggcaagauguuggcauagcugaug...	18	0	seq
..........aggcaagauguuggcauagcugauga..	3	0	seq
..........aggcaagauguuggcauagcugaugac.......................................	2	0	seq
..........aggcaagauguuggcauagcugaugacua.....................................	1	0	seq
...........ggcaagauguuggcauag...	35	0	seq
...........ggcaagauguuggcauagc..	263	0	seq
...........ggcaagauguuggcauagcu...	730	0	seq
...........ggcaagauguuggcauagcug..	197	0	seq
...........ggcaagauguuggcauagcuga...	207	0	seq
...........ggcaagauguuggcauagcugau..	3	0	seq
............gcaagauguuggcauagc..	52	0	seq
............gcaagauguuggcauagcu...	125	0	seq
............gcaagauguuggcauagcug..	25	0	seq
............gcaagauguuggcauagcuga...	6	0	seq
.............caagauguuggcauagcu...	35	0	seq

```
          cgi-miR-72-5p
                                            cgi-miR-72-3p
gauucagacuaggcaagauguuggcauagcugaugacuaggcucagcucugucacaugcugccauuucugggucau

.............caagauguuggcauagcug............................................   10     0     seq
.............caagauguuggcauagcuga...........................................   7      0     seq
..............aagauguuggcauagcug............................................   9      0     seq
..............aagauguuggcauagcuga...........................................   3      0     seq
...............agauguuggcauagcuga...........................................   7      0     seq
...........................................cagcucugucacaugcugccau...........   1      0     seq
............................................agcucugucacaugcugc..............   84     0     seq
............................................agcucugucacaugcugcc.............   55     0     seq
............................................agcucugucacaugcugcca............   113    0     seq
............................................agcucugucacaugcugccau...........   1347   0     seq
............................................agcucugucacaugcugccauu..........   4436   0     seq
............................................agcucugucacaugcugccauuu.........   158    0     seq
.............................................gcucugucacaugcugcc.............   2      0     seq
.............................................gcucugucacaugcugcca............   1      0     seq
.............................................gcucugucacaugcugccau...........   50     0     seq
.............................................gcucugucacaugcugccauu..........   218    0     seq
.............................................gcucugucacaugcugccauuu.........   14     0     seq
..............................................cucugucacaugcugccau...........   1      0     seq
...............................................ucugucacaugcugccau...........   1      0     seq
...............................................ucugucacaugcugccauu..........   5      0     seq
...............................................ucugucacaugcugccauuu.........   2      0     seq
```
