# Supplementary material for: Identification of Conserved and Novel MicroRNAs in the Pacific Oyster Crassostrea gigas by Deep Sequencing
Source: PLoS One. 2014 Aug 19;9(8):e104371. doi: 10.1371/journal.pone.0104371 (PMC4138081; doi:10.1371/journal.pone.0104371)
Supplement: File S2 — The compressed/ZIP file archive for the predicted precursors' secondary structures and reads alignment. (ZIP) [file pone.0104371.s010.zip › second structure and reads alignment for oyster miRNAs/conserved in table S4/cgi-miR-745a.pdf]

miRBase precursor : cgi-miR-745a
Total read count : 418330
cgi-miR-745a-5p read count: 958
cgi-miR-745a-3p read count: 417371
remaining reads : 1

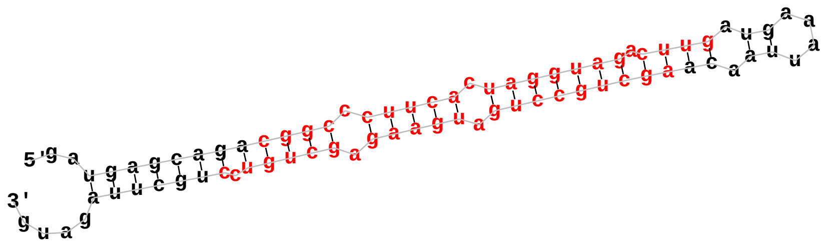

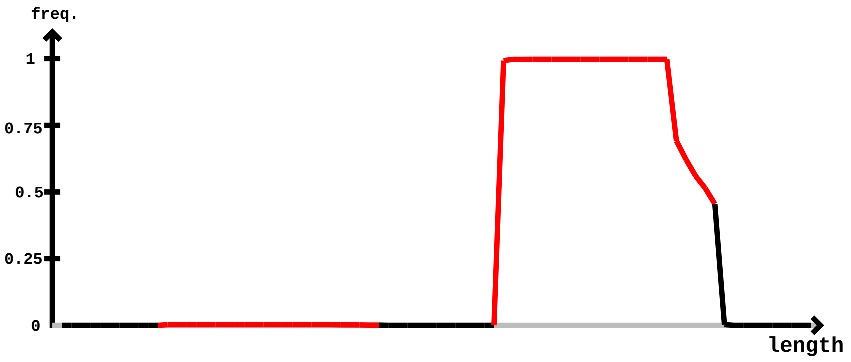


```
                          cgi-miR-745a-5p
                                                              cgi-miR-745a-3p
5'- gaugagcagacggcccuucacuagguagacuugaugaaauuaacaagcugccugaugaagagcuguccugcuuagaug   -3'    exp
    ..((((((((((((.(((((.(((((((.((((.((....)).))))))))))).))))).))))).)))))))....   reads     mm        sample
    .........acggcccuucacuaggua...................................................   9         0         seq
    .........acggcccuucacuagguag..................................................   1         0         seq
    .........acggcccuucacuagguagac................................................   1         0         seq
    .........acggcccuucacuagguagacu...............................................   3         0         seq
    ..........cggcccuucacuagguag..................................................   224       0         seq
    ..........cggcccuucacuagguaga.................................................   47        0         seq
    ..........cggcccuucacuagguagac................................................   92        0         seq
    ..........cggcccuucacuagguagacu...............................................   194       0         seq
    ..........cggcccuucacuagguagacuu..............................................   41        0         seq
    ..........cggcccuucacuagguagacuug.............................................   336       0         seq
    ..........cggcccuucacuagguagacuuga............................................   1         0         seq
    ...........ggcccuucacuagguaga.................................................   1         0         seq
    ...........ggcccuucacuagguagac................................................   1         0         seq
    ...........ggcccuucacuagguagacuug.............................................   7         0         seq
    ..........................................acaagcugccugaugaagagcugucc..........   1         0         seq
    ...........................................caagcugccugaugaagagcug.............   1         0         seq
    ............................................aagcugccugaugaagag................   13        0         seq
    ............................................aagcugccugaugaagagc...............   9         0         seq
    ............................................aagcugccugaugaagagcu..............   6         0         seq
    ............................................aagcugccugaugaagagcug.............   2         0         seq
    ............................................aagcugccugaugaagagcugu............   6         0         seq
    ............................................aagcugccugaugaagagcuguc...........   12        0         seq
    ............................................aagcugccugaugaagagcugucc..........   22        0         seq
    .............................................agcugccugaugaagagc...............   128075    0         seq
    .............................................agcugccugaugaagagcu..............   28938     0         seq
    .............................................agcugccugaugaagagcug.............   25590     0         seq
    .............................................agcugccugaugaagagcugu............   19032     0         seq
    .............................................agcugccugaugaagagcuguc...........   24367     0         seq
    .............................................agcugccugaugaagagcugucc..........   188094    0         seq
    .............................................agcugccugaugaagagcuguccu.........   1096      0         seq
    ..............................................gcugccugaugaagagcu..............   245       0         seq
    ..............................................gcugccugaugaagagcug.............   265       0         seq
    ..............................................gcugccugaugaagagcugu............   166       0         seq
    ..............................................gcugccugaugaagagcuguc...........   220       0         seq
```

```
          cgi-miR-745a-5p
                                             cgi-miR-745a-3p
gaugagcagacggcccuucacuagguagacuugaugaaauuaacaagcugccugaugaagagcuguccugcuuagaug

..............................................gcugccugaugaagagcuguccc..........    1073       0          seq
..............................................gcugccugaugaagagcuguccu.........    7          0          seq
...............................................cugccugaugaagagcuguc...........    2          0          seq
...............................................cugccugaugaagagcugucc..........    2          0          seq
................................................ugccugaugaagagcugu............    6          0          seq
................................................ugccugaugaagagcuguc...........    10         0          seq
................................................ugccugaugaagagcugucc..........    104        0          seq
................................................ugccugaugaagagcuguccu.........    1          0          seq
.................................................gccugaugaagagcugucc..........    1          0          seq
..................................................ccugaugaagagcugucc..........    6          0          seq
```
