# Supplementary material for: Identification of Conserved and Novel MicroRNAs in the Pacific Oyster Crassostrea gigas by Deep Sequencing
Source: PLoS One. 2014 Aug 19;9(8):e104371. doi: 10.1371/journal.pone.0104371 (PMC4138081; doi:10.1371/journal.pone.0104371)
Supplement: File S2 — The compressed/ZIP file archive for the predicted precursors' secondary structures and reads alignment. (ZIP) [file pone.0104371.s010.zip › second structure and reads alignment for oyster miRNAs/conserved in table S4/cgi-miR-745b.pdf]

miRBase precursor : cgi-miR-745b
Total read count : 35874
cgi-miR-745b-5p read count : 458
cgi-miR-745b-3p read count : 35389
remaining reads : 27

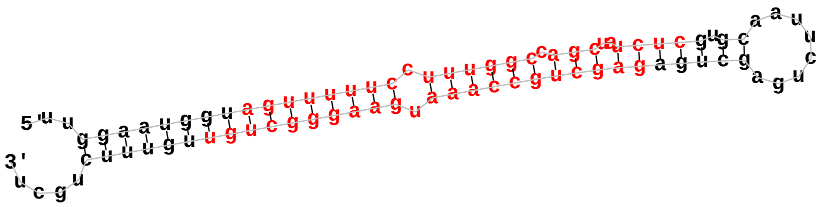

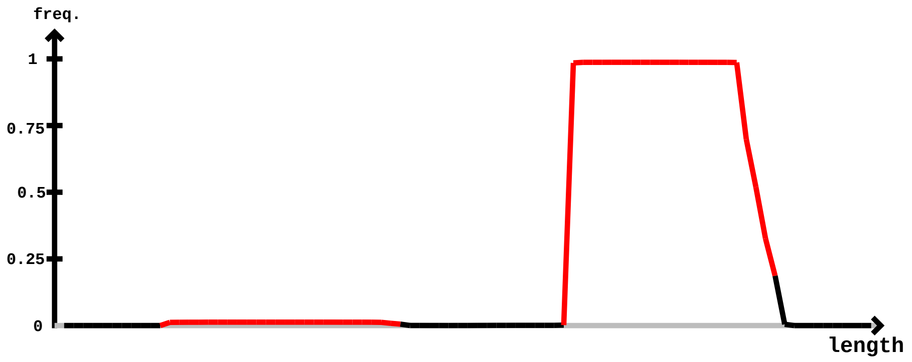


```
                                                          cgi-miR-745b-3p
                 cgi-miR-745b-5p
5'- uuggaaugguaguuuuuccuuuggccagcuaucucgugcaauucugagcugagagcugccaaaugaagggcuguuguuucugcu   -3'    exp
    ..((((((((((((((((.((((((.(((..(((((.((........)))))))))))))))).))))))))))))))))....   reads      mm          sample
    .........aguuuuuccuuuggccag.........................................................   1          0           seq
    .........aguuuuuccuuuggccagcua......................................................   5          0           seq
    .........aguuuuuccuuuggccagcuau.....................................................   17         0           seq
    .........aguuuuuccuuuggccagcuauc....................................................   128        0           seq
    .........aguuuuuccuuuggccagcuaucu...................................................   101        0           seq
    .........aguuuuuccuuuggccagcuaucuc..................................................   174        0           seq
    .........aguuuuuccuuuggccagcuaucucgu................................................   2          0           seq
    .........aguuuuuccuuuggccagcuaucucgug...............................................   1          0           seq
    ..........guuuuuccuuuggccagcu.......................................................   1          0           seq
    ..........guuuuuccuuuggccagcuau.....................................................   1          0           seq
    ..........guuuuuccuuuggccagcuauc....................................................   1          0           seq
    ..........guuuuuccuuuggccagcuaucu...................................................   4          0           seq
    ..........guuuuuccuuuggccagcuaucuc..................................................   4          0           seq
    ..........guuuuuccuuuggccagcuaucucgugc..............................................   1          0           seq
    ...........uuuuuccuuuggccagcuauc....................................................   3          0           seq
    ...........uuuuuccuuuggccagcuaucu...................................................   3          0           seq
    ...........uuuuuccuuuggccagcuaucucgugca.............................................   1          0           seq
    ............uuuccuuuggccagcuaucucgu.................................................   1          0           seq
    .............uuccuuuggccagcuauc.....................................................   1          0           seq
    .............uuccuuuggccagcuaucu....................................................   3          0           seq
    .............uuccuuuggccagcuaucuc...................................................   2          0           seq
    .............uuccuuuggccagcuaucucgugc...............................................   3          0           seq
    .................................ucgugcaauucugagcugagagcu...........................   1          0           seq
    ....................................ugcaauucugagcugagagcugccaaa.....................   1          0           seq
    .......................................aauucugagcugagagcugccaaaug...................   2          0           seq
    ........................................auucugagcugagagcugccaaau....................   1          0           seq
    ........................................auucugagcugagagcugccaaauga..................   1          0           seq
    .........................................uucugagcugagagcugccaaaugaagggc.............   1          0           seq
    ..........................................ucugagcugagagcugccaaaugaagg...............   5          0           seq
    ..........................................ucugagcugagagcugccaaaugaagggc.............   1          0           seq
    ...........................................cugagcugagagcugccaaaugaagg...............   2          0           seq
    ...........................................cugagcugagagcugccaaaugaaggg..............   2          0           seq
    ............................................ugagcugagagcugccaaaugaa.................   1          0           seq
    ............................................ugagcugagagcugccaaaugaag................   1          0           seq
```

```
                                          cgi-miR-745b-3p
          cgi-miR-745b-5p
uuggaaugguaguuuuuccuuuggccagcuaucucgugcaauucugagcugagagcugccaaaugaagggcuguuguuucugcu

............................................ugagcugagagcugccaaaugaagg...............    2       0       seq
............................................ugagcugagagcugccaaaugaaggg..............    1       0       seq
............................................ugagcugagagcugccaaaugaagggc.............    2       0       seq
.............................................gagcugagagcugccaaaugaagggc.............    1       0       seq
.............................................gagcugagagcugccaaaugaagggcug...........    1       0       seq
..............................................agcugagagcugccaaaugaaggg..............    1       0       seq
...................................................agagcugccaaaugaagg...............    12      0       seq
...................................................agagcugccaaaugaaggg..............    4       0       seq
...................................................agagcugccaaaugaagggc.............    3       0       seq
...................................................agagcugccaaaugaagggcu............    6       0       seq
...................................................agagcugccaaaugaagggcugu..........    7       0       seq
...................................................agagcugccaaaugaagggcuguu.........    1       0       seq
....................................................gagcugccaaaugaaggg..............    10319   0       seq
....................................................gagcugccaaaugaagggc.............    6385    0       seq
....................................................gagcugccaaaugaagggcu............    6889    0       seq
....................................................gagcugccaaaugaagggcug...........    5014    0       seq
....................................................gagcugccaaaugaagggcugu..........    6497    0       seq
....................................................gagcugccaaaugaagggcuguu.........    167     0       seq
.....................................................agcugccaaaugaagggc.............    29      0       seq
.....................................................agcugccaaaugaagggcu............    30      0       seq
.....................................................agcugccaaaugaagggcug...........    9       0       seq
.....................................................agcugccaaaugaagggcugu..........    8       0       seq
......................................................gcugccaaaugaagggcu............    2       0       seq
......................................................gcugccaaaugaagggcugu..........    3       0       seq
........................................................ugccaaaugaagggcugu..........    3       0       seq
........................................................ugccaaaugaagggcuguu.........    1       0       seq
```
