# Supplementary material for: Identification of Conserved and Novel MicroRNAs in the Pacific Oyster Crassostrea gigas by Deep Sequencing
Source: PLoS One. 2014 Aug 19;9(8):e104371. doi: 10.1371/journal.pone.0104371 (PMC4138081; doi:10.1371/journal.pone.0104371)
Supplement: File S2 — The compressed/ZIP file archive for the predicted precursors' secondary structures and reads alignment. (ZIP) [file pone.0104371.s010.zip › second structure and reads alignment for oyster miRNAs/conserved in table S4/cgi-miR-750.pdf]

miRBase precursor : cgi-miR-750
Total read count : 59784
cgi-miR-750-5p read count : 771
cgi-miR-750-3p read count : 58962
remaining reads : 51

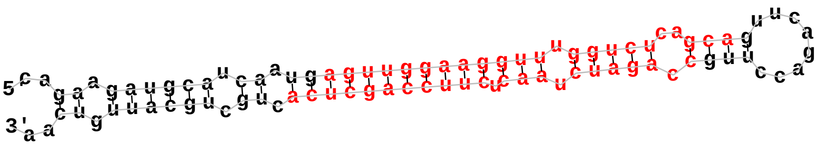

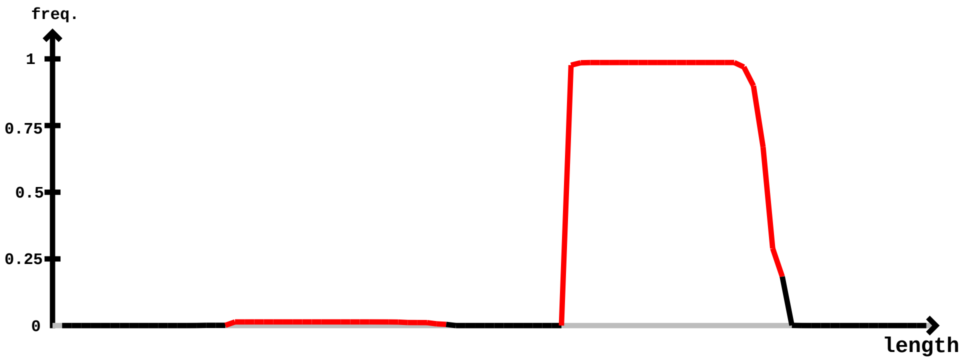

cgi-miR-750-3p

cgi-miR-750-5p

```
5'- cagaagaugcaucaaugaguuggaagguuuggucucagcaguucagaccuugccagaucuaacucuuccagcucacugcugcauugucaa   -3'    exp
    ..((.((((((.((.((((((((((((((.(((((..((((........)))).))))).))).))))))))))).)).)))))).))..    reads      mm         sample
    ...........ucaaugaguuggaagguu.............................................................    1          0          seq
    .............aaugaguuggaagguuug...........................................................    2          0          seq
    .............aaugaguuggaagguuugguc........................................................    5          0          seq
    .............aaugaguuggaagguuuggucu.......................................................    2          0          seq
    ..............augaguuggaagguuugg..........................................................    7          0          seq
    ..............augaguuggaagguuuggu.........................................................    2          0          seq
    ..............augaguuggaagguuugguc........................................................    21         0          seq
    ..............augaguuggaagguuuggucu.......................................................    11         0          seq
    ...............ugaguuggaagguuugguc........................................................    1          0          seq
    ...............ugaguuggaagguuuggucu.......................................................    2          0          seq
    ................gaguuggaagguuuggucucagc...................................................    1          0          seq
    .................aguuggaagguuuggucu.......................................................    89         0          seq
    .................aguuggaagguuuggucuc......................................................    12         0          seq
    .................aguuggaagguuuggucuca.....................................................    11         0          seq
    .................aguuggaagguuuggucucag....................................................    270        0          seq
    .................aguuggaagguuuggucucagc...................................................    120        0          seq
    .................aguuggaagguuuggucucagca..................................................    257        0          seq
    .................aguuggaagguuuggucucagcag.................................................    1          0          seq
    ..................guuggaagguuuggucucag....................................................    1          0          seq
    ...................uuggaagguuuggucucagc...................................................    2          0          seq
    ...................uuggaagguuuggucucagca..................................................    2          0          seq
    ...................uuggaagguuuggucucagcag.................................................    1          0          seq
    ....................uggaagguuuggucucagca..................................................    1          0          seq
    ....................................................ccagaucuaacucuucca....................    1006       0          seq
    ....................................................ccagaucuaacucuuccag...................    4192       0          seq
    ....................................................ccagaucuaacucuuccagc..................    13624      0          seq
    ....................................................ccagaucuaacucuuccagcu.................    22596      0          seq
    ....................................................ccagaucuaacucuuccagcuc................    6245       0          seq
    ....................................................ccagaucuaacucuuccagcuca...............    10687      0          seq
    ....................................................ccagaucuaacucuuccagcucac..............    27         0          seq
    ....................................................ccagaucuaacucuuccagcucacu.............    3          0          seq
    .....................................................cagaucuaacucuuccag...................    32         0          seq
    .....................................................cagaucuaacucuuccagc..................    88         0          seq
    .....................................................cagaucuaacucuuccagcu.................    106        0          seq
```

```
                                   cgi-miR-750-3p
                 cgi-miR-750-5p
cagaagaugcaucaaugaguuggaagguuuggucucagcaguucagaccuugccagaucuaacucuuccagcucacugcugcauugucaa

.....................................................cagaucuaacucuuccagcuc................    86     0     seq
.....................................................cagaucuaacucuuccagcuca...............    201    0     seq
.....................................................cagaucuaacucuuccagcucac..............    20     0     seq
......................................................agaucuaacucuuccagc..................    8      0     seq
......................................................agaucuaacucuuccagcu.................    11     0     seq
......................................................agaucuaacucuuccagcuc................    2      0     seq
......................................................agaucuaacucuuccagcuca...............    26     0     seq
........................................................aucuaacucuuccagcuca...............    1      0     seq
.........................................................ucuaacucuuccagcuca...............    1      0     seq
```
