# Supplementary material for: Identification of Conserved and Novel MicroRNAs in the Pacific Oyster Crassostrea gigas by Deep Sequencing
Source: PLoS One. 2014 Aug 19;9(8):e104371. doi: 10.1371/journal.pone.0104371 (PMC4138081; doi:10.1371/journal.pone.0104371)
Supplement: File S2 — The compressed/ZIP file archive for the predicted precursors' secondary structures and reads alignment. (ZIP) [file pone.0104371.s010.zip › second structure and reads alignment for oyster miRNAs/conserved in table S4/cgi-miR-8.pdf]

miRBase precursor : cgi-miR-8
Total read count : 131365
cgi-miR-8-5p read count : 2858
cgi-miR-8-3p read count : 128496
remaining reads : 11

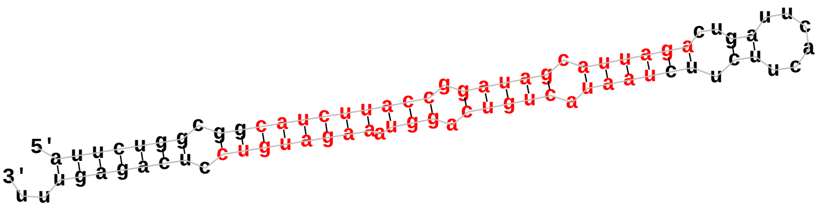

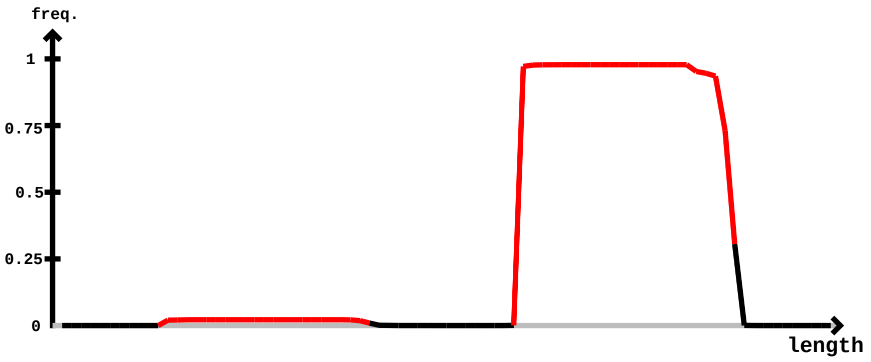


```
                                                   cgi-miR-8-3p
             cgi-miR-8-5p
5'- auucuggcggcaucuuaccggauagcauuagacugauucacuucuucuaauacugucagguaaagaugucucagaguuu   -3'    exp
    (((((((.(((((((((((.(((((.((((((..((......)).)))))).))))).))).)))))))).)))))))..    reads      mm         sample
    .........gcaucuuaccggauagcauuag.................................................    1          0          seq
    .........gcaucuuaccggauagcauuagac...............................................    1          0          seq
    .........gcaucuuaccggauagcauuagacu..............................................    1          0          seq
    ..........caucuuaccggauagcau....................................................    11         0          seq
    ..........caucuuaccggauagcauu...................................................    63         0          seq
    ..........caucuuaccggauagcauua..................................................    352        0          seq
    ..........caucuuaccggauagcauuag.................................................    1132       0          seq
    ..........caucuuaccggauagcauuaga................................................    1025       0          seq
    ..........caucuuaccggauagcauuagac...............................................    28         0          seq
    ..........caucuuaccggauagcauuagacu..............................................    29         0          seq
    ..........caucuuaccggauagcauuagacug.............................................    1          0          seq
    ..........caucuuaccggauagcauuagacuga............................................    2          0          seq
    ..........caucuuaccggauagcauuagacugau...........................................    1          0          seq
    ..........caucuuaccggauagcauuagacugauu..........................................    5          0          seq
    ..........caucuuaccggauagcauuagacugauuc.........................................    1          0          seq
    ...........aucuuaccggauagcauu...................................................    2          0          seq
    ...........aucuuaccggauagcauua..................................................    14         0          seq
    ...........aucuuaccggauagcauuag.................................................    40         0          seq
    ...........aucuuaccggauagcauuaga................................................    11         0          seq
    ...........aucuuaccggauagcauuagac...............................................    10         0          seq
    ...........aucuuaccggauagcauuagacu..............................................    3          0          seq
    ............ucuuaccggauagcauua..................................................    5          0          seq
    ............ucuuaccggauagcauuag.................................................    28         0          seq
    ............ucuuaccggauagcauuaga................................................    34         0          seq
    ............ucuuaccggauagcauuagac...............................................    18         0          seq
    ............ucuuaccggauagcauuagacu..............................................    38         0          seq
    ............ucuuaccggauagcauuagacugauu..........................................    1          0          seq
    .............cuuaccggauagcauuaga................................................    2          0          seq
    .............cuuaccggauagcauuagacu..............................................    1          0          seq
    ..............uuaccggauagcauuaga................................................    1          0          seq
    ..............uuaccggauagcauuagac...............................................    1          0          seq
    ..............uuaccggauagcauuagacug.............................................    1          0          seq
    ..............uuaccggauagcauuagacugau...........................................    1          0          seq
    ..............uuaccggauagcauuagacugauu..........................................    3          0          seq
```

cgi-miR-8-3p

cgi-miR-8-5p

auucuggcggcaucuuaccggauagcauuagacugauucacuucuucuaauacugucagguaaagauguccucagaguuu

..............uuaccggauagcauuagacugauucacu......................................	1	0	seq
................accggauagcauuagacug...	1	0	seq
....ucuaauacugucagguaaagau.............	1	0	seq
....ucuaauacugucagguaaagaug............	13	0	seq
....ucuaauacugucagguaaagauguc..........	4	0	seq
....cuaauacugucagguaaag...............	3	0	seq
....cuaauacugucagguaaagau.............	2	0	seq
....cuaauacugucagguaaagaug............	16	0	seq
....cuaauacugucagguaaagaugu...........	42	0	seq
....cuaauacugucagguaaagauguc..........	13	0	seq
....uaauacugucagguaaag...............	3332	0	seq
....uaauacugucagguaaaga..............	869	0	seq
....uaauacugucagguaaagau.............	1398	0	seq
....uaauacugucagguaaagaug............	26655	0	seq
....uaauacugucagguaaagaugu...........	55576	0	seq
....uaauacugucagguaaagauguc..........	39656	0	seq
....uaauacugucagguaaagauguccu........	74	0	seq
....uaauacugucagguaaagauguccu........	3	0	seq
....aauacugucagguaaaga..............	5	0	seq
....aauacugucagguaaagau.............	4	0	seq
....aauacugucagguaaagaug............	105	0	seq
....aauacugucagguaaagaugu...........	237	0	seq
....aauacugucagguaaagauguc..........	290	0	seq
....aauacugucagguaaagaugucc.........	12	0	seq
....auacugucagguaaagau.............	3	0	seq
....auacugucagguaaagaug............	17	0	seq
....auacugucagguaaagaugu...........	36	0	seq
....auacugucagguaaagauguc..........	81	0	seq
....auacugucagguaaagauguccu........	5	0	seq
....uacugucagguaaagaug............	4	0	seq
....uacugucagguaaagaugu...........	7	0	seq
....uacugucagguaaagauguc..........	10	0	seq
....uacugucagguaaagauguccu........	2	0	seq
....uacugucagguaaagauguccuca......	1	0	seq
....acugucagguaaagaugu...........	11	0	seq
....acugucagguaaagauguc..........	7	0	seq
....cugucagguaaagauguc..........	2	0	seq
