# Supplementary material for: Identification of Conserved and Novel MicroRNAs in the Pacific Oyster Crassostrea gigas by Deep Sequencing
Source: PLoS One. 2014 Aug 19;9(8):e104371. doi: 10.1371/journal.pone.0104371 (PMC4138081; doi:10.1371/journal.pone.0104371)
Supplement: File S2 — The compressed/ZIP file archive for the predicted precursors' secondary structures and reads alignment. (ZIP) [file pone.0104371.s010.zip › second structure and reads alignment for oyster miRNAs/conserved in table S4/cgi-miR-87.pdf]

miRBase precursor : cgi-miR-87
Total read count : 24161
cgi-miR-87-5p read count : 674
cgi-miR-87-3p read count : 23484
remaining reads : 3

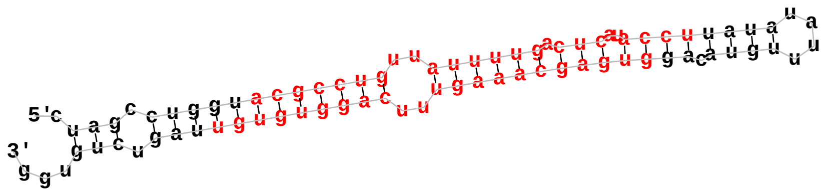

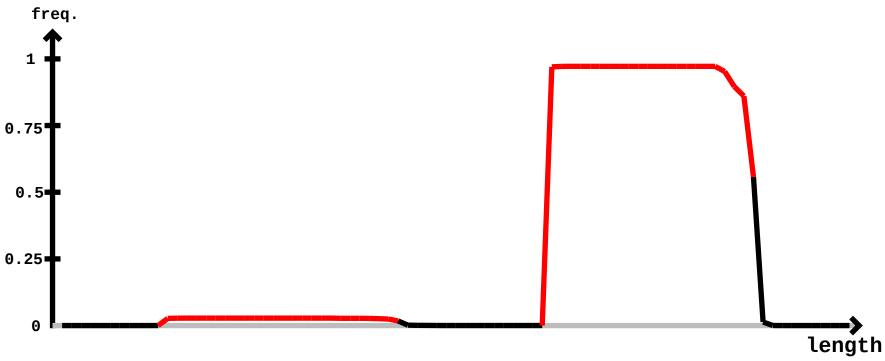

cgi-miR-87-5p
cgi-miR-87-3p

```
5'- cuagccugguacgccuguuauuuugacucauaccuuauauauuuguacaggugagcaaaguuucagguguguuagucugugg   -3'    exp
    .(((.((((((((((((..((((((.(((..((((((((....)))).)))))))))))))..))))))))))))).)))...    reads      mm         sample
    .........uacgccuguuauuuugac.......................................................    1          0          seq
    .........uacgccuguuauuuugacucauacc................................................    1          0          seq
    ..........acgccuguuauuuugacu......................................................    16         0          seq
    ..........acgccuguuauuuugacucau...................................................    12         0          seq
    ..........acgccuguuauuuugacucaua..................................................    20         0          seq
    ..........acgccuguuauuuugacucauac.................................................    35         0          seq
    ..........acgccuguuauuuugacucauacc................................................    168        0          seq
    ..........acgccuguuauuuugacucauaccu...............................................    359        0          seq
    ..........acgccuguuauuuugacucauaccuu..............................................    19         0          seq
    ..........acgccuguuauuuugacucauaccuua.............................................    6          0          seq
    ..........acgccuguuauuuugacucauaccuuau............................................    8          0          seq
    ..........acgccuguuauuuugacucauaccuuaua...........................................    4          0          seq
    ..........acgccuguuauuuugacucauaccuuauau..........................................    1          0          seq
    ...........cgccuguuauuuugacucaua..................................................    1          0          seq
    ...........cgccuguuauuuugacucauacc................................................    4          0          seq
    ...........cgccuguuauuuugacucauaccu...............................................    14         0          seq
    ...........cgccuguuauuuugacucauaccuu..............................................    1          0          seq
    ............gccuguuauuuugacucauacc................................................    1          0          seq
    ............gccuguuauuuugacucauaccu...............................................    3          0          seq
    .....................................uauauuuguacaggugag...........................    2          0          seq
    ...............................................caggugagcaaaguuucaggugug...........    1          0          seq
    .................................................ggugagcaaaguuucaggugugu..........    2          0          seq
    ..................................................gugagcaaaguuucaggu..............    466        0          seq
    ..................................................gugagcaaaguuucaggug.............    1360       0          seq
    ..................................................gugagcaaaguuucaggugu............    867        0          seq
    ..................................................gugagcaaaguuucaggugug...........    7311       0          seq
    ..................................................gugagcaaaguuucaggugugu..........    13108      0          seq
    ..................................................gugagcaaaguuucagguguguu.........    318        0          seq
    ..................................................gugagcaaaguuucagguguguua........    4          0          seq
    ...................................................ugagcaaaguuucaggug.............    3          0          seq
    ...................................................ugagcaaaguuucaggugu............    2          0          seq
    ...................................................ugagcaaaguuucaggugug...........    15         0          seq
    ...................................................ugagcaaaguuucaggugugu..........    16         0          seq
    ...................................................ugagcaaaguuucagguguguu.........    2          0          seq
```

```
         cgi-miR-87-5p
                                         cgi-miR-87-3p
cuagccugguacgccuguuauuuugacucauaccuuauauauuuguacaggugagcaaaguuucagguguguuagucugugg
```

....gagcaaaguuucaggugug...........	2	0	seq
....gagcaaaguuucaggugugu..........	7	0	seq
....agcaaaguuucaggugugu u.........	1	0	seq
