# Supplementary material for: Identification of Conserved and Novel MicroRNAs in the Pacific Oyster Crassostrea gigas by Deep Sequencing
Source: PLoS One. 2014 Aug 19;9(8):e104371. doi: 10.1371/journal.pone.0104371 (PMC4138081; doi:10.1371/journal.pone.0104371)
Supplement: File S2 — The compressed/ZIP file archive for the predicted precursors' secondary structures and reads alignment. (ZIP) [file pone.0104371.s010.zip › second structure and reads alignment for oyster miRNAs/conserved in table S4/cgi-miR-92a.pdf]

miRBase precursor : cgi-miR-92a
Total read count : 58368
cgi-miR-92a-5p read count : 5750
cgi-miR-92a-3p read count : 52617
remaining reads : 1

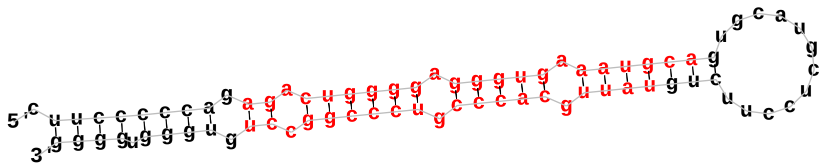

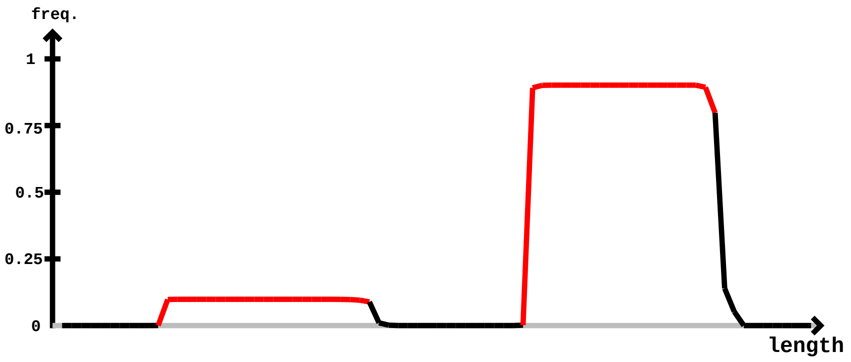


```
                          cgi-miR-92a-5p
                                               cgi-miR-92a-3p
5'- cuucccccagagacuggggagggugaaaugcagugcaugcuccuucuguauugcacccgucccggccugugggugggg  -3'   exp
    .((((((((.((.((((((.(((((.(((((((............))))))).))))).)))))).)).)))).))))   reads     mm        sample
    ........agagacuggggagggugaaau.................................................   1         0         seq
    .........gagacuggggagggugaaaug................................................   2         0         seq
    .........gagacuggggagggugaaaugc...............................................   12        0         seq
    ..........agacuggggagggugaaa..................................................   16        0         seq
    ..........agacuggggagggugaaau.................................................   40        0         seq
    ..........agacuggggagggugaaaug................................................   135       0         seq
    ..........agacuggggagggugaaaugc...............................................   359       0         seq
    ..........agacuggggagggugaaaugca..............................................   4555      0         seq
    ..........agacuggggagggugaaaugcag.............................................   505       0         seq
    ..........agacuggggagggugaaaugcagu............................................   87        0         seq
    ..........agacuggggagggugaaaugcagugc..........................................   1         0         seq
    ...........gacuggggagggugaaaug................................................   1         0         seq
    ...........gacuggggagggugaaaugc...............................................   1         0         seq
    ...........gacuggggagggugaaaugca..............................................   26        0         seq
    ...........gacuggggagggugaaaugcag.............................................   3         0         seq
    ...........gacuggggagggugaaaugcagu............................................   1         0         seq
    ............acuggggagggugaaaugca..............................................   2         0         seq
    ............acuggggagggugaaaugcag.............................................   1         0         seq
    ............acuggggagggugaaaugcagu............................................   1         0         seq
    .............cuggggagggugaaaugc...............................................   1         0         seq
    ........................................uccuucuguauugcacccgucccggccu..........   1         0         seq
    ...............................................guauugcacccgucccgg.............   1         0         seq
    ...............................................guauugcacccgucccggc............   1         0         seq
    ...............................................guauugcacccgucccggcc...........   7         0         seq
    ...............................................guauugcacccgucccggccu..........   66        0         seq
    ...............................................guauugcacccgucccggccug.........   5         0         seq
    ...............................................guauugcacccgucccggccugu........   9         0         seq
    ................................................uauugcacccgucccggc............   465       0         seq
    ................................................uauugcacccgucccggcc...........   5531      0         seq
    ................................................uauugcacccgucccggccu..........   37933     0         seq
    ................................................uauugcacccgucccggccug.........   5020      0         seq
    ................................................uauugcacccgucccggccugu........   2994      0         seq
    .................................................auugcacccgucccggcc...........   38        0         seq
    .................................................auugcacccgucccggccu..........   420       0         seq
```

```
          cgi-miR-92a-5p
                                                      cgi-miR-92a-3p
cuucccccagagacuggggagggugaaaugcagugcaugcuccuucuguauugcacccgucccggccugugggugggg

.................................................auugcacccgucccggccug.........    53         0          seq
.................................................auugcacccgucccggccugu........    32         0          seq
..................................................uugcacccgucccggccu..........    28         0          seq
..................................................uugcacccgucccggccug.........    8          0          seq
..................................................uugcacccgucccggccugu........    6          0          seq
```
