# Supplementary material for: Identification of Conserved and Novel MicroRNAs in the Pacific Oyster Crassostrea gigas by Deep Sequencing
Source: PLoS One. 2014 Aug 19;9(8):e104371. doi: 10.1371/journal.pone.0104371 (PMC4138081; doi:10.1371/journal.pone.0104371)
Supplement: File S2 — The compressed/ZIP file archive for the predicted precursors' secondary structures and reads alignment. (ZIP) [file pone.0104371.s010.zip › second structure and reads alignment for oyster miRNAs/conserved in table S4/cgi-miR-92b.pdf]

miRBase precursor : cgi-miR-92b
Total read count : 246761
cgi-miR-92b-5p read count : 460
cgi-miR-92b-3p read count : 246300
remaining reads : 1

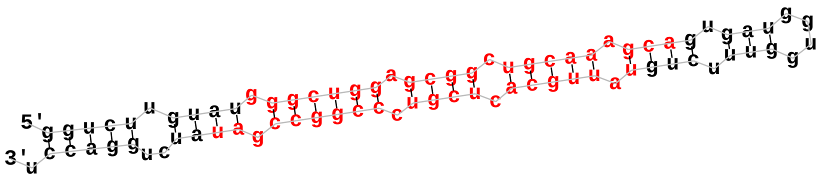

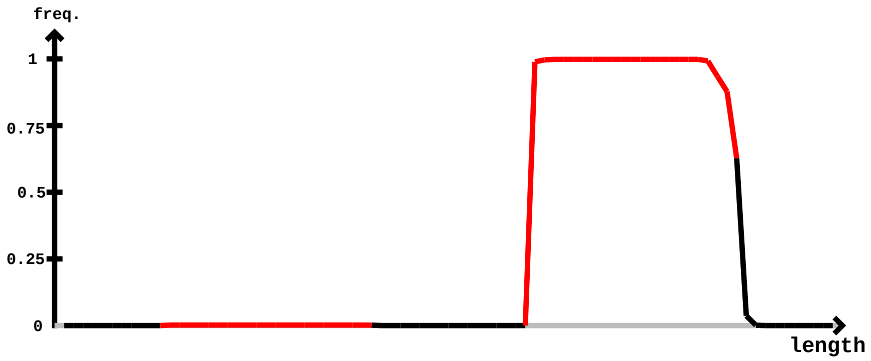

cgi-miR-92b-5p

cgi-miR-92b-3p

```
5'- ggucuuguaugggcuggagcggcugcaaagcagugauggugguuucuguauugcacucgucccggccgauaucuggaccu   -3'    exp
    (((((.((((.((((((.((((.(((((.((((.(((....))).)))).))))).)))).)))))).))))..))))).    reads      mm        sample
    ..........gggcuggagcggcugcaaag..................................................    7          0         seq
    ..........gggcuggagcggcugcaaagc.................................................    65         0         seq
    ..........gggcuggagcggcugcaaagca................................................    382        0         seq
    ..........gggcuggagcggcugcaaagcag...............................................    5          0         seq
    ..........gggcuggagcggcugcaaagcagu..............................................    1          0         seq
    ...........................................uucuguauugcacucgucccggccgau..........    1          0         seq
    ..............................................guauugcacucgucccgg................    1          0         seq
    ..............................................guauugcacucgucccggc...............    1          0         seq
    ..............................................guauugcacucgucccggcc..............    4          0         seq
    ..............................................guauugcacucgucccggccg.............    4          0         seq
    ..............................................guauugcacucgucccggccga............    15         0         seq
    ..............................................guauugcacucgucccggccgau...........    31         0         seq
    ..............................................guauugcacucgucccggccgaua..........    1          0         seq
    ................................................uauugcacucgucccggc..............    1469       0         seq
    ................................................uauugcacucgucccggcc.............    14252      0         seq
    ................................................uauugcacucgucccggccg............    13924      0         seq
    ................................................uauugcacucgucccggccga...........    61315      0         seq
    ................................................uauugcacucgucccggccgau..........    144469     0         seq
    ................................................uauugcacucgucccggccgaua.........    8234       0         seq
    ................................................uauugcacucgucccggccgauau........    304        0         seq
    ................................................uauugcacucgucccggccgauauc.......    1          0         seq
    .................................................auugcacucgucccggcc.............    61         0         seq
    .................................................auugcacucgucccggccg............    54         0         seq
    .................................................auugcacucgucccggccga...........    254        0         seq
    .................................................auugcacucgucccggccgau..........    919        0         seq
    .................................................auugcacucgucccggccgaua.........    478        0         seq
    .................................................auugcacucgucccggccgauau........    21         0         seq
    ..................................................uugcacucgucccggccg............    9          0         seq
    ..................................................uugcacucgucccggccga...........    86         0         seq
    ..................................................uugcacucgucccggccgau..........    355        0         seq
    ..................................................uugcacucgucccggccgaua.........    17         0         seq
    ...................................................ugcacucgucccggccga...........    5          0         seq
    ...................................................ugcacucgucccggccgau..........    3          0         seq
    ....................................................gcacucgucccggccgau..........    12         0         seq
```

cgi-miR-92b-5p

cgi-miR-92b-3p

ggucuuguaugggcuggagcggcugcaaagcagugaugguggguuucuguauugcacucgucccggccgauaucuggaccu

....gcacucgucccggccgaua......... 1 0 seq
