# Supplementary material for: Identification of Conserved and Novel MicroRNAs in the Pacific Oyster Crassostrea gigas by Deep Sequencing
Source: PLoS One. 2014 Aug 19;9(8):e104371. doi: 10.1371/journal.pone.0104371 (PMC4138081; doi:10.1371/journal.pone.0104371)
Supplement: File S2 — The compressed/ZIP file archive for the predicted precursors' secondary structures and reads alignment. (ZIP) [file pone.0104371.s010.zip › second structure and reads alignment for oyster miRNAs/conserved in table S4/cgi-miR-92c.pdf]

miRBase precursor : cgi-miR-92c
Total read count : 146906
cgi-miR-92c-5p read count : 2677
cgi-miR-92c-3p read count : 144211
remaining reads : 18

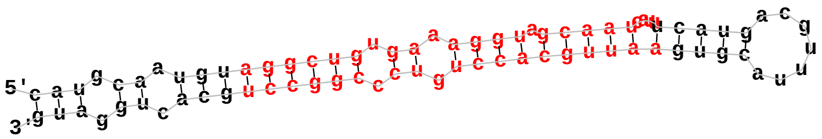

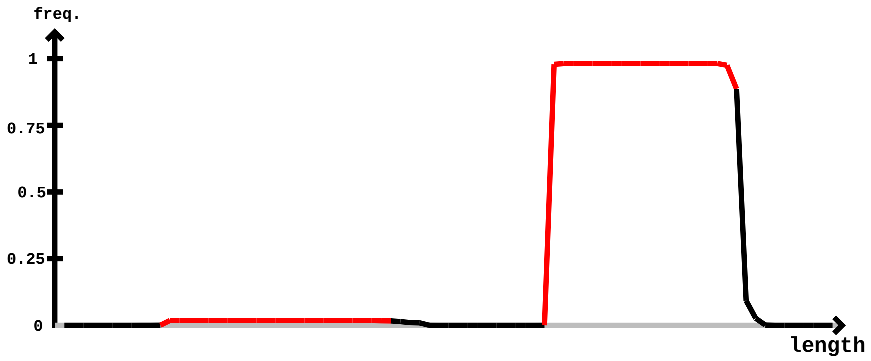

cgi-miR-92c-5p

cgi-miR-92c-3p

```
5'- caugcaauguaggcugugaaagguagcaaucauuucaugacguuuacgugaauugcaccugucccggccugcacuggaug  -3'    exp
    (((.((.(((((((((.((.((((.(((((....(((((.......))))))))))))))).)).))))))))).)).)))    reads      mm     sample
    ......auguaggcugugaaagguagc.....................................................    1          0      seq
    ......auguaggcugugaaagguagca....................................................    1          0      seq
    ......auguaggcugugaaagguagcaa...................................................    3          0      seq
    .......uguaggcugugaaagguagcaa...................................................    2          0      seq
    .......uguaggcugugaaagguagcaau..................................................    6          0      seq
    .........uaggcugugaaagguagcaauc.................................................    4          0      seq
    .........uaggcugugaaagguagcaauca................................................    43         0      seq
    .........uaggcugugaaagguagcaaucauu..............................................    1          0      seq
    ..........aggcugugaaagguagca....................................................    2          0      seq
    ..........aggcugugaaagguagcaa...................................................    7          0      seq
    ..........aggcugugaaagguagcaau..................................................    4          0      seq
    ..........aggcugugaaagguagcaauc.................................................    11         0      seq
    ..........aggcugugaaagguagcaauca................................................    94         0      seq
    ..........aggcugugaaagguagcaaucau...............................................    69         0      seq
    ..........aggcugugaaagguagcaaucauu..............................................    387        0      seq
    ..........aggcugugaaagguagcaaucauuu.............................................    563        0      seq
    ..........aggcugugaaagguagcaaucauuuc............................................    123        0      seq
    ..........aggcugugaaagguagcaaucauuuca...........................................    1365       0      seq
    ..........aggcugugaaagguagcaaucauuucau..........................................    4          0      seq
    .....................................ugacguuuacgugaauugca.......................    1          0      seq
    .....................................ugacguuuacgugaauugcacc.....................    1          0      seq
    .....................................ugacguuuacgugaauugcaccu....................    2          0      seq
    .....................................ugacguuuacgugaauugcaccug...................    1          0      seq
    .................................................gaauugcaccugucccggccu..........    2          0      seq
    .................................................gaauugcaccugucccggccugc........    1          0      seq
    ..................................................aauugcaccugucccggc............    969        0      seq
    ..................................................aauugcaccugucccggcc...........    12953      0      seq
    ..................................................aauugcaccugucccggccu..........    116366     0      seq
    ..................................................aauugcaccugucccggccug.........    9653       0      seq
    ..................................................aauugcaccugucccggccugc........    3692       0      seq
    ..................................................aauugcaccugucccggccugca.......    122        0      seq
    ...................................................auugcaccugucccggcc...........    42         0      seq
    ...................................................auugcaccugucccggccu..........    278        0      seq
    ...................................................auugcaccugucccggccug.........    74         0      seq
```

```
         cgi-miR-92c-5p
                                       cgi-miR-92c-3p
caugcaauguaggcugugaaagguagcaaucauuucaugacguuuacgugaauugcaccugucccggccugcacuggaug

...................................................auugcaccugucccggccugc........    23    0    seq
...................................................auugcaccugucccggccugca.......    5     0    seq
....................................................uugcaccugucccggccu..........    19    0    seq
....................................................uugcaccugucccggccug.........    3     0    seq
....................................................uugcaccugucccggccugc........    2     0    seq
....................................................uugcaccugucccggccugca.......    1     0    seq
.....................................................ugcaccugucccggccug.........    2     0    seq
.....................................................ugcaccugucccggccugc........    3     0    seq
.....................................................ugcaccugucccggccugcac......    1     0    seq
```
