# Supplementary material for: Identification of Conserved and Novel MicroRNAs in the Pacific Oyster Crassostrea gigas by Deep Sequencing
Source: PLoS One. 2014 Aug 19;9(8):e104371. doi: 10.1371/journal.pone.0104371 (PMC4138081; doi:10.1371/journal.pone.0104371)
Supplement: File S2 — The compressed/ZIP file archive for the predicted precursors' secondary structures and reads alignment. (ZIP) [file pone.0104371.s010.zip › second structure and reads alignment for oyster miRNAs/conserved in table S4/cgi-miR-92d.pdf]

miRBase precursor : cgi-miR-92d
Total read count : 174037
cgi-miR-92d-5p read count : 26662
cgi-miR-92d-3p read count : 147375
remaining reads : 0

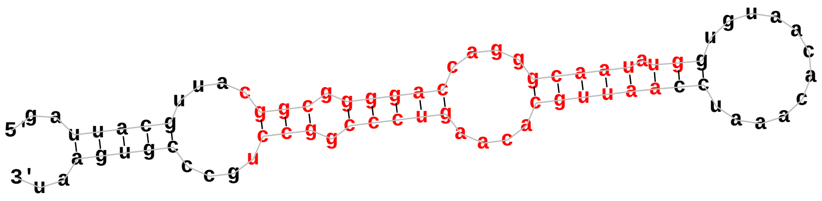

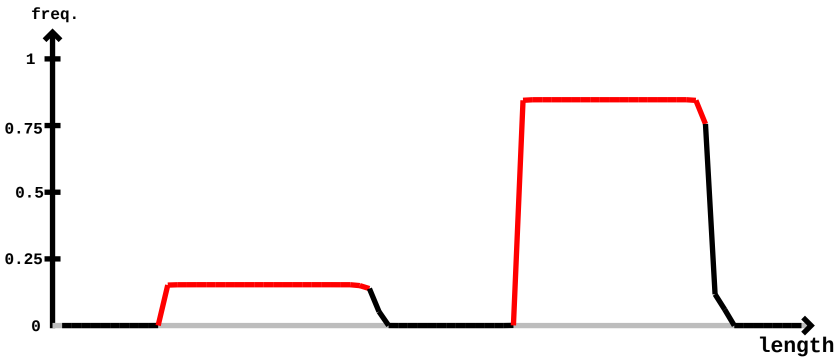


```
                     cgi-miR-92d-5p
                                                 cgi-miR-92d-3p
5'- gauuacguuacggcggggaccagggcaauaugguguaacacaaauccaauugcacaagucccggccugcccgugaau  -3'    exp
    ..(((((....(((.(((((....(((((.(((............))))))))....))))).)))....)))))..    reads      mm         sample
    ........uacggcggggaccagggcaaua...............................................    1          0          seq
    .........acggcggggaccagggcaauau..............................................    12         0          seq
    .........acggcggggaccagggcaauaug.............................................    23         0          seq
    ..........cggcggggaccagggcaa.................................................    10         0          seq
    ..........cggcggggaccagggcaau................................................    15         0          seq
    ..........cggcggggaccagggcaaua...............................................    558        0          seq
    ..........cggcggggaccagggcaauau..............................................    1914       0          seq
    ..........cggcggggaccagggcaauaug.............................................    14876      0          seq
    ..........cggcggggaccagggcaauaugg............................................    8969       0          seq
    ..........cggcggggaccagggcaauauggu...........................................    39         0          seq
    ..........cggcggggaccagggcaauauggug..........................................    1          0          seq
    ...........ggcggggaccagggcaaua...............................................    4          0          seq
    ...........ggcggggaccagggcaauau..............................................    11         0          seq
    ...........ggcggggaccagggcaauaug.............................................    119        0          seq
    ...........ggcggggaccagggcaauaugg............................................    83         0          seq
    ............gcggggaccagggcaaua...............................................    1          0          seq
    ............gcggggaccagggcaauaug.............................................    6          0          seq
    ............gcggggaccagggcaauaugg............................................    4          0          seq
    .............cggggaccagggcaauau..............................................    1          0          seq
    .............cggggaccagggcaauaug.............................................    7          0          seq
    .............cggggaccagggcaauaugg............................................    5          0          seq
    ..............ggggaccagggcaauaug.............................................    2          0          seq
    ..............ggggaccagggcaauaugg............................................    1          0          seq
    .............................................ccaauugcacaagucccggccu..........    1          0          seq
    ..............................................caauugcacaagucccgg.............    2          0          seq
    ..............................................caauugcacaagucccggcc...........    10         0          seq
    ..............................................caauugcacaagucccggccu..........    97         0          seq
    ..............................................caauugcacaagucccggccug.........    9          0          seq
    ..............................................caauugcacaagucccggccugc........    9          0          seq
    ...............................................aauugcacaagucccggc............    420        0          seq
    ...............................................aauugcacaagucccggcc...........    15330      0          seq
    ...............................................aauugcacaagucccggccu..........    110837     0          seq
    ...............................................aauugcacaagucccggccug.........    9854       0          seq
    ...............................................aauugcacaagucccggccugc........    10439      0          seq
```

```
          cgi-miR-92d-5p
                                               cgi-miR-92d-3p
gauuacguuacggcggggaccagggcaauaugguguaacacaaauccaauugcacaagucccggccugcccgugaau

...............................................aauugcacaagucccggccugcc.......    7    0    seq
................................................auugcacaagucccggcc...........    22   0    seq
................................................auugcacaagucccggccu..........    226  0    seq
................................................auugcacaagucccggccug.........    43   0    seq
................................................auugcacaagucccggccugc........    45   0    seq
.................................................uugcacaagucccggccu..........    9    0    seq
.................................................uugcacaagucccggccug.........    6    0    seq
.................................................uugcacaagucccggccugc........    2    0    seq
..................................................ugcacaagucccggccugc........    6    0    seq
...................................................gcacaagucccggccugc........    1    0    seq
```
