# Supplementary material for: Identification of Conserved and Novel MicroRNAs in the Pacific Oyster Crassostrea gigas by Deep Sequencing
Source: PLoS One. 2014 Aug 19;9(8):e104371. doi: 10.1371/journal.pone.0104371 (PMC4138081; doi:10.1371/journal.pone.0104371)
Supplement: File S2 — The compressed/ZIP file archive for the predicted precursors' secondary structures and reads alignment. (ZIP) [file pone.0104371.s010.zip › second structure and reads alignment for oyster miRNAs/conserved in table S4/cgi-miR-96a.pdf]

miRBase precursor : cgi-miR-96a
Total read count : 4034753
cgi-miR-96a-5p read count : 4034738
cgi-miR-96a-3p read count : 15
remaining reads : 0

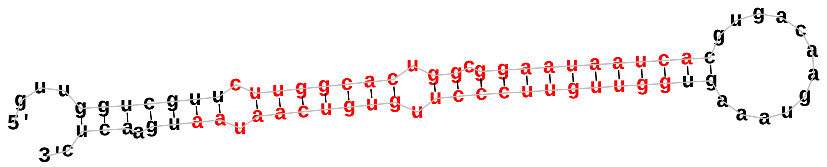

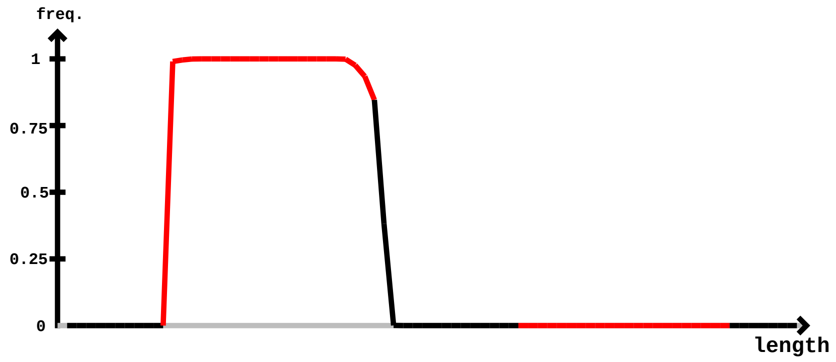

cgi-miR-96a-3p

cgi-miR-96a-5p

```
5'- guuggucguucuuggcacuggcggaauaaucacgugacaaguaaagugguuguucccuugugucaauaaugaacuc   -3'    exp
    ...((((((.(((((((.((.((((((((((((............))))))))))))).))))))).)))).))).    reads      mm         sample
    ........uucuuggcacuggcggaauaa...............................................    1          0          seq
    ........uucuuggcacuggcggaauaau..............................................    2          0          seq
    ........uucuuggcacuggcggaauaauc.............................................    1          0          seq
    ........uucuuggcacuggcggaauaauca............................................    2          0          seq
    .........ucuuggcacuggcggaaua................................................    1          0          seq
    .........ucuuggcacuggcggaauaa...............................................    86         0          seq
    .........ucuuggcacuggcggaauaau..............................................    1          0          seq
    .........ucuuggcacuggcggaauaauc.............................................    7          0          seq
    .........ucuuggcacuggcggaauaauca............................................    25         0          seq
    .........ucuuggcacuggcggaauaaucac...........................................    28         0          seq
    ..........cuuggcacuggcggaaua................................................    4431       0          seq
    ..........cuuggcacuggcggaauaa...............................................    91639      0          seq
    ..........cuuggcacuggcggaauaau..............................................    167210     0          seq
    ..........cuuggcacuggcggaauaauc.............................................    352607     0          seq
    ..........cuuggcacuggcggaauaauca............................................    1858777    0          seq
    ..........cuuggcacuggcggaauaaucac...........................................    1518539    0          seq
    ..........cuuggcacuggcggaauaaucacg..........................................    1690       0          seq
    ..........cuuggcacuggcggaauaaucacgu.........................................    117        0          seq
    ..........cuuggcacuggcggaauaaucacgug........................................    19         0          seq
    ..........cuuggcacuggcggaauaaucacguga.......................................    7          0          seq
    ...........uggcacuggcggaauaa................................................    322        0          seq
    ...........uggcacuggcggaauaau...............................................    652        0          seq
    ...........uggcacuggcggaauaauc..............................................    1747       0          seq
    ...........uggcacuggcggaauaauca.............................................    9060       0          seq
    ...........uggcacuggcggaauaaucac............................................    10706      0          seq
    ...........uggcacuggcggaauaaucacg...........................................    502        0          seq
    ...........uggcacuggcggaauaaucacgu..........................................    8          0          seq
    ...........uggcacuggcggaauaaucacgug.........................................    1          0          seq
    ............uggcacuggcggaauaau..............................................    499        0          seq
    ............uggcacuggcggaauaauc.............................................    1289       0          seq
    ............uggcacuggcggaauaauca............................................    6165       0          seq
    ............uggcacuggcggaauaaucac...........................................    6415       0          seq
    ............uggcacuggcggaauaaucacg..........................................    13         0          seq
    ............uggcacuggcggaauaaucacgu.........................................    9          0          seq
```

```
                                               cgi-miR-96a-3p
          cgi-miR-96a-5p
guuggucguucuuggcacuggcggaauaaucacgugacaaguaaagugguuguucccuugugucaauaaugaacuc

............uggcacuggcggaauaaucacgug........................................    1      0      seq
.............ggcacuggcggaauaauc.............................................    160    0      seq
.............ggcacuggcggaauaauca............................................    784    0      seq
.............ggcacuggcggaauaaucac...........................................    1135   0      seq
.............ggcacuggcggaauaaucacg..........................................    2      0      seq
..............gcacuggcggaauaauca............................................    24     0      seq
..............gcacuggcggaauaaucac...........................................    37     0      seq
...............cacuggcggaauaaucac...........................................    17     0      seq
..............................................ugguuguucccuugugucaaua........    1      0      seq
...............................................gguuguucccuugugucaa..........    1      0      seq
...............................................gguuguucccuugugucaaua........    2      0      seq
...............................................gguuguucccuugugucaauaa.......    8      0      seq
................................................guuguucccuugugucaauaa.......    3      0      seq
```
