# Supplementary material for: Identification of Conserved and Novel MicroRNAs in the Pacific Oyster Crassostrea gigas by Deep Sequencing
Source: PLoS One. 2014 Aug 19;9(8):e104371. doi: 10.1371/journal.pone.0104371 (PMC4138081; doi:10.1371/journal.pone.0104371)
Supplement: File S2 — The compressed/ZIP file archive for the predicted precursors' secondary structures and reads alignment. (ZIP) [file pone.0104371.s010.zip › second structure and reads alignment for oyster miRNAs/conserved in table S4/cgi-miR-96b.pdf]

miRBase precursor : cgi-miR-96b
Total read count : 63318
cgi-miR-96b-5p read count : 62959
cgi-miR-96b-3p read count : 359
remaining reads : 0

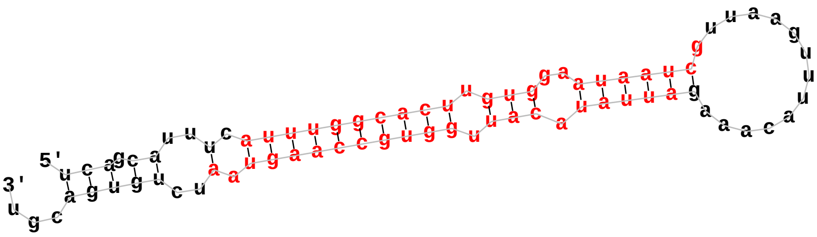

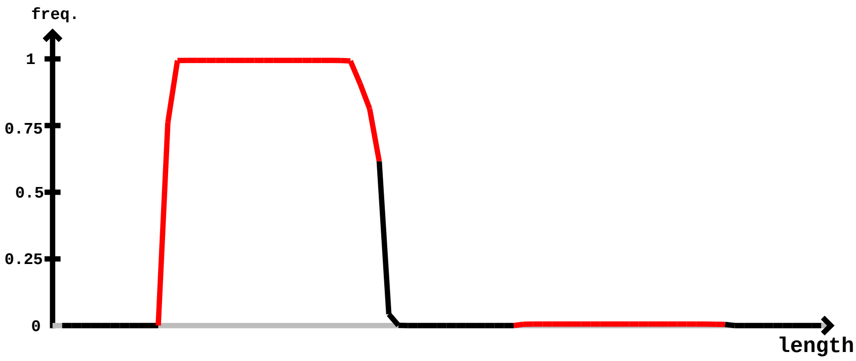


```
                             cgi-miR-96b-5p
                                                              cgi-miR-96b-3p
5'- ucagcauuucauuuggcacuuguggaauaaucguuaaguuuacaaagauuauacauuggugccaaguaaucugugacgu   -3'    exp
    .(((.(((..((((((((((.(((..((((((..............)))))).))).))))))))))))))))......    reads    mm    sample
    ........ucauuuggcacuuguggaauaauc...............................................    1        0     seq
    .........cauuuggcacuuguggaauaa.................................................    3        0     seq
    .........cauuuggcacuuguggaauaau................................................    1        0     seq
    .........cauuuggcacuuguggaauaaucg..............................................    3        0     seq
    .........cauuuggcacuuguggaauaaucgu.............................................    1        0     seq
    ..........auuuggcacuuguggaau...................................................    28       0     seq
    ..........auuuggcacuuguggaaua..................................................    91       0     seq
    ..........auuuggcacuuguggaauaa.................................................    4336     0     seq
    ..........auuuggcacuuguggaauaau................................................    5666     0     seq
    ..........auuuggcacuuguggaauaauc...............................................    11556    0     seq
    ..........auuuggcacuuguggaauaaucg..............................................    26062    0     seq
    ..........auuuggcacuuguggaauaaucgu.............................................    438      0     seq
    ..........auuuggcacuuguggaauaaucguu............................................    7        0     seq
    ..........auuuggcacuuguggaauaaucguuaag.........................................    1        0     seq
    ...........uuuggcacuuguggaaua..................................................    7        0     seq
    ...........uuuggcacuuguggaauaa.................................................    1016     0     seq
    ...........uuuggcacuuguggaauaau................................................    295      0     seq
    ...........uuuggcacuuguggaauaauc...............................................    960      0     seq
    ...........uuuggcacuuguggaauaaucg..............................................    10217    0     seq
    ...........uuuggcacuuguggaauaaucgu.............................................    2201     0     seq
    ...........uuuggcacuuguggaauaaucguu............................................    40       0     seq
    ...........uuuggcacuuguggaauaaucguuaag.........................................    2        0     seq
    ............uuggcacuuguggaauaau................................................    1        0     seq
    ............uuggcacuuguggaauaaucg..............................................    15       0     seq
    ............uuggcacuuguggaauaaucgu.............................................    1        0     seq
    .............uggcacuuguggaauaau................................................    2        0     seq
    .............uggcacuuguggaauaauc...............................................    2        0     seq
    .............uggcacuuguggaauaaucg..............................................    5        0     seq
    .............uggcacuuguggaauaaucgu.............................................    1        0     seq
    ..............................................auuauacauuggugccaa...............    3        0     seq
    ..............................................auuauacauuggugccaag..............    6        0     seq
    ..............................................auuauacauuggugccaagu.............    40       0     seq
    ..............................................auuauacauuggugccaagua............    33       0     seq
    ..............................................auuauacauuggugccaaguaa...........    230      0     seq
```

```
         cgi-miR-96b-5p
                                                cgi-miR-96b-3p
ucagcauuucauuuggcacuuguggaauaaucguuaaguuuacaaagauuauacauuggugccaaguaaucugugacgu

...............................................auuauacauuggugccaaguaau.........    2          0          seq
................................................uuauacauuggugccaag.............    1          0          seq
................................................uuauacauuggugccaagu............    1          0          seq
................................................uuauacauuggugccaagua...........    1          0          seq
................................................uuauacauuggugccaaguaa..........    40         0          seq
.................................................uauacauuggugccaagua...........    1          0          seq
.................................................uauacauuggugccaaguaa..........    1          0          seq
```
