# Supplementary material for: Identification of Conserved and Novel MicroRNAs in the Pacific Oyster Crassostrea gigas by Deep Sequencing
Source: PLoS One. 2014 Aug 19;9(8):e104371. doi: 10.1371/journal.pone.0104371 (PMC4138081; doi:10.1371/journal.pone.0104371)
Supplement: File S2 — The compressed/ZIP file archive for the predicted precursors' secondary structures and reads alignment. (ZIP) [file pone.0104371.s010.zip › second structure and reads alignment for oyster miRNAs/conserved in table S4/cgi-miR-981.pdf]

miRBase precursor : cgi-miR-981
Total read count : 1199903
cgi-miR-981-5p read count : 2
cgi-miR-981-3p read count : 1192863
remaining reads : 7038

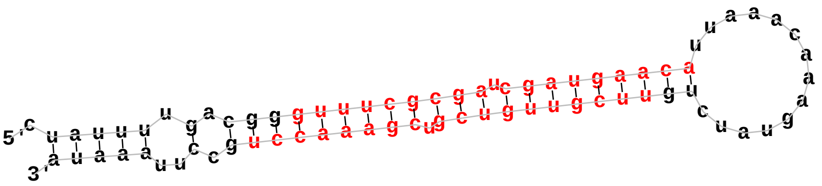

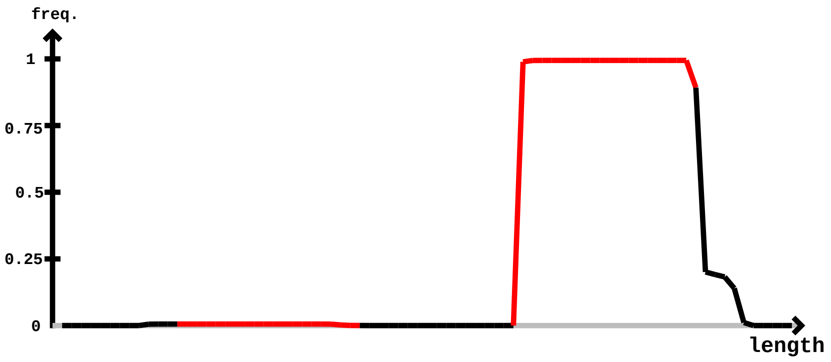


```
                    cgi-miR-981-5p
                                              cgi-miR-981-3p
5'- cuauuuugacggguuucgcgaucgaugaacauuaaacaaaguaucuguucguugucgucgaaaccugccuuaaaua   -3'    exp
    .........((((((((((((.(((((((((..............))))))))))))).)))))))))).........   reads      mm         sample
    .......gacggguuucgcgaucga...................................................   1          0          seq
    .......gacggguuucgcgaucgaugaa...............................................   1          0          seq
    ........acggguuucgcgaucgau..................................................   52         0          seq
    ........acggguuucgcgaucgaug.................................................   290        0          seq
    ........acggguuucgcgaucgauga................................................   3168       0          seq
    ........acggguuucgcgaucgaugaa...............................................   2113       0          seq
    ........acggguuucgcgaucgaugaac..............................................   305        0          seq
    ........acggguuucgcgaucgaugaaca.............................................   252        0          seq
    ........acggguuucgcgaucgaugaacau............................................   3          0          seq
    .........cggguuucgcgaucgaug.................................................   58         0          seq
    .........cggguuucgcgaucgauga................................................   358        0          seq
    .........cggguuucgcgaucgaugaa...............................................   209        0          seq
    .........cggguuucgcgaucgaugaac..............................................   15         0          seq
    .........cggguuucgcgaucgaugaaca.............................................   29         0          seq
    .........cggguuucgcgaucgaugaacau............................................   1          0          seq
    ..........ggguuucgcgaucgaugaaca.............................................   1          0          seq
    ...........gguuucgcgaucgaugaa...............................................   1          0          seq
    ...............................uuaaacaaaguaucuguu...........................   1          0          seq
    ...............................uuaaacaaaguaucuguuc..........................   1          0          seq
    ...............................uuaaacaaaguaucuguucguu.......................   1          0          seq
    ...............................uuaaacaaaguaucuguucguug......................   5          0          seq
    ...............................uuaaacaaaguaucuguucguuguc....................   2          0          seq
    ...............................uuaaacaaaguaucuguucguugucg...................   2          0          seq
    ............................................cuguucguugucgucgaaac............   1          0          seq
    ............................................cuguucguugucgucgaaaccu..........   1          0          seq
    .............................................uguucguugucgucgaaaccu..........   1          0          seq
    ..............................................guucguugucgucgaaacc...........   5          0          seq
    ..............................................guucguugucgucgaaaccu..........   21         0          seq
    ..............................................guucguugucgucgaaaccug.........   1          0          seq
    ...............................................uucguugucgucgaaacc...........   122363     0          seq
    ...............................................uucguugucgucgaaaccu..........   824548     0          seq
    ...............................................uucguugucgucgaaaccug.........   11548      0          seq
    ...............................................uucguugucgucgaaaccugc........   10632      0          seq
    ...............................................uucguugucgucgaaaccugcc.......   50870      0          seq
```

```
            cgi-miR-981-5p
                                               cgi-miR-981-3p
cuauuuugacggguuucgcgaucgaugaacauuaaacaaaguaucuguucguugucgucgaaaccugccuuaaaua

...............................................uucguugucgucgaaaccugccu......    153131     0          seq
...............................................uucguugucgucgaaaccugccuu.....    13282      0          seq
...............................................uucguugucgucgaaaccugccuua....    140        0          seq
...............................................uucguugucgucgaaaccugccuuaa...    29         0          seq
................................................ucguugucgucgaaaccu..........    4889       0          seq
................................................ucguugucgucgaaaccug.........    53         0          seq
................................................ucguugucgucgaaaccugc........    51         0          seq
................................................ucguugucgucgaaaccugcc.......    223        0          seq
................................................ucguugucgucgaaaccugccu......    855        0          seq
................................................ucguugucgucgaaaccugccuu.....    76         0          seq
.................................................cguugucgucgaaaccug.........    9          0          seq
.................................................cguugucgucgaaaccugc........    7          0          seq
.................................................cguugucgucgaaaccugcc.......    41         0          seq
.................................................cguugucgucgaaaccugccu......    168        0          seq
.................................................cguugucgucgaaaccugccuu.....    21         0          seq
..................................................guugucgucgaaaccugc........    1          0          seq
..................................................guugucgucgaaaccugcc.......    10         0          seq
..................................................guugucgucgaaaccugccu......    43         0          seq
..................................................guugucgucgaaaccugccuu.....    7          0          seq
...................................................uugucgucgaaaccugcc.......    1          0          seq
...................................................uugucgucgaaaccugccu......    6          0          seq
```
