# Supplementary material for: Identification of Conserved and Novel MicroRNAs in the Pacific Oyster Crassostrea gigas by Deep Sequencing
Source: PLoS One. 2014 Aug 19;9(8):e104371. doi: 10.1371/journal.pone.0104371 (PMC4138081; doi:10.1371/journal.pone.0104371)
Supplement: File S2 — The compressed/ZIP file archive for the predicted precursors' secondary structures and reads alignment. (ZIP) [file pone.0104371.s010.zip › second structure and reads alignment for oyster miRNAs/conserved in table S4/cgi-miR-9a.pdf]

miRBase precursor	: cgi-miR-9a
Total read count	: 145
cgi-miR-9a-5p read count	: 116
cgi-miR-9a-3p read count	: 29
remaining reads	: 0

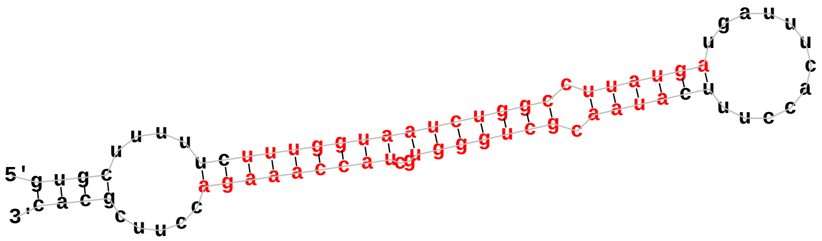

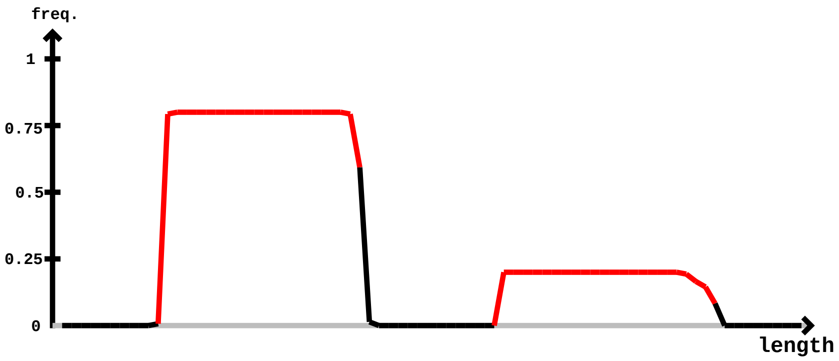


```
                                              cgi-miR-9a-3p
          cgi-miR-9a-5p
5'- gugcuuuuucuuugguaaucuggccuuaugaugauuucaccuuucauaacgcugggugcuaccaaagaccuucgcac   -3'   exp
    ((((....((((((((((((((((.((((((............)))))).)))))))..))))))))).....))))    reads    mm     sample
    .........cuuugguaaucuggccuuaugau.............................................    1        0      seq
    ..........uuugguaaucuggccuuau................................................    1        0      seq
    ..........uuugguaaucuggccuuaug...............................................    28       0      seq
    ..........uuugguaaucuggccuuauga..............................................    84       0      seq
    ..........uuugguaaucuggccuuaugau.............................................    1        0      seq
    ...........uugguaaucuggccuuaug...............................................    1        0      seq
    .............................................auaacgcugggugcuacca.............    1        0      seq
    .............................................auaacgcugggugcuaccaa............    4        0      seq
    .............................................auaacgcugggugcuaccaaa...........    3        0      seq
    .............................................auaacgcugggugcuaccaaag..........    9        0      seq
    .............................................auaacgcugggugcuaccaaaga.........    12       0      seq
```
