# Supplementary material for: Identification of Conserved and Novel MicroRNAs in the Pacific Oyster Crassostrea gigas by Deep Sequencing
Source: PLoS One. 2014 Aug 19;9(8):e104371. doi: 10.1371/journal.pone.0104371 (PMC4138081; doi:10.1371/journal.pone.0104371)
Supplement: File S2 — The compressed/ZIP file archive for the predicted precursors' secondary structures and reads alignment. (ZIP) [file pone.0104371.s010.zip › second structure and reads alignment for oyster miRNAs/conserved in table S4/cgi-miR-9b.pdf]

miRBase precursor : cgi-miR-9b
Total read count : 128726
cgi-miR-9b-5p read count : 116785
cgi-miR-9b-3p read count : 11939
remaining reads : 2

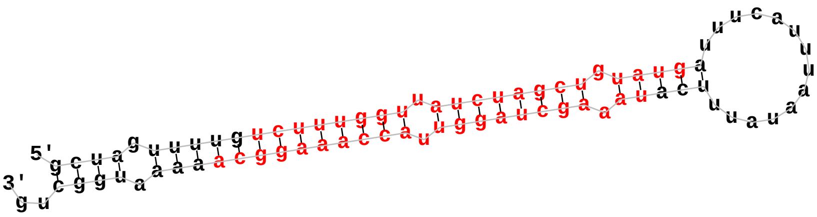

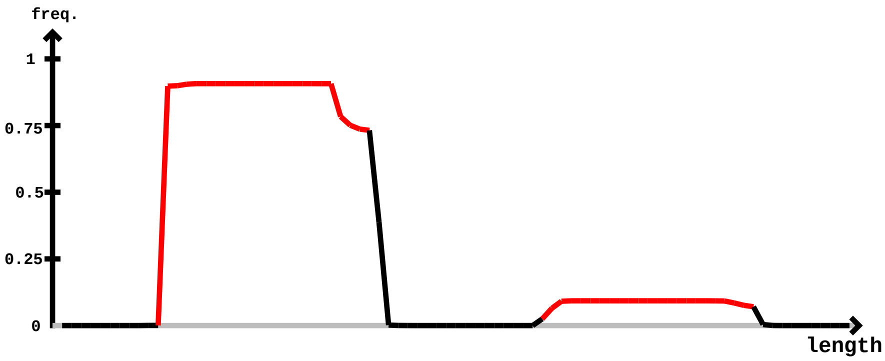

cgi-miR-9b-3p

cgi-miR-9b-5p

5'- gcuaguuuugucuuugguuaucuagcuguaugauuucauuuaauauuucauaaagcuagguuaccaaaggcaaaaauggcug -3'	exp		
((((.(((((((((((((.((((((((.(((((..............))))).)))))))).)))))))))))))).))))..	reads	mm	sample
........ugucuuugguuaucuagc..	27	0	seq
........ugucuuugguuaucuagcu...	17	0	seq
........ugucuuugguuaucuagcug..	9	0	seq
........ugucuuugguuaucuagcugu...	3	0	seq
........ugucuuugguuaucuagcugua..	4	0	seq
.........gucuuugguuaucuagcu...	3	0	seq
.........gucuuugguuaucuagcug..	3	0	seq
..........ucuuugguuaucuagcug..	15853	0	seq
..........ucuuugguuaucuagcugu...	4173	0	seq
..........ucuuugguuaucuagcugua..	1846	0	seq
..........ucuuugguuaucuagcuguau...	583	0	seq
..........ucuuugguuaucuagcuguaug..	43828	0	seq
..........ucuuugguuaucuagcuguauga...	49020	0	seq
..........ucuuugguuaucuagcuguaugau..	168	0	seq
..........ucuuugguuaucuagcuguaugauu...	29	0	seq
..........ucuuugguuaucuagcuguaugauuu..	14	0	seq
...........cuuugguuaucuagcugu...	12	0	seq
...........cuuugguuaucuagcuguau...	1	0	seq
...........cuuugguuaucuagcuguaug..	61	0	seq
...........cuuugguuaucuagcuguauga...	106	0	seq
...........cuuugguuaucuagcuguaugau..	11	0	seq
...........cuuugguuaucuagcuguaugauu...	1	0	seq
...........cuuugguuaucuagcuguaugauuu..	1	0	seq
............uuugguuaucuagcugua..	11	0	seq
............uuugguuaucuagcuguau...	5	0	seq
............uuugguuaucuagcuguaug..	271	0	seq
............uuugguuaucuagcuguauga...	448	0	seq
............uuugguuaucuagcuguaugau..	4	0	seq
.............uugguuaucuagcuguau...	1	0	seq
.............uugguuaucuagcuguaug..	95	0	seq
.............uugguuaucuagcuguauga...	164	0	seq
.............uugguuaucuagcuguaugau..	2	0	seq
.............uugguuaucuagcuguaugauu...	1	0	seq
..............ugguuaucuagcuguaug..	3	0	seq

```
                                                  cgi-miR-9b-3p
          cgi-miR-9b-5p
gcuaguuuugucuuugguuaucuagcuguaugauuucauuuaauauuucauaaagcuagguuaccaaaggcaaaauggcug

..............ugguuaucuagcuguauga.................................................   6       0       seq
..............ugguuaucuagcuguaugauuu..............................................   1       0       seq
..............................................uucauaaagcuagguuaccaaag.............   1       0       seq
...............................................ucauaaagcuagguuaccaaa..............   1       0       seq
................................................cauaaagcuagguuaccaaagg............   2       0       seq
................................................cauaaagcuagguuaccaaaggc...........   1       0       seq
................................................cauaaagcuagguuaccaaaggcaa.........   1       0       seq
.................................................auaaagcuagguuaccaa...............   17      0       seq
.................................................auaaagcuagguuaccaaa..............   33      0       seq
.................................................auaaagcuagguuaccaaag.............   362     0       seq
.................................................auaaagcuagguuaccaaagg............   327     0       seq
.................................................auaaagcuagguuaccaaaggc...........   466     0       seq
.................................................auaaagcuagguuaccaaaggca..........   1911    0       seq
.................................................auaaagcuagguuaccaaaggcaa.........   97      0       seq
.................................................auaaagcuagguuaccaaaggcaaa........   3       0       seq
.................................................auaaagcuagguuaccaaaggcaaaa.......   1       0       seq
..................................................uaaagcuagguuaccaaa..............   37      0       seq
..................................................uaaagcuagguuaccaaag.............   450     0       seq
..................................................uaaagcuagguuaccaaagg............   499     0       seq
..................................................uaaagcuagguuaccaaaggc...........   132     0       seq
..................................................uaaagcuagguuaccaaaggca..........   3680    0       seq
..................................................uaaagcuagguuaccaaaggcaa.........   277     0       seq
..................................................uaaagcuagguuaccaaaggcaaa........   17      0       seq
..................................................uaaagcuagguuaccaaaggcaaaa.......   1       0       seq
..................................................uaaagcuagguuaccaaaggcaaaaa......   1       0       seq
...................................................aaagcuagguuaccaaag.............   153     0       seq
...................................................aaagcuagguuaccaaagg............   265     0       seq
...................................................aaagcuagguuaccaaaggc...........   69      0       seq
...................................................aaagcuagguuaccaaaggca..........   2854    0       seq
...................................................aaagcuagguuaccaaaggcaa.........   80      0       seq
...................................................aaagcuagguuaccaaaggcaaa........   6       0       seq
....................................................aagcuagguuaccaaagg............   30      0       seq
....................................................aagcuagguuaccaaaggc...........   7       0       seq
....................................................aagcuagguuaccaaaggca..........   129     0       seq
....................................................aagcuagguuaccaaaggcaa.........   11      0       seq
....................................................aagcuagguuaccaaaggcaaa........   13      0       seq
.....................................................agcuagguuaccaaaggca..........   7       0       seq
```
