# Supplementary material for: Identification of Conserved and Novel MicroRNAs in the Pacific Oyster Crassostrea gigas by Deep Sequencing
Source: PLoS One. 2014 Aug 19;9(8):e104371. doi: 10.1371/journal.pone.0104371 (PMC4138081; doi:10.1371/journal.pone.0104371)
Supplement: File S2 — The compressed/ZIP file archive for the predicted precursors' secondary structures and reads alignment. (ZIP) [file pone.0104371.s010.zip › second structure and reads alignment for oyster miRNAs/conserved in table S4/cgi-miR-9c.pdf]

miRBase precursor : cgi-miR-9c
Total read count : 58362
cgi-miR-9c-5p read count : 40869
cgi-miR-9c-3p read count : 17481
remaining reads : 12

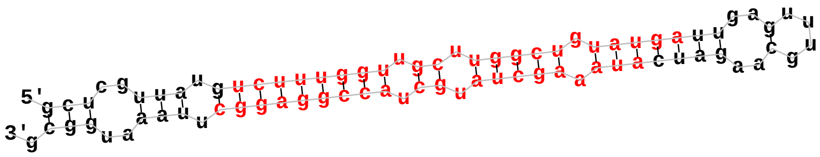

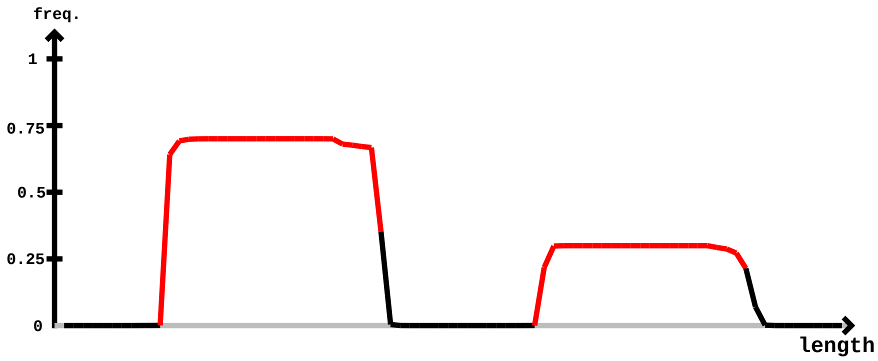


```
                                          cgi-miR-9c-3p
             cgi-miR-9c-5p
5'- gcucguuaugucuuugguugcuuggcuguaugauugaguuugcaagaucauaaagcuaugcuaccggaggcuuaaauggcg  -3'    exp
    (((..(((.(((((((((.((.(((((.(((((((..(....)..))))))).))))).)).))))))))).)))..))).      reads      mm         sample
    ......uaugucuuugguugcuuggcu......................................................      1          0          seq
    .......augucuuugguugcuuggc.......................................................      1          0          seq
    .......augucuuugguugcuuggcu......................................................      5          0          seq
    .......augucuuugguugcuuggcug.....................................................      2          0          seq
    ........ugucuuugguugcuuggcu......................................................      5          0          seq
    ........ugucuuugguugcuuggcug.....................................................      1          0          seq
    ........ugucuuugguugcuuggcugu....................................................      1          0          seq
    .........gucuuugguugcuuggcu......................................................      12         0          seq
    .........gucuuugguugcuuggcug.....................................................      2          0          seq
    .........gucuuugguugcuuggcugu....................................................      1          0          seq
    ..........ucuuugguugcuuggcug.....................................................      1148       0          seq
    ..........ucuuugguugcuuggcugu....................................................      205        0          seq
    ..........ucuuugguugcuuggcugua...................................................      283        0          seq
    ..........ucuuugguugcuuggcuguau..................................................      214        0          seq
    ..........ucuuugguugcuuggcuguaug.................................................      17068      0          seq
    ..........ucuuugguugcuuggcuguauga................................................      18390      0          seq
    ..........ucuuugguugcuuggcuguaugau...............................................      90         0          seq
    ..........ucuuugguugcuuggcuguaugauu..............................................      3          0          seq
    ...........cuuugguugcuuggcugu....................................................      12         0          seq
    ...........cuuugguugcuuggcugua...................................................      19         0          seq
    ...........cuuugguugcuuggcuguau..................................................      10         0          seq
    ...........cuuugguugcuuggcuguaug.................................................      1220       0          seq
    ...........cuuugguugcuuggcuguauga................................................      1695       0          seq
    ...........cuuugguugcuuggcuguaugau...............................................      14         0          seq
    ...........cuuugguugcuuggcuguaugauu..............................................      1          0          seq
    ............uuugguugcuuggcuguaug.................................................      98         0          seq
    ............uuugguugcuuggcuguauga................................................      153        0          seq
    ............uuugguugcuuggcuguaugau...............................................      121        0          seq
    ............uuugguugcuuggcuguaugauu..............................................      2          0          seq
    .............uugguugcuuggcuguaug.................................................      33         0          seq
    .............uugguugcuuggcuguauga................................................      50         0          seq
    .............uugguugcuuggcuguaugau...............................................      2          0          seq
    ..............ugguugcuuggcuguaug.................................................      8          0          seq
    ..............ugguugcuuggcuguauga................................................      5          0          seq
```

```
                                                      cgi-miR-9c-3p
          cgi-miR-9c-5p
gcucguuaugucuuugguugcuuggcuguaugauugaguuugcaagaucauaaaagcuaugcuaccggaggcuuaaauggcg

..............ugguugcuuggcuguaugau................................................    1      0      seq
..............ugguugcuuggcuguaugauu...............................................    1      0      seq
...............gguugcuuggcuguauga.................................................    1      0      seq
.................uugcuuggcuguaugauugaguuugca......................................    1      0      seq
....................cuuggcuguaugauugaguuugcaagau..................................    1      0      seq
.............................................gaucauaaaagcuaugcuaccggaggc..........    1      0      seq
...............................................ucauaaaagcuaugcuaccggaggc..........    4      0      seq
................................................cauaaaagcuaugcuaccg...............    3      0      seq
................................................cauaaaagcuaugcuaccggagg...........    2      0      seq
................................................cauaaaagcuaugcuaccggaggc..........    5      0      seq
................................................cauaaaagcuaugcuaccggaggcu.........    3      0      seq
.................................................auaaaagcuaugcuaccgg..............    403    0      seq
.................................................auaaaagcuaugcuaccgga.............    265    0      seq
.................................................auaaaagcuaugcuaccggag............    743    0      seq
.................................................auaaaagcuaugcuaccggagg...........    3043   0      seq
.................................................auaaaagcuaugcuaccggaggc..........    7371   0      seq
.................................................auaaaagcuaugcuaccggaggcu.........    863    0      seq
.................................................auaaaagcuaugcuaccggaggcuu........    17     0      seq
.................................................auaaaagcuaugcuaccggaggcuua.......    3      0      seq
..................................................uaaaagcuaugcuaccgga.............    54     0      seq
..................................................uaaaagcuaugcuaccggag............    142    0      seq
..................................................uaaaagcuaugcuaccggagg...........    304    0      seq
..................................................uaaaagcuaugcuaccggaggc..........    1044   0      seq
..................................................uaaaagcuaugcuaccggaggcu.........    3072   0      seq
..................................................uaaaagcuaugcuaccggaggcuu........    64     0      seq
....................................................aaagcuaugcuaccggag............    1      0      seq
....................................................aaagcuaugcuaccggagg...........    8      0      seq
....................................................aaagcuaugcuaccggaggc..........    15     0      seq
....................................................aaagcuaugcuaccggaggcu.........    36     0      seq
....................................................aaagcuaugcuaccggaggcuu........    7      0      seq
.....................................................aagcuaugcuaccggaggc..........    2      0      seq
.....................................................aagcuaugcuaccggaggcu.........    6      0      seq
......................................................agcuaugcuaccggaggc..........    1      0      seq
```
