# Supplementary material for: Identification of Conserved and Novel MicroRNAs in the Pacific Oyster Crassostrea gigas by Deep Sequencing
Source: PLoS One. 2014 Aug 19;9(8):e104371. doi: 10.1371/journal.pone.0104371 (PMC4138081; doi:10.1371/journal.pone.0104371)
Supplement: File S2 — The compressed/ZIP file archive for the predicted precursors' secondary structures and reads alignment. (ZIP) [file pone.0104371.s010.zip › second structure and reads alignment for oyster miRNAs/conserved in table S4/cgi-miR-9d.pdf]

miRBase precursor : cgi-miR-9d
Total read count : 58446
cgi-miR-9d-5p read count : 40870
cgi-miR-9d-3p read count : 17570
remaining reads : 6

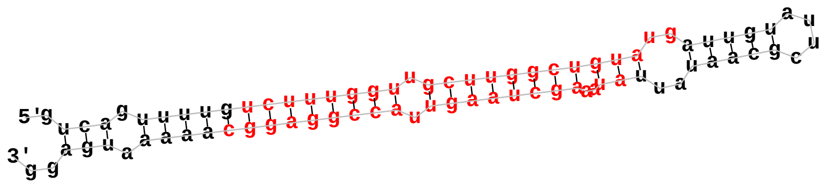

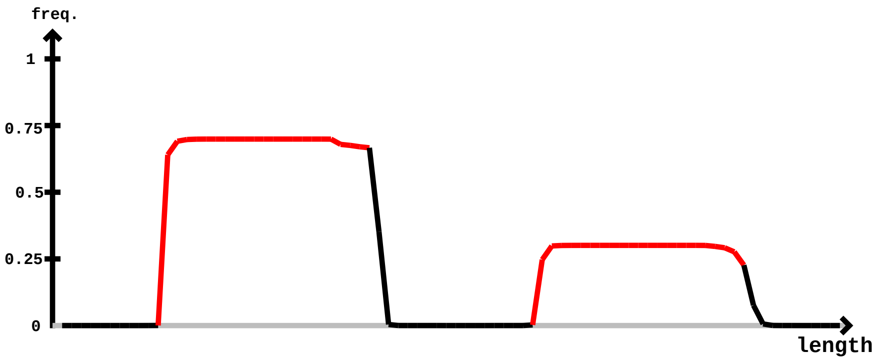

cgi-miR-9d-5p

cgi-miR-9d-3p

```
5'- gucaguuuugucuuugguugcuuggcuguaugauuguauucgcaauauuauaaagcuaaguuaccggaggcaaaaaugagg  -3'    exp
    .(((.(((((((((((((.((((((((((..(((((....)))))..)))..)))))))).))))))))))))).)))..   reads   mm   sample
    ........ugucuuugguugcuuggcu......................................................   5       0    seq
    ........ugucuuugguugcuuggcug.....................................................   1       0    seq
    ........ugucuuugguugcuuggcugu....................................................   1       0    seq
    .........gucuuugguugcuuggcu......................................................   12      0    seq
    .........gucuuugguugcuuggcug.....................................................   2       0    seq
    .........gucuuugguugcuuggcugu....................................................   1       0    seq
    ..........ucuuugguugcuuggcug.....................................................   1148    0    seq
    ..........ucuuugguugcuuggcugu....................................................   205     0    seq
    ..........ucuuugguugcuuggcugua...................................................   283     0    seq
    ..........ucuuugguugcuuggcuguau..................................................   214     0    seq
    ..........ucuuugguugcuuggcuguaug.................................................   17068   0    seq
    ..........ucuuugguugcuuggcuguauga................................................   18390   0    seq
    ..........ucuuugguugcuuggcuguaugau...............................................   90      0    seq
    ..........ucuuugguugcuuggcuguaugauu..............................................   3       0    seq
    ..........ucuuugguugcuuggcuguaugauugu............................................   1       0    seq
    ...........cuugguugcuuggcugu.....................................................   12      0    seq
    ...........cuugguugcuuggcugua....................................................   19      0    seq
    ...........cuugguugcuuggcuguau...................................................   10      0    seq
    ...........cuugguugcuuggcuguaug..................................................   1220    0    seq
    ...........cuugguugcuuggcuguauga.................................................   1695    0    seq
    ...........cuugguugcuuggcuguaugau................................................   14      0    seq
    ...........cuugguugcuuggcuguaugauu...............................................   1       0    seq
    ...........cuugguugcuuggcuguaugauugua............................................   1       0    seq
    ...........cuugguugcuuggcuguaugauuguau...........................................   1       0    seq
    ............uugguugcuuggcuguaug..................................................   98      0    seq
    ............uugguugcuuggcuguauga.................................................   153     0    seq
    ............uugguugcuuggcuguaugau................................................   121     0    seq
    ............uugguugcuuggcuguaugauu...............................................   2       0    seq
    .............ugguugcuuggcuguaug..................................................   33      0    seq
    .............ugguugcuuggcuguauga.................................................   50      0    seq
    .............ugguugcuuggcuguaugau................................................   2       0    seq
    ..............gguugcuuggcuguaug..................................................   8       0    seq
    ..............gguugcuuggcuguauga.................................................   5       0    seq
    ..............gguugcuuggcuguaugau................................................   1       0    seq
```

```
          cgi-miR-9d-5p
                                                cgi-miR-9d-3p
gucaguuuugucuuugguugcuuggcuguaugauuguauucgcaauauuauaaagcuaaguuaccggaggcaaaaaugagg

..............ugguugcuuggcuguaugauu..............................................    1      0      seq
...............gguugcuuggcuguauga................................................    1      0      seq
.....................uuggcuguaugauuguauucgcaauauu................................    1      0      seq
......................uggcuguaugauuguauucgcaau...................................    1      0      seq
............................................auauuauaaagcuaaguuaccggaggc..........    2      0      seq
................................................uauaaagcuaaguuaccg...............    12     0      seq
................................................uauaaagcuaaguuaccgg..............    4      0      seq
................................................uauaaagcuaaguuaccgga.............    2      0      seq
................................................uauaaagcuaaguuaccggag............    8      0      seq
................................................uauaaagcuaaguuaccggagg...........    32     0      seq
................................................uauaaagcuaaguuaccggaggc..........    71     0      seq
................................................uauaaagcuaaguuaccggaggca.........    12     0      seq
................................................uauaaagcuaaguuaccggaggcaa........    2      0      seq
.................................................auaaagcuaaguuaccgg..............    212    0      seq
.................................................auaaagcuaaguuaccgga.............    274    0      seq
.................................................auaaagcuaaguuaccggag............    830    0      seq
.................................................auaaagcuaaguuaccggagg...........    2783   0      seq
.................................................auaaagcuaaguuaccggaggc..........    8610   0      seq
.................................................auaaagcuaaguuaccggaggca.........    1432   0      seq
.................................................auaaagcuaaguuaccggaggcaa........    143    0      seq
.................................................auaaagcuaaguuaccggaggcaaa.......    13     0      seq
.................................................auaaagcuaaguuaccggaggcaaaaa.....    3      0      seq
..................................................uaaagcuaaguuaccgga.............    21     0      seq
..................................................uaaagcuaaguuaccggag............    53     0      seq
..................................................uaaagcuaaguuaccggagg...........    92     0      seq
..................................................uaaagcuaaguuaccggaggc..........    96     0      seq
..................................................uaaagcuaaguuaccggaggca.........    2582   0      seq
..................................................uaaagcuaaguuaccggaggcaa........    150    0      seq
..................................................uaaagcuaaguuaccggaggcaaa.......    5      0      seq
..................................................uaaagcuaaguuaccggaggcaaaa......    2      0      seq
...................................................aaagcuaaguuaccggag............    1      0      seq
...................................................aaagcuaaguuaccggagg...........    6      0      seq
...................................................aaagcuaaguuaccggaggc..........    9      0      seq
...................................................aaagcuaaguuaccggaggca.........    52     0      seq
...................................................aaagcuaaguuaccggaggcaa........    34     0      seq
...................................................aaagcuaaguuaccggaggcaaa.......    1      0      seq
....................................................aagcuaaguuaccggagg...........    1      0      seq
....................................................aagcuaaguuaccggaggc..........    9      0      seq
....................................................aagcuaaguuaccggaggca.........    7      0      seq
....................................................aagcuaaguuaccggaggcaa........    2      0      seq
.....................................................agcuaaguuaccggaggc..........    2      0      seq
.....................................................agcuaaguuaccggaggca.........    2      0      seq
```
