# Supplementary material for: Identification of Conserved and Novel MicroRNAs in the Pacific Oyster Crassostrea gigas by Deep Sequencing
Source: PLoS One. 2014 Aug 19;9(8):e104371. doi: 10.1371/journal.pone.0104371 (PMC4138081; doi:10.1371/journal.pone.0104371)
Supplement: File S2 — The compressed/ZIP file archive for the predicted precursors' secondary structures and reads alignment. (ZIP) [file pone.0104371.s010.zip › second structure and reads alignment for oyster miRNAs/novel in table S5/m0092.pdf]

miRBase precursor : m0092
Total read count : 43915
m0092_5p read count : 43883
m0092_3p read count : 32
remaining reads : 0

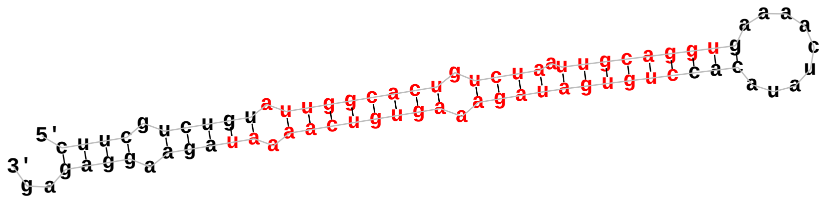


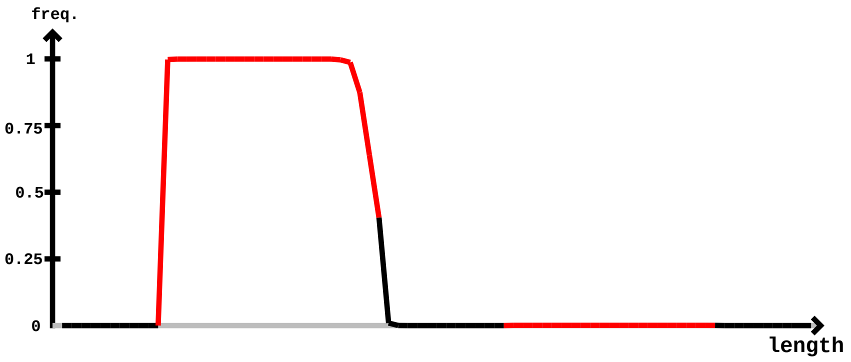


```
                                              m0092_3p
           m0092_5p
5'- cuucgucuguauuggcacugucuaauugcaggugaaaacuauacaccugugauagaaagugucaaaauagaaggagag   -3'    exp
    ((((.(((((.((((((((.((((.(((((((((........))))))))))))).)))))))).))))).))))..   reads      mm         sample
    ........guauuggcacugucuaauugcag...............................................   2          0          seq
    ..........auuggcacugucuaauug..................................................   131        0          seq
    ..........auuggcacugucuaauugc.................................................   417        0          seq
    ..........auuggcacugucuaauugca................................................   4922       0          seq
    ..........auuggcacugucuaauugcag...............................................   10303      0          seq
    ..........auuggcacugucuaauugcagg..............................................   10305      0          seq
    ..........auuggcacugucuaauugcaggu.............................................   17311      0          seq
    ..........auuggcacugucuaauugcaggug............................................   403        0          seq
    ..........auuggcacugucuaauugcagguga...........................................   11         0          seq
    ..........auuggcacugucuaauugcaggugaa..........................................   2          0          seq
    ...........uggcacugucuaauugc..................................................   1          0          seq
    ...........uggcacugucuaauugca.................................................   9          0          seq
    ...........uggcacugucuaauugcag................................................   8          0          seq
    ...........uggcacugucuaauugcagg...............................................   13         0          seq
    ...........uggcacugucuaauugcaggu..............................................   41         0          seq
    ...........uggcacugucuaauugcaggug.............................................   1          0          seq
    .............ggcacugucuaauugcagg..............................................   1          0          seq
    .............ggcacugucuaauugcaggu.............................................   2          0          seq
    ..............................................cugugauagaaaguguca..............   1          0          seq
    ..............................................cugugauagaaagugucaaa............   1          0          seq
    ..............................................cugugauagaaagugucaaaa...........   3          0          seq
    ..............................................cugugauagaaagugucaaaau..........   26         0          seq
    ...............................................ugugauagaaagugucaaaau..........   1          0          seq
```
