# Supplementary material for: Identification of Conserved and Novel MicroRNAs in the Pacific Oyster Crassostrea gigas by Deep Sequencing
Source: PLoS One. 2014 Aug 19;9(8):e104371. doi: 10.1371/journal.pone.0104371 (PMC4138081; doi:10.1371/journal.pone.0104371)
Supplement: File S2 — The compressed/ZIP file archive for the predicted precursors' secondary structures and reads alignment. (ZIP) [file pone.0104371.s010.zip › second structure and reads alignment for oyster miRNAs/novel in table S5/m0103.pdf]

miRBase precursor : m0103
Total read count : 13588
m0103_5p read count : 13282
m0103_3p read count : 305
remaining reads : 1

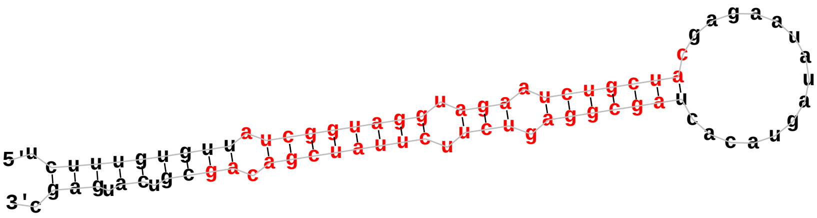

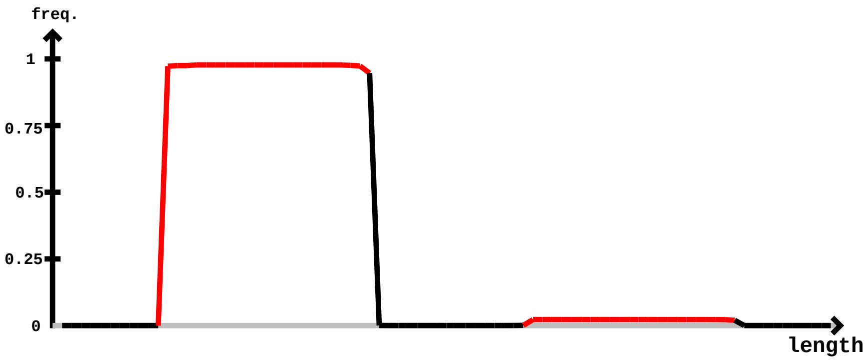


```
                                                m0103_3p
           m0103_5p
5'- ucuuuguguuaucgguagguagaaucugcuacgagaauauaguacacuagcggagucuucuuaucgacagcgucaugagc   -3'    exp
    .(((((((((.((((((((.(((.(((((((................))))))).))).)))))))).)))).)).))).    reads       mm          sample
    .........uaucgguagguagaaucugcua.................................................    2           0           seq
    ..........aucgguagguagaaucug....................................................    1           0           seq
    ..........aucgguagguagaaucugc...................................................    24          0           seq
    ..........aucgguagguagaaucugcu..................................................    28          0           seq
    ..........aucgguagguagaaucugcua.................................................    359         0           seq
    ..........aucgguagguagaaucugcuac................................................    12801       0           seq
    ..........aucgguagguagaaucugcuacg...............................................    2           0           seq
    ..........aucgguagguagaaucugcuacgagaa...........................................    1           0           seq
    ...........ucgguagguagaaucugc...................................................    1           0           seq
    ...........ucgguagguagaaucugcua.................................................    3           0           seq
    ...........ucgguagguagaaucugcuac................................................    24          0           seq
    ............cgguagguagaaucugcuac................................................    1           0           seq
    .............gguagguagaaucugcuac................................................    35          0           seq
    ................................gagaauauaguacacuagcggagucuucu...................    1           0           seq
    ..............................................cuagcggagucuucuuaucg..............    1           0           seq
    ..............................................cuagcggagucuucuuaucgacag..........    1           0           seq
    ...............................................uagcggagucuucuuaucga.............    1           0           seq
    ...............................................uagcggagucuucuuaucgaca...........    5           0           seq
    ...............................................uagcggagucuucuuaucgacag..........    1           0           seq
    ................................................agcggagucuucuuaucgac............    3           0           seq
    ................................................agcggagucuucuuaucgaca...........    25          0           seq
    ................................................agcggagucuucuuaucgacag..........    265         0           seq
    ................................................agcggagucuucuuaucgacagc.........    1           0           seq
    .................................................gcggagucuucuuaucgacag..........    2           0           seq
```
