# Supplementary material for: Identification of Conserved and Novel MicroRNAs in the Pacific Oyster Crassostrea gigas by Deep Sequencing
Source: PLoS One. 2014 Aug 19;9(8):e104371. doi: 10.1371/journal.pone.0104371 (PMC4138081; doi:10.1371/journal.pone.0104371)
Supplement: File S2 — The compressed/ZIP file archive for the predicted precursors' secondary structures and reads alignment. (ZIP) [file pone.0104371.s010.zip › second structure and reads alignment for oyster miRNAs/novel in table S5/m0146.pdf]

miRBase precursor	: m0146
Total read count	: 3592
m0146_5p read count	: 15
m0146_3p read count	: 3577
remaining reads	: 0

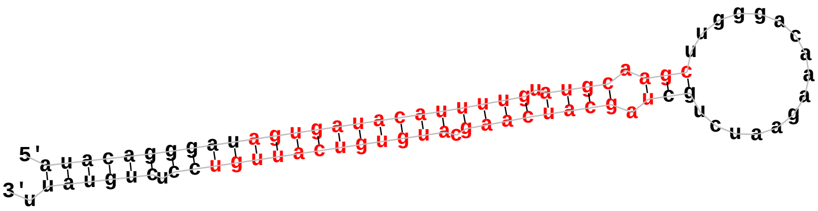

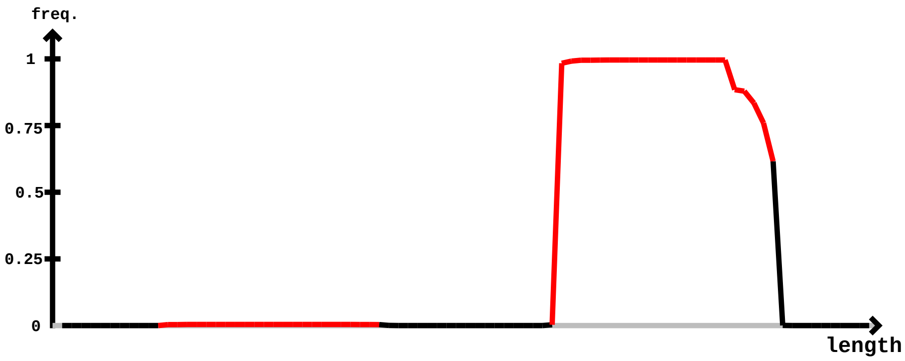


```
                                                    m0146_3p
           m0146_5p
5'- auacagggauagugauacauuuuguaugcaagcuugggacaaagaaucugcuagcaucaagcaugugucauuguccucuguauu  -3'    exp
    ((((((((((((((((((((((((.((((.(((................))).)))))))).)))))))))))))).)))))).   reads      mm         sample
    ..........agugauacauuuuguaugca......................................................   1          0          seq
    ..........agugauacauuuuguaugcaag....................................................   1          0          seq
    ..........agugauacauuuuguaugcaagc...................................................   11         0          seq
    ............ugauacauuuuguaugcaagcu..................................................   2          0          seq
    ..................................................cuagcaucaagcaugugucau.............   1          0          seq
    ..................................................cuagcaucaagcaugugucauu............   3          0          seq
    ..................................................cuagcaucaagcaugugucauug...........   1          0          seq
    ..................................................cuagcaucaagcaugugucauugu..........   6          0          seq
    ...................................................uagcaucaagcaugugu c...............   400        0          seq
    ...................................................uagcaucaagcaugugucа..............   20         0          seq
    ...................................................uagcaucaagcaugugucau.............   158        0          seq
    ...................................................uagcaucaagcaugugucauu............   263        0          seq
    ...................................................uagcaucaagcaugugucauug...........   507        0          seq
    ...................................................uagcaucaagcaugugucauugu..........   2172       0          seq
    ...................................................uagcaucaagcaugugucauuguc.........   2          0          seq
    ....................................................agcaucaagcaugugucau.............   1          0          seq
    ....................................................agcaucaagcaugugucauu............   2          0          seq
    ....................................................agcaucaagcaugugucauug...........   7          0          seq
    ....................................................agcaucaagcaugugucauugu..........   19         0          seq
    .....................................................gcaucaagcaugugucauug...........   1          0          seq
    .....................................................gcaucaagcaugugucauugu..........   11         0          seq
    .......................................................aucaagcaugugucauug...........   1          0          seq
    .......................................................aucaagcaugugucauugu..........   1          0          seq
    ........................................................ucaagcaugugucauugu..........   1          0          seq
```
