# Supplementary material for: Identification of Conserved and Novel MicroRNAs in the Pacific Oyster Crassostrea gigas by Deep Sequencing
Source: PLoS One. 2014 Aug 19;9(8):e104371. doi: 10.1371/journal.pone.0104371 (PMC4138081; doi:10.1371/journal.pone.0104371)
Supplement: File S2 — The compressed/ZIP file archive for the predicted precursors' secondary structures and reads alignment. (ZIP) [file pone.0104371.s010.zip › second structure and reads alignment for oyster miRNAs/novel in table S5/m0158.pdf]

miRBase precursor : m0158
Total read count : 4978
m0158_5p read count : 81
m0158_3p read count : 4895
remaining reads : 2

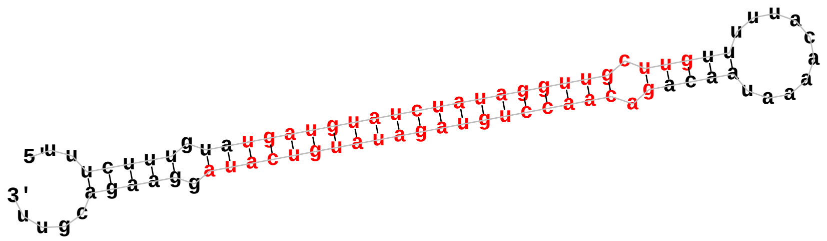

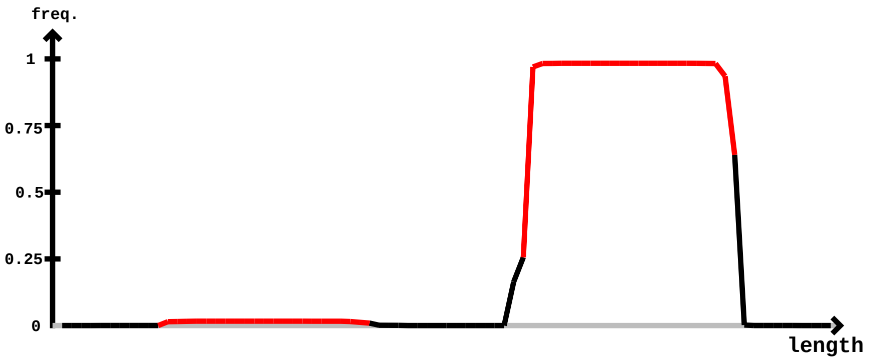


```
                  m0158_5p
                                              m0158_3p
5'- uuucuuuguaugauguaucuauagguugcuuguuuuuacaaaauaacagacaaccuguagauaugucauaggaagacguu   -3'    exp
    ..(((((.((((((((((((((((((((.(((((..........))))).)))))))))))))))))))).)))))....    reads   mm   sample
    ...cuuuguaugauguaucuauagg.......................................................    1       0    seq
    ..........ugauguaucuauagguugc...................................................    6       0    seq
    ..........ugauguaucuauagguugcu..................................................    14      0    seq
    ..........ugauguaucuauagguugcuu.................................................    13      0    seq
    ..........ugauguaucuauagguugcuug................................................    38      0    seq
    ...........gauguaucuauagguugcuu.................................................    1       0    seq
    ...........gauguaucuauagguugcuuguu..............................................    1       0    seq
    ...........gauguaucuauagguugcuuguuu.............................................    1       0    seq
    ............auguaucuauagguugcu..................................................    1       0    seq
    ............auguaucuauagguugcuug................................................    2       0    seq
    ............auguaucuauagguugcuugu...............................................    1       0    seq
    .............uguaucuauagguugcuuguuu.............................................    3       0    seq
    ..............................................cagacaaccuguagauauguca............    129     0    seq
    ..............................................cagacaaccuguagauaugucau...........    651     0    seq
    ..............................................cagacaaccuguagauaugucaua..........    38      0    seq
    ...............................................agacaaccuguagauaug...............    1       0    seq
    ...............................................agacaaccuguagauauguc.............    1       0    seq
    ...............................................agacaaccuguagauauguca............    48      0    seq
    ...............................................agacaaccuguagauaugucau...........    365     0    seq
    ...............................................agacaaccuguagauaugucaua..........    41      0    seq
    ................................................gacaaccuguagauaugu..............    2       0    seq
    ................................................gacaaccuguagauauguc.............    1       0    seq
    ................................................gacaaccuguagauauguca............    56      0    seq
    ................................................gacaaccuguagauaugucau...........    443     0    seq
    ................................................gacaaccuguagauaugucaua..........    3043    0    seq
    ................................................gacaaccuguagauaugucauag.........    8       0    seq
    .................................................acaaccuguagauauguca............    3       0    seq
    .................................................acaaccuguagauaugucau...........    10      0    seq
    .................................................acaaccuguagauaugucaua..........    51      0    seq
    .................................................acaaccuguagauaugucauag.........    1       0    seq
    ..................................................caaccuguagauaugucaua..........    1       0    seq
    ...................................................aaccuguagauaugucau...........    1       0    seq
    ...................................................aaccuguagauaugucaua..........    1       0    seq
    ...................................................aaccuguagauaugucauaggaaga....    1       0    seq
```

m0158_5p

m0158_3p

uuucuuuguaugauguaucuauagguugcuuguuuuuacaaaauaacagacaaccuguagauaugucauaggaagacguu
