# Supplementary material for: Identification of Conserved and Novel MicroRNAs in the Pacific Oyster Crassostrea gigas by Deep Sequencing
Source: PLoS One. 2014 Aug 19;9(8):e104371. doi: 10.1371/journal.pone.0104371 (PMC4138081; doi:10.1371/journal.pone.0104371)
Supplement: File S2 — The compressed/ZIP file archive for the predicted precursors' secondary structures and reads alignment. (ZIP) [file pone.0104371.s010.zip › second structure and reads alignment for oyster miRNAs/novel in table S5/m0214.pdf]

miRBase precursor	: m0214
Total read count	: 1034
m0214_5p read count	: 831
m0214_3p read count	: 203
remaining reads	: 0

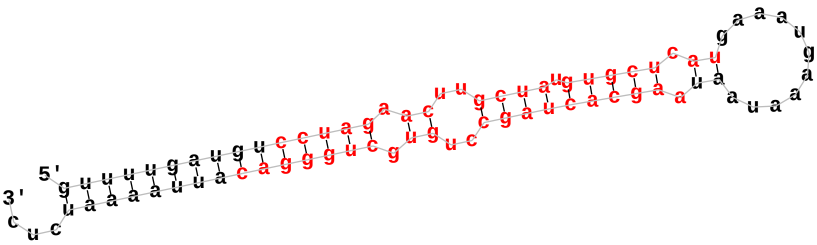

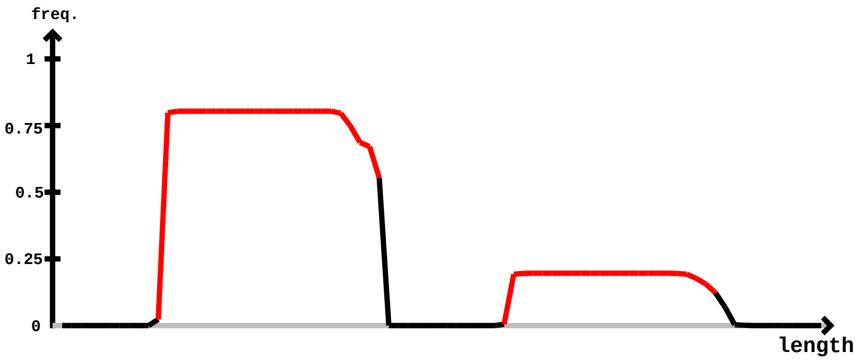


```
                                                m0214_3p
            m0214_5p
5'- guuuugauguccuagaacuugcuaugugcucaugaaaugaaauaauaagcacuagccugugcugggacauuaaaaucuc   -3'    exp
    ((((((((((((((.((..((((.(((((.((.............)).)))))))))..)).))))))))))))))...    reads      mm         sample
    .........uccuagaacuugcuaugugc..................................................    2          0          seq
    .........uccuagaacuugcuaugugcu.................................................    9          0          seq
    .........uccuagaacuugcuaugugcuc................................................    3          0          seq
    .........uccuagaacuugcuaugugcuca...............................................    10         0          seq
    ..........ccuagaacuugcuaugug...................................................    8          0          seq
    ..........ccuagaacuugcuaugugc..................................................    47         0          seq
    ..........ccuagaacuugcuaugugcu.................................................    53         0          seq
    ..........ccuagaacuugcuaugugcuc................................................    13         0          seq
    ..........ccuagaacuugcuaugugcuca...............................................    110        0          seq
    ..........ccuagaacuugcuaugugcucau..............................................    570        0          seq
    ...........cuagaacuugcuaugugcu.................................................    2          0          seq
    ...........cuagaacuugcuaugugcuca...............................................    1          0          seq
    ...........cuagaacuugcuaugugcucau..............................................    3          0          seq
    .............................................uaagcacuagccugugcu................    1          0          seq
    .............................................uaagcacuagccugugcugg..............    2          0          seq
    .............................................uaagcacuagccugugcuggga............    2          0          seq
    ..............................................aagcacuagccugugcug...............    3          0          seq
    ..............................................aagcacuagccugugcugg..............    14         0          seq
    ..............................................aagcacuagccugugcuggg.............    21         0          seq
    ..............................................aagcacuagccugugcuggga............    29         0          seq
    ..............................................aagcacuagccugugcugggac...........    56         0          seq
    ..............................................aagcacuagccugugcugggaca..........    68         0          seq
    ..............................................aagcacuagccugugcugggacau.........    2          0          seq
    ..............................................aagcacuagccugugcugggacauu........    1          0          seq
    ...............................................agcacuagccugugcuggg.............    1          0          seq
    ...............................................agcacuagccugugcuggga............    2          0          seq
    ................................................gcacuagccugugcuggga............    1          0          seq
```
