# Supplementary material for: Identification of Conserved and Novel MicroRNAs in the Pacific Oyster Crassostrea gigas by Deep Sequencing
Source: PLoS One. 2014 Aug 19;9(8):e104371. doi: 10.1371/journal.pone.0104371 (PMC4138081; doi:10.1371/journal.pone.0104371)
Supplement: File S2 — The compressed/ZIP file archive for the predicted precursors' secondary structures and reads alignment. (ZIP) [file pone.0104371.s010.zip › second structure and reads alignment for oyster miRNAs/novel in table S5/m0235.pdf]

miRBase precursor	: m0235
Total read count	: 112
m0235_5p read count	: 95
m0235_3p read count	: 17
remaining reads	: 0

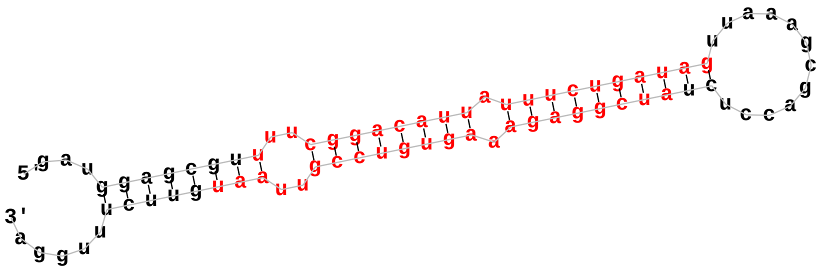

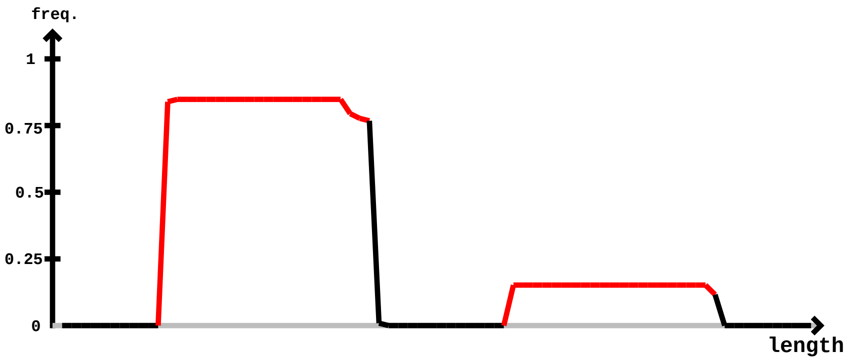

m0235_3p

m0235_5p

```
5'- gauggagcguuuucggacauuauuucugauaguuaaagcgaccucuaucggagaaaguguccguuaauguucuuugga   -3'    exp
    ...((((((((..((((((((.((((((((((............)))))))))).))))))))..)))))))).....    reads      mm         sample
    ..........uuucggacauuauuucuga.................................................    6          0          seq
    ..........uuucggacauuauuucugau................................................    2          0          seq
    ..........uuucggacauuauuucugaua...............................................    1          0          seq
    ..........uuucggacauuauuucugauag..............................................    85         0          seq
    ...........uucggacauuauuucugauagu.............................................    1          0          seq
    ..............................................aucggagaaaguguccguuaa...........    4          0          seq
    ..............................................aucggagaaaguguccguuaau..........    13         0          seq
```
