# Supplementary material for: Identification of Conserved and Novel MicroRNAs in the Pacific Oyster Crassostrea gigas by Deep Sequencing
Source: PLoS One. 2014 Aug 19;9(8):e104371. doi: 10.1371/journal.pone.0104371 (PMC4138081; doi:10.1371/journal.pone.0104371)
Supplement: File S2 — The compressed/ZIP file archive for the predicted precursors' secondary structures and reads alignment. (ZIP) [file pone.0104371.s010.zip › second structure and reads alignment for oyster miRNAs/novel in table S5/m0250.pdf]

miRBase precursor : m0250
Total read count : 4674562
m0250_5p read count : 22938
m0250_3p read count : 4651566
remaining reads : 58

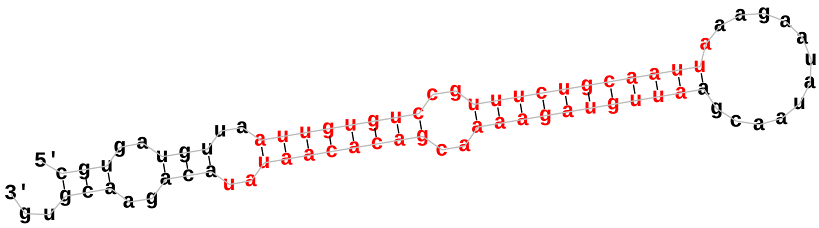

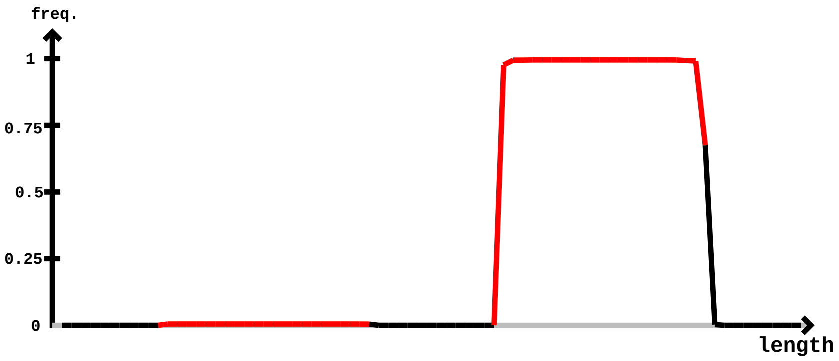


```
                           m0250_5p
                                                                 m0250_3p
5'- cgugauguuaauuguguccguuucugcaauuaaagaauauaacgaauuguagaaaacgacacaauauacagaacgug   -3'    exp
    (((..(((..((((((((..(((((((((((.............)))))))))))..))))))))..)))..)))..    reads      mm         sample
    ........uaauuguguccguuucug...................................................    1          0          seq
    ........uaauuguguccguuucugcaa................................................    1          0          seq
    ........uaauuguguccguuucugcaau...............................................    2          0          seq
    .........aauuguguccguuucugcaauua.............................................    6          0          seq
    ..........auuguguccguuucugca.................................................    83         0          seq
    ..........auuguguccguuucugcaa................................................    156        0          seq
    ..........auuguguccguuucugcaau...............................................    394        0          seq
    ..........auuguguccguuucugcaauu..............................................    1198       0          seq
    ..........auuguguccguuucugcaauua.............................................    20760      0          seq
    ..........auuguguccguuucugcaauuaa............................................    61         0          seq
    ..........auuguguccguuucugcaauuaaa...........................................    5          0          seq
    ..........auuguguccguuucugcaauuaaag..........................................    1          0          seq
    ...........uuguguccguuucugcaa................................................    1          0          seq
    ...........uuguguccguuucugcaau...............................................    6          0          seq
    ...........uuguguccguuucugcaauu..............................................    13         0          seq
    ...........uuguguccguuucugcaauua.............................................    236        0          seq
    ...........uuguguccguuucugcaauuaa............................................    3          0          seq
    ............uguguccguuucugcaauua.............................................    1          0          seq
    .............guguccguuucugcaauu..............................................    2          0          seq
    .............guguccguuucugcaauua.............................................    8          0          seq
    ........................................aacgaauuguagaaaacgacac...............    5          0          seq
    ........................................aacgaauuguagaaaacgacacaa.............    1          0          seq
    ........................................aacgaauuguagaaaacgacacaaua...........    7          0          seq
    ........................................aacgaauuguagaaaacgacacaauau..........    6          0          seq
    .........................................acgaauuguagaaaacgaca................    1          0          seq
    .........................................acgaauuguagaaaacgacaca..............    1          0          seq
    .........................................acgaauuguagaaaacgacacaaua...........    4          0          seq
    .........................................acgaauuguagaaaacgacacaauau..........    18         0          seq
    ..........................................cgaauuguagaaaacgacacaa.............    1          0          seq
    ..........................................cgaauuguagaaaacgacacaau............    1          0          seq
    ..........................................cgaauuguagaaaacgacacaaua...........    4          0          seq
    ..........................................cgaauuguagaaaacgacacaauau..........    9          0          seq
    ...........................................gaauuguagaaaacgacacaau............    3          0          seq
    ...........................................gaauuguagaaaacgacacaaua...........    3          0          seq
```

```
          m0250_5p
                                                     m0250_3p
cgugauguuaauuguguccguuucugcaauuaaagaauauaacgaauuguagaaaacgacacaauauacagaacgug

...........................................gaauuguagaaaacgacacaauau..........	14	0	seq
............................................aauuguagaaaacgacacaa.............	10	0	seq
............................................aauuguagaaaacgacacaau............	10	0	seq
............................................aauuguagaaaacgacacaaua...........	73	0	seq
............................................aauuguagaaaacgacacaauau..........	248	0	seq
............................................aauuguagaaaacgacacaauaua.........	6	0	seq
.............................................auuguagaaaacgacaca..............	227	0	seq
.............................................auuguagaaaacgacacaa.............	10027	0	seq
.............................................auuguagaaaacgacacaau............	7658	0	seq
.............................................auuguagaaaacgacacaaua...........	1453669	0	seq
.............................................auuguagaaaacgacacaauau..........	3078788	0	seq
.............................................auuguagaaaacgacacaauaua.........	11847	0	seq
.............................................auuguagaaaacgacacaauauac........	10	0	seq
.............................................auuguagaaaacgacacaauauaca.......	2	0	seq
.............................................auuguagaaaacgacacaauauacag......	1	0	seq
..............................................uuguagaaaacgacacaa.............	139	0	seq
..............................................uuguagaaaacgacacaau............	106	0	seq
..............................................uuguagaaaacgacacaaua...........	24889	0	seq
..............................................uuguagaaaacgacacaauau..........	60271	0	seq
..............................................uuguagaaaacgacacaauaua.........	858	0	seq
..............................................uuguagaaaacgacacaauauac........	1	0	seq
...............................................uguagaaaacgacacaaua...........	119	0	seq
...............................................uguagaaaacgacacaauau..........	244	0	seq
...............................................uguagaaaacgacacaauaua.........	7	0	seq
................................................guagaaaacgacacaaua...........	448	0	seq
................................................guagaaaacgacacaauau..........	1865	0	seq
................................................guagaaaacgacacaauaua.........	7	0	seq
.................................................uagaaaacgacacaauau..........	16	0	seq
```
