# Supplementary material for: Identification of Conserved and Novel MicroRNAs in the Pacific Oyster Crassostrea gigas by Deep Sequencing
Source: PLoS One. 2014 Aug 19;9(8):e104371. doi: 10.1371/journal.pone.0104371 (PMC4138081; doi:10.1371/journal.pone.0104371)
Supplement: File S2 — The compressed/ZIP file archive for the predicted precursors' secondary structures and reads alignment. (ZIP) [file pone.0104371.s010.zip › second structure and reads alignment for oyster miRNAs/novel in table S5/m0315.pdf]

miRBase precursor : m0315
Total read count : 30306
m0315_5p read count : 367
m0315_3p read count : 29937
remaining reads : 2

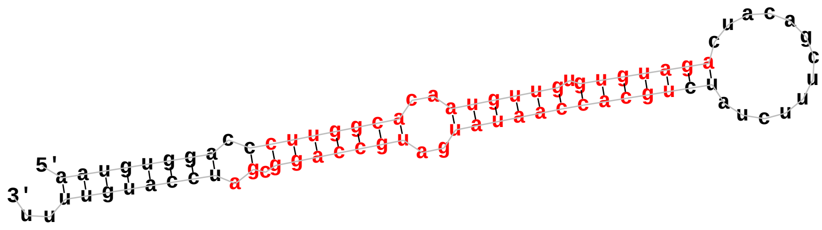

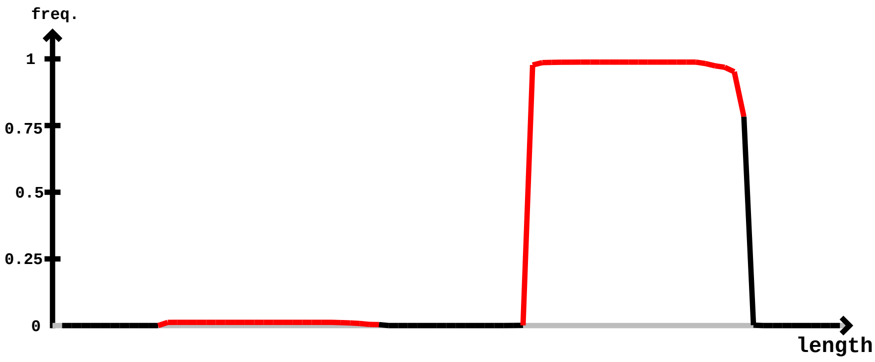

m0315_5p
m0315_3p

```
5'- aauguggacccuuggcacaauguuguguguagacuacagcuuucuaucugcaccaauaugaugccaggcgauccauguuuu   -3'    exp
    ((((((((.((((((((..((((((.(((((((.............)))))))))))))..))))))).).))))))))..    reads      mm         sample
    ..........cuuggcacaauguugugu.....................................................    32         0          seq
    ..........cuuggcacaauguugugug....................................................    37         0          seq
    ..........cuuggcacaauguugugugu...................................................    61         0          seq
    ..........cuuggcacaauguugugugua..................................................    105        0          seq
    ..........cuuggcacaauguuguguguag.................................................    21         0          seq
    ..........cuuggcacaauguuguguguaga................................................    106        0          seq
    ...........uuggcacaauguugugug....................................................    1          0          seq
    ...........uuggcacaauguugugugua..................................................    1          0          seq
    ...........uuggcacaauguuguguguaga................................................    2          0          seq
    ............uggcacaauguugugugua..................................................    1          0          seq
    ....................uguuguguguagacuacagcuuucu....................................    1          0          seq
    .............................................aucugcaccaauaugaugccaggcga..........    1          0          seq
    ..............................................ucugcaccaauaugaugccaggcga..........    8          0          seq
    ...............................................cugcaccaauaugaugcca...............    1          0          seq
    ...............................................cugcaccaauaugaugccaggcga..........    15         0          seq
    ................................................ugcaccaauaugaugcca...............    160        0          seq
    ................................................ugcaccaauaugaugccag..............    251        0          seq
    ................................................ugcaccaauaugaugccagg.............    160        0          seq
    ................................................ugcaccaauaugaugccaggc............    494        0          seq
    ................................................ugcaccaauaugaugccaggcg...........    5058       0          seq
    ................................................ugcaccaauaugaugccaggcga..........    23417      0          seq
    ................................................ugcaccaauaugaugccaggcgau.........    43         0          seq
    .................................................gcaccaauaugaugccag..............    10         0          seq
    .................................................gcaccaauaugaugccagg.............    4          0          seq
    .................................................gcaccaauaugaugccaggc............    15         0          seq
    .................................................gcaccaauaugaugccaggcg...........    40         0          seq
    .................................................gcaccaauaugaugccaggcga..........    199        0          seq
    .................................................gcaccaauaugaugccaggcgau.........    5          0          seq
    ..................................................caccaauaugaugccaggcg...........    2          0          seq
    ..................................................caccaauaugaugccaggcga..........    20         0          seq
    ..................................................caccaauaugaugccaggcgau.........    2          0          seq
    ...................................................accaauaugaugccaggc............    2          0          seq
    ...................................................accaauaugaugccaggcg...........    1          0          seq
    ...................................................accaauaugaugccaggcga..........    18         0          seq
```

```
          m0315_5p
                                                     m0315_3p
aauguggacccuuggcacaauguuguguguagacuacagcuuucuaucugcaccaauaugaugccaggcgauccauguuuu

....................................................ccaauaugaugccaggcga..........    4           0           seq
.....................................................caauaugaugccaggcga..........    8           0           seq
```
