# Supplementary material for: Identification of Conserved and Novel MicroRNAs in the Pacific Oyster Crassostrea gigas by Deep Sequencing
Source: PLoS One. 2014 Aug 19;9(8):e104371. doi: 10.1371/journal.pone.0104371 (PMC4138081; doi:10.1371/journal.pone.0104371)
Supplement: File S2 — The compressed/ZIP file archive for the predicted precursors' secondary structures and reads alignment. (ZIP) [file pone.0104371.s010.zip › second structure and reads alignment for oyster miRNAs/novel in table S5/m0317.pdf]

miRBase precursor : m0317
Total read count : 3275
m0317_5p read count : 334
m0317_3p read count : 2941
remaining reads : 0

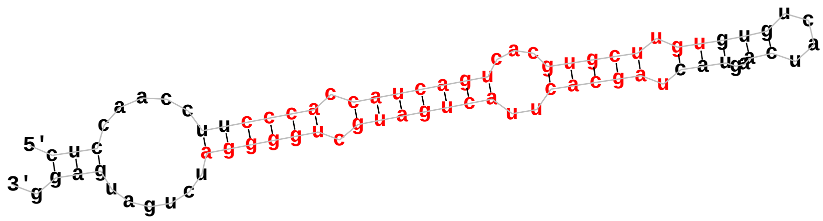

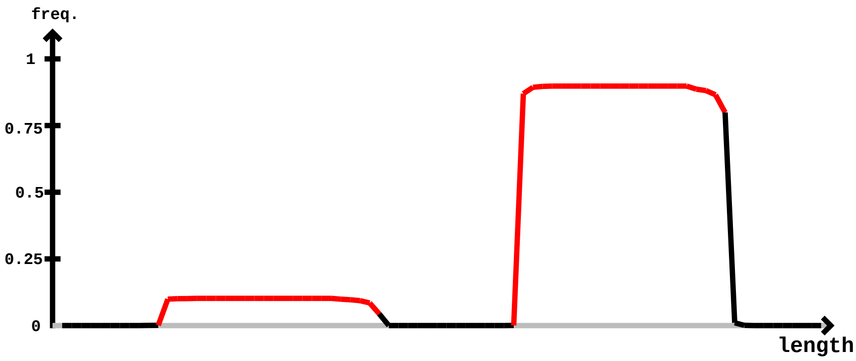


```
                                          m0317_3p
                 m0317_5p
5'- cuccaaccuucccaccaucagucacgugcuugugugucaucaaguacuagcacuuacugaugcuggggaucugaugagg   -3'   exp
    (((.....((((((.(((((((...(((((.(((((....))..))).)))))..))))))).)))))).......))).   reads     mm        sample
    ........uucccaccaucagucacgugc..................................................   1         0         seq
    ........uucccaccaucagucacgugcu.................................................   1         0         seq
    .........ucccaccaucagucacgugcuug...............................................   1         0         seq
    ..........cccaccaucagucacgug...................................................   10        0         seq
    ..........cccaccaucagucacgugc..................................................   6         0         seq
    ..........cccaccaucagucacgugcu.................................................   10        0         seq
    ..........cccaccaucagucacgugcuu................................................   26        0         seq
    ..........cccaccaucagucacgugcuug...............................................   130       0         seq
    ..........cccaccaucagucacgugcuugu..............................................   140       0         seq
    ...........ccaccaucagucacgugcuu................................................   2         0         seq
    ...........ccaccaucagucacgugcuug...............................................   2         0         seq
    ...........ccaccaucagucacgugcuugu..............................................   1         0         seq
    ............caccaucagucacgugcuug...............................................   1         0         seq
    .............accaucagucacgugcuugu..............................................   3         0         seq
    ..............................................cuagcacuuacugaugcugggga..........   2         0         seq
    ...............................................uagcacuuacugaugcug..............   38        0         seq
    ...............................................uagcacuuacugaugcugg.............   16        0         seq
    ...............................................uagcacuuacugaugcuggg............   49        0         seq
    ...............................................uagcacuuacugaugcugggg...........   213       0         seq
    ...............................................uagcacuuacugaugcugggga..........   2499      0         seq
    ...............................................uagcacuuacugaugcuggggau.........   28        0         seq
    ...............................................uagcacuuacugaugcuggggauc........   3         0         seq
    ................................................agcacuuacugaugcugg.............   1         0         seq
    ................................................agcacuuacugaugcuggg............   2         0         seq
    ................................................agcacuuacugaugcugggg...........   3         0         seq
    ................................................agcacuuacugaugcugggga..........   70        0         seq
    ................................................agcacuuacugaugcuggggau.........   2         0         seq
    .................................................gcacuuacugaugcuggg............   1         0         seq
    .................................................gcacuuacugaugcugggga..........   10        0         seq
    ..................................................cacuuacugaugcugggg...........   1         0         seq
    ..................................................cacuuacugaugcugggga..........   2         0         seq
    ..................................................cacuuacugaugcuggggau.........   1         0         seq
```
