# Supplementary material for: Identification of Conserved and Novel MicroRNAs in the Pacific Oyster Crassostrea gigas by Deep Sequencing
Source: PLoS One. 2014 Aug 19;9(8):e104371. doi: 10.1371/journal.pone.0104371 (PMC4138081; doi:10.1371/journal.pone.0104371)
Supplement: File S2 — The compressed/ZIP file archive for the predicted precursors' secondary structures and reads alignment. (ZIP) [file pone.0104371.s010.zip › second structure and reads alignment for oyster miRNAs/novel in table S5/m0347.pdf]

miRBase precursor : m0347
Total read count : 117
m0347_5p read count : 52
m0347_3p read count : 64
remaining reads : 1

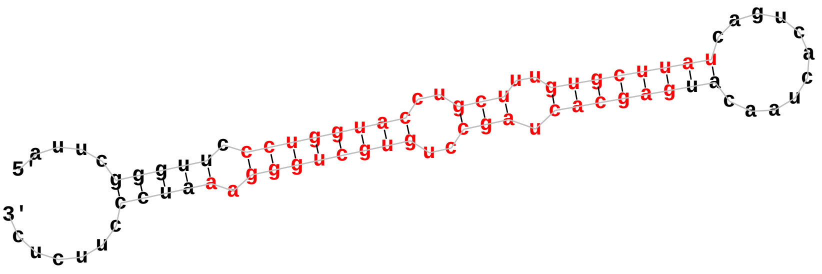

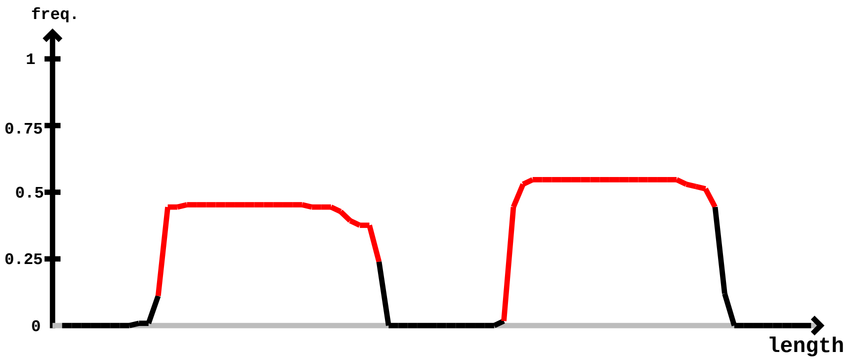


```
                                                m0347_3p
           m0347_5p
5'- auucggguucccugguaccugcuuugugcuuaucagucacuaacaugagcacuagccugugcugggaaaucccuucuc   -3'    exp
    ....(((((.((((((((..(((..((((((((..........)))))))).)))..)))))))).)))))......    reads      mm         sample
    .......uucccugguaccugcuuu.....................................................    1          0          seq
    .........cccugguaccugcuuugug..................................................    1          0          seq
    .........cccugguaccugcuuugugc.................................................    1          0          seq
    .........cccugguaccugcuuugugcu................................................    2          0          seq
    .........cccugguaccugcuuugugcuua..............................................    8          0          seq
    ..........ccugguaccugcuuugug..................................................    1          0          seq
    ..........ccugguaccugcuuugugc.................................................    3          0          seq
    ..........ccugguaccugcuuugugcuua..............................................    7          0          seq
    ..........ccugguaccugcuuugugcuuau.............................................    28         0          seq
    ............ugguaccugcuuugugcuua..............................................    1          0          seq
    .............................................ugagcacuagccugugcug..............    2          0          seq
    ..............................................gagcacuagccugugcugg.............    1          0          seq
    ..............................................gagcacuagccugugcuggga...........    5          0          seq
    ..............................................gagcacuagccugugcugggaa..........    33         0          seq
    ..............................................gagcacuagccugugcugggaaa.........    11         0          seq
    ...............................................agcacuagccugugcuggg............    1          0          seq
    ...............................................agcacuagccugugcuggga...........    2          0          seq
    ...............................................agcacuagccugugcugggaa..........    4          0          seq
    ...............................................agcacuagccugugcugggaaa.........    3          0          seq
    ................................................gcacuagccugugcuggga...........    1          0          seq
    ................................................gcacuagccugugcugggaa..........    1          0          seq
```
