# Supplementary material for: Identification of Conserved and Novel MicroRNAs in the Pacific Oyster Crassostrea gigas by Deep Sequencing
Source: PLoS One. 2014 Aug 19;9(8):e104371. doi: 10.1371/journal.pone.0104371 (PMC4138081; doi:10.1371/journal.pone.0104371)
Supplement: File S2 — The compressed/ZIP file archive for the predicted precursors' secondary structures and reads alignment. (ZIP) [file pone.0104371.s010.zip › second structure and reads alignment for oyster miRNAs/novel in table S5/m0420.pdf]

miRBase precursor : m0420
Total read count : 736094
m0420_5p read count : 736011
m0420_3p read count : 75
remaining reads : 8

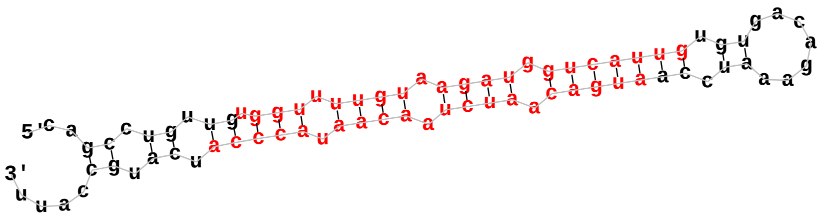

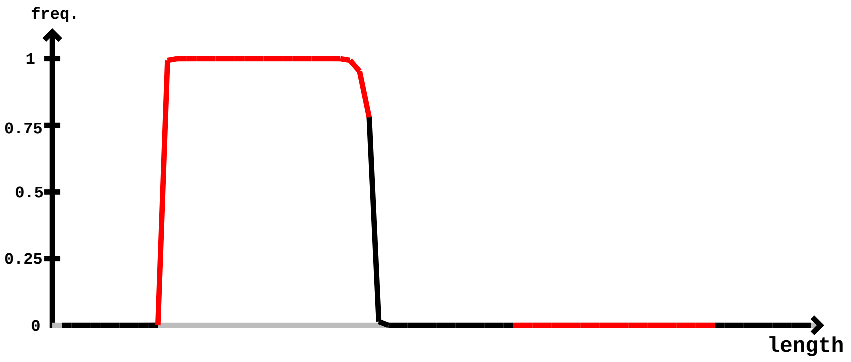


```
                                                m0420_3p
          m0420_5p
5'- cagccuguugugguuuuguaagauggucauugugugacagaaauccaaugacaaucuaacaauacccaucaugccauu   -3'   exp
    ..((.((.((.(((.((((.((((.(((((((.((.......)).))))))).)))).)))).))))).)).))....    reads      mm      sample
    .......uugugguuuuguaagaugguca.................................................    7          0       seq
    ........ugugguuuuguaagauggu...................................................    2          0       seq
    ........ugugguuuuguaagaugguca.................................................    2          0       seq
    ........ugugguuuuguaagauggucau................................................    29         0       seq
    ........ugugguuuuguaagauggucauu...............................................    3          0       seq
    .........gugguuuuguaagaugguc..................................................    1          0       seq
    .........gugguuuuguaagaugguca.................................................    1          0       seq
    .........gugguuuuguaagauggucau................................................    13         0       seq
    .........gugguuuuguaagauggucauu...............................................    69         0       seq
    .........gugguuuuguaagauggucauug..............................................    2          0       seq
    ..........ugguuuuguaagaugguc..................................................    179        0       seq
    ..........ugguuuuguaagaugguca.................................................    4298       0       seq
    ..........ugguuuuguaagauggucau................................................    30087      0       seq
    ..........ugguuuuguaagauggucauu...............................................    126710     0       seq
    ..........ugguuuuguaagauggucauug..............................................    559865     0       seq
    ..........ugguuuuguaagauggucauugu.............................................    9999       0       seq
    ..........ugguuuuguaagauggucauugug............................................    6          0       seq
    ..........ugguuuuguaagauggucauugugugaca.......................................    1          0       seq
    ...........gguuuuguaagaugguca.................................................    30         0       seq
    ...........gguuuuguaagauggucau................................................    85         0       seq
    ...........gguuuuguaagauggucauu...............................................    491        0       seq
    ...........gguuuuguaagauggucauug..............................................    3768       0       seq
    ...........gguuuuguaagauggucauugu.............................................    206        0       seq
    ............guuuuguaagauggucau................................................    4          0       seq
    ............guuuuguaagauggucauu...............................................    10         0       seq
    ............guuuuguaagauggucauug..............................................    61         0       seq
    ............guuuuguaagauggucauugu.............................................    4          0       seq
    ............guuuuguaagauggucauugug............................................    2          0       seq
    .............uuuuguaagauggucauu...............................................    11         0       seq
    .............uuuuguaagauggucauug..............................................    45         0       seq
    ..............uuuguaagauggucauug..............................................    25         0       seq
    ...............uuguaagauggucauugu.............................................    3          0       seq
    ..............................................aaugacaaucuaacaaua..............    1          0       seq
    ..............................................aaugacaaucuaacaauaccc...........    2          0       seq
```

```
                                             m0420_3p
         m0420_5p
cagccuguugugguuuuguaagauggucauugugugacagaaauccaaugacaaucuaacaauacccaucaugccauu

.............................................aaugacaaucuaacaauaccca..........    14         0          seq
.............................................aaugacaaucuaacaauacccau.........    4          0          seq
..............................................augacaaucuaacaauac.............    2          0          seq
..............................................augacaaucuaacaauacc............    4          0          seq
..............................................augacaaucuaacaauaccc...........    5          0          seq
..............................................augacaaucuaacaauaccca..........    42         0          seq
..............................................augacaaucuaacaauacccau.........    1          0          seq
```
