# Supplementary material for: Identification of Conserved and Novel MicroRNAs in the Pacific Oyster Crassostrea gigas by Deep Sequencing
Source: PLoS One. 2014 Aug 19;9(8):e104371. doi: 10.1371/journal.pone.0104371 (PMC4138081; doi:10.1371/journal.pone.0104371)
Supplement: File S2 — The compressed/ZIP file archive for the predicted precursors' secondary structures and reads alignment. (ZIP) [file pone.0104371.s010.zip › second structure and reads alignment for oyster miRNAs/novel in table S5/m0443.pdf]

miRBase precursor : m0443
Total read count : 105
m0443_5p read count : 1
m0443_3p read count : 104
remaining reads : 0

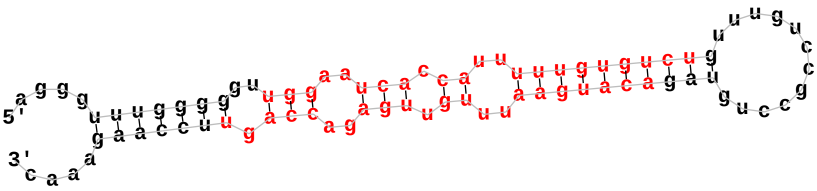

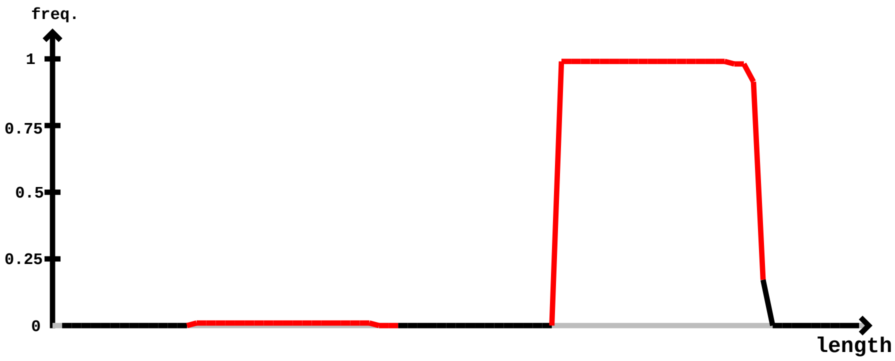


```
                                                  m0443_3p
             m0443_5p
5'- aggguuuggggguuggaaucaccauuuuugugucuguuuguccgccuguagacaugaauuuguugagaccaguuccaagaaac   -3'    exp
    ....(((((((..(((..(((.((..((((((((((............))))))))))..)).)))..))).)))))))....     reads      mm         sample
    .............uggaaucaccauuuuugug...................................................     1          0          seq
    ...................................................acaugaauuuguugagac..............     1          0          seq
    ...................................................acaugaauuuguugagacca............     7          0          seq
    ...................................................acaugaauuuguugagaccag...........     78         0          seq
    ...................................................acaugaauuuguugagaccagu..........     18         0          seq
```
