# Supplementary material for: Identification of Conserved and Novel MicroRNAs in the Pacific Oyster Crassostrea gigas by Deep Sequencing
Source: PLoS One. 2014 Aug 19;9(8):e104371. doi: 10.1371/journal.pone.0104371 (PMC4138081; doi:10.1371/journal.pone.0104371)
Supplement: File S2 — The compressed/ZIP file archive for the predicted precursors' secondary structures and reads alignment. (ZIP) [file pone.0104371.s010.zip › second structure and reads alignment for oyster miRNAs/novel in table S5/m0489.pdf]

miRBase precursor	: m0489
Total read count	: 2125
m0489_5p read count	: 1607
m0489_3p read count	: 518
remaining reads	: 0

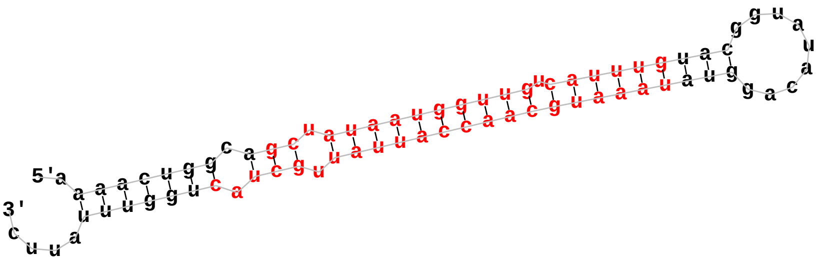

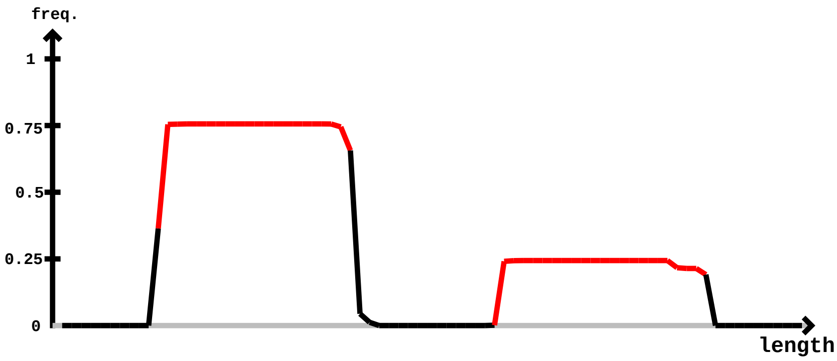


```
                m0489_5p
                                  m0489_3p
5'- aaaacuggcagcuauaaugguugucauuuguacgguauacagguauaaaugcaaccauuauugcuacugguuuauuc   -3'    exp
    .(((((((.(((.((((((((((.(((((((((.........))))))))))))))))))).))).)))))))....    reads      mm         sample
    .........agcuauaaugguugucau..................................................    1          0          seq
    .........agcuauaaugguugucauu.................................................    10         0          seq
    .........agcuauaaugguugucauuu................................................    100        0          seq
    .........agcuauaaugguugucauuug...............................................    621        0          seq
    .........agcuauaaugguugucauuugu..............................................    27         0          seq
    .........agcuauaaugguugucauuugua.............................................    15         0          seq
    ..........gcuauaaugguugucauu.................................................    13         0          seq
    ..........gcuauaaugguugucauuu................................................    88         0          seq
    ..........gcuauaaugguugucauuug...............................................    677        0          seq
    ..........gcuauaaugguugucauuugu..............................................    41         0          seq
    ..........gcuauaaugguugucauuugua.............................................    10         0          seq
    ...........cuauaaugguugucauuug...............................................    2          0          seq
    ............uauaaugguugucauuug...............................................    1          0          seq
    ............uauaaugguugucauuugua.............................................    1          0          seq
    ............................................auaaaugcaaccauuauugcua...........    2          0          seq
    ............................................auaaaugcaaccauuauugcuac..........    1          0          seq
    .............................................uaaaugcaaccauuauug..............    57         0          seq
    .............................................uaaaugcaaccauuauugc.............    5          0          seq
    .............................................uaaaugcaaccauuauugcua...........    47         0          seq
    .............................................uaaaugcaaccauuauugcuac..........    400        0          seq
    ..............................................aaaugcaaccauuauugc.............    1          0          seq
    ..............................................aaaugcaaccauuauugcuac..........    4          0          seq
    ...............................................aaugcaaccauuauugcuac..........    1          0          seq
```
