# Supplementary material for: Identification of Conserved and Novel MicroRNAs in the Pacific Oyster Crassostrea gigas by Deep Sequencing
Source: PLoS One. 2014 Aug 19;9(8):e104371. doi: 10.1371/journal.pone.0104371 (PMC4138081; doi:10.1371/journal.pone.0104371)
Supplement: File S2 — The compressed/ZIP file archive for the predicted precursors' secondary structures and reads alignment. (ZIP) [file pone.0104371.s010.zip › second structure and reads alignment for oyster miRNAs/potential in table S7/m0044.pdf]

miRBase precursor : m0044
Total read count : 104
m0044_5p read count : 102
m0044_3p read count : 2
remaining reads : 0

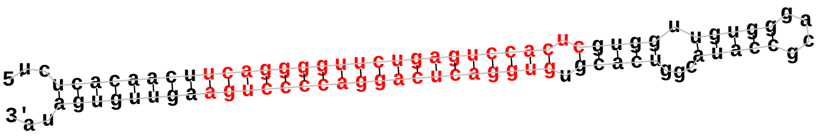

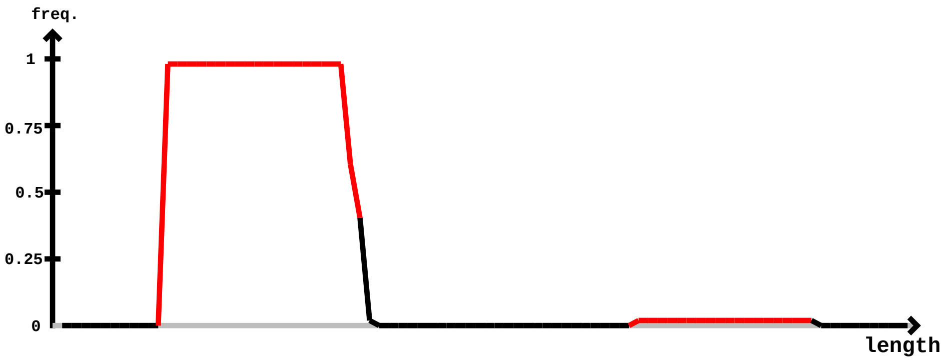

m0044_5p

m0044_3p

```
5'- ucucacaacuucagggguucugaguccacucgugguugugggacgccauacggucacguguggacucaggaccccugaaguugugaua   -3'    exp
    ..(((((((((((((((((((((((((((.(((((.(((((....)))))...))))).))))))))))))))))))))))))))).. 	reads	mm	sample
    ..........ucagggguucugaguccac...........................................................	39	0	seq
    ..........ucagggguucugaguccacu..........................................................	21	0	seq
    ..........ucagggguucugaguccacuc.........................................................	40	0	seq
    ..........ucagggguucugaguccacucg........................................................	2	0	seq
    ...........................................................guggacucaggaccccuga..........	2	0	seq
```
