# Supplementary material for: Identification of Conserved and Novel MicroRNAs in the Pacific Oyster Crassostrea gigas by Deep Sequencing
Source: PLoS One. 2014 Aug 19;9(8):e104371. doi: 10.1371/journal.pone.0104371 (PMC4138081; doi:10.1371/journal.pone.0104371)
Supplement: File S2 — The compressed/ZIP file archive for the predicted precursors' secondary structures and reads alignment. (ZIP) [file pone.0104371.s010.zip › second structure and reads alignment for oyster miRNAs/potential in table S7/m0054.pdf]

miRBase precursor : m0054
Total read count : 47835
m0054_5p read count : 20770
m0054_3p read count : 27062
remaining reads : 3

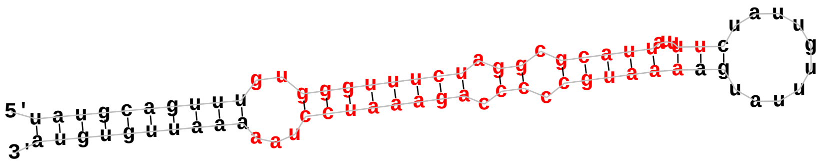

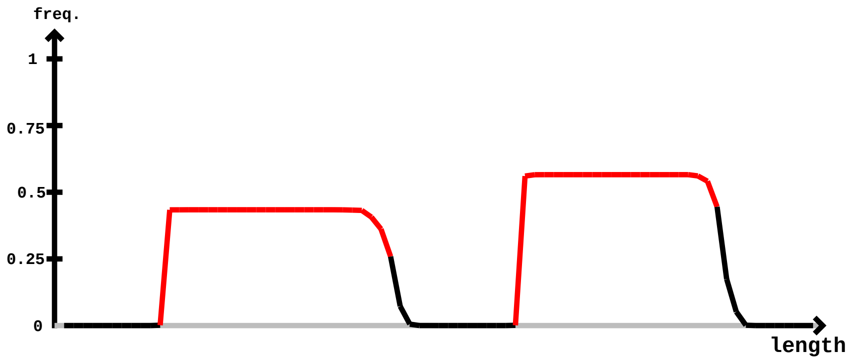


```
                                           m0054_3p
            m0054_5p
5'- uaugcaguuuguggguuucuaggcgcauuauuuucuauuguuuaugaaaaugccccagaaauccuaaaaauugugua   -3'   exp
    ((((((((((..((((((((.((.(((((...(((.........))))))))).)).))))))))...))))))))))   reads    mm    sample
    .......uuuguggguuucuaggcgcau.................................................   1        0     seq
    ........uuguggguuucuaggcgcauua...............................................   1        0     seq
    .........uguggguuucuaggcgcauu................................................   1        0     seq
    .........uguggguuucuaggcgcauua...............................................   1        0     seq
    .........uguggguuucuaggcgcauuau..............................................   15       0     seq
    .........uguggguuucuaggcgcauuauu.............................................   19       0     seq
    .........uguggguuucuaggcgcauuauuu............................................   11       0     seq
    .........uguggguuucuaggcgcauuauuuu...........................................   15       0     seq
    ..........guggguuucuaggcgcau.................................................   14       0     seq
    ..........guggguuucuaggcgcauu................................................   42       0     seq
    ..........guggguuucuaggcgcauua...............................................   50       0     seq
    ..........guggguuucuaggcgcauuau..............................................   1190     0     seq
    ..........guggguuucuaggcgcauuauu.............................................   2122     0     seq
    ..........guggguuucuaggcgcauuauuu............................................   4921     0     seq
    ..........guggguuucuaggcgcauuauuuu...........................................   8894     0     seq
    ..........guggguuucuaggcgcauuauuuuc..........................................   3200     0     seq
    ..........guggguuucuaggcgcauuauuuucu.........................................   254      0     seq
    ..........guggguuucuaggcgcauuauuuucua........................................   3        0     seq
    ..........guggguuucuaggcgcauuauuuucuau.......................................   1        0     seq
    ...........uggguuucuaggcgcauuauuu............................................   1        0     seq
    ...........uggguuucuaggcgcauuauuuu...........................................   4        0     seq
    ...........uggguuucuaggcgcauuauuuuc..........................................   2        0     seq
    ............ggguuucuaggcgcauuau..............................................   1        0     seq
    ............ggguuucuaggcgcauuauuu............................................   2        0     seq
    ............ggguuucuaggcgcauuauuuu...........................................   2        0     seq
    ............ggguuucuaggcgcauuauuuuc..........................................   2        0     seq
    ..............guuucuaggcgcauuauuuu...........................................   1        0     seq
    ................uucuaggcgcauuauuuuc..........................................   1        0     seq
    ...................................uauuguuuaugaaaaugc........................   1        0     seq
    .....................................uuguuuaugaaaaugccccagaaaucc.............   1        0     seq
    ..............................................aaaaugccccagaaauccu............   2        0     seq
    ..............................................aaaaugccccagaaauccua...........   10       0     seq
    ..............................................aaaaugccccagaaauccuaa..........   24       0     seq
    ..............................................aaaaugccccagaaauccuaaa.........   9        0     seq
```

m0054_3p

m0054_5p

uaugcaguuuguggguuucuaggcgcauuauuuucuauuguuuaugaaaaugcccccagaaauccuaaaaauugugua

....aaaugcccccagaaaucc.............	221	0	seq
....aaaugcccccagaaauccu............	950	0	seq
....aaaugcccccagaaauccua...........	4534	0	seq
....aaaugcccccagaaauccuaa..........	12834	0	seq
....aaaugcccccagaaauccuaaa.........	5779	0	seq
....aaaugcccccagaaauccuaaaa........	2388	0	seq
....aaaugcccccagaaauccuaaaaa.......	62	0	seq
....aaaugcccccagaaauccuaaaaau......	1	0	seq
....aaugcccccagaaauccu............	1	0	seq
....aaugcccccagaaauccua...........	39	0	seq
....aaugcccccagaaauccuaa..........	110	0	seq
....aaugcccccagaaauccuaaa.........	61	0	seq
....aaugcccccagaaauccuaaaa........	26	0	seq
....aaugcccccagaaauccuaaaaa.......	1	0	seq
....augcccccagaaauccua...........	1	0	seq
....augcccccagaaauccuaa..........	5	0	seq
....augcccccagaaauccuaaa.........	2	0	seq
....augcccccagaaauccuaaaa........	2	0	seq
