# Supplementary material for: Identification of Conserved and Novel MicroRNAs in the Pacific Oyster Crassostrea gigas by Deep Sequencing
Source: PLoS One. 2014 Aug 19;9(8):e104371. doi: 10.1371/journal.pone.0104371 (PMC4138081; doi:10.1371/journal.pone.0104371)
Supplement: File S2 — The compressed/ZIP file archive for the predicted precursors' secondary structures and reads alignment. (ZIP) [file pone.0104371.s010.zip › second structure and reads alignment for oyster miRNAs/potential in table S7/m0113.pdf]

miRBase precursor : m0113
Total read count : 596
m0113_5p read count : 589
m0113_3p read count : 3
remaining reads : 4

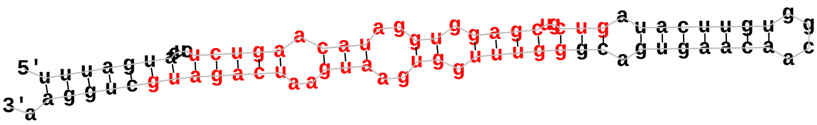

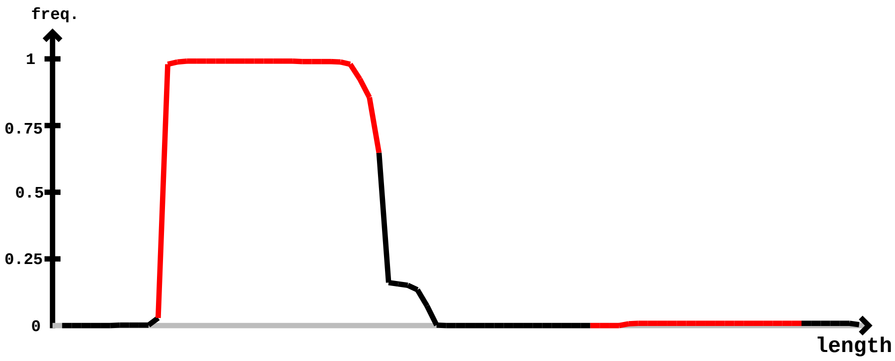


```
           m0113_5p
                                                m0113_3p
5'- uuuaguacucucugaacauagguggagcugcugauacuuguggcaacaagugacgguuuggugaaugaaucagaugcuggaa   -3'    exp
    .((((((((((((.......)).)))).))))))(((((((....))))))).((((((((((......))))))).)))...    reads      mm         sample
    .....uacucucugaacauaggug...........................................................    1          0          seq
    .........cucugaacauagguggagcugcug..................................................    1          0          seq
    .........cucugaacauagguggagcugcugaua...............................................    1          0          seq
    .........cucugaacauagguggagcugcugauac..............................................    5          0          seq
    .........cucugaacauagguggagcugcugauacu.............................................    9          0          seq
    ..........ucugaacauagguggagc.......................................................    1          0          seq
    ..........ucugaacauagguggagcu......................................................    5          0          seq
    ..........ucugaacauagguggagcug.....................................................    32         0          seq
    ..........ucugaacauagguggagcugc....................................................    40         0          seq
    ..........ucugaacauagguggagcugcu...................................................    120        0          seq
    ..........ucugaacauagguggagcugcug..................................................    288        0          seq
    ..........ucugaacauagguggagcugcuga.................................................    3          0          seq
    ..........ucugaacauagguggagcugcugau................................................    3          0          seq
    ..........ucugaacauagguggagcugcugaua...............................................    9          0          seq
    ..........ucugaacauagguggagcugcugauac..............................................    31         0          seq
    ..........ucugaacauagguggagcugcugauacu.............................................    34         0          seq
    ..........ucugaacauagguggagcugcugauacuu............................................    1          0          seq
    ...........cugaacauagguggagcugc....................................................    1          0          seq
    ...........cugaacauagguggagcugcu...................................................    3          0          seq
    ...........cugaacauagguggagcugcug..................................................    1          0          seq
    ............ugaacauagguggagcug.....................................................    1          0          seq
    ............ugaacauagguggagcugcu...................................................    1          0          seq
    ..........................................................uuggugaaugaaucagaugcugga.    3          0          seq
    ..........................................................uuggugaaugaaucagaugcuggaa    1          0          seq
    ...........................................................uggugaaugaaucagaugcuggaa    1          0          seq
```
