# Supplementary material for: Identification of Conserved and Novel MicroRNAs in the Pacific Oyster Crassostrea gigas by Deep Sequencing
Source: PLoS One. 2014 Aug 19;9(8):e104371. doi: 10.1371/journal.pone.0104371 (PMC4138081; doi:10.1371/journal.pone.0104371)
Supplement: File S2 — The compressed/ZIP file archive for the predicted precursors' secondary structures and reads alignment. (ZIP) [file pone.0104371.s010.zip › second structure and reads alignment for oyster miRNAs/potential in table S7/m0137.pdf]

miRBase precursor : m0137
Total read count : 99671
m0137_5p read count : 39317
m0137_3p read count : 190
remaining reads : 60164

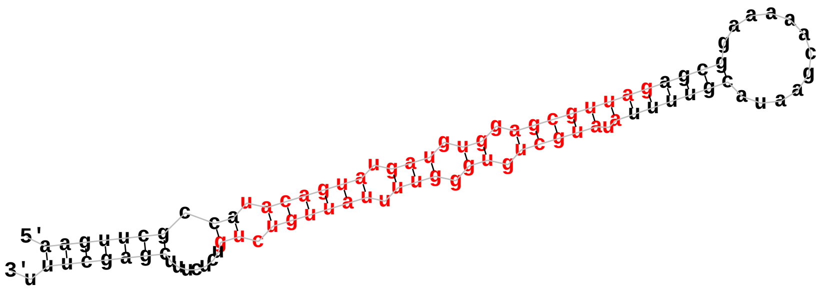

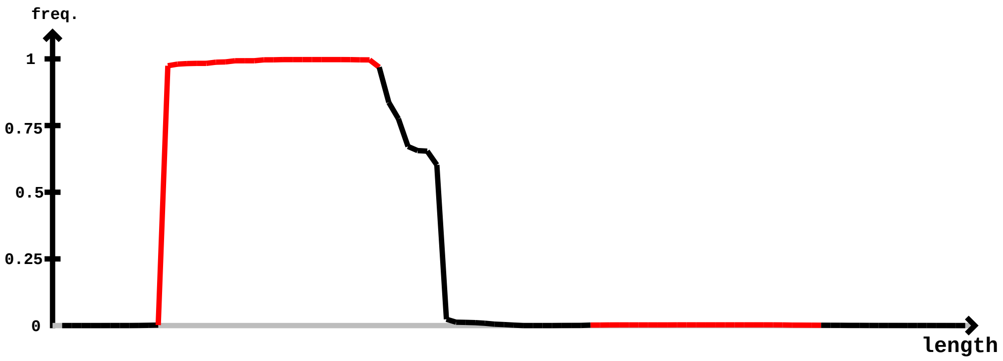

m0137_3p

m0137_5p

```
5'- aaguucgccauacaguaugauguggagcguuagagcggaaaaacgaauacguuuuauaugcuguggguuuuauugucugucucuuucgagcuuu   -3'    exp
    (((((((.((.((((((.(((.((.((((((((((((............))))))).))))).)).))).)))))).)).......))))))).   reads   mm   sample
    ....ucgccauacaguaugauguggagcg.................................................................   1       0    seq
    ....ucgccauacaguaugauguggagcgu................................................................   1       0    seq
    ....ucgccauacaguaugauguggagcguua..............................................................   6       0    seq
    ....ucgccauacaguaugauguggagcguuag.............................................................   1       0    seq
    .......ccauacaguaugauguggagcguua..............................................................   2       0    seq
    .......ccauacaguaugauguggagcguuaga............................................................   4       0    seq
    .......ccauacaguaugauguggagcguuagag...........................................................   12      0    seq
    .......ccauacaguaugauguggagcguuagagc..........................................................   13      0    seq
    ........cauacaguaugauguggagcguua..............................................................   6       0    seq
    ........cauacaguaugauguggagcguuag.............................................................   21      0    seq
    ........cauacaguaugauguggagcguuaga............................................................   10      0    seq
    ........cauacaguaugauguggagcguuagag...........................................................   9       0    seq
    ........cauacaguaugauguggagcguuagagc..........................................................   2       0    seq
    ........cauacaguaugauguggagcguuagagcgg........................................................   15      0    seq
    .........auacaguaugauguggagcguua..............................................................   16      0    seq
    .........auacaguaugauguggagcguuag.............................................................   3       0    seq
    .........auacaguaugauguggagcguuaga............................................................   1       0    seq
    .........auacaguaugauguggagcguuagag...........................................................   6       0    seq
    .........auacaguaugauguggagcguuagagc..........................................................   3       0    seq
    .........auacaguaugauguggagcguuagagcgg........................................................   47      0    seq
    .........auacaguaugauguggagcguuagagcgga.......................................................   1       0    seq
    ..........uacaguaugauguggagcg.................................................................   28      0    seq
    ..........uacaguaugauguggagcgu................................................................   68      0    seq
    ..........uacaguaugauguggagcguu...............................................................   21      0    seq
    ..........uacaguaugauguggagcguua..............................................................   2692    0    seq
    ..........uacaguaugauguggagcguuag.............................................................   13134   0    seq
    ..........uacaguaugauguggagcguuaga............................................................   6062    0    seq
    ..........uacaguaugauguggagcguuagag...........................................................   10297   0    seq
    ..........uacaguaugauguggagcguuagagc..........................................................   1511    0    seq
    ..........uacaguaugauguggagcguuagagcg.........................................................   211     0    seq
    ..........uacaguaugauguggagcguuagagcgg........................................................   4999    0    seq
    ..........uacaguaugauguggagcguuagagcgga.......................................................   57042   0    seq
    ..........uacaguaugauguggagcguuagagcggaa......................................................   852     0    seq
    ..........uacaguaugauguggagcguuagagcggaaa.....................................................   1       0    seq
```

```
                                                       m0137_3p
          m0137_5p
aaguucgccauacaguaugauguggagcguuagagcggaaaaacgaauacguuuuauaugcugugguuuuauugucugucucuuucgagcuuu

...........acaguaugauguggagcg................................................................    1      0      seq
...........acaguaugauguggagcguua.............................................................    3      0      seq
...........acaguaugauguggagcguuag............................................................    2      0      seq
...........acaguaugauguggagcguuaga...........................................................    8      0      seq
...........acaguaugauguggagcguuagag..........................................................    12     0      seq
...........acaguaugauguggagcguuagagc.........................................................    3      0      seq
...........acaguaugauguggagcguuagagcgg.......................................................    37     0      seq
...........acaguaugauguggagcguuagagcgga......................................................    416    0      seq
...........acaguaugauguggagcguuagagcggaa.....................................................    124    0      seq
............caguaugauguggagcguua.............................................................    1      0      seq
............caguaugauguggagcguuag............................................................    5      0      seq
............caguaugauguggagcguuaga...........................................................    13     0      seq
............caguaugauguggagcguuagag..........................................................    9      0      seq
............caguaugauguggagcguuagagc.........................................................    7      0      seq
............caguaugauguggagcguuagagcgg.......................................................    17     0      seq
............caguaugauguggagcguuagagcgga......................................................    109    0      seq
............caguaugauguggagcguuagagcggaa.....................................................    45     0      seq
............caguaugauguggagcguuagagcggaaa....................................................    11     0      seq
.............aguaugauguggagcguuagagcgg.......................................................    2      0      seq
.............aguaugauguggagcguuagagcgga......................................................    34     0      seq
.............aguaugauguggagcguuagagcggaa.....................................................    9      0      seq
.............aguaugauguggagcguuagagcggaaa....................................................    11     0      seq
.............aguaugauguggagcguuagagcggaaaa...................................................    15     0      seq
.............aguaugauguggagcguuagagcggaaaaa..................................................    1      0      seq
..............guaugauguggagcguuagagcggaaa....................................................    3      0      seq
..............guaugauguggagcguuagagcggaaaa...................................................    1      0      seq
..............guaugauguggagcguuagagcggaaaaac.................................................    2      0      seq
...............uaugauguggagcguuag............................................................    2      0      seq
...............uaugauguggagcguuaga...........................................................    3      0      seq
...............uaugauguggagcguuagag..........................................................    5      0      seq
...............uaugauguggagcguuagagc.........................................................    5      0      seq
...............uaugauguggagcguuagagcgg.......................................................    18     0      seq
...............uaugauguggagcguuagagcgga......................................................    66     0      seq
...............uaugauguggagcguuagagcggaa.....................................................    39     0      seq
...............uaugauguggagcguuagagcggaaa....................................................    34     0      seq
...............uaugauguggagcguuagagcggaaaa...................................................    62     0      seq
...............uaugauguggagcguuagagcggaaaaa..................................................    105    0      seq
...............uaugauguggagcguuagagcggaaaaac.................................................    85     0      seq
...............uaugauguggagcguuagagcggaaaaacg................................................    12     0      seq
................augauguggagcguuaga...........................................................    1      0      seq
................augauguggagcguuagagcgga......................................................    8      0      seq
................augauguggagcguuagagcggaa.....................................................    2      0      seq
................augauguggagcguuagagcggaaa....................................................    5      0      seq
................augauguggagcguuagagcggaaaa...................................................    3      0      seq
................augauguggagcguuagagcggaaaaa..................................................    26     0      seq
................augauguggagcguuagagcggaaaaac.................................................    53     0      seq
................augauguggagcguuagagcggaaaaacg................................................    2      0      seq
................augauguggagcguuagagcggaaaaacga...............................................    1      0      seq
.................ugauguggagcguuagagcgg.......................................................    1      0      seq
.................ugauguggagcguuagagcgga......................................................    10     0      seq
.................ugauguggagcguuagagcggaa.....................................................    15     0      seq
.................ugauguggagcguuagagcggaaa....................................................    8      0      seq
.................ugauguggagcguuagagcggaaaa...................................................    12     0      seq
.................ugauguggagcguuagagcggaaaaa..................................................    85     0      seq
.................ugauguggagcguuagagcggaaaaac.................................................    181    0      seq
.................ugauguggagcguuagagcggaaaaacg................................................    76     0      seq
.................ugauguggagcguuagagcggaaaaacga...............................................    38     0      seq
..................gauguggagcguuagagcgga......................................................    1      0      seq
..................gauguggagcguuagagcggaaaaacg................................................    2      0      seq
..................gauguggagcguuagagcggaaaaacga...............................................    4      0      seq
..................gauguggagcguuagagcggaaaaacgaa..............................................    1      0      seq
...................auguggagcguuagagcggaaaaac.................................................    1      0      seq
...................auguggagcguuagagcggaaaaacga...............................................    2      0      seq
...................auguggagcguuagagcggaaaaacgaa..............................................    3      0      seq
....................uguggagcguuagagcgga......................................................    2      0      seq
....................uguggagcguuagagcggaa.....................................................    7      0      seq
....................uguggagcguuagagcggaaa....................................................    4      0      seq
....................uguggagcguuagagcggaaaa...................................................    5      0      seq
....................uguggagcguuagagcggaaaaa..................................................    2      0      seq
```

```
                                                       m0137_3p
          m0137_5p
aaguucgccauacaguaugauguggagcguuagagcggaaaaacgaauacguuuuauaugcugugguuuuauugucugucucuuucgagcuuu

....................uguggagcguuagagcggaaaaac.................................................    5      0      seq
....................uguggagcguuagagcggaaaaacg................................................    64     0      seq
....................uguggagcguuagagcggaaaaacga...............................................    123    0      seq
....................uguggagcguuagagcggaaaaacgaa..............................................    126    0      seq
....................uguggagcguuagagcggaaaaacgaau.............................................    1      0      seq
.....................guggagcguuagagcgga......................................................    2      0      seq
.....................guggagcguuagagcggaaaaacg................................................    3      0      seq
.....................guggagcguuagagcggaaaaacgaa..............................................    3      0      seq
......................uggagcguuagagcggaa.....................................................    1      0      seq
......................uggagcguuagagcggaaa....................................................    1      0      seq
......................uggagcguuagagcggaaaa...................................................    1      0      seq
......................uggagcguuagagcggaaaaa..................................................    4      0      seq
......................uggagcguuagagcggaaaaac.................................................    10     0      seq
......................uggagcguuagagcggaaaaacg................................................    5      0      seq
......................uggagcguuagagcggaaaaacga...............................................    25     0      seq
......................uggagcguuagagcggaaaaacgaa..............................................    32     0      seq
.......................ggagcguuagagcggaaaaacg................................................    1      0      seq
.............................uuagagcggaaaaacgaa..............................................    5      0      seq
...................................................uuuuauaugcugugguuuuau.....................    2      0      seq
...................................................uuuuauaugcugugguuuuauu....................    3      0      seq
...................................................uuuuauaugcugugguuuuauug...................    18     0      seq
...................................................uuuuauaugcugugguuuuauugu..................    2      0      seq
...................................................uuuuauaugcugugguuuuauuguc.................    2      0      seq
...................................................uuuuauaugcugugguuuuauugucu................    1      0      seq
...................................................uuuuauaugcugugguuuuauugucug...............    2      0      seq
....................................................uuuauaugcugugguuuuau.....................    1      0      seq
....................................................uuuauaugcugugguuuuauu....................    1      0      seq
....................................................uuuauaugcugugguuuuauug...................    6      0      seq
....................................................uuuauaugcugugguuuuauugucu................    1      0      seq
....................................................uuuauaugcugugguuuuauugucug...............    1      0      seq
.....................................................uuauaugcugugguuuuauu....................    1      0      seq
.....................................................uuauaugcugugguuuuauug...................    3      0      seq
.....................................................uuauaugcugugguuuuauugucu................    1      0      seq
.....................................................uuauaugcugugguuuuauugucugu..............    1      0      seq
......................................................uauaugcugugguuuua......................    2      0      seq
......................................................uauaugcugugguuuuau.....................    4      0      seq
......................................................uauaugcugugguuuuauu....................    16     0      seq
......................................................uauaugcugugguuuuauug...................    23     0      seq
......................................................uauaugcugugguuuuauugu..................    10     0      seq
......................................................uauaugcugugguuuuauuguc.................    7      0      seq
......................................................uauaugcugugguuuuauugucu................    12     0      seq
......................................................uauaugcugugguuuuauugucug...............    12     0      seq
......................................................uauaugcugugguuuuauugucugu..............    9      0      seq
......................................................uauaugcugugguuuuauugucuguc.............    28     0      seq
......................................................uauaugcugugguuuuauugucugucu............    2      0      seq
......................................................uauaugcugugguuuuauugucugucuc...........    9      0      seq
.......................................................auaugcugugguuuuauug...................    1      0      seq
.......................................................auaugcugugguuuuauugucu................    1      0      seq
.......................................................auaugcugugguuuuauugucugucuc...........    2      0      seq
.......................................................auaugcugugguuuuauugucugucucu..........    1      0      seq
........................................................uaugcugugguuuuauu....................    1      0      seq
........................................................uaugcugugguuuuauug...................    7      0      seq
........................................................uaugcugugguuuuauugu..................    3      0      seq
........................................................uaugcugugguuuuauuguc.................    7      0      seq
........................................................uaugcugugguuuuauugucu................    6      0      seq
........................................................uaugcugugguuuuauugucug...............    1      0      seq
........................................................uaugcugugguuuuauugucugu..............    1      0      seq
........................................................uaugcugugguuuuauugucuguc.............    6      0      seq
........................................................uaugcugugguuuuauugucugucu............    2      0      seq
........................................................uaugcugugguuuuauugucugucuc...........    4      0      seq
........................................................uaugcugugguuuuauugucugucucu..........    5      0      seq
........................................................uaugcugugguuuuauugucugucucuu.........    4      0      seq
.........................................................augcugugguuuuauuguc.................    1      0      seq
.........................................................augcugugguuuuauugucugucucuu.........    2      0      seq
..........................................................ugcugugguuuuauugucugucucuu.........    1      0      seq
..........................................................ugcugugguuuuauugucugucucuuuc.......    1      0      seq
.............................................................uguggguuuuauugucugucucuuucgagc...    1      0      seq
...............................................................uggguuuuauugucugucuc...........    1      0      seq
...............................................................uggguuuuauugucugucucuuucga.....    3      0      seq
```

```
                                                               m0137_3p
          m0137_5p
aaguucgccauacaguaugauguggagcguuagagcggaaaaacgaauacguuuuauaugcuguggguuuuauugucugucucuuucgagcuuu

...............................................................uggguuuuauugucugucucuuucgag....    9    0    seq
...............................................................uggguuuuauugucugucucuuucgagc...    1    0    seq
...............................................................uggguuuuauugucugucucuuucgagcu..    3    0    seq
...............................................................uggguuuuauugucugucucuuucgagcuu.    2    0    seq
...................................................................uuuuauugucugucucuu.........    1    0    seq
...................................................................uuuuauugucugucucuuucgag....    1    0    seq
......................................................................uauugucugucucuuucga.....    1    0    seq
......................................................................uauugucugucucuuucgag....    2    0    seq
......................................................................uauugucugucucuuucgagcu..    1    0    seq
```
