# Supplementary material for: Identification of Conserved and Novel MicroRNAs in the Pacific Oyster Crassostrea gigas by Deep Sequencing
Source: PLoS One. 2014 Aug 19;9(8):e104371. doi: 10.1371/journal.pone.0104371 (PMC4138081; doi:10.1371/journal.pone.0104371)
Supplement: File S2 — The compressed/ZIP file archive for the predicted precursors' secondary structures and reads alignment. (ZIP) [file pone.0104371.s010.zip › second structure and reads alignment for oyster miRNAs/potential in table S7/m0157.pdf]

miRBase precursor : m0157
Total read count : 2839
m0157_5p read count : 3
m0157_3p read count : 2825
remaining reads : 11

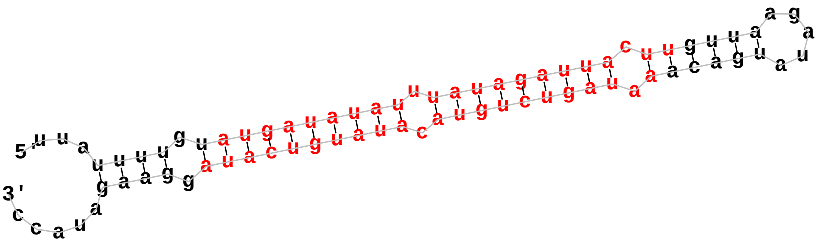

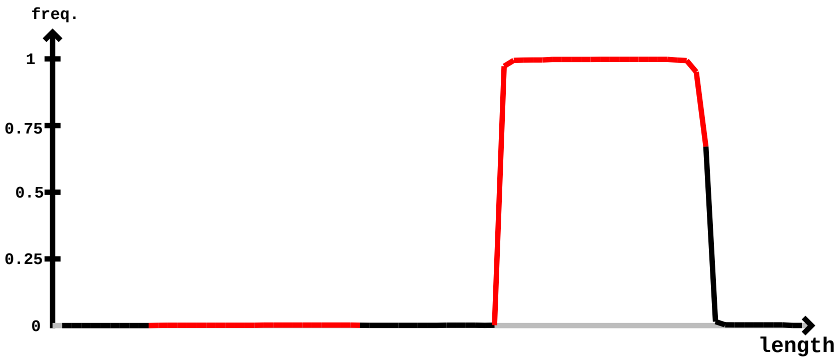


```
         m0157_5p
                                             m0157_3p
5'- uuauuuuguaugauauauuuauagauuacuuguuaagauaugacaaauagucuguacauaugucauaggaagauacc   -3'    exp
    ...((((.((((((((((.(((((((((.((((((.....)))))).))))))))).)))))))))).)))).....    reads      mm         sample
    .........augauauauuuauagauuacu...............................................    2          0          seq
    ..........ugauauauuuauagauuacuu..............................................    1          0          seq
    ....................auagauuacuuguuaagauauga..................................    2          0          seq
    .......................................augacaaauagucuguacauauguc.............    2          0          seq
    ............................................aaauagucuguacauaugucau...........    1          0          seq
    ............................................aaauagucuguacauaugucauag.........    1          0          seq
    .............................................aauagucuguacauaugu..............    8          0          seq
    .............................................aauagucuguacauauguc.............    3          0          seq
    .............................................aauagucuguacauauguca............    121        0          seq
    .............................................aauagucuguacauaugucau...........    787        0          seq
    .............................................aauagucuguacauaugucaua..........    1834       0          seq
    .............................................aauagucuguacauaugucauag.........    4          0          seq
    ..............................................auagucuguacauaugucau...........    7          0          seq
    ..............................................auagucuguacauaugucaua..........    26         0          seq
    ..............................................auagucuguacauaugucauag.........    28         0          seq
    ..............................................auagucuguacauaugucauagg........    1          0          seq
    ...............................................uagucuguacauaugucaua..........    3          0          seq
    ................................................agucuguacauaugucauag.........    1          0          seq
    ..................................................ucuguacauaugucauaggaagaua..    6          0          seq
    .......................................................acauaugucauaggaagauacc    1          0          seq
```
