# Supplementary material for: Identification of Conserved and Novel MicroRNAs in the Pacific Oyster Crassostrea gigas by Deep Sequencing
Source: PLoS One. 2014 Aug 19;9(8):e104371. doi: 10.1371/journal.pone.0104371 (PMC4138081; doi:10.1371/journal.pone.0104371)
Supplement: File S2 — The compressed/ZIP file archive for the predicted precursors' secondary structures and reads alignment. (ZIP) [file pone.0104371.s010.zip › second structure and reads alignment for oyster miRNAs/potential in table S7/m0173.pdf]

miRBase precursor : m0173
Total read count : 53
m0173_5p read count : 37
m0173_3p read count : 14
remaining reads : 2

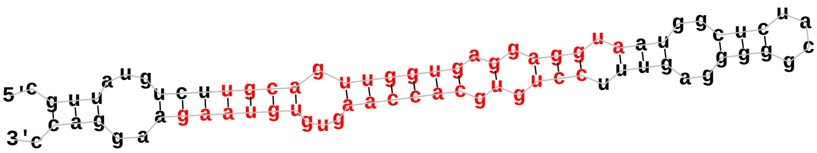

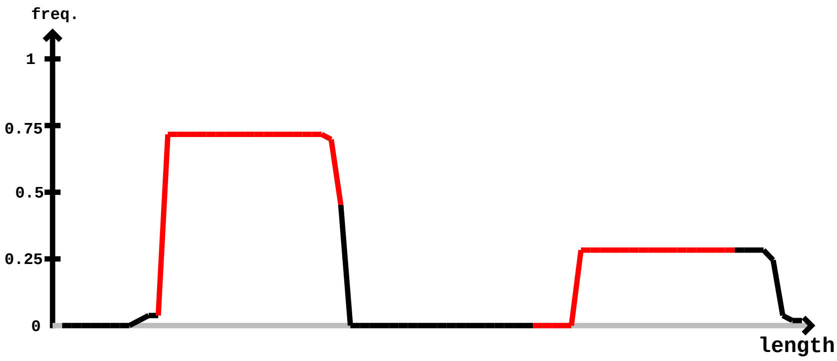


```
                                                 m0173_3p
           m0173_5p
5'- cguuaugucuugcaguuggugaggagguaauggcucuacgggggaguuuccugugcaccaagugugua aagaaggacc   -3'    exp
    ......((((((((.((((((.(.(((.(((..(((....)))..))).))).).)))))).)))......)))))).   reads      mm         sample
    .......ucuugcaguuggugaggaggua................................................   1          0          seq
    ........cuugcaguuggugaggagg..................................................   1          0          seq
    ..........ugcaguuggugaggaggu.................................................   13         0          seq
    ..........ugcaguuggugaggaggua................................................   23         0          seq
    .....................................................ugcaccaagugugua agaag....   2          0          seq
    .....................................................ugcaccaagugugua agaagg...   11         0          seq
    .....................................................ugcaccaagugugua agaagga..   1          0          seq
    .....................................................ugcaccaagugugua agaaggacc   1          0          seq
```
