# Supplementary material for: Identification of Conserved and Novel MicroRNAs in the Pacific Oyster Crassostrea gigas by Deep Sequencing
Source: PLoS One. 2014 Aug 19;9(8):e104371. doi: 10.1371/journal.pone.0104371 (PMC4138081; doi:10.1371/journal.pone.0104371)
Supplement: File S2 — The compressed/ZIP file archive for the predicted precursors' secondary structures and reads alignment. (ZIP) [file pone.0104371.s010.zip › second structure and reads alignment for oyster miRNAs/potential in table S7/m0212.pdf]

miRBase precursor : m0212
Total read count : 2580
m0212_5p read count : 3
m0212_3p read count : 1707
remaining reads : 870

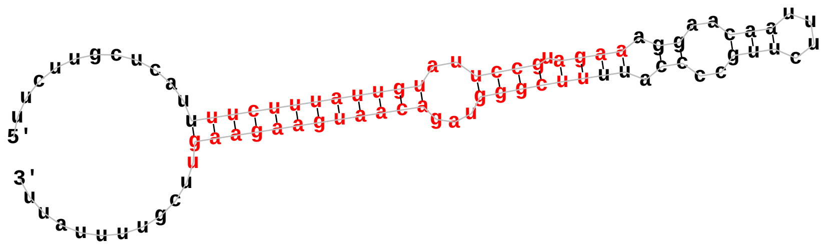

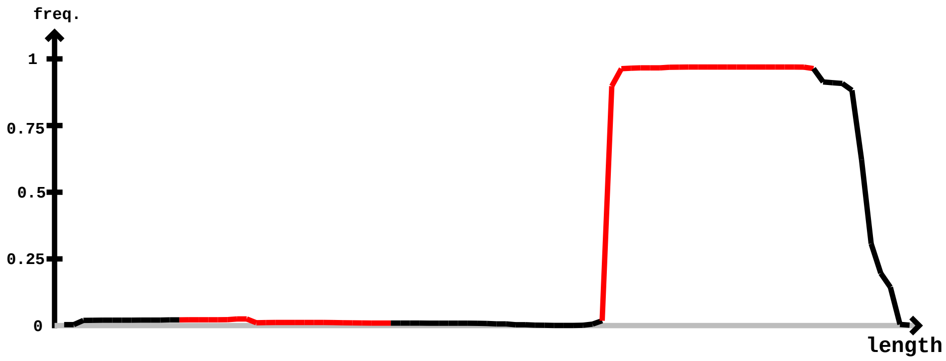

m0212_5p

m0212_3p

```
5'- uucuugcucauuuucuuuauuguauuccguagaaaggaacaauuucuugccccauuuucggguagacaaugaagaaguucguuuuauu   -3'    exp
    ...........((((((((((((..((((.((((.((..(((....)))..)).))))))))...))))))))))))...........    reads    mm    sample
    uucuugcucauuuucuuua.....................................................................    8        0     seq
    uucuugcucauuuucuuuauuguauuccg...........................................................    1        0     seq
    .ucuugcucauuuucuuua.....................................................................    39       0     seq
    .ucuugcucauuuucuuuauuguau...............................................................    1        0     seq
    .ucuugcucauuuucuuuauuguauuccgu..........................................................    1        0     seq
    ..cuugcucauuuucuuuauuguauuccgua.........................................................    1        0     seq
    ...uugcucauuuucuuuauuguauuc.............................................................    1        0     seq
    .......ucauuuucuuuauuguau...............................................................    1        0     seq
    ..........uuuucuuuauuguauucc............................................................    2        0     seq
    ............uucuuuauuguauuccguagaaagg...................................................    1        0     seq
    ................uuauuguauuccguagaaaggaacaau.............................................    1        0     seq
    .................uauuguauuccguagaaaggaacaa..............................................    1        0     seq
    .................uauuguauuccguagaaaggaacaau.............................................    1        0     seq
    .................uauuguauuccguagaaaggaacaauu............................................    2        0     seq
    .................uauuguauuccguagaaaggaacaauuuc..........................................    3        0     seq
    ...................uuguauuccguagaaaggaacaauu............................................    1        0     seq
    ...................uuguauuccguagaaaggaacaauuuc..........................................    5        0     seq
    ...................uuguauuccguagaaaggaacaauuucuu........................................    3        0     seq
    ...................uuguauuccguagaaaggaacaauuucuug.......................................    1        0     seq
    ....................uguauuccguagaaaggaacaauuucuu........................................    1        0     seq
    ....................uguauuccguagaaaggaacaauuucuugc......................................    1        0     seq
    .....................guauuccguagaaaggaacaauu............................................    1        0     seq
    .........................uccguagaaaggaacaauuucuugc......................................    1        0     seq
    .........................uccguagaaaggaacaauuucuugccccau.................................    1        0     seq
    .....................................................auuuucggguagacaaugaagaaguucgu......    2        0     seq
    ......................................................uuuucggguagacaaugaagaagu..........    2        0     seq
    ......................................................uuuucggguagacaaugaagaaguucg.......    1        0     seq
    ......................................................uuuucggguagacaaugaagaaguucgu......    3        0     seq
    ......................................................uuuucggguagacaaugaagaaguucguu.....    5        0     seq
    .......................................................uuucggguagacaaugaagaag...........    1        0     seq
    .......................................................uuucggguagacaaugaagaagu..........    1        0     seq
    .......................................................uuucggguagacaaugaagaaguuc........    1        0     seq
    .......................................................uuucggguagacaaugaagaaguucg.......    3        0     seq
    .......................................................uuucggguagacaaugaagaaguucgu......    6        0     seq
```

```
            m0212_5p
                                                        m0212_3p
uucuugcucauuuucuuuauuguauuccguagaaaggaacaauuucuugccccauuuucggguagacaaugaagaaguucguuuuauu

.......................................................uuucggguagacaaugaagaaguucguu.....    19    0    seq
.......................................................uuucggguagacaaugaagaaguucguuu....    3     0    seq
........................................................uucggguagacaaugaagaa............    1     0    seq
........................................................uucggguagacaaugaagaag...........    13    0    seq
........................................................uucggguagacaaugaagaagu..........    120   0    seq
........................................................uucggguagacaaugaagaaguu.........    8     0    seq
........................................................uucggguagacaaugaagaaguuc........    5     0    seq
........................................................uucggguagacaaugaagaaguucg.......    59    0    seq
........................................................uucggguagacaaugaagaaguucgu......    640   0    seq
........................................................uucggguagacaaugaagaaguucguu.....    765   0    seq
........................................................uucggguagacaaugaagaaguucguuu....    278   0    seq
........................................................uucggguagacaaugaagaaguucguuuu...    124   0    seq
........................................................uucggguagacaaugaagaaguucguuuua..    256   0    seq
.........................................................ucggguagacaaugaagaagu..........    6     0    seq
.........................................................ucggguagacaaugaagaaguuc........    1     0    seq
.........................................................ucggguagacaaugaagaaguucg.......    3     0    seq
.........................................................ucggguagacaaugaagaaguucgu......    20    0    seq
.........................................................ucggguagacaaugaagaaguucguu.....    21    0    seq
.........................................................ucggguagacaaugaagaaguucguuu....    4     0    seq
.........................................................ucggguagacaaugaagaaguucguuuu...    9     0    seq
.........................................................ucggguagacaaugaagaaguucguuuua..    100   0    seq
.........................................................ucggguagacaaugaagaaguucguuuuau.    5     0    seq
..........................................................cggguagacaaugaagaaguucgu......    1     0    seq
..........................................................cggguagacaaugaagaaguucguuuu...    1     0    seq
..........................................................cggguagacaaugaagaaguucguuuua..    1     0    seq
..........................................................cggguagacaaugaagaaguucguuuuau.    1     0    seq
..........................................................cggguagacaaugaagaaguucguuuuauu    1     0    seq
...........................................................ggguagacaaugaagaaguucguu.....    2     0    seq
...........................................................ggguagacaaugaagaaguucguuu....    1     0    seq
..............................................................uagacaaugaagaaguucguuuu...    1     0    seq
..............................................................uagacaaugaagaaguucguuuua..    2     0    seq
..............................................................uagacaaugaagaaguucguuuuauu    3     0    seq
...............................................................agacaaugaagaaguucguuuuau.    1     0    seq
................................................................gacaaugaagaaguucguuuua..    1     0    seq
```
