# Supplementary material for: Identification of Conserved and Novel MicroRNAs in the Pacific Oyster Crassostrea gigas by Deep Sequencing
Source: PLoS One. 2014 Aug 19;9(8):e104371. doi: 10.1371/journal.pone.0104371 (PMC4138081; doi:10.1371/journal.pone.0104371)
Supplement: File S2 — The compressed/ZIP file archive for the predicted precursors' secondary structures and reads alignment. (ZIP) [file pone.0104371.s010.zip › second structure and reads alignment for oyster miRNAs/potential in table S7/m0215.pdf]

miRBase precursor : m0215
Total read count : 679
m0215_5p read count : 477
m0215_3p read count : 202
remaining reads : 0

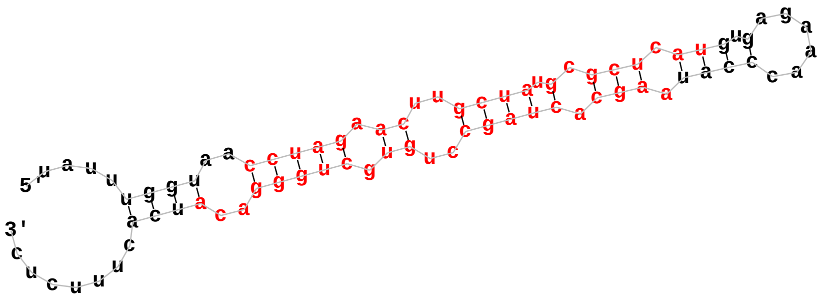

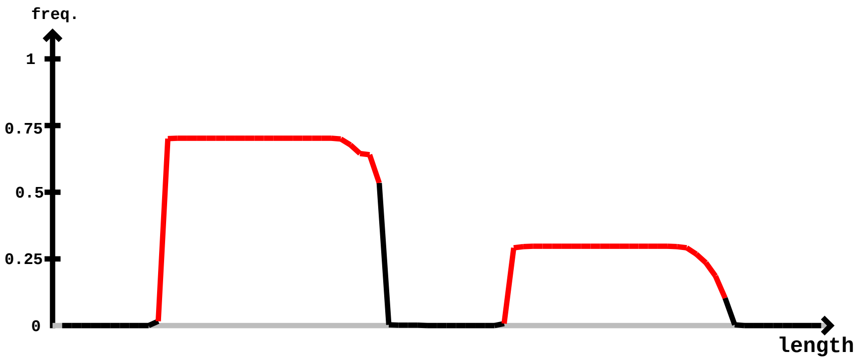


```
                  m0215_5p
                                               m0215_3p
5'- uauuugguaaccuagaacuugcuaugcgcucaugugagaaacccauaagcacuagccugugcugggacaucacuuucuc   -3'    exp
    ....((((..(((((.((..((((.(.(((.(((.(......)))).))).))))).)).))))).))))).......    reads      mm         sample
    .........accuagaacuugcuaugcgc..................................................    1          0          seq
    .........accuagaacuugcuaugcgcu.................................................    2          0          seq
    .........accuagaacuugcuaugcgcuca...............................................    7          0          seq
    .........accuagaacuugcuaugcgcucau..............................................    1          0          seq
    ..........ccuagaacuugcuaugcg...................................................    2          0          seq
    ..........ccuagaacuugcuaugcgc..................................................    14         0          seq
    ..........ccuagaacuugcuaugcgcu.................................................    20         0          seq
    ..........ccuagaacuugcuaugcgcuc................................................    3          0          seq
    ..........ccuagaacuugcuaugcgcuca...............................................    64         0          seq
    ..........ccuagaacuugcuaugcgcucau..............................................    360        0          seq
    ..........ccuagaacuugcuaugcgcucaug.............................................    1          0          seq
    ..........ccuagaacuugcuaugcgcucauguga..........................................    1          0          seq
    ...........cuagaacuugcuaugcgcuca...............................................    1          0          seq
    .............................................uaagcacuagccugugcu................    1          0          seq
    .............................................uaagcacuagccugugcugg..............    2          0          seq
    .............................................uaagcacuagccugugcuggga............    2          0          seq
    ..............................................aagcacuagccugugcug...............    3          0          seq
    ..............................................aagcacuagccugugcugg..............    14         0          seq
    ..............................................aagcacuagccugugcuggg.............    21         0          seq
    ..............................................aagcacuagccugugcuggga............    29         0          seq
    ..............................................aagcacuagccugugcugggac...........    56         0          seq
    ..............................................aagcacuagccugugcugggaca..........    68         0          seq
    ..............................................aagcacuagccugugcugggacau.........    2          0          seq
    ...............................................agcacuagccugugcuggg.............    1          0          seq
    ...............................................agcacuagccugugcuggga............    2          0          seq
    ................................................gcacuagccugugcuggga............    1          0          seq
```
