# Supplementary material for: Identification of Conserved and Novel MicroRNAs in the Pacific Oyster Crassostrea gigas by Deep Sequencing
Source: PLoS One. 2014 Aug 19;9(8):e104371. doi: 10.1371/journal.pone.0104371 (PMC4138081; doi:10.1371/journal.pone.0104371)
Supplement: File S2 — The compressed/ZIP file archive for the predicted precursors' secondary structures and reads alignment. (ZIP) [file pone.0104371.s010.zip › second structure and reads alignment for oyster miRNAs/potential in table S7/m0269.pdf]

miRBase precursor	: m0269
Total read count	: 89
m0269_5p read count	: 84
m0269_3p read count	: 2
remaining reads	: 3

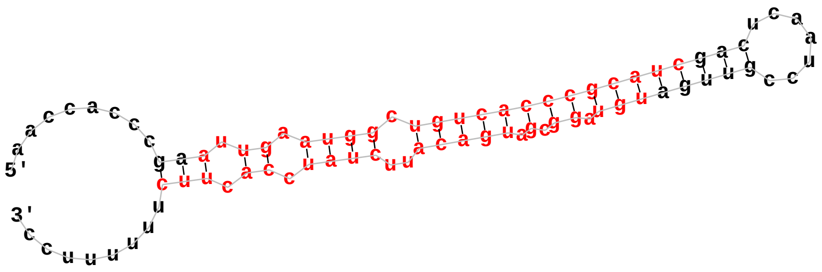

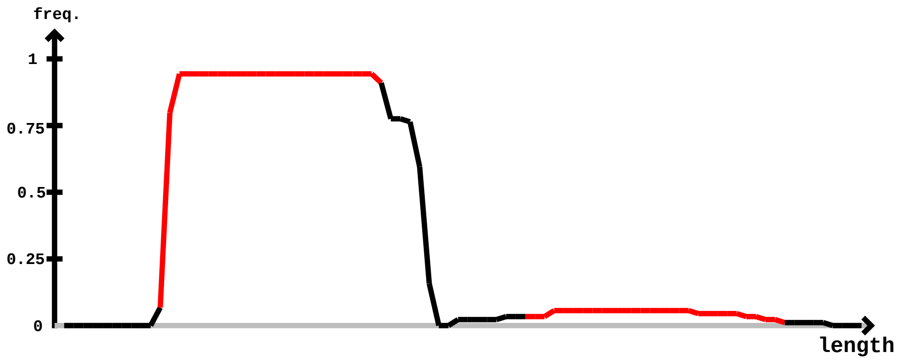


```
                 m0269_5p
                                                  m0269_3p
5'- aaccacccgaauugaauggcugucacccgcaucgacucaauccguugauguaggcgaugacauucuauccacuucuuuuuucc   -3'    exp
    ........(((.((.((((.((((((((((((((((.......)))))))).)).).)))))..)))).)).)))........    reads      mm         sample
    .........aauugaauggcugucacccgcaucgacuc.............................................    6          0          seq
    ..........auugaauggcugucacccgcau...................................................    3          0          seq
    ..........auugaauggcugucacccgcauc..................................................    12         0          seq
    ..........auugaauggcugucacccgcaucga................................................    1          0          seq
    ..........auugaauggcugucacccgcaucgac...............................................    14         0          seq
    ..........auugaauggcugucacccgcaucgacu..............................................    31         0          seq
    ..........auugaauggcugucacccgcaucgacuc.............................................    4          0          seq
    ...........uugaauggcugucacccgcaucgac...............................................    1          0          seq
    ...........uugaauggcugucacccgcaucgacu..............................................    8          0          seq
    ...........uugaauggcugucacccgcaucgacuc.............................................    4          0          seq
    ........................................uccguugauguaggcgaugacauuc..................    1          0          seq
    ........................................uccguugauguaggcgaugacauucuaucc.............    1          0          seq
    .............................................ugauguaggcgaugacauucuauccacuu.........    1          0          seq
    ..................................................uaggcgaugacauucuauccac...........    1          0          seq
    ..................................................uaggcgaugacauucuauccacuucuuuu....    1          0          seq
```
