# Supplementary material for: Identification of Conserved and Novel MicroRNAs in the Pacific Oyster Crassostrea gigas by Deep Sequencing
Source: PLoS One. 2014 Aug 19;9(8):e104371. doi: 10.1371/journal.pone.0104371 (PMC4138081; doi:10.1371/journal.pone.0104371)
Supplement: File S2 — The compressed/ZIP file archive for the predicted precursors' secondary structures and reads alignment. (ZIP) [file pone.0104371.s010.zip › second structure and reads alignment for oyster miRNAs/potential in table S7/m0309.pdf]

miRBase precursor : m0309
Total read count : 8175
m0309_5p read count : 8172
m0309_3p read count : 3
remaining reads : 0

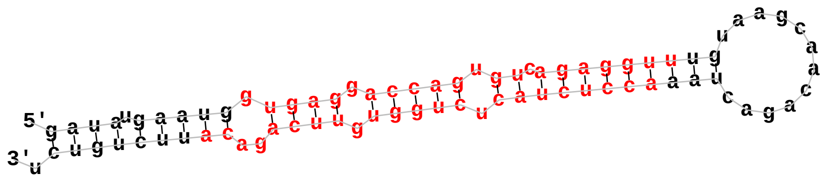

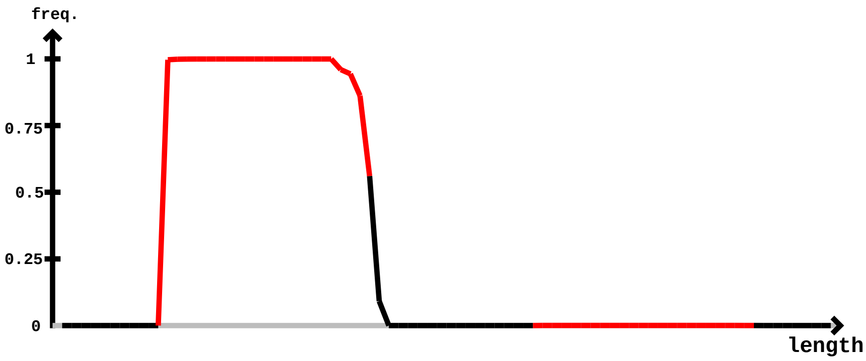


```
                                                 m0309_3p
               m0309_5p
5'- gauaugaauggugaggaccagugucagagguuuguaagcaacagacuaaaccucuacucugguguucagacauucugucu   -3'    exp
    ((((.(((((.((((.(((((.((.(((((((((............))))))))))).))))).)))).)).)))))))))).   reads      mm          sample
    ..........gugaggaccagugucaga....................................................   326        0           seq
    ..........gugaggaccagugucagag...................................................   131        0           seq
    ..........gugaggaccagugucagagg..................................................   665        0           seq
    ..........gugaggaccagugucagaggu.................................................   2463       0           seq
    ..........gugaggaccagugucagagguu................................................   3825       0           seq
    ..........gugaggaccagugucagagguuu...............................................   738        0           seq
    ...........ugaggaccagugucagagg..................................................   3          0           seq
    ...........ugaggaccagugucagaggu.................................................   2          0           seq
    ...........ugaggaccagugucagagguu................................................   3          0           seq
    ...........ugaggaccagugucagagguuu...............................................   8          0           seq
    ............gaggaccagugucagaggu.................................................   1          0           seq
    ............gaggaccagugucagagguu................................................   3          0           seq
    ............gaggaccagugucagagguuu...............................................   1          0           seq
    .............aggaccagugucagagguu................................................   1          0           seq
    .............aggaccagugucagagguuu...............................................   1          0           seq
    ...............gaccagugucagagguuu...............................................   1          0           seq
    .................................................accucuacucugguguucaga..........   1          0           seq
    .................................................accucuacucugguguucagaca........   2          0           seq
```
