# Supplementary material for: Identification of Conserved and Novel MicroRNAs in the Pacific Oyster Crassostrea gigas by Deep Sequencing
Source: PLoS One. 2014 Aug 19;9(8):e104371. doi: 10.1371/journal.pone.0104371 (PMC4138081; doi:10.1371/journal.pone.0104371)
Supplement: File S2 — The compressed/ZIP file archive for the predicted precursors' secondary structures and reads alignment. (ZIP) [file pone.0104371.s010.zip › second structure and reads alignment for oyster miRNAs/potential in table S7/m0316.pdf]

miRBase precursor : m0316
Total read count : 5146
m0316_5p read count : 227
m0316_3p read count : 4919
remaining reads : 0

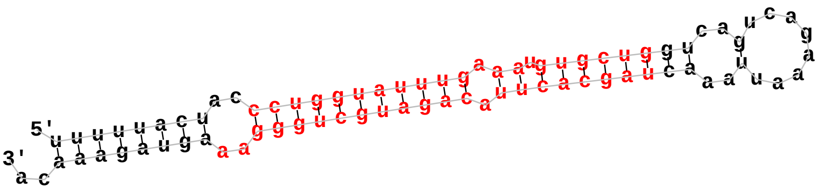

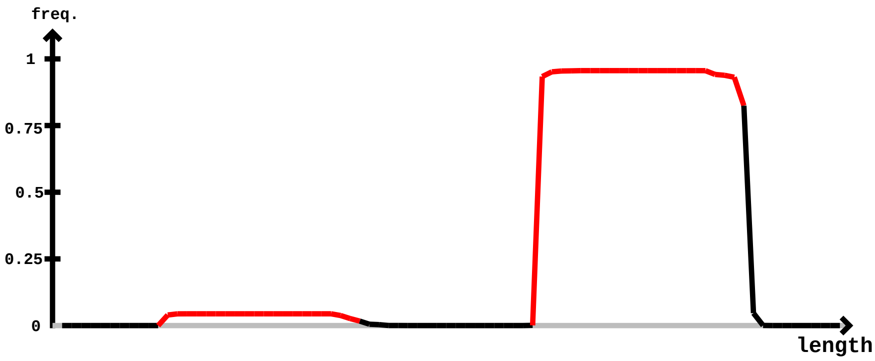


```
                                                 m0316_3p
          m0316_5p
5'- uuuuuacuacccugguauuugaaaugugcuggucagucagaaauuaaacuagcacuuacagaugcugggaaaguagaaaca   -3'    exp
    ((((((((..(((((((((((.((.((((((((..(........)..)))))))))).)))))))))))..))))))))..    reads      mm         sample
    ..........ccugguauuugaaaugug.....................................................    33         0          seq
    ..........ccugguauuugaaaugugc....................................................    53         0          seq
    ..........ccugguauuugaaaugugcu...................................................    41         0          seq
    ..........ccugguauuugaaaugugcug..................................................    55         0          seq
    ..........ccugguauuugaaaugugcugg.................................................    7          0          seq
    ..........ccugguauuugaaaugugcuggu................................................    14         0          seq
    ..........ccugguauuugaaaugugcugguca..............................................    1          0          seq
    ...........cugguauuugaaaugugc....................................................    6          0          seq
    ...........cugguauuugaaaugugcu...................................................    8          0          seq
    ...........cugguauuugaaaugugcug..................................................    5          0          seq
    ...........cugguauuugaaaugugcuggu................................................    3          0          seq
    ............ugguauuugaaaugugcugg.................................................    1          0          seq
    ................................................cuagcacuuacagaugcu...............    1          0          seq
    ................................................cuagcacuuacagaugcuggga...........    1          0          seq
    ................................................cuagcacuuacagaugcugggaa..........    2          0          seq
    .................................................uagcacuuacagaugcug..............    72         0          seq
    .................................................uagcacuuacagaugcugg.............    16         0          seq
    .................................................uagcacuuacagaugcuggg............    31         0          seq
    .................................................uagcacuuacagaugcuggga...........    523        0          seq
    .................................................uagcacuuacagaugcugggaa..........    3950       0          seq
    .................................................uagcacuuacagaugcugggaaa.........    207        0          seq
    .................................................uagcacuuacagaugcugggaaag........    2          0          seq
    ..................................................agcacuuacagaugcugg.............    1          0          seq
    ..................................................agcacuuacagaugcuggg............    6          0          seq
    ..................................................agcacuuacagaugcuggga...........    17         0          seq
    ..................................................agcacuuacagaugcugggaa..........    38         0          seq
    ..................................................agcacuuacagaugcugggaaa.........    29         0          seq
    ...................................................gcacuuacagaugcuggga...........    6          0          seq
    ...................................................gcacuuacagaugcugggaa..........    8          0          seq
    ...................................................gcacuuacagaugcugggaaa.........    2          0          seq
    ....................................................cacuuacagaugcuggga...........    2          0          seq
    ....................................................cacuuacagaugcugggaa..........    1          0          seq
    .....................................................acuuacagaugcugggaa..........    4          0          seq
```
