# Supplementary material for: Identification of Conserved and Novel MicroRNAs in the Pacific Oyster Crassostrea gigas by Deep Sequencing
Source: PLoS One. 2014 Aug 19;9(8):e104371. doi: 10.1371/journal.pone.0104371 (PMC4138081; doi:10.1371/journal.pone.0104371)
Supplement: File S2 — The compressed/ZIP file archive for the predicted precursors' secondary structures and reads alignment. (ZIP) [file pone.0104371.s010.zip › second structure and reads alignment for oyster miRNAs/potential in table S7/m0338.pdf]

miRBase precursor : m0338
Total read count : 54
m0338_5p read count : 52
m0338_3p read count : 0
remaining reads : 2

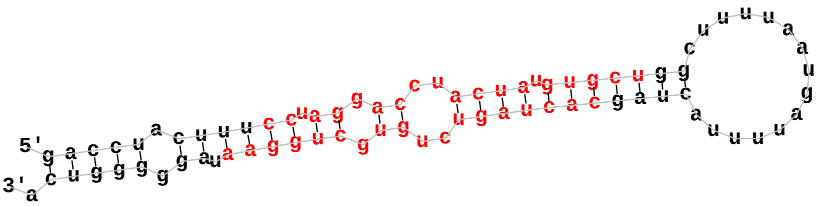

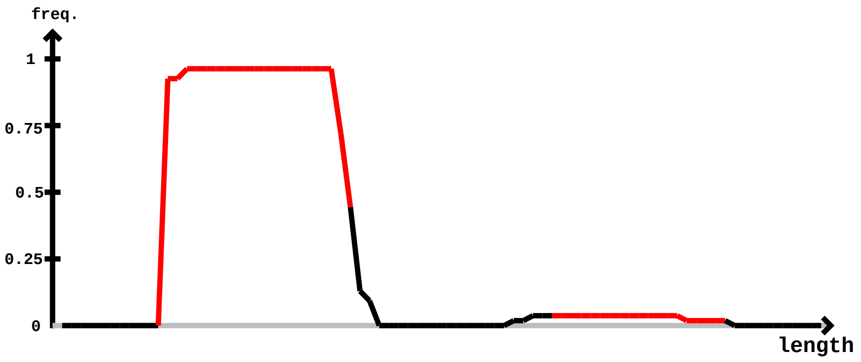


```
                 m0338_5p
                                                m0338_3p
5'- gaccuacuuuccuaggaccuacuaugugcuggcuuuuaaugauuuuacuagcacuagucugugcuggaauaggggguca   -3'    exp
    (((((.((((((.((.((..((((.(((((((...............)))))))))))..)).)))))).)).))))).     reads      mm         sample
    ..........ccuaggaccuacuaugug...................................................     13         0          seq
    ..........ccuaggaccuacuaugugc..................................................     15         0          seq
    ..........ccuaggaccuacuaugugcu.................................................     15         0          seq
    ..........ccuaggaccuacuaugugcug................................................     2          0          seq
    ..........ccuaggaccuacuaugugcugg...............................................     5          0          seq
    ............uaggaccuacuaugugcu.................................................     2          0          seq
    ...............................................acuagcacuagucugugc..............     1          0          seq
    ................................................uagcacuagucugugcuggaa..........     1          0          seq
```
