# Supplementary material for: Identification of Conserved and Novel MicroRNAs in the Pacific Oyster Crassostrea gigas by Deep Sequencing
Source: PLoS One. 2014 Aug 19;9(8):e104371. doi: 10.1371/journal.pone.0104371 (PMC4138081; doi:10.1371/journal.pone.0104371)
Supplement: File S2 — The compressed/ZIP file archive for the predicted precursors' secondary structures and reads alignment. (ZIP) [file pone.0104371.s010.zip › second structure and reads alignment for oyster miRNAs/potential in table S7/m0341.pdf]

miRBase precursor : m0341
Total read count : 117
m0341_5p read count : 52
m0341_3p read count : 64
remaining reads : 1

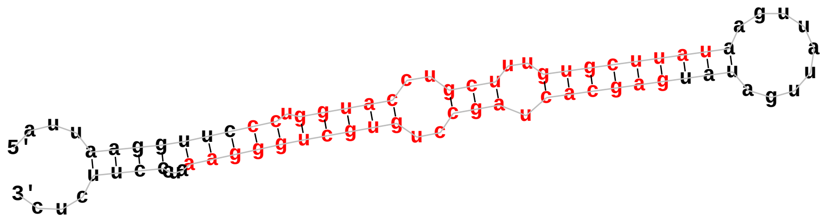

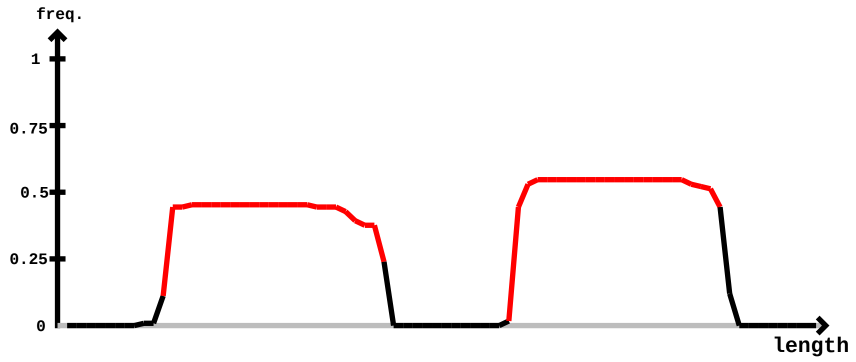


```
                                                   m0341_3p
              m0341_5p
5'- auuaagguucccugguaccugcuuugugcuuauaaguuauugauaugagcacuagccugugcugggaaaucccuucuc   -3'    exp
    ...(((((((((.(((((..(((..(((((((((.........))))))))).)))..))))))))))...))))...   reads      mm         sample
    .......uucccugguaccugcuuu.....................................................   1          0          seq
    .........cccugguaccugcuuugug..................................................   1          0          seq
    .........cccugguaccugcuuugugc.................................................   1          0          seq
    .........cccugguaccugcuuugugcu................................................   2          0          seq
    .........cccugguaccugcuuugugcuua..............................................   8          0          seq
    ..........ccugguaccugcuuugug..................................................   1          0          seq
    ..........ccugguaccugcuuugugc.................................................   3          0          seq
    ..........ccugguaccugcuuugugcuua..............................................   7          0          seq
    ..........ccugguaccugcuuugugcuuau.............................................   28         0          seq
    ............ugguaccugcuuugugcuua..............................................   1          0          seq
    .............................................ugagcacuagccugugcug..............   2          0          seq
    ..............................................gagcacuagccugugcugg.............   1          0          seq
    ..............................................gagcacuagccugugcuggga...........   5          0          seq
    ..............................................gagcacuagccugugcugggaa..........   33         0          seq
    ..............................................gagcacuagccugugcugggaaa.........   11         0          seq
    ...............................................agcacuagccugugcuggg............   1          0          seq
    ...............................................agcacuagccugugcuggga...........   2          0          seq
    ...............................................agcacuagccugugcugggaa..........   4          0          seq
    ...............................................agcacuagccugugcugggaaa.........   3          0          seq
    ................................................gcacuagccugugcuggga...........   1          0          seq
    ................................................gcacuagccugugcugggaa..........   1          0          seq
```
