# Supplementary material for: Identification of Conserved and Novel MicroRNAs in the Pacific Oyster Crassostrea gigas by Deep Sequencing
Source: PLoS One. 2014 Aug 19;9(8):e104371. doi: 10.1371/journal.pone.0104371 (PMC4138081; doi:10.1371/journal.pone.0104371)
Supplement: File S2 — The compressed/ZIP file archive for the predicted precursors' secondary structures and reads alignment. (ZIP) [file pone.0104371.s010.zip › second structure and reads alignment for oyster miRNAs/potential in table S7/m0342.pdf]

miRBase precursor : m0342
Total read count : 105
m0342_5p read count : 79
m0342_3p read count : 25
remaining reads : 1

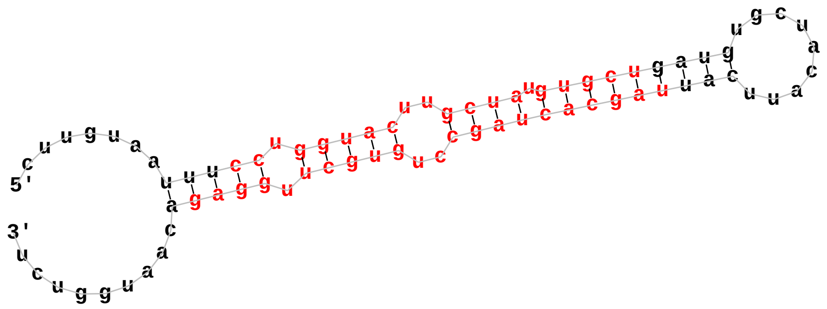

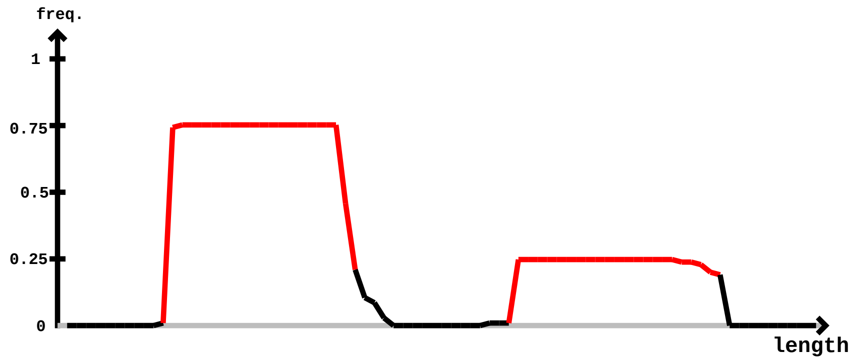


```
                                                          m0342_3p
          m0342_5p
5'- cuuguaauuuccugguacuugcuaugugcugaugugcuacauucauuagcacuagccugugcuuggagacaauggucu   -3'    exp
    .......(((((.(((((..((((.(((((((((.........)))))))))))))..))))).)))))..........    reads      mm         sample
    .........uccugguacuugcuaugugcu................................................    1          0          seq
    ..........ccugguacuugcuaugug..................................................    31         0          seq
    ..........ccugguacuugcuaugugc.................................................    25         0          seq
    ..........ccugguacuugcuaugugcu................................................    10         0          seq
    ..........ccugguacuugcuaugugcug...............................................    2          0          seq
    ..........ccugguacuugcuaugugcuga..............................................    6          0          seq
    ..........ccugguacuugcuaugugcugau.............................................    3          0          seq
    ...........cugguacuugcuaugugc.................................................    1          0          seq
    ...........................................cauuagcacuagccugugcu...............    1          0          seq
    ..............................................uagcacuagccugugcuug.............    1          0          seq
    ..............................................uagcacuagccugugcuugg............    3          0          seq
    ..............................................uagcacuagccugugcuugga...........    1          0          seq
    ..............................................uagcacuagccugugcuuggag..........    20         0          seq
```
