# Supplementary material for: Identification of Conserved and Novel MicroRNAs in the Pacific Oyster Crassostrea gigas by Deep Sequencing
Source: PLoS One. 2014 Aug 19;9(8):e104371. doi: 10.1371/journal.pone.0104371 (PMC4138081; doi:10.1371/journal.pone.0104371)
Supplement: File S2 — The compressed/ZIP file archive for the predicted precursors' secondary structures and reads alignment. (ZIP) [file pone.0104371.s010.zip › second structure and reads alignment for oyster miRNAs/potential in table S7/m0345.pdf]

miRBase precursor	: m0345
Total read count	: 39
m0345_5p read count	: 9
m0345_3p read count	: 30
remaining reads	: 0

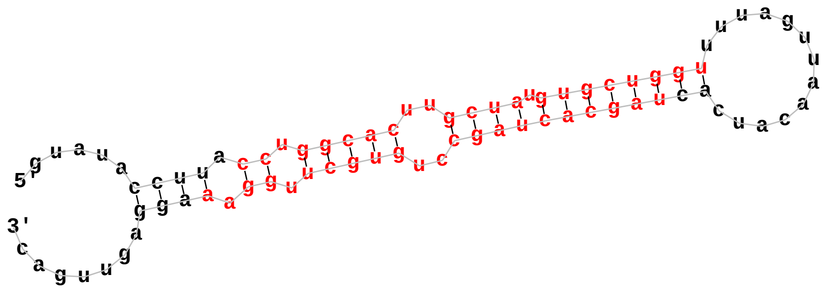

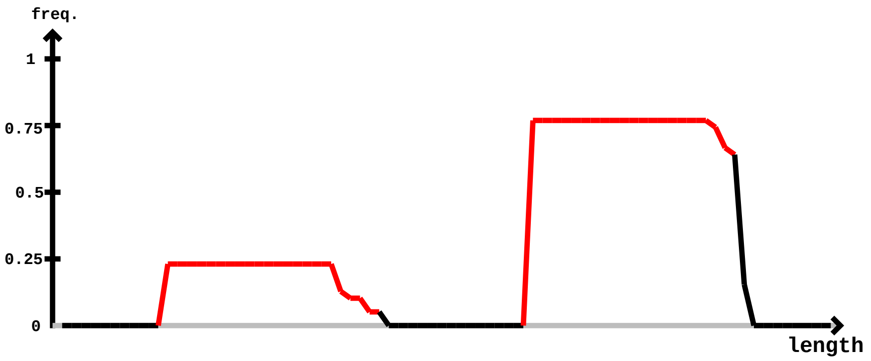


```
                    m0345_5p
                                                m0345_3p
5'- guauaccuuaccuggcacuugcuaugugcugguuuuaguuaacaucacuagcacuagccugugcuuggaaaggaguugac   -3'    exp
    .....((((.((.(((((..((((.((((((((............))))))))))))..))))).)).)))).......     reads      mm         sample
    ..........ccuggcacuugcuaugug..................................................     4          0          seq
    ..........ccuggcacuugcuaugugc.................................................     1          0          seq
    ..........ccuggcacuugcuaugugcug...............................................     2          0          seq
    ..........ccuggcacuugcuaugugcuggu.............................................     2          0          seq
    ..............................................uagcacuagccugugcuug.............     1          0          seq
    ..............................................uagcacuagccugugcuugg............     3          0          seq
    ..............................................uagcacuagccugugcuugga...........     1          0          seq
    ..............................................uagcacuagccugugcuuggaa..........     19         0          seq
    ..............................................uagcacuagccugugcuuggaaa.........     6          0          seq
```
