# Supplementary material for: Identification of Conserved and Novel MicroRNAs in the Pacific Oyster Crassostrea gigas by Deep Sequencing
Source: PLoS One. 2014 Aug 19;9(8):e104371. doi: 10.1371/journal.pone.0104371 (PMC4138081; doi:10.1371/journal.pone.0104371)
Supplement: File S2 — The compressed/ZIP file archive for the predicted precursors' secondary structures and reads alignment. (ZIP) [file pone.0104371.s010.zip › second structure and reads alignment for oyster miRNAs/potential in table S7/m0348.pdf]

miRBase precursor : m0348
Total read count : 256
m0348_5p read count : 56
m0348_3p read count : 200
remaining reads : 0

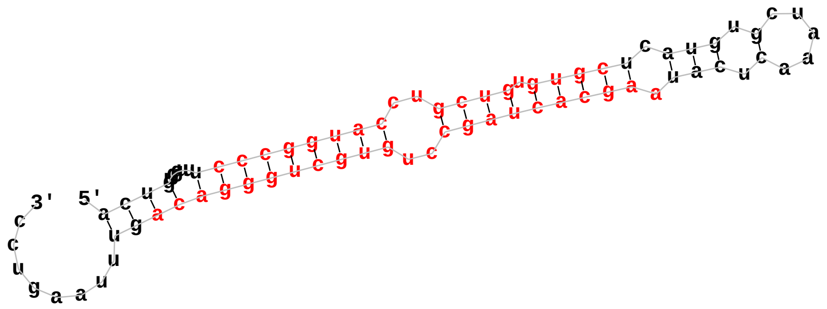

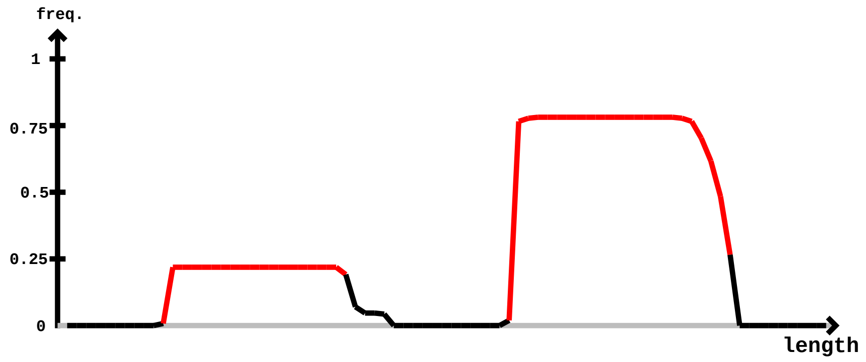


```
                                             m0348_3p
                m0348_5p
5'- acugugguuucccgguaccugcugugugcucaugugcuaaacucauaagcacuagccugugcugggacaguuuaagucc   -3'    exp
    ((((.....(((((((((..((((.(((((.(((.(.....).))).)))))))))..)))))))))))))........   reads      mm        sample
    .........ucccgguaccugcugugug...................................................   1          0         seq
    .........ucccgguaccugcugugugc..................................................   1          0         seq
    ..........cccgguaccugcugugug...................................................   6          0         seq
    ..........cccgguaccugcugugugc..................................................   30         0         seq
    ..........cccgguaccugcugugugcu.................................................   6          0         seq
    ..........cccgguaccugcugugugcuca...............................................   1          0         seq
    ..........cccgguaccugcugugugcucau..............................................   11         0         seq
    .............................................uaagcacuagccugugcu................   1          0         seq
    .............................................uaagcacuagccugugcugg..............   2          0         seq
    .............................................uaagcacuagccugugcuggga............   2          0         seq
    ..............................................aagcacuagccugugcug...............   3          0         seq
    ..............................................aagcacuagccugugcugg..............   14         0         seq
    ..............................................aagcacuagccugugcuggg.............   21         0         seq
    ..............................................aagcacuagccugugcuggga............   29         0         seq
    ..............................................aagcacuagccugugcugggac...........   56         0         seq
    ..............................................aagcacuagccugugcugggaca..........   68         0         seq
    ...............................................agcacuagccugugcuggg.............   1          0         seq
    ...............................................agcacuagccugugcuggga............   2          0         seq
    ................................................gcacuagccugugcuggga............   1          0         seq
```
