# Supplementary material for: Identification of Conserved and Novel MicroRNAs in the Pacific Oyster Crassostrea gigas by Deep Sequencing
Source: PLoS One. 2014 Aug 19;9(8):e104371. doi: 10.1371/journal.pone.0104371 (PMC4138081; doi:10.1371/journal.pone.0104371)
Supplement: File S2 — The compressed/ZIP file archive for the predicted precursors' secondary structures and reads alignment. (ZIP) [file pone.0104371.s010.zip › second structure and reads alignment for oyster miRNAs/potential in table S7/m0445.pdf]

miRBase precursor : m0445
Total read count : 170
m0445_5p read count : 1
m0445_3p read count : 169
remaining reads : 0

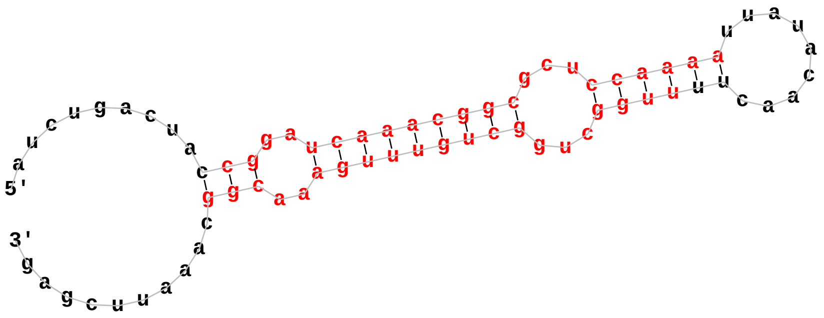

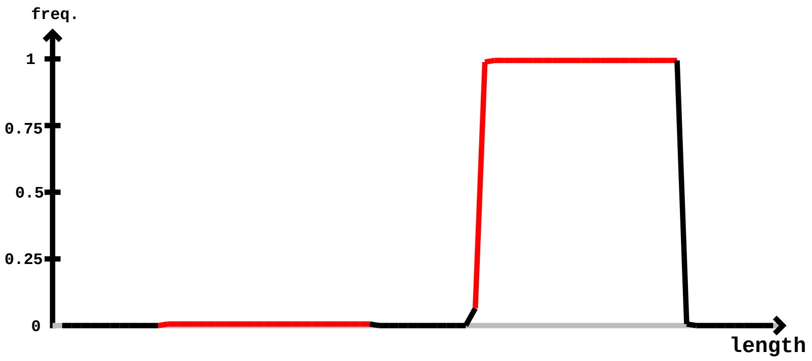


```
                m0445_5p
                                             m0445_3p
5'- aucugacuaccggaucaaacggcgcuccaaaauuauacaacuuuuggcuggcuguuugaaacggcaaauucgag   -3'    exp
    .........(((..(((((((((...((((((.........))))))...)))))))))..)))..........    reads      mm         sample
    ..........cggaucaaacggcgcuccaaaa..........................................    1          0          seq
    ..........................................uuuggcuggcuguuugaaacgg..........    11         0          seq
    ...........................................uuggcuggcuguuugaaacgg..........    156        0          seq
    ...........................................uuggcuggcuguuugaaacggc.........    1          0          seq
    ............................................uggcuggcuguuugaaacgg..........    1          0          seq
```
