# Supplementary material for: Identification of Conserved and Novel MicroRNAs in the Pacific Oyster Crassostrea gigas by Deep Sequencing
Source: PLoS One. 2014 Aug 19;9(8):e104371. doi: 10.1371/journal.pone.0104371 (PMC4138081; doi:10.1371/journal.pone.0104371)
Supplement: File S2 — The compressed/ZIP file archive for the predicted precursors' secondary structures and reads alignment. (ZIP) [file pone.0104371.s010.zip › second structure and reads alignment for oyster miRNAs/potential in table S7/m0452.pdf]

miRBase precursor	: m0452
Total read count	: 104
m0452_5p read count	: 102
m0452_3p read count	: 2
remaining reads	: 0

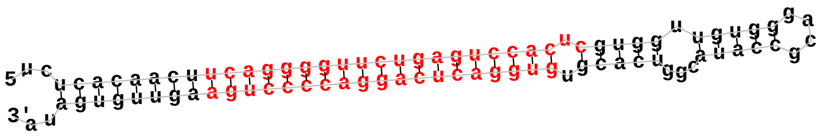

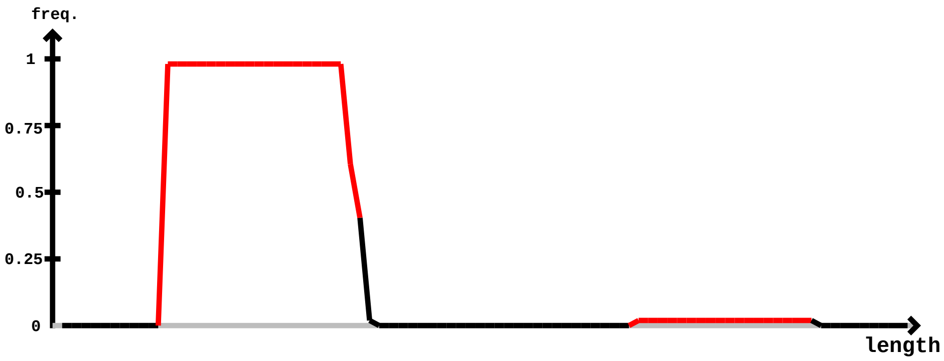


```
                                                          m0452_3p
          m0452_5p
5'- ucucacaacuucagggguucugaguccacucgugguugugggacgccauacggucacguguggacucaggaccccugaaguugugaua   -3'    exp
    ..(((((((((((((((((((((((((((.(((((.(((((....)))))...))))).)))))))))))))))))))))))))))..    reads      mm         sample
    ..........ucagggguucugaguccac...........................................................    39         0          seq
    ..........ucagggguucugaguccacu..........................................................    21         0          seq
    ..........ucagggguucugaguccacuc.........................................................    40         0          seq
    ..........ucagggguucugaguccacucg........................................................    2          0          seq
    ...........................................................guggacucaggaccccuga..........    2          0          seq
```
