# Supplementary material for: Identification of Conserved and Novel MicroRNAs in the Pacific Oyster Crassostrea gigas by Deep Sequencing
Source: PLoS One. 2014 Aug 19;9(8):e104371. doi: 10.1371/journal.pone.0104371 (PMC4138081; doi:10.1371/journal.pone.0104371)
Supplement: File S2 — The compressed/ZIP file archive for the predicted precursors' secondary structures and reads alignment. (ZIP) [file pone.0104371.s010.zip › second structure and reads alignment for oyster miRNAs/potential in table S7/m0538.pdf]

miRBase precursor : m0538
Total read count : 540
m0538_5p read count : 9
m0538_3p read count : 216
remaining reads : 315

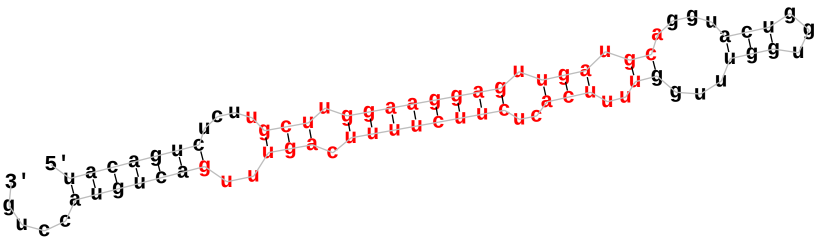

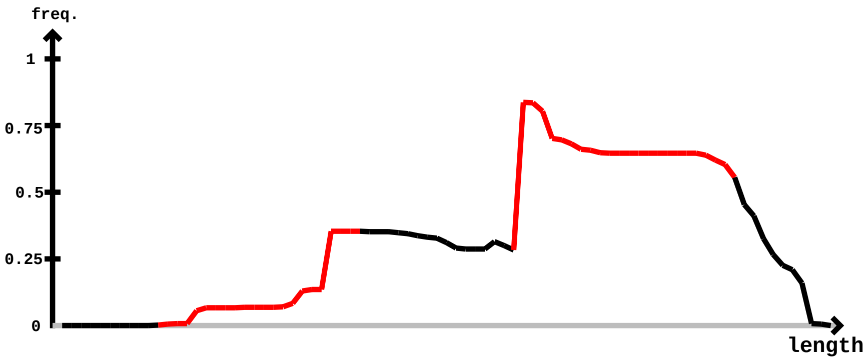

m0538_3p

m0538_5p

```
5'- uacagucucuugcuuggaaggaguugaugcagguacuggugguuugguuuucacucuucuuuucaguuugacuguaccug   -3'    exp
    (((((((....(((.((((((((.(((.((....(((...)))...))..)))..)))))))).))).))..)))))))....    reads      mm         sample
    .........uugcuuggaaggaguugaugcaggu..............................................    1          0          seq
    ..........ugcuuggaaggaguugaugca.................................................    1          0          seq
    ..........ugcuuggaaggaguugaugcagguacu...........................................    1          0          seq
    ...........gcuuggaaggaguugaugcagguac............................................    1          0          seq
    .............uuggaaggaguugaugcaggu..............................................    1          0          seq
    .............uuggaaggaguugaugcaggua.............................................    2          0          seq
    .............uuggaaggaguugaugcagguac............................................    3          0          seq
    .............uuggaaggaguugaugcagguacu...........................................    1          0          seq
    .............uuggaaggaguugaugcagguacug..........................................    2          0          seq
    .............uuggaaggaguugaugcagguacugg.........................................    8          0          seq
    .............uuggaaggaguugaugcagguacuggu........................................    8          0          seq
    .............uuggaaggaguugaugcagguacuggug.......................................    1          0          seq
    ..............uggaaggaguugaugcagguacu...........................................    1          0          seq
    ..............uggaaggaguugaugcagguacugg.........................................    1          0          seq
    ..............uggaaggaguugaugcagguacuggu........................................    3          0          seq
    ..............uggaaggaguugaugcagguacuggug.......................................    1          0          seq
    ..................aggaguugaugcagguacuggugguu....................................    1          0          seq
    ......................guugaugcagguacuggugguuuggu................................    1          0          seq
    .......................uugaugcagguacugguggu.....................................    1          0          seq
    .......................uugaugcagguacuggugguu....................................    2          0          seq
    .......................uugaugcagguacuggugguuu...................................    1          0          seq
    .......................uugaugcagguacuggugguuug..................................    1          0          seq
    .......................uugaugcagguacuggugguuugg.................................    1          0          seq
    .......................uugaugcagguacuggugguuugguuu..............................    1          0          seq
    ........................ugaugcagguacuggugguuu...................................    5          0          seq
    ........................ugaugcagguacuggugguuug..................................    3          0          seq
    ........................ugaugcagguacuggugguuugguu...............................    3          0          seq
    ........................ugaugcagguacuggugguuugguuu..............................    11         0          seq
    ........................ugaugcagguacuggugguuugguuuu.............................    1          0          seq
    ........................ugaugcagguacuggugguuugguuuuc............................    2          0          seq
    .........................gaugcagguacuggugguu....................................    1          0          seq
    .........................gaugcagguacuggugguuugguu...............................    1          0          seq
    .........................gaugcagguacuggugguuugguuuu.............................    1          0          seq
    ...........................ugcagguacuggugguuu...................................    4          0          seq
```

```
                                            m0538_3p
          m0538_5p
uacagucucuugcuuggaaggaguugaugcagguacuggugguuugguuuucacucuucuuuucaguuugacuguaccug

...........................ugcagguacuggugguuug..................................    5     0     seq
...........................ugcagguacuggugguuugg.................................    12    0     seq
...........................ugcagguacuggugguuuggu................................    2     0     seq
...........................ugcagguacuggugguuugguu...............................    14    0     seq
...........................ugcagguacuggugguuugguuu..............................    54    0     seq
...........................ugcagguacuggugguuugguuuu.............................    1     0     seq
...........................ugcagguacuggugguuugguuuuc............................    7     0     seq
...........................ugcagguacuggugguuugguuuuca...........................    11    0     seq
...........................ugcagguacuggugguuugguuuucac..........................    2     0     seq
...........................ugcagguacuggugguuugguuuucacu.........................    5     0     seq
...........................ugcagguacuggugguuugguuuucacuc........................    1     0     seq
...........................................uugguuuucacucuucuuuucaguuugac........    1     0     seq
............................................ugguuuucacucuucuuuucaguu............    1     0     seq
............................................ugguuuucacucuucuuuucaguuug..........    1     0     seq
............................................ugguuuucacucuucuuuucaguuugac........    12    0     seq
............................................ugguuuucacucuucuuuucaguuugacu.......    5     0     seq
.............................................gguuuucacucuucuuuucaguuu...........    2     0     seq
...............................................uuuucacucuucuuuucag..............    4     0     seq
...............................................uuuucacucuucuuuucagu.............    10    0     seq
...............................................uuuucacucuucuuuucaguu............    8     0     seq
...............................................uuuucacucuucuuuucaguuu...........    24    0     seq
...............................................uuuucacucuucuuuucaguuug..........    54    0     seq
...............................................uuuucacucuucuuuucaguuuga.........    23    0     seq
...............................................uuuucacucuucuuuucaguuugac........    32    0     seq
...............................................uuuucacucuucuuuucaguuugacu.......    25    0     seq
...............................................uuuucacucuucuuuucaguuugacug......    19    0     seq
...............................................uuuucacucuucuuuucaguuugacugu.....    7     0     seq
...............................................uuuucacucuucuuuucaguuugacugua....    26    0     seq
...............................................uuuucacucuucuuuucaguuugacuguac...    80    0     seq
................................................uuucacucuucuuuucaguuugacuguac...    2     0     seq
.................................................uucacucuucuuuucaguuugacu.......    1     0     seq
..................................................ucacucuucuuuucaguuugac........    1     0     seq
..................................................ucacucuucuuuucaguuugacu.......    1     0     seq
..................................................ucacucuucuuuucaguuugacug......    3     0     seq
..................................................ucacucuucuuuucaguuugacugu.....    2     0     seq
..................................................ucacucuucuuuucaguuugacugua....    1     0     seq
..................................................ucacucuucuuuucaguuugacuguaccu.    3     0     seq
....................................................acucuucuuuucaguuugacuguacc..    1     0     seq
```
