# Supplementary material for: Identification of Conserved and Novel MicroRNAs in the Pacific Oyster Crassostrea gigas by Deep Sequencing
Source: PLoS One. 2014 Aug 19;9(8):e104371. doi: 10.1371/journal.pone.0104371 (PMC4138081; doi:10.1371/journal.pone.0104371)
Supplement: File S2 — The compressed/ZIP file archive for the predicted precursors' secondary structures and reads alignment. (ZIP) [file pone.0104371.s010.zip › second structure and reads alignment for oyster miRNAs/potential in table S7/m0556.pdf]

miRBase precursor : m0556
Total read count : 426
m0556_5p read count : 425
m0556_3p read count : 1
remaining reads : 0

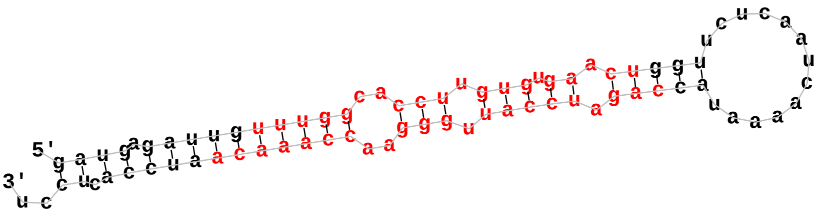

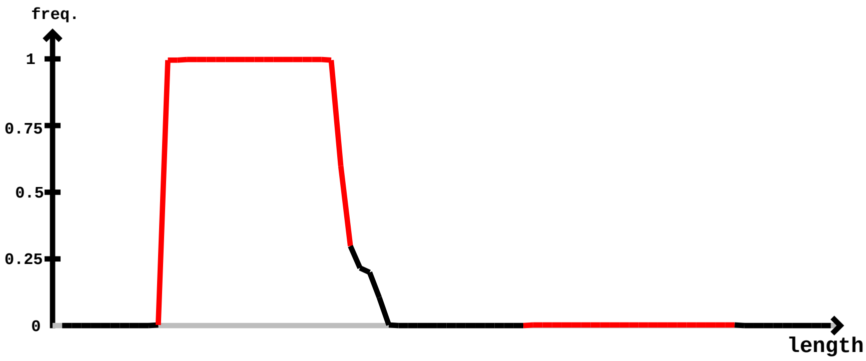


```
                m0556_5p
                                                m0556_3p
5'- gaugagauuguuuggcaccuugugugaacugguucucaaucaaaauaccagauccauugggaaccaaacaauccacuccu   -3'   exp
    ..((.((((((((((..(((.(((.((.(((((.............))))).))))).)))..)))))))))))).....    reads   mm   sample
    .........guuuggcaccuuguguga.....................................................    1       0    seq
    ..........uuuggcaccuugugugaa....................................................    169     0    seq
    ..........uuuggcaccuugugugaac...................................................    128     0    seq
    ..........uuuggcaccuugugugaacu..................................................    35      0    seq
    ..........uuuggcaccuugugugaacug.................................................    7       0    seq
    ..........uuuggcaccuugugugaacugg................................................    40      0    seq
    ..........uuuggcaccuugugugaacuggu...............................................    44      0    seq
    ............uggcaccuugugugaacugguu..............................................    1       0    seq
    ................................................cagauccauugggaaccaaaca..........    1       0    seq
```
