# Supplementary material for: Identification of Conserved and Novel MicroRNAs in the Pacific Oyster Crassostrea gigas by Deep Sequencing
Source: PLoS One. 2014 Aug 19;9(8):e104371. doi: 10.1371/journal.pone.0104371 (PMC4138081; doi:10.1371/journal.pone.0104371)
Supplement: File S2 — The compressed/ZIP file archive for the predicted precursors' secondary structures and reads alignment. (ZIP) [file pone.0104371.s010.zip › second structure and reads alignment for oyster miRNAs/potential in table S7/m0571.pdf]

miRBase precursor : m0571
Total read count : 11482
m0571_5p read count : 8678
m0571_3p read count : 2727
remaining reads : 77

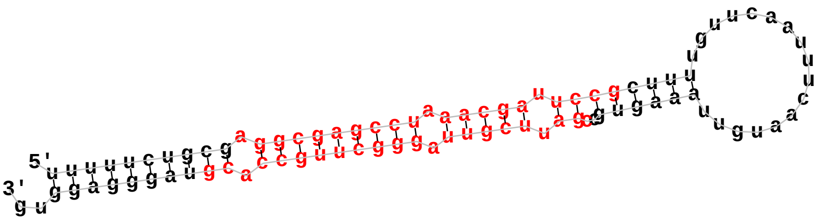

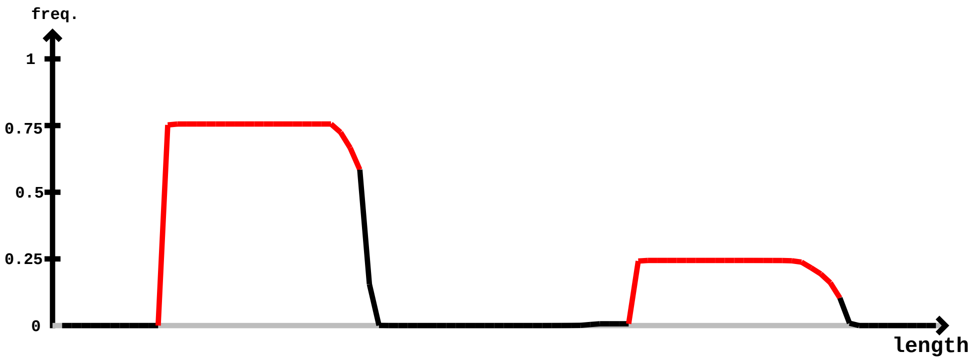


```
                                                                 m0571_3p
           m0571_5p
5'- uuuuucugcgaggcgagccuaaacgauuccgcuuuuguucaauuucaauguuaaagugccgauucguuagggcuugccacguagggaggug   -3'    exp
    .(((((((((.(((((((((.(((((.((((((((.................))))))..)).))))).))))))))).)))))))))...    reads     mm        sample
    ..........aggcgagccuaaacgauu...............................................................    361       0         seq
    ..........aggcgagccuaaacgauuc..............................................................    661       0         seq
    ..........aggcgagccuaaacgauucc.............................................................    933       0         seq
    ..........aggcgagccuaaacgauuccg............................................................    4907      0         seq
    ..........aggcgagccuaaacgauuccgc...........................................................    1770      0         seq
    ..........aggcgagccuaaacgauuccgcu..........................................................    5         0         seq
    ..........aggcgagccuaaacgauuccgcuu.........................................................    1         0         seq
    ...........ggcgagccuaaacgauuc..............................................................    6         0         seq
    ...........ggcgagccuaaacgauucc.............................................................    5         0         seq
    ...........ggcgagccuaaacgauuccg............................................................    22        0         seq
    ...........ggcgagccuaaacgauuccgc...........................................................    6         0         seq
    ............gcgagccuaaacgauuccgc...........................................................    1         0         seq
    ..................................................uuaaagugccgauucguuagggcu.................    1         0         seq
    ...................................................uaaagugccgauucguuagggcuug...............    1         0         seq
    ....................................................aaagugccgauucguuagggcuug...............    3         0         seq
    .....................................................aagugccgauucguuagggcuug...............    4         0         seq
    ......................................................agugccgauucguuaggg...................    1         0         seq
    ......................................................agugccgauucguuagggcu.................    1         0         seq
    ......................................................agugccgauucguuagggcuu................    3         0         seq
    ......................................................agugccgauucguuagggcuug...............    31        0         seq
    .......................................................gugccgauucguuagggc..................    1         0         seq
    .......................................................gugccgauucguuagggcuu................    8         0         seq
    .......................................................gugccgauucguuagggcuug...............    19        0         seq
    .......................................................gugccgauucguuagggcuugc..............    4         0         seq
    ...........................................................cgauucguuagggcuugc..............    240       0         seq
    ...........................................................cgauucguuagggcuugcc.............    260       0         seq
    ...........................................................cgauucguuagggcuugcca............    377       0         seq
    ...........................................................cgauucguuagggcuugccac...........    647       0         seq
    ...........................................................cgauucguuagggcuugccacg..........    1077      0         seq
    ...........................................................cgauucguuagggcuugccacgu.........    100       0         seq
    ............................................................gauucguuagggcuugcc.............    2         0         seq
    ............................................................gauucguuagggcuugcca............    3         0         seq
    ............................................................gauucguuagggcuugccac...........    10        0         seq
    ............................................................gauucguuagggcuugccacg..........    9         0         seq
```

```
                                                            m0571_3p
          m0571_5p
uuuuucugcgaggcgagccuaaacgauuccgcuuuguucaauuucaauguuaaagugccgauucguuagggcuugccacguagggaggug

...........................................................gauucguuagggcuugccacgu.........    2          0          seq
```
