# Supplementary figures and images for: Identification of Conserved and Novel MicroRNAs in the Pacific Oyster Crassostrea gigas by Deep Sequencing
Source: PLoS One. 2014 Aug 19;9(8):e104371. doi: 10.1371/journal.pone.0104371 (PMC4138081; doi:10.1371/journal.pone.0104371)

```

cgi-miR-1994b-5p read count25
cgi-miR-1994b-3p read count88448
remaining reads          : 1

```

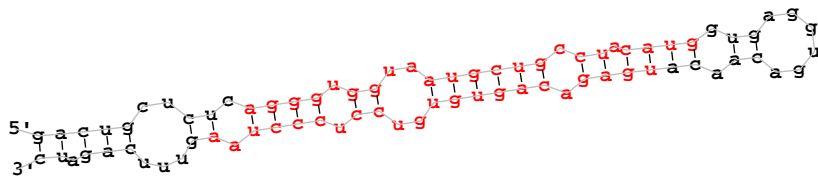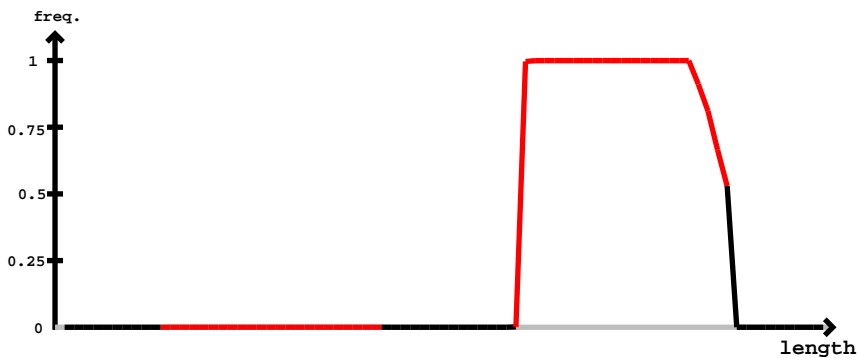

cgi-miR-1994b-3p

cgi-miR-1994b-5p

[illegible]

Supplement: File S2 — The compressed/ZIP file archive for the predicted precursors' secondary structures and reads alignment. (ZIP) [file pone.0104371.s010.zip › second structure and reads alignment for oyster miRNAs/conserved in table S4/cgi-miR-1994b.pdf]

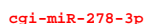[illegible]

Supplement: File S2 — The compressed/ZIP file archive for the predicted precursors' secondary structures and reads alignment. (ZIP) [file pone.0104371.s010.zip › second structure and reads alignment for oyster miRNAs/conserved in table S4/cgi-miR-278.pdf]

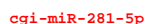

cgi-miR-281-3p

[illegible]

Supplement: File S2 — The compressed/ZIP file archive for the predicted precursors' secondary structures and reads alignment. (ZIP) [file pone.0104371.s010.zip › second structure and reads alignment for oyster miRNAs/conserved in table S4/cgi-miR-281.pdf]

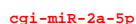

cqi-miR-2a-3p

[illegible]

Supplement: File S2 — The compressed/ZIP file archive for the predicted precursors' secondary structures and reads alignment. (ZIP) [file pone.0104371.s010.zip › second structure and reads alignment for oyster miRNAs/conserved in table S4/cgi-miR-2a.pdf]

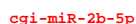

cqi-miR-2b-3p

[illegible]

Supplement: File S2 — The compressed/ZIP file archive for the predicted precursors' secondary structures and reads alignment. (ZIP) [file pone.0104371.s010.zip › second structure and reads alignment for oyster miRNAs/conserved in table S4/cgi-miR-2b.pdf]

[illegible]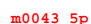

m0043 3p

[illegible]

Supplement: File S2 — The compressed/ZIP file archive for the predicted precursors' secondary structures and reads alignment. (ZIP) [file pone.0104371.s010.zip › second structure and reads alignment for oyster miRNAs/novel in table S5/m0043.pdf]

5, a u u g u u a u g u a a u g c u u g c a a a c u u c a u u g c u a g u c a g u u a u

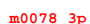[illegible]

Supplement: File S2 — The compressed/ZIP file archive for the predicted precursors' secondary structures and reads alignment. (ZIP) [file pone.0104371.s010.zip › second structure and reads alignment for oyster miRNAs/novel in table S5/m0078.pdf]

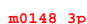[illegible]

Supplement: File S2 — The compressed/ZIP file archive for the predicted precursors' secondary structures and reads alignment. (ZIP) [file pone.0104371.s010.zip › second structure and reads alignment for oyster miRNAs/novel in table S5/m0148.pdf]

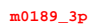[illegible]

Supplement: File S2 — The compressed/ZIP file archive for the predicted precursors' secondary structures and reads alignment. (ZIP) [file pone.0104371.s010.zip › second structure and reads alignment for oyster miRNAs/novel in table S5/m0189.pdf]

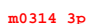[illegible]

m0314\_5p

```
.....acuuaucaugcuggggca..... 1 0 seq
```

Supplement: File S2 — The compressed/ZIP file archive for the predicted precursors' secondary structures and reads alignment. (ZIP) [file pone.0104371.s010.zip › second structure and reads alignment for oyster miRNAs/novel in table S5/m0314.pdf]

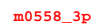[illegible]

Supplement: File S2 — The compressed/ZIP file archive for the predicted precursors' secondary structures and reads alignment. (ZIP) [file pone.0104371.s010.zip › second structure and reads alignment for oyster miRNAs/novel in table S5/m0558.pdf]

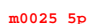

m0025 3p

[illegible]

Supplement: File S2 — The compressed/ZIP file archive for the predicted precursors' secondary structures and reads alignment. (ZIP) [file pone.0104371.s010.zip › second structure and reads alignment for oyster miRNAs/potential in table S7/m0025.pdf]

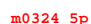

m0324 3p

[illegible]

Supplement: File S2 — The compressed/ZIP file archive for the predicted precursors' secondary structures and reads alignment. (ZIP) [file pone.0104371.s010.zip › second structure and reads alignment for oyster miRNAs/potential in table S7/m0324.pdf]

[illegible]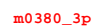[illegible]

Supplement: File S2 — The compressed/ZIP file archive for the predicted precursors' secondary structures and reads alignment. (ZIP) [file pone.0104371.s010.zip › second structure and reads alignment for oyster miRNAs/potential in table S7/m0380.pdf]

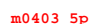

m0403 3p

[illegible]

Supplement: File S2 — The compressed/ZIP file archive for the predicted precursors' secondary structures and reads alignment. (ZIP) [file pone.0104371.s010.zip › second structure and reads alignment for oyster miRNAs/potential in table S7/m0403.pdf]

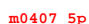

m0407 3p

[illegible]

Supplement: File S2 — The compressed/ZIP file archive for the predicted precursors' secondary structures and reads alignment. (ZIP) [file pone.0104371.s010.zip › second structure and reads alignment for oyster miRNAs/potential in table S7/m0407.pdf]

[illegible]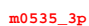[illegible]

Supplement: File S2 — The compressed/ZIP file archive for the predicted precursors' secondary structures and reads alignment. (ZIP) [file pone.0104371.s010.zip › second structure and reads alignment for oyster miRNAs/potential in table S7/m0535.pdf]
